# A palmitoyl transferase chemical–genetic system to map ZDHHC-specific *S*-acylation

**DOI:** 10.1038/s41587-023-02030-0

**Published:** 2024-01-08

**Authors:** Cory A. Ocasio, Marc P. Baggelaar, James Sipthorp, Ana Losada de la Lastra, Manuel Tavares, Jana Volarić, Christelle Soudy, Elisabeth M. Storck, Jack W. Houghton, Susana A. Palma-Duran, James I. MacRae, Goran Tomić, Lotte Carr, Julian Downward, Ulrike S. Eggert, Edward W. Tate

**Affiliations:** 1 The Francis Crick Institute, London, UK; 2 Imperial College London, Department of Chemistry, Molecular Sciences Research Hub, London, UK; 3 King’s College London, Randall Centre for Cell and Molecular Biophysics, School of Basic and Medical Biosciences and Department of Chemistry, London, UK; 4 Present Address: Utrecht University, Biomolecular Mass Spectrometry & Proteomics Group, Utrecht, The Netherlands; 5 Present Address: Department of Food Science, Research Center in Food and Development A.C., Hermosillo, Mexico

**Keywords:** Post-translational modifications, Chemical tools

## Abstract

The 23 human zinc finger Asp-His-His-Cys motif-containing (ZDHHC) *S*-acyltransferases catalyze long-chain *S*-acylation at cysteine residues across an extensive network of hundreds of proteins important for normal physiology or dysregulated in disease. Here we present a technology to directly map the protein substrates of a specific ZDHHC at the whole-proteome level, in intact cells. Structure-guided engineering of paired ZDHHC ‘hole’ mutants and ‘bumped’ chemically tagged fatty acid probes enabled probe transfer to specific protein substrates with excellent selectivity over wild-type ZDHHCs. Chemical–genetic systems were exemplified for five human ZDHHCs (3, 7, 11, 15 and 20) and applied to generate de novo ZDHHC substrate profiles, identifying >300 substrates and *S*-acylation sites for new functionally diverse proteins across multiple cell lines. We expect that this platform will elucidate *S*-acylation biology for a wide range of models and organisms.

## Abstract (web summary)

The substrates of specific *S*-acyltransferases are mapped proteome-wide in live cells.

## Main

**Fig. 1:**
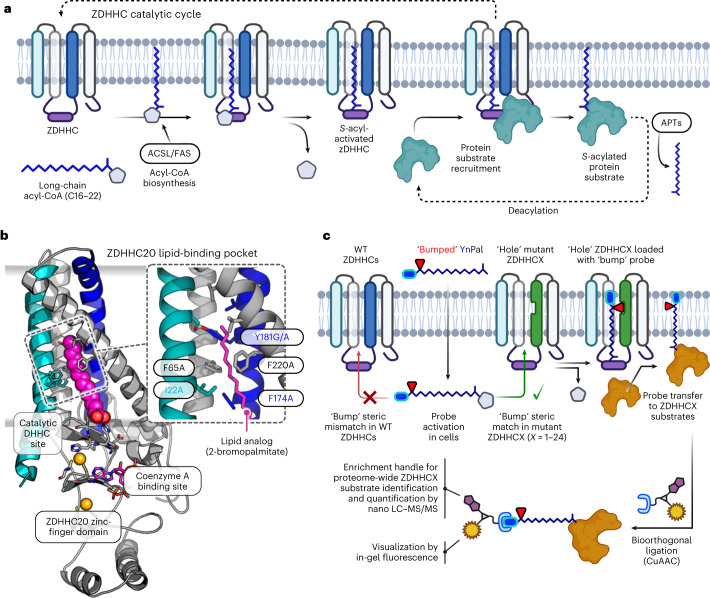
ZDHHC chemical genetics. **a**, *S*-acylation is mediated by ZDHHC loading of long-chain acyl-CoA derived from lipid biosynthesis followed by acyl transfer to a proximal Cys of a protein substrate and regeneration of apo-ZDHHC. The reversible cycle is closed by thioester hydrolysis by APTs. **b**, X-ray structure of human ZDHHC20 irreversibly inhibited by lipid mimic 2-bromopalmitate (PDB ID: 6BML). Inset, sterically demanding residues in the ZDHHC20 lipid-binding pocket contact the acyl chain distal to the DHHC catalytic site. **c**, Steric complementation between a ZDHHC ‘hole’ mutant and an alkyne-tagged ‘bumped’ lipid substrate probe enables selective loading and tag transfer to ZDHHC substrates, bypassing endogenous (WT) ZDHHCs. Fluorescence visualization and chemical proteomics are enabled by bioorthogonal conjugation to multifunctional capture reagents. Source data

The chemical and functional diversity of proteins encoded by the human genome is expanded by orders of magnitude through post-translational modification (PTM)^1,2^, of which long-chain *S*-acylation is among the most widespread. This process is mediated in all eukaryotes by the zinc finger Asp-His-His-Cys motif-containing (ZDHHC) *S*-acyltransferase family of integral membrane enzymes, including 23 known human ZDHHCs that together acylate >3,000 cysteine residues across ca. 12% of the human proteome^3–6^. The ZDHHC catalytic cycle occurs in the following two stages: auto-*S*-acylation of a conserved Cys in the DHHC motif by C14:0 to C22:0 acyl-CoA (commonly palmitoyl (C16:0)-CoA) with release of CoA-SH, followed by *S*-acyl transfer to a substrate protein cysteine proximal to the ZDHHC catalytic site (Fig. 1a,b)^7–9^. Protein substrates lack a consensus sequence beyond the requirement for a free cysteine^10^, and substrate recruitment occurs through colocalization by ZDHHC–protein interactions, membrane-associated domains or prior lipid PTMs^5,11–13^. *S*-acylation increases local hydrophobicity and membrane affinity and can regulate protein membrane microdomain partitioning, stability, trafficking, nuclear localization, secretion and protein interactions^14,15^. De-*S*-acylation by acyl-protein thioesterase (APT; Fig. 1a) can generate a dynamic *S*-acylation cycle implicated in signaling cascades^16–18^, with numerous examples of upregulation or downregulation of *S-*acylation promoting pathological conditions including cancer, inflammatory disease or neurodegeneration^4,19–22^.

Despite the importance of ZDHHCs in health and disease, mapping the substrate network of a specific ZDHHC remains a formidable challenge. Global enrichment of *S*-acylated proteins through metabolic labeling with alkyne-tagged lipid analogs or chemical exchange of *S*-acyl thioesters for affinity tags can circumvent the difficulty of direct *S*-acylated peptide detection by liquid chromatography–mass spectrometry (LC–MS), leading to large databases of putative substrate proteins^6,23,24^. However, the lack of selective ZDHHC inhibitors^25,26^ and the confounding influence of ZDHHC overexpression, knockdown or knockout (KO) that can lead to redundancy, compensation or loss of ZDHHC–protein interactions and coregulation^27–30^ currently prevent the direct association of a specific ZDHHC with its cognate *S*-acylated protein substrates.

Here we establish a chemical–genetic system for direct labeling and identification of the substrates of a specific ZDHHC in intact cells, through steric complementation (so-called ‘bump and hole’; Fig. 1c)^31–33^. We report mutant/probe pairs for five diverse human ZDHHCs (3, 7, 11, 15 and 20) and demonstrate mutant-specific ZDHHC-loading and protein substrate transfer with high selectivity over wild-type (WT) ZDHHCs. Coupled with chemical proteomics, this technology enabled de novo identification of >300 putative ZDHHC-specific substrates and *S*-acylation sites in varied human cell lines and extended substrate networks for ZDHHCs 7, 15 and 20. Adaptability and ease of implementation across cellular models suggest that ZDHHC chemical genetics offers a new platform for systematic investigation of ZDHHC biology, with the potential to catalyze knowledge‐driven selection of ZDHHCs and ZDHHC‐mediated pathways for future therapeutic validation or biomarker discovery.

## Results

### Selective *S*-acylation by an engineered ZDHHC20/probe pair

**Fig. 2:**
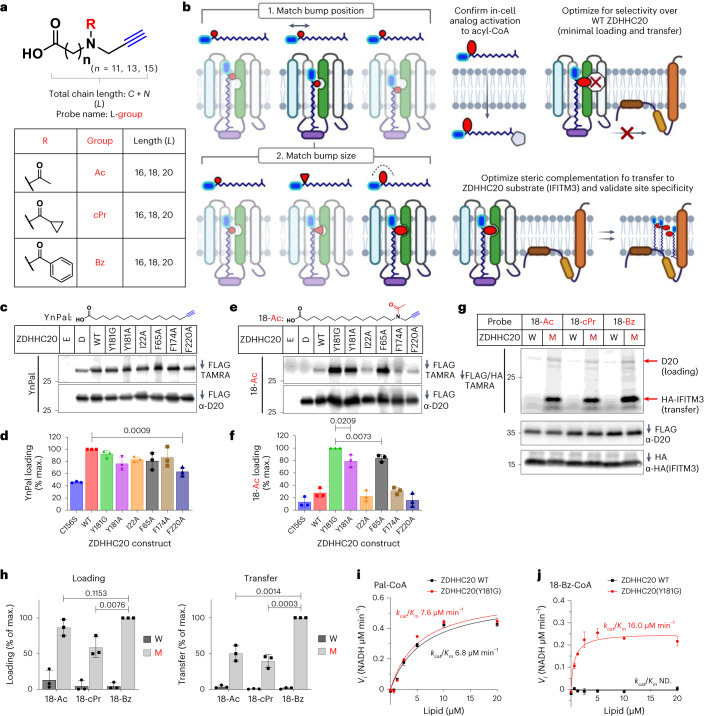
Engineering a ‘bump’ probe and ‘hole’ mutant pair for ZDHHC20. **a**, Fatty acid probes containing an alkynyl click-handle (blue), varying chain length *L* = 16, 18 or 20 heavy atoms in the chain (carbons + nitrogen) and an R ‘bump’ group (red)—Ac, *c*Pr or Bz. **b**, Two-stage pairing strategy for a designed ZDHHC20 mutant optimizes probe chain length and then bump size to match the new binding cavity, with probe activation, selectivity over ZDHHC20 WT and transfer to a known ZDHHC20 substrate (IFITM3) optimized in parallel. **c**–**f**, Bump-hole loading analysis of C-terminal FLAG-tagged ZDHHC20 WT and mutants in HEK293T cells treated with 15 μM YnPal (**c**,**d**) or 18-Ac (**e**,**f**) for 4 h (D, catalytic-dead ZDHHC20(C156S); E, empty vector; *n* = 3 independent biological replicates average ± s.d.). **g**, Probe bump-size optimization by transfer assays with HA-IFITM3 and either WT ZDHHC20 (W) or ZDHHC20(Y181G) (M) co-expression in HEK293T cells (*n* = 3 independent biological replicates average ± s.d.). **h**, Average loading and transfer activity relative to highest fluorescent/input ratio (*n* = 3 independent biological replicates average ± s.d.). **i**,**j**, Enzyme kinetics for WT ZDHHC20 and ZDHHC20(Y181G) treated with Pal-CoA (**i**) or 18-Bz-CoA (**j**) using a KDH assay (*3*). Michaelis–Menten plots generated from average reaction rate (NADH generated μM min^−1^, *n* = 3 independent experiments) ± s.d. versus lipid concentration (μM). **d**,**f**,**h**, The two-tailed unpaired *t* test of Prism 9.0 was used to determine *P* values and noted above relevant comparisons.

Steric complementation imposes stringent requirements on mutant and probe design, which are as follows: mutant ZDHHC should retain WT catalytic activity and protein substrate specificity; the probe must bear both a ‘bump’ and a bioorthogonal tag^34^ and be efficiently activated to the CoA thioester form in the cell, without interfering with endogenous lipid metabolism; the activated probe must be minimally processed by WT ZDHHCs to deliver selectivity for ZDHHC-specific substrate identification. Human ZDHHCs feature a conical four multipass transmembrane (4TM) helix lipid-binding domain adjacent to the cytosolic catalytic site^3,35^. Reasoning that mutations distal to the DHHC active site would minimize interference with catalytic activity and lipid probe activation, we used the reported structure of ZDHHC20 to design ‘hole’ mutations toward the 4TM apex (Fig. 1c). A panel of nine alkyne-tagged bumped lipid analogs were designed and synthesized, positioning small (acetyl, Ac), medium (cyclopropanecarbonyl, *c*Pr) or large (benzoyl, Bz) bump groups at increasing distance from the acid (Fig. 2a and Methods), encompassing the most common *S*-acylation chain lengths (16, 18 or 20 atoms)^8^. This design enables systematic bump pairing to ZDHHC mutant ‘hole’ size and position, while the bioorthogonal alkyne tag permits ligation of fluorescent reporters and/or affinity handles to modified proteins by copper-catalyzed alkyne-azide cycloaddition (CuAAC; Fig. 1c), revealing ZDHHC autoacylation and substrates in cellular assays. Probe/mutant optimization was envisaged in two stages, determining bump placement followed by optimal bump size (Fig. 2b).

**Extended Data Fig. 1:**
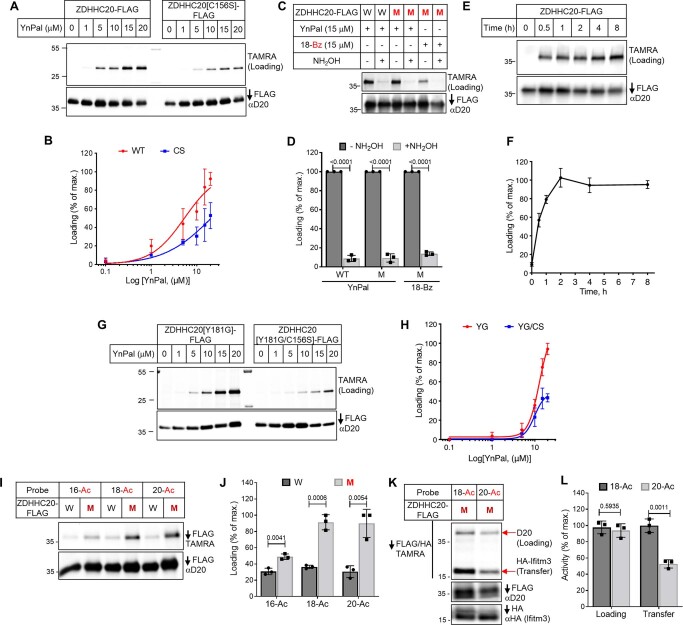
Establishing assay conditions to measure ZDHHC20 lipid-loading. (**a-b**) Catalytically dead ZDHHC20 is appreciably labeled by YnPal at peripheral cysteine sites. FLAG-tagged WT and ZDHHC20[C156S] constructs were transfected in HEK293T cells and treated with the indicated concentration of YnPal for 4 h. After lysis and IP with anti-FLAG resin, samples were subjected to CuAAC with TAMRA azide and separated by SDS-PAGE. ZDHHC20 loading and input were measured by in-gel fluorescence and anti-ZDHHC20 immunoblot (n = 3 independent biological replicates). (**c-d**) Thioester dependence of ZDHHC20 labeling was demonstrated upon treatment of YnPal and C18-Bz treated samples with 0.8 M neutralized NH_2_OH following IP and CuAAC with TAMRA azide (n = 3 independent biological replicates). (**e-f**) Time-course measuring 15 µM YnPal labeling of ZDHHC20 WT expressing HEK293T cells (n = 3 independent biological replicates). (**g-h**) Labeling activity of the indicated concentrations of YnPal in FLAG-tagged ZDHHC20[Y181G] and ZDHHC20[Y181G/C156S] expressing HEK293T cells (n = 3 independent biological replicates). The average (n = 3 independent biological replicates) loading (**b**, **d**, **f** and **h**) was reported as a percent of the maximal fluorescent: input ratios ± S.D. between treatments with and without hydroxylamine. (**i-l**) Probe chain-length was optimized against ZDHHC20[Y181G] using cell-based loading (**i-j**) and transfer (**k-l**) assays in HEK293T using ZDHHC20 WT (W) and ZDHHC20[Y181G] (M). (**i**) HEK293T cells were treated with 15 µM acetyl bumped probes of L = 16, 18 and 20 for 4 h and enzyme loading assessed by in-gel fluorescence following anti-FLAG IP and CuAAC with TAMRA azide (n = 3 independent biological replicates). (**k**) HEK293T cells co-expressing ZDHHC20[Y181G] and HA-Ifitm3 were treated with 15 µM 18-Ac or 20-Ac for 4 h with loading and transfer of the probe assessed following by anti-FLAG/anti-HA IP and CuAAC with TAMRA azide (n = 3 independent biological replicates). (**j-l**) The average (n = 3 independent biological replicates) loading and transfer activity were reported as a percent of the maximal fluorescent/input ratios ± S.D. The two tailed unpaired t-test of Prism 9.0 was used to determine p-values and are note above relevant comparisons.

Baseline autoacylation activity for each FLAG-tagged ZDHHC20 mutant was determined by metabolic labeling with YnPal (an alkyne-tagged analog of palmitate, C16:0) in HEK293T cells (Fig. 2c,d)^23^. FLAG immunoprecipitation (IP) followed by CuAAC ligation to tetramethylrhodamine (TAMRA) and analysis by in-gel fluorescence confirmed autoacylation activity for all mutants, with ZDHHC20(Y181G) showing labeling equivalent to WT. Residual acylation for catalytically dead Cys mutant ZDHHC20(C156S) (lane ‘D’; Fig. 2c,d and Extended Data Fig. 1a,b) was consistent with previously characterized *S*-acylation at noncatalytic ZDHHC20 Cys residues mediated by endogenous ZDHHCs^24,36^. Labeling was sensitive to thioester cleavage by hydroxylamine (HA) and increased with YnPal concentration and incubation time, leading to steady-state labeling after 2 h of incubation with 15 µM YnPal (Extended Data Fig. 1a–h). An initial screen of ZDHHC20 mutants under similar conditions against bumped probe 18-Ac (18-atom chain length, smallest bump) revealed strong complementation for F65 and Y181 mutants and reduced loading with WT ZDHHC20, with Y181G exhibiting fivefold higher loading than WT (Fig. 2e,f). Furthermore, residual ZDHHC20(C156S) labeling was suppressed to the background, suggesting that the bumped probe is a poor substrate for endogenous ZDHHCs that *S*-acylate ZDHHC20 in *trans*. These data, consistent with previous evidence that mutations in the 4TM domain can tolerate longer chain lipids^3,8^, encouraged us to proceed to optimize steric complementation with ZDHHC20(Y181G).

Structure-guided screening commenced with the smallest ‘bump’ probes of increasing chain length (16-Ac, 18-Ac or 20-Ac) to identify the length register matching the bump to the mutant cavity (Fig. 2b and Extended Data Fig. 1i,j). Both lipid loading and transfer to the substrate were assayed together by co-expressing ZDHHC20-FLAG (WT or Y181G) with HA-tagged canonical ZDHHC20 substrate IFITM3 (refs. ^21,37^), enabling sensitive in-gel fluorescence quantification of ZDHHC20 and protein substrate labeling following dual FLAG/HA IP and on-bead CuAAC ligation to TAMRA-azide. Chain lengths 18-Ac and 20-Ac were superior to 16-Ac in ZDHHC20(Y181G) loading, with 18-Ac showing twofold higher transfer over 20-Ac (Extended Data Fig. 1k,l), implying improved catalytic efficiency. Bump-size screening (Ac, *c*Pr or Bz) at the 18-atom length identified 18-Bz as an optimal probe for ZDHHC20(Y181G), exhibiting >20-fold higher loading and >60-fold more efficient transfer than WT ZDHHC20 (Fig. 2g,h).

### Orthogonal catalytic efficiency by steric complementation

**Extended Data Fig. 2:**
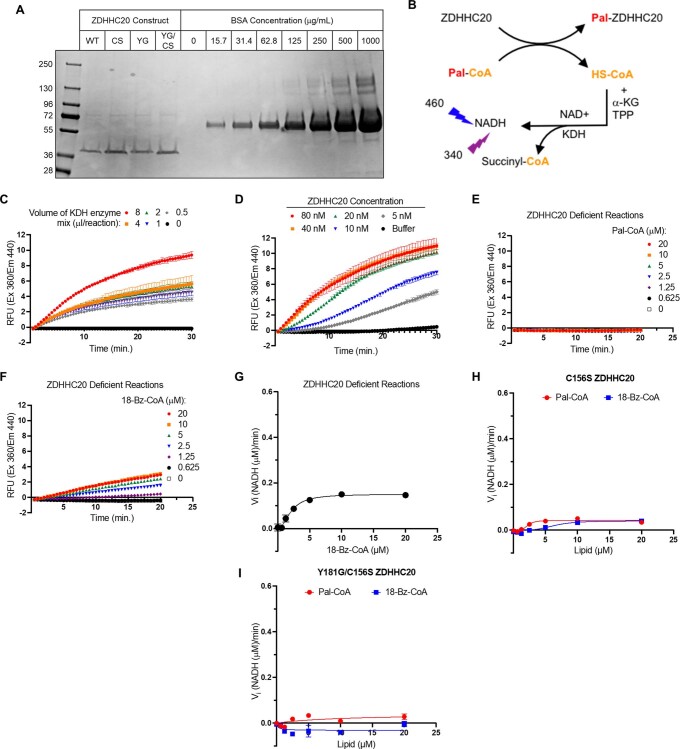
Establishing kinetic parameters for an optimal Y181G-ZDHHC20 bump probe pair. (**a**) Wild-type (WT), Y181G (YG), C156S (CS) and Y181G/C156S (YGCS) FLAG-tagged ZDHHC20 constructs were transfected into HEK293T cells and purified by anti-FLAG agarose affinity chromatography. After enzyme elution with 3X FLAG-peptide, buffer was exchanged using 50 kDa M.W. cut-off protein concentrator tubes and sample concentration determined using a BSA standard curve. All samples were run on SDS-PAGE gels and protein visualized by Coomassie staining (n = 2 independent experiments). (**b**) An enzyme-coupled assay monitoring ZDHHC20 autoacylation was established using commercial α-ketoglutarate dehydrogenase enzyme (KDH) along with its substrates α-ketoglutarate (α-KG), thiamine pyrophosphate (TPP) and NAD+. Optimization of α-ketoglutarate dehydrogenase (KDH) (**c**) and WT ZDHHC20 (**d**) concentrations. Pal-CoA (**e**) and 18-Bz-CoA (**f**) KDH activities were determined in the absence of ZDHHC20, to establish background rates for each probe. (**g**) 18-Bz-CoA displayed significant background activity in the KDH assay without ZDHHC20. Reaction rates for ZDHHC20[C156S] (**h**) and ZDHHC20[Y181G, C156S] (**i**) treated with Pal-CoA or 18-Bz-CoA. Michaelis-Menten plots generated by plotting average (n = 3 independent experiments) reaction rates (NADH generated (µM)/min) ± S.D.) versus lipid concentration (µM) using Prism 9.0. For reactions with 18-Bz-CoA, the basal rates at all concentrations tested were subtracted from the corresponding total reaction rates.

Enzyme kinetic parameters of activated 18-Bz CoA thioester (18-Bz-CoA) and YnPal-CoA were measured for recombinant FLAG-purified WT ZDHHC20 or ZDHHC20(Y181G) using a real-time enzyme-coupled assay, measuring CoA generation during spontaneous auto-*S*-acylated ZDHHC20 turnover in the absence of a protein substrate (Supplementary Table 1)^3,38^. Consistent with cellular assay data, YnPal-CoA had similar catalytic efficiency (*k*_cat_/*K*_*M*_) for WT and ZDHHC20(Y181G) (6.8 ± 0.3 and 7.6 ± 0.3 μM min^−1^, respectively; Fig. 2i and Supplementary Table 1). Furthermore, 18-Bz-CoA had even greater catalytic efficiency with ZDHHC20(Y181G) (16.0 ± 1.0 μM min^−1^), with slightly reduced *k*_cat_ and *K*_*M*_ relative to YnPal-CoA, while showing no measurable activity with WT ZDHHC20 (Fig. 2j and Supplementary Table 1). As expected, catalytically dead ZDHHC20(C156S) and (Y181G/C156S) mutants were inactive in this assay (Extended Data Fig. 2). Together, these data provide compelling biochemical evidence that designed ZDHHC steric complementation delivers orthogonal ZDHHC loading at an efficiency comparable to WT.

### De novo ZDHHC20 substrate discovery by chemical genetics

**Extended Data Fig. 3:**
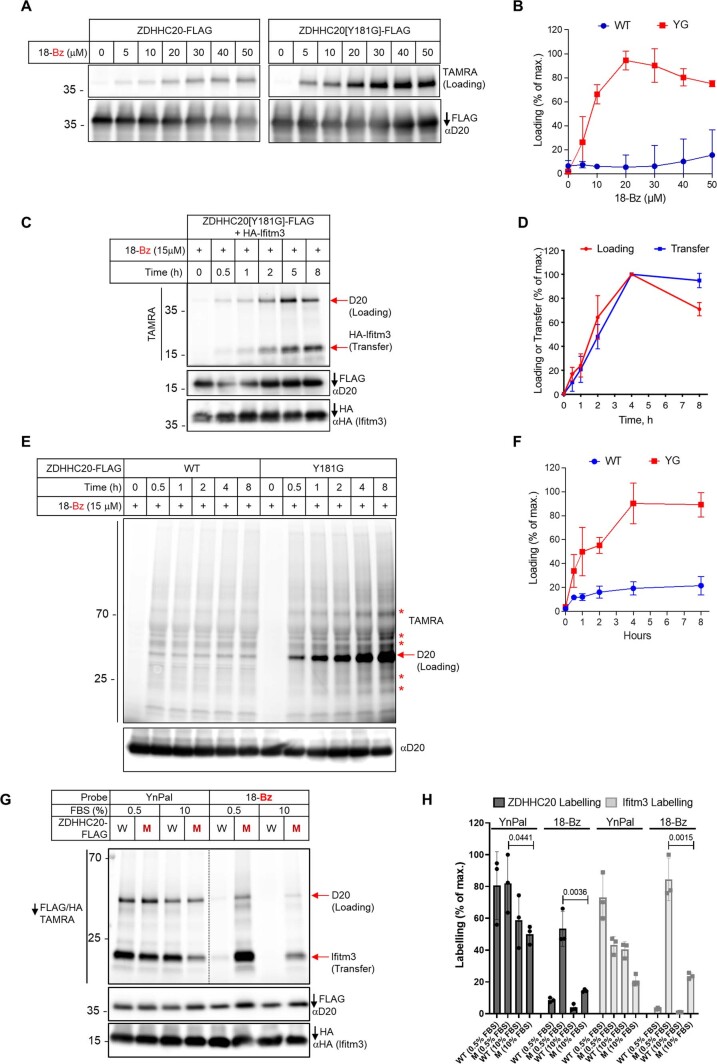
Optimization of conditions for characterization of substrates using 18-Bz in ZDHHC20[Y181G] expressing cells. FLAG-tagged ZDHHC20 WT and ZDHHC20[Y181G] expressing HEK293T cells were treated with the indicated concentration of 18-Bz (**a-b**) for 4 h in cell-based loading assays (n = 3 independent biological replicates). (**c-d**) FLAG-tagged ZDHHC20[Y181G] and HA-Ifitm3 expressing HEK293T cells were treated with 15 µM 18-Bz for the indicated time in cell-based loading and transfer assays (n = 3 independent biological replicates). (**e-f**) FLAG-tagged ZDHHC20 WT and ZDHHC20[Y181G] expressing HEK293T cells were treated with 15 µM 18-Bz for the indicated times (n = 3 independent biological replicates). Lysates were clicked with TAMRA azide then analyzed by in-gel fluorescence and SDS-PAGE; note YG-dependent labeling of substrate protein bands (*). Input was assessed by anti-ZDHHC20 (D20) immunoblot. The average (n = 3 independent biological replicates) loading (**b, d** & **f**) and transfer (**d**) were reported as a percent of the maximal fluorescent: input ratios ± S.D. (**g-h**) The effect of FBS concentration on ZDHHC20 loading and transfer. (**g**) FLAG-tagged wild-type (WT) or ZDHHC20[Y181G] (M) and HA-Iftim3 expressing HEK293T cells were treated with 15 µM YnPal or 18-Bz in the presence of 0.5 or 10% FBS for 4 h in cell-based transfer assays. (**h**) The average (n = 3 independent biological replicates) loading and transfer were reported as a percent of the maximal fluorescent: input ratio ± S.D. The two tailed unpaired t-test of Prism 9.0 was used to determine p-values and noted above the relevant comparisons.

**Extended Data Fig. 4:**
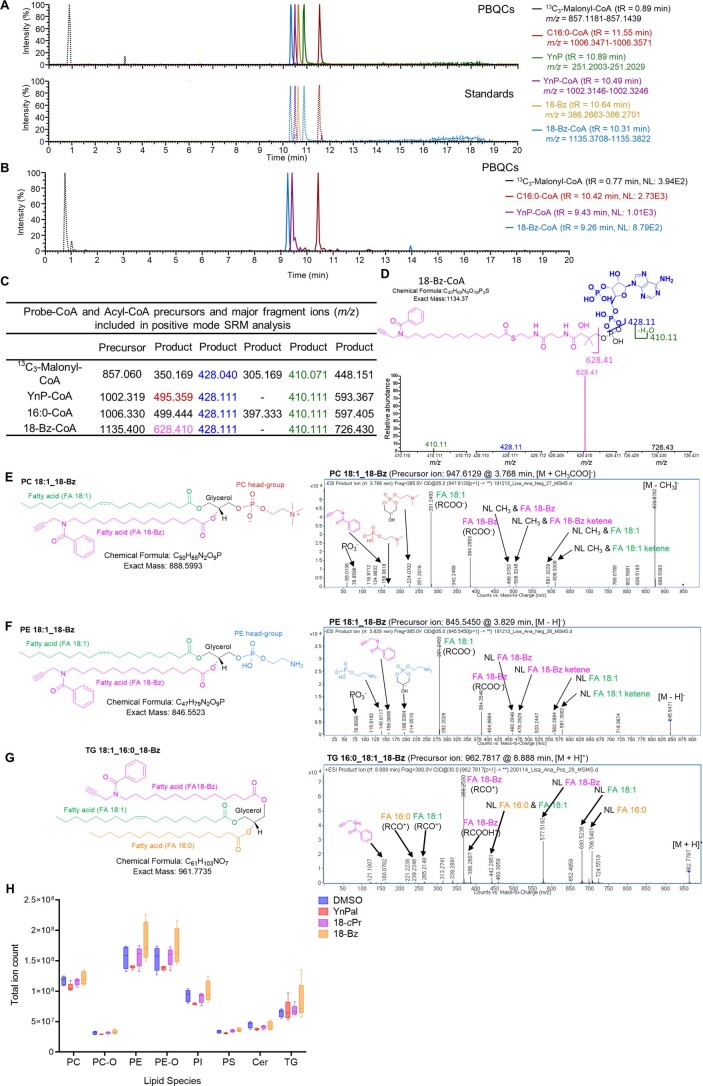
Metabolomic analysis of HEK293T cells. HEK293T cells were treated with 30 µM YnPal or 18-Bz for 2 h in media containing 0.5% FBS. Cells were lysed, and metabolites extracted (see Supplementary Methods, Metabolomics). Polar metabolites (including probe and probe-CoA molecules) were analyzed by LC-MS. (**a**) Probes and probe-CoA molecules were detected by LC-HRMS in positive and negative modes. Features from sample pooled biological controls (PBQCs, solid lines, upper panel) and authentic standards (dotted lines, lower panel) had identical retention times (shown). ^13^C_3_-Malonyl-CoA (black dotted line) was used as an internal standard. Retention times (tR) and mass ranges *(m/z*) used for each molecule are shown. (**b-e**) Probe-CoA identifications were confirmed by LC-MS/MS in positive mode. **(b)** Panel shows an LC-MS/MS chromatogram of sample PBQCs, using selected ion monitoring (SRM) specific for probe-CoAs. Retention times (tR) and normalized intensities (NL) for each molecule are shown. **(c)** SRM transitions (precursor and most abundant product [M + H]^+^ ions) used for identification of probe-CoAs. (**d**) Upper panel show chemical structures, formulae, and exact masses of 18-Bz-CoA. Lower panel show spectra of major LC-MS/MS product ions for 18-Bz-CoA, with colors equivalent to fragmentations depicted in the upper panel. Product ion of *m/z* 726.43 (18-Bz-CoA) is equivalent to the ion *m/z* 628.41, but where an acyl chain moiety is retained. Abundance is shown relative to the most abundant product ion (*m/z* 628.41 for 18-Bz-CoA. (**e-i**) Lipidomic analysis of cells treated with 18-bump series. HEK293T cells were treated with DMSO and 15 µM YnPal, 18-*c*Pr and 18-Bz for 4 h. After treatment, cells were lysed, extracted with *tert*-butyl methyl ether/methanol/water and sample lipids analyzed by UHPLC-MS/MS in the positive and negative mode. (**e-g**) Chemical structures and MS/MS spectra of selected PC, PE and TG lipid species incorporating an 18-Bz lipid side-chain. PC and PE spectra were acquired in negative polarity and TG spectrum was acquired in positive polarity. Note that TG species with an 18-Bz side chain preferentially formed the [M + H]+ ion, in contrast to endogenous TG species where the predominant ion forms are [M + NH3]+ and [M+Na]+, presumably due to facile protonation at the 18-Bz amide moiety. (**h**) Summed ion intensity of all endogenous lipid species identified within each major lipid class plotted using Prism 9.0. Box and whisker plots represent median values (center lines) and 25th and 75th percentiles (box limits) with Tukey whiskers, n = 5 independent experiments. FA, fatty acid; NL, neutral loss; PC, phosphatidylcholine; PE, phosphatidylethanolamine; TG, triacylglycerol; PI, phosphatidylinositol; PS, phosphatidylserine; Cer, ceramide. PC-O, ether-linked PC; PE-O, ether-linked PE.

**Extended Data Fig. 5:**
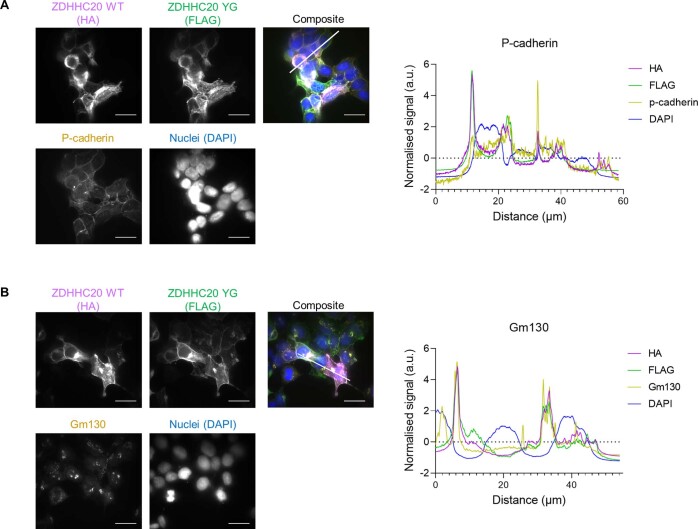
Localization of ZDHHC20 WT and Y181G mutant in HEK293T cells by overexpression. (**a**) Left: representative confocal microscopy images showing average signal of Z-stacks of HEK293T cells transiently co-expressing ZDHHC20 WT HA-tagged and Y181G mutant FLAG-tagged. Each image shows signal for HA (magenta), FLAG (green), p-cadherin as plasma membrane marker (yellow), nucleus (blue) and a composite image of all signals. Scale bar at the bottom right marks 20 mm, while the other white line highlights the region of interest (ROI) used for image analysis. Right: plot showing normalized fluorescence signal for each of the channels in the ROI (1 biological replicate). (**b**) As **a**, but with using Gm130 as Golgi marker (1 biological replicate).

Chemical–genetic complementation offers an opportunity to discover ZDHHC/substrate networks de novo through chemical proteomics, by coupling metabolic labeling to enrichment and quantitative proteomics. Loading and transfer selectivity for 18-Bz/ZDHHC20(Y181G) over 18-Bz/WT ZDHHC20 were optimized with respect to probe concentration (15 μM 18-Bz) and time (8 h; Extended Data Fig. 3). Direct 18-Bz to 18-Bz-CoA conversion in cells was confirmed by liquid chromatography coupled to tandem mass spectrometry (LC–MS/MS) analysis of extracted metabolites, while lipidomic analyses revealed 18-cPr and 18-Bz incorporation into structural and storage lipids consistent with in situ activation to the CoA ester, and no significant perturbation of endogenous lipid classes relative to vehicle or YnPal-treated cells (Extended Data Fig. 4). Confocal immunofluorescence microscopy demonstrated that transfected ZDHHC20(Y181G) and WT ZDHHC20 colocalize primarily in the Golgi and plasma membrane (Extended Data Fig. 5), consistent with previously reported data on ZDHHC20 cellular localization that also used overexpression constructs due to the absence of reliable antibodies for imaging endogenous ZDHHC20 (refs. ^39,40^).

**Fig. 3:**
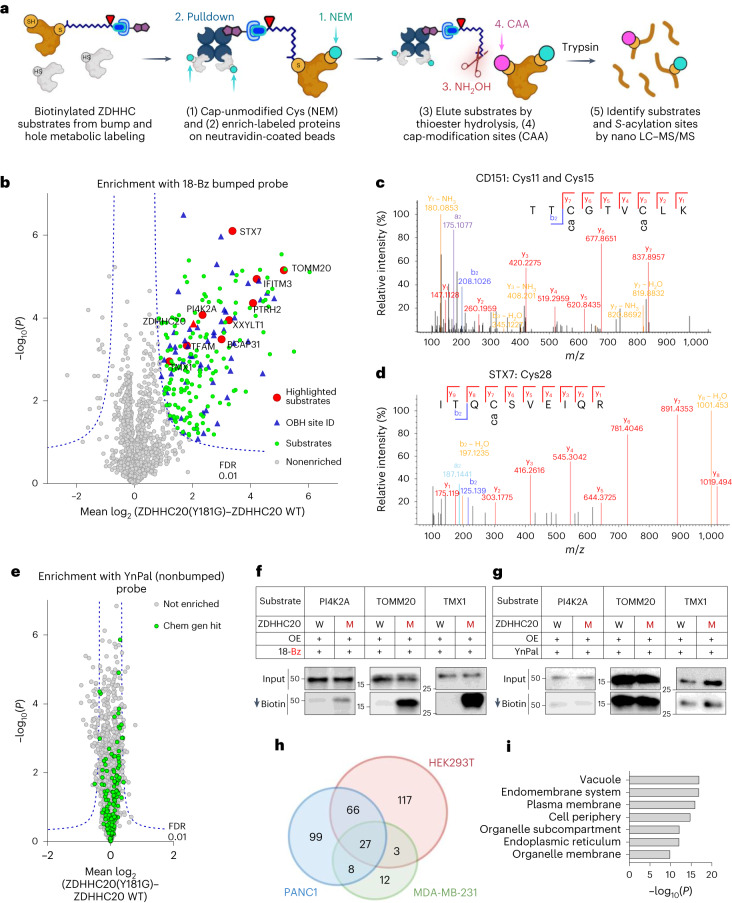
Chemical proteomic ZDHHC20 substrate and modification site identification. **a**, Chemical proteomic OBH workflow for enrichment and identification of *S*-acyltransferase substrates and *S*-acylation sites by LC–MS/MS. **b**, Chemical proteomic analysis of ZDHHC20 substrates in HEK293T cells (15 µM 18-Bz, 8 h). Enrichment in ZDHHC20(Y181G) cells over WT ZDHHC20 reveals selective ZDHHC20 loading (red triangle), and significantly enriched substrates (green circles) selected for further validation (red circles), with site identification data (blue triangles; Student’s two-tailed unpaired *t* test, *S*_0_ = 0.5, adjusted FDR = 0.01, *n* = 4 independent biological replicates per condition). **c**,**d**, LC–MS/MS spectrum corroborating reported sites of CD151 (**c**) *S*-acylation at Cys11 and Cys15 and of STX7 (**d**) *S*-acylation at Cys28 (see also Extended Data Fig. 10). **e**, *S*-acylated proteome profiling using YnPal. HEK293T cells transiently transfected with WT ZDHHC20 or ZDHHC20(Y181G) were treated with 15 µM YnPal for 8 h before processing using the on-bead digestion workflow. Substrates highlighted in green had been identified using a chemical–genetic system (Student’s two-tailed unpaired *t* test, *S*_0_ = 0.5, adjusted FDR = 0.01, *n* = 4 independent biological replicates per condition). **f**,**g**, Validation of *S*-acylation for substrates at endogenous levels. HEK293T cells transiently transfected with WT ZDHHC20 (W) or ZDHHC20(Y181G) (M) were treated with 15 µM 18-Bz (**f**) or 15 µM YnPal (**g**) for 24 h. Lysates were clicked with biotin azide before enrichment on neutravidin magnetic beads. Representative immunoblots are shown for input and pull-down signals (*n* = 2 independent biological replicates). **h**, Venn diagram of putative ZDHHC20 substrates identified in HEK293T, MDA-MB231 and PANC1 cells. **i**, Statistical overrepresentation analysis of putative ZDHHC20 substrate cellular compartment (Slim)-GO terms compared to the full human genome using the PANTHER classification system showing terms with >9 −log_10_(*P* value) from an FDR-adjusted two-tailed Fisher’s exact test.

**Extended Data Fig. 6:**
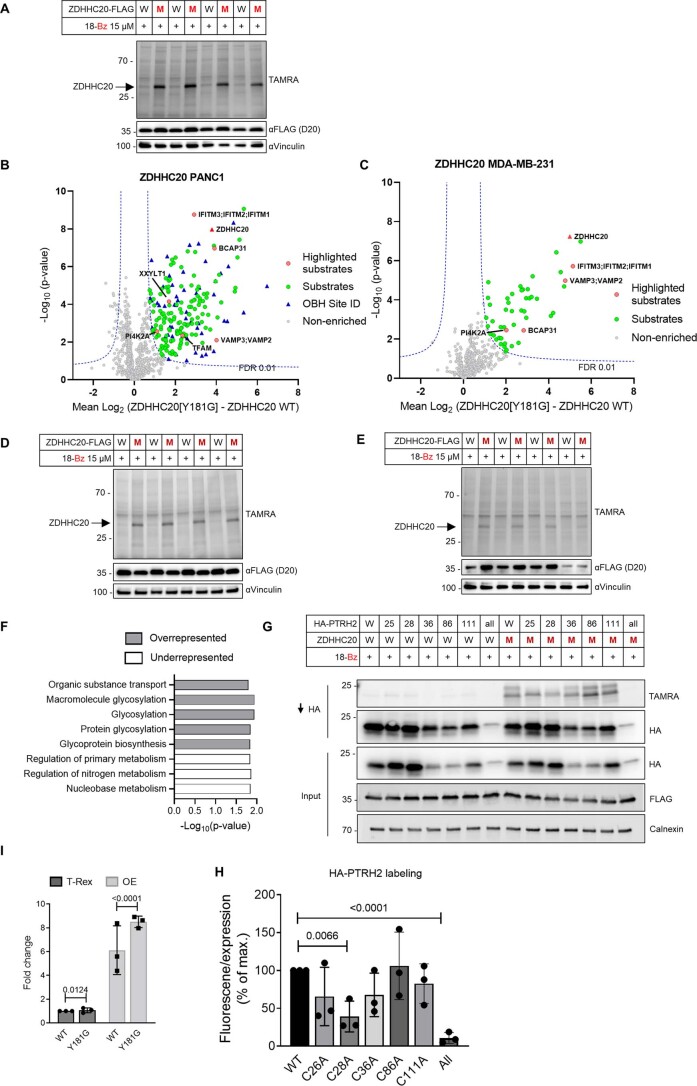
ZDHHC substrate profiling in different cell lines. (**a**) Full gel and western blots of all replicates corresponding to the chemical proteomics ZDHHC20 substrate identification (Fig. 3b) performed in HEK293T cells. A portion of the lysate was clicked with TAMRA azide for analysis by in-gel fluorescence. The bands present at ~35 kDa in ZDHHC20[Y181G] (M) lanes but absent in WT ZDHHC20 lanes indicate selective loading of 18-Bz on ZDHHC20[Y181G] over WT-ZDHHC20. Anti-FLAG WB indicates similar expression levels of WT construct compared to ZDHHC20[Y181G] construct. Vinculin is used as loading control (n = 4 independent biological replicates). (**b-e**) Chemical proteomics ZDHHC20 substrate detection with 18-Bz probe (15 µM) in (**b**) PANC1 cells and (C) MDA-MB-231 cells. Cells were transiently transfected with WT ZDHHC20 versus ZDHHC20[Y181G] (M) then clicked with biotin azide and enriched on neutravidin agarose for proteomic processing. Significantly enriched putative substrates (Student’s two tailed unpaired t-test S0 - 0.5, adjusted FDR - 0.01) are shown as green circles, hits with site identification data are shown in as blue triangles and other validated substrates are highlighted as red circles. 200 putative ZDHHC20 substrates are identified in (B) PANC1 cells and 50 putative substrates in (**c**) MDA-MB-231 cells. (**d-e**) Gel and western blots corresponding to the volcano plot in (**a-b**) where a portion of the lysate was clicked with TAMRA azide as described in B (n = 4 independent biological replicates). **(f)** Statistical over/underrepresentation analysis of putative ZDHHC20 substrate biological process GO-terms compared to a reference list containing reported *S*-acylated proteins (SwissPalm) using the PANTHER classification system showing terms with >1.5 -Log_10_(p-value) from an FDR adjusted Fisher’s exact two tailed test. **(g-h)** PTRH2 Site ID analysis and quantification. **(g**) Validation of HA-PTRH2 *S*-acylation by ZDHHC20 using the bumped probe 18-Bz and *S*-acylation site mutants. Representative images (n = 3 independent biological replicates) for TAMRA signal are shown, as well as for HA and FLAG immunoblots for HA pull down and input. Calnexin was used as loading control. **(h**) Bar plot showing the ratio of TAMRA fluorescence and HA pulldown signal of PTRH2 cysteine mutants as a percentage of WT PTRH2 ratio. The two tailed unpaired t-test statistical module of Prism 9.0 was used to calculate p-values and noted above relevant comparisons. **(i)** Profiling of Flp-in T-Rex substrates ZDHHC20 cell lines. The average (n = 3 independent biological replicates) Fold change of FLAG signal is reported as a percent of the maximal ratios ± S.D. The unpaired t-test statistical module of Prism 9.0 was used to determine p-values and noted above relevant comparisons. Related to main Fig. 5b.

We sought to discover substrates modified by ZDHHC20(Y181G) de novo through comparative quantitative MS-based analysis of proteins labeled by 18-Bz in HEK293T cells expressing either ZDHHC20(Y181G) or WT ZDHHC20 (ref. ^24^), combined with on-bead thioester hydrolysis (OBH) and differential cysteine capping for *S*-acylation site identification (Fig. 3a)^41^. Label-free quantification (LFQ) revealed that 213 proteins were significantly enriched from HEK293T cells expressing ZDHHC20(Y181G) but not in ZDHHC20 WT (Fig. 3b and Extended Data Fig. 6a), with IFITM3 among the most significantly enriched, demonstrating identification of endogenous ZDHHC20 substrates. Ninety-nine potential *S*-acylation sites were identified (Fig. 3b,c and Supplementary Data 1 and 2), including ZDHHC20 auto-*S*-acylation^36^ and 28 sites previously reported in targeted and global *S*-acylation studies, for example, CD151 Cys11 and Cys15 (Fig. 3c) and STX7 Cys28 (Fig. 3d), consistent with detection of genuine *S*-acylation sites (Supplementary Fig. 1)^6,42^. Notably, differential enrichment was strictly dependent on the presence of the bump in the lipid probe (Fig. 3e and Supplementary Data 3). We further validated selective chemical–genetic labeling of a range of substrates by western blot (Fig. 3f,g), confirming chemical–genetic identification of ZDHHC substrates at endogenous abundance.

We further extended chemical proteomic substrate identification to MDA-MB-231 and PANC1 cell lines, identifying 50 and 200 substrates, respectively, alongside 89 sites of modification (Extended Data Fig. 6b–e and Supplementary Data 1 and 2), underscoring the versatility and adaptability of the system. While 104 substrates were common to at least two of three cell lines (HEK293, MDA-MB-231 and PANC1; Fig. 3h), we also identified unique sets in individual cell lines, which may indicate differences in substrate expression or context-specific regulation of *S*-acylation by ZDHHC20. Substrates with endomembrane compartment, endoplasmic reticulum (ER), plasma membrane and intracellular vesicle localization were enriched relative to the reference human genome, consistent with the cellular localization of ZDHHC20 (Fig. 3i)^18,39,40^, and enriched in transport and glycosylation functional annotation compared to an *S*-acylated protein reference database (Extended Data Fig. 6f and Methods)^6^, consistent with a differentiated set of ZDHHC20 substrates.

### Chemical–genetic discovery of new sites of *S*-acylation

**Fig. 4:**
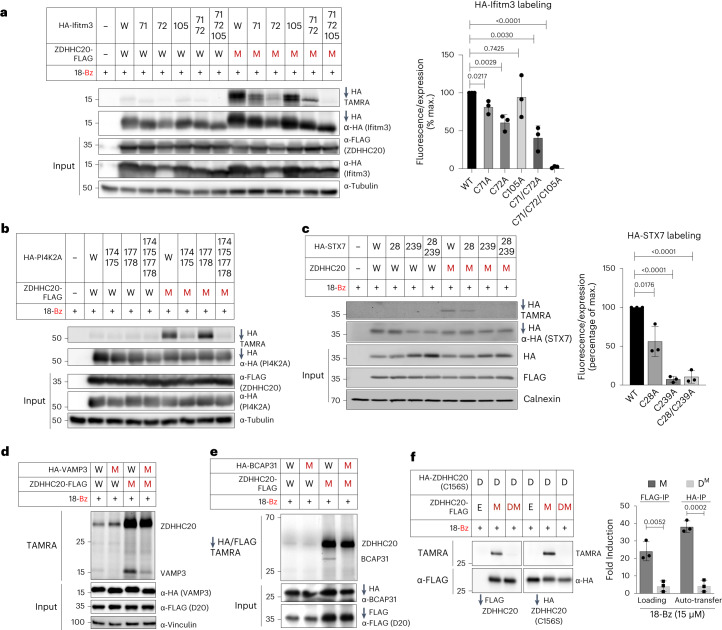
ZDHHC20 substrate and *S*-acylation site analysis. **a**,**b**, ZDHHC20(Y181G) retains exquisite selectivity for specific cysteines on substrates IFITM3 (**a**) and PI4K2A (**b**; *n* = 3 independent biological replicates average ± s.d.), matching previously reported labeling, with the 18-Bz bumped probe. **c**, Validation of HA-STX7 *S*-acylation by ZDHHC20 using the bumped probe 18-Bz and *S*-acylation site mutants (C28A) and (C239A). Representative images (*n* = 3 independent biological replicates average ± s.d.) for TAMRA signal are shown, as well as for HA and FLAG immunoblots for HA pull down and input. Calnexin was used as loading control. **d**,**e**, Validation of HA-VAMP3 and HA-BCAP31 site *S*-acylation by ZDHHC20 using the bumped probe 18-Bz and *S*-acylation site mutants, VAMP3(C76A) and BCAP31(C23A). **d**, Cell-based transfer assays were performed without FLAG-ZDHHC20 and HA-VAMP3 enrichment, but rather with direct labeling of cell lysates by TAMRA-azide click followed by SDS–PAGE and anti-HA, anti-FLAG and anti-vinculin immunoblot analysis. **e**, FLAG-ZDHHC20 and HA-BCAP31 constructs were enriched before TAMRA-azide click labeling. **f**, Confirmation of *trans*-auto-*S*-acylation in peripheral cysteines on a catalytically dead C-HA-ZDHHC20(C156S) (D) by a mutant C-FLAG-ZDHHC20(Y181G) (M) with 15 μM 18-Bz. Catalytically dead C-FLAG-ZDHHC20(Y181G) (DM) did not transfer the probe to D. Cells transfected with an empty vector (E) were used as negative control. HA- and FLAG-tagged ZDHHC20 constructs were transiently cotransfected into HEK293T cells and treated with 15 μM 18-Bz for 4 h. After cell lysis, constructs were separately enriched on anti-HA and anti-FLAG resins, clicked with TAMRA-azide and separated by SDS–PAGE. Loading and input were visualized by in-gel fluorescence and immunoblot, respectively. The average (*n* = 3 independent biological replicates) loading and transfer activity were reported as a percent of the maximal fluorescent:input ratios ± s.d. **a**,**c**,**f**, The two-tailed unpaired *t* test of Prism 9.0 was used to determine *P* values and noted above relevant comparisons.

We next explored conservation of site-specificity of chemical–genetic *S*-acylation compared to WT ZDHHC20-mediated *S*-acylation of established *S*-acylated substrates IFITM3 and PI4K2A by Ala scanning mutagenesis of known *S*-acylated cysteines. 18-Bz/ZDHHC20(Y181G) labeling showed specific *S*-acylation patterns exactly in line with those previously reported for IFITM33 and PI4K2A (Fig. 4a,b)^43,44^. We further validated new ZDHHC20(Y181G) substrate *S*-acylation sites by Ala scanning mutagenesis analysis of individual or all Cys residues for STX7 (Fig. 4c) and PTRH2 (Extended Data Fig. 6g,h), confirming the importance of STX7 Cys28 and Cys239, and PTRH2 Cys28 for *S*-acylation. We further mutated the sole cysteines of VAMP3 and BCAP31 to Ala and confirmed new *S*-acylation sites for VAMP3 at Cys76 (Fig. 4d) and BCAP31 at Cys23 (Fig. 4e).

Interestingly, several ZDHHC20 sites (for example, Cys263) were also identified, suggesting that ZDHHC20 may auto-*S*-acylate in *trans* at noncatalytic sites. To test this hypothesis, we compared the labeling of active versus catalytically inactive ZDHHC20 in HEK293T cells cotransfected with orthogonally HA-tagged inactive ZDHHC20(C156S) (D), and either empty vector (E), FLAG-tagged active (M) or inactive (D^M^) ZDHHC20(Y181G). IP using anti-FLAG or anti-HA beads showed that only active ZDHHC20(Y181G) is loaded with 18-Bz and can transfer probe to the HA-tagged inactive counterpart, confirming auto-*S*-acylation of ZDHHC20 in *trans* (Fig. 4f).

### Efficient substrate profiling at low ZDHHC mutant expression

**Fig. 5:**
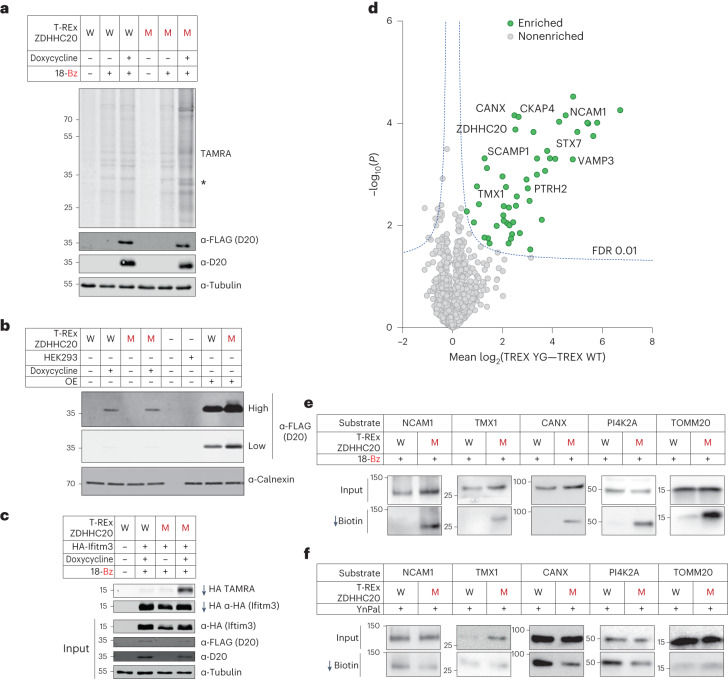
Chemical–genetic analysis under inducible low-expression of ZDHHC20(Y181G). **a**, Profile of WT ZDHHC20 (W) or ZDHHC20(Y181G) Flp-In 293 T-REx cell lines treated with 18-Bz (15 µM, 24 h). Lysates were clicked with TAMRA azide and then analyzed by in-gel fluorescence and SDS–PAGE. Note that the asterisk represents YG-dependent labeling of substrate protein bands. **b**, Comparison of protein expression levels between doxycycline induction of Flp-In 293 T-REx cells and overexpression by transient expression in HEK293T cells. Representative immunoblots are shown for FLAG at high or low exposure, to probe for ZDHHC20 WT versus ZDHHC20(Y181G), and calnexin as loading control (*n* = 3 independent biological replicates). **c**, In Flp-In 293 T-REx cells ZDHHC20(Y181G) retains exquisite selectivity for its substrate IFITM3 with the 18-Bz bumped probe, as seen in prior experiments. **d**, Chemical proteomic analysis of ZDHHC20 substrates in Flp-In 293 T-REx cells (15 µM 18-Bz, 24 h). Enrichment in T-REx ZDHHC20(Y181G) cells over T-REx WT ZDHHC20 reveals selective ZDHHC20 modification of substrates (green) (Student’s two-tailed unpaired *t* test, *S*_0_ = 0.5, adjusted FDR = 0.01, *n* = 4 independent biological replicates per condition). **e**,**f**, Validation of *S*-acylation for T-REx ZDHHC20(Y181G) substrates at endogenous levels. Flp-In 293 T-REx cells, WT ZDHHC20 (W) or ZDHHC20(Y181G) (M), induced with doxycycline for 24 h, were treated with 15 µM 18-Bz (**e**) or YnPal (**f**) for 24 h. Lysates were clicked with biotin azide before enrichment on neutravidin magnetic beads. Representative immunoblots are shown for input and pull-down signals (*n* = 2 independent replicates).

We reasoned that ZDHHC overexpression would maximize sensitivity, and confocal immunofluorescence microscopy (Extended Data Fig. 5) and recapitulation of endogenous substrates and *S*-acylation sites (Fig. 4) suggest limited distortion of overexpression on substrate profiles. Nevertheless, overexpression has the potential to drive non-native ZDHHC localization and substrate interactions, and we sought to determine whether chemical–genetic substrate profiling could be achieved at tightly regulated and reduced expression levels. We established a panel of stable Flp-In T-REx HEK293 lines in which a ZDHHC20 construct is integrated at a single locus under control of a doxycycline-inducible promoter, enabling fine control of WT or Y181G ZDHHC20 expression to an identical level in the same background (Fig. 5a) and at ca. eightfold reduced expression relative to ectopic overexpression (Fig. 5b and Extended Data Fig. 6i).

Consistent with data from overexpression, 18-Bz was incorporated on induction of ZDHHC20(Y181G) expression at the whole-proteome level (Fig. 5a) and into HA-tagged Ifitm3 with excellent selectivity over WT ZDHHC20 (Fig. 5c). Selective quantitative enrichment of ZDHHC20(Y181G) substrates over WT was achieved on induction, covering a similar range of enrichment (up to 100-fold) as for ectopic expression (Fig. 5d and Supplementary Data 4). Consistent with reduced ZDHHC abundance, enrichment under inducible expression featured fewer hits overall than with ectopic overexpression (43 versus 214); >80% of hits were conserved between analyses with improved significance of enrichment in the inducible system (Supplementary Table 8), suggesting a promising approach for future studies. Selective chemical–genetic labeling of multiple endogenous substrates was also observed in the inducible system (Fig. 5e,f). Overall, these data show that chemical–genetic labeling remains selective and efficient at greatly reduced ZDHHC20 expression level, permitting selection of either inducible or ectopic expression depending on the system. However, labeling in a HEK293T cell line bearing a Y181G knock-in mutation at the endogenous locus by CRISPR/Cas9 gene editing did not enrich substrates at the level of endogenous ZDHHC20(Y181G) expression in HEK cells, suggesting a direction for future optimization (Supplementary Fig. 2).

### Chemical genetics enhances specificity and sensitivity

**Extended Data Fig. 7:**
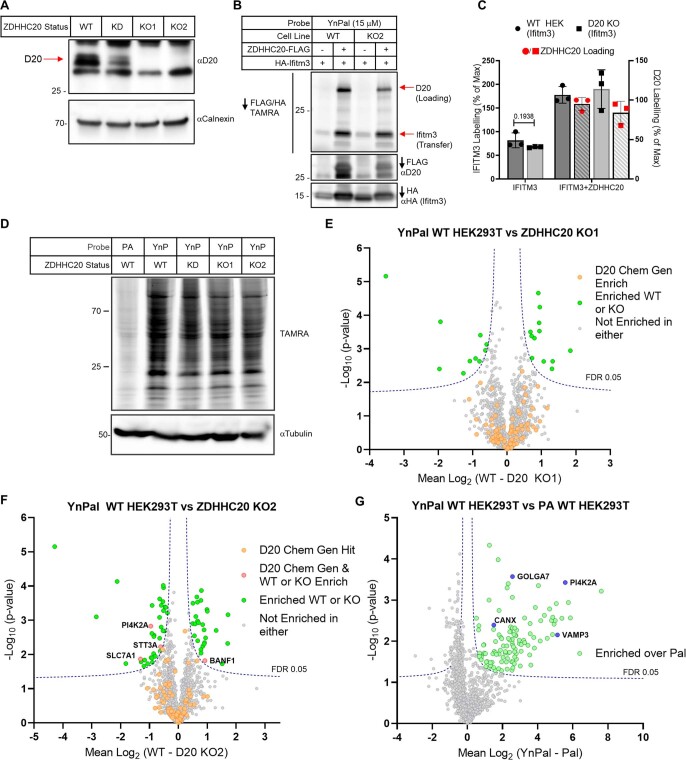
IFITM3 labeling in ZDHHC20 knock-out HEK293T cells. (**a**) Untreated (UT) or gRNA/CAS9 treated (pSpCas9(BB)-2A-Puro, PX459 plasmid) HEK293T cells were probed with anti-ZDHHC20 (D20) and –vinculin antibodies. Cells treated with gRNA1/CAS9 resulted in knockdown (KD); whereas cells treated with gRNA2/CAS9 yielded two ZDHHC20-knockout (D20-KO) clones: KO1 and KO2 (n = 2 independent biological replicates). (**b**) WT or KO2 HEK293T cells were transfected with HA-IFITM3 and empty vector or C-FLAG-tagged ZDHHC20. Cells were then treated with 15 mM YnPal for 4 h before being harvested and lysed. IFITM3 and D20 were enriched in one pot with a mix of anti-HA and –FLAG resins before being treated with TAMRA-azide and click reagents. Tagged proteins were eluted from beads with 1X Laemmli buffer and separated by SDS-PAGE. YnPal ZDHHC20-loading and transfer to IFITM3 and input were visualized by in-gel fluorescence and anti-HA and -FLAG immunoblot, respectively (n = 2 independent biological replicates). (**c**) The average (n = 3 independent biological replicates) loading and transfer activity was reported as a percent of the maximal D20 fluorescent: input ratio and as a percent of the WT IFITM3 (empty vector) fluorescent: input ratio ± S.D. The two tailed unpaired t-test of Prism 9.0 was used to determine p-values and noted above relevant comparisons (**d-g**) WT HEK293T cells, two ZDHHC20 KO clones, and one partial knockdown (KD) clone were treated with 15 μM YnPal for 8 h. As a control for lipidation, HEK293T cells were treated with palmitic acid (Pal) and also taken through the experiment. Samples were then clicked with biotin-TAMRA-azide, 10% of which was analyzed by SDS-PAGE, in-gel fluorescence, and anti-tubulin western blot (**d**) (n = 3 independent biological replicates). The remainder was enriched on dimethylated neutravidin beads and digested for LC-MS/MS LFQ analysis. (E-G) Whilst a small number of proteins are identified as being significantly enriched/depleted (Student’s two tailed unpaired T-test S_0_ – 0.1, adjusted FDR – 0.05), they are few in number and none are consistently found which correspond to our putative chemical genetic substrates found in HEK293T cells. (**f**) Analysis of YnPal treated cells against Pal shows a large number a potentially lipidated proteins have been identified, with many well validated *S*-acylation proteins identified, some of which have been highlighted in blue.

**Extended Data Fig. 8:**
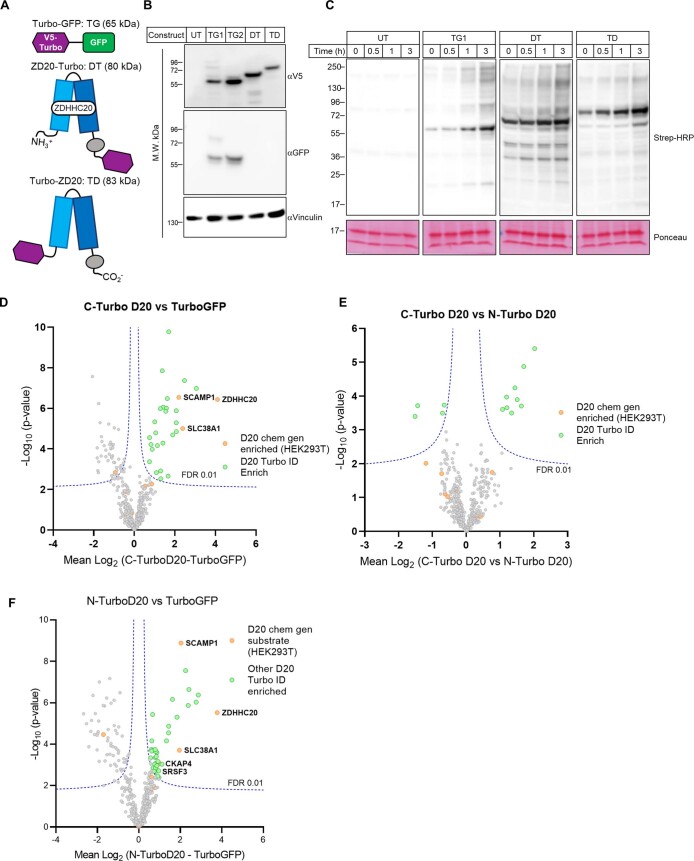
Cloning of TurboID chimeras and optimization of conditions for TurboID-enabled proximity labeling. (**a**) Schematic representation of TurboID fusion proteins used for proximity labeling experiments. (**b-c**) Confirmation of the expression of each fusion protein by western blot after generation of ‘Jump-in’ cell lines using either anti-V5 antibody (**b**) (n = 2 independent biological replicates) or an anti-GFP antibody (**c**) (n = 2 independent biological replicates). The labeling efficiency of the TurboID biotin ligase was confirmed by the addition of 500 μM biotin for the indicated times. Only those cells expressing the ligase show an increase in the biotinylation of proteins, as determined by Streptavidin conjugated HRP, compared to the UT HEK293T cells, and also in a time dependent manner. (**d**) Volcano plot showing the enrichment of proteins when comparing the C-terminally tagged ZDHHC20 with the N-terminally tagged construct (Student’s two tailed unpaired t-test S_0_ – 0.1, adjusted FDR 0.01). There does appear to be a preference for either the N- or C- terminus for some interactors, none of these correspond to our chemical genetic hits. (**e**) Volcano plot showing the enrichment of proteins when comparing the N-terminally tagged ZDHHC20 with the Turbo GFP construct (Student’s two tailed unpaired t-test S_0_ – 0.1, adjusted FDR 0.01). (**f**) TurboID-based proximity-labeing enabled detection of ZDHHC20 (D20) interactors. Volcano plot showing the mean log_2_ difference in protein group intensities between N-TurboID-ZDHHC20 and TurboID-GFP clones (Student’s two tailed unpaired T-test S_0_ – 0.1, adjusted FDR 0.01).

ZDHHC20 substrates identified through chemical genetics were next compared with conventional substrate identification approaches, including chemical proteomic analyses in ZDHHC20 KO cell lines and interactome analyses by proximity labeling using TurboID-fused ZDHHC20 (ref. ^45^). No changes were apparent between two clonal ZDHHC20 KO HEK293T cell lines generated by CRIPSR/Cas9 versus WT cells in either Ifitm3 *S*-acylation by western blot or chemical proteomic analyses of *S*-acylated proteins using YnPal, with approximately equal numbers of enriched or depleted proteins (Extended Data Fig. 7 and Supplementary Data 5). Quantitative interactome analyses of either N- or C-terminal ZDHHC20 TurboID fusions versus TurboID-GFP in HEK293T cells together identified only five known ZDHHC20 substrates, consistent with the common observation that transferase substrates are not typically strong interactors (Extended Data Fig. 8)^46,47^. These data suggest that ZDHHC chemical genetics offers a complementary approach to existing technologies by circumventing redundancy within ZDHHC substrate networks while enhancing chemical proteomic specificity and sensitivity toward bona fide substrates.

### Chemical–genetic systems across diverse ZDHHC family members

**Fig. 6:**
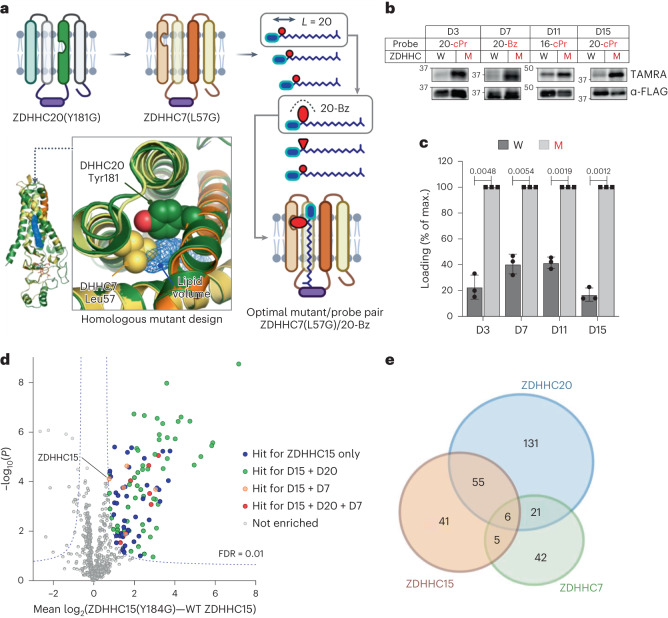
Extension of ZDDHC chemical genetics to ZDHHC3, 7, 11 and 15. **a**, Structure-guided ZDHHC engineering exemplified for ZDHHC7 (see also Extended Data Figs. 9 and 10). ZDHHC7 homology model (yellow/orange) overlayed on experimental ZDHHC20 structure (dark green) identifies a potential hole-generating amino acid (Leu57) on an adjacent helix in the vicinity of ZDHHC20 Tyr181; lipid density (blue mesh), and length/size probe analysis identifies a mutant/probe pair (ZDHHC7(L57G)/20-Bz) with optimal activity and selectivity over WT ZDHHC7. **b**, Bump-hole analysis of N-FLAG-tagged WT ZDHHCs or mutant ZDHHCs ZDHHC3(I182G) (D3), ZDHHC7(L57G) (D7), ZDHHC11(M181A) (D11) and ZDHHC15(Y184G) (D15) in HEK293T cell-based loading assays using 15 µM corresponding optimized probe. **c**, Average (*n* = 3 independent biological replicates) loading reported as a percent of maximal fluorescent:input ratio ± s.d. *P* values determined by Prism 9.0 two-tailed unpaired *t* test statistical module and noted above relevant comparisons. **d**, ZDHHC15 substrate discovery in HEK293T cells treated with 15 µM 20-*c*Pr in HEK293T cells using the OBH workflow. In total, 107 chemical–genetic ZDHHC15 substrates were identified (Student’s two-tailed unpaired *t* test, *S*_0_ = 0.5, adjusted FDR = 0.01, *n* = 4 independent biological replicates). Substrates unique or in common with parallel analyses for DHHC7 and DHHC20 in HEK293T cells are highlighted (Extended Data Fig. 10). **e**, Overlap of chemical–genetic ZDHHC substrates identified in HEK293T cells. Of 301 total substrates, only 87 are shared by 2 or more family members, suggesting distinct substrate pools for each ZDHHC.

We next generated models for the 4TM helices defining the lipid-binding pocket and catalytic domain of the remaining 22 human ZDHHCs using human ZDHHC20 as a template, to identify suitable residues for mutagenesis and activity studies (Supplementary Fig. 3)^3^. We prioritized bulky residues buried in rigid hydrophobic pockets aligning to ZDHHC20 Tyr181 over mutations on flexible loops or potentially destabilizing mutations at the helix-bilayer boundary because these are more likely to present suitable sites for steric complementation^48^. Our structure predictions for the 4TM lipid-binding core in the 6TM ZDHHCs 13 and 17 were well-correlated with AlphaFold predictions, with root-mean-square deviations of 1.5 and 1.7 Å, respectively (Supplementary Fig. 4)^49^. We generated Ala, Gly or double mutant constructs for each human ZDHHC and subjected them to the same two-stage screening strategy used for ZDHHC20 (Fig. 6a).

**Extended Data Fig. 9:**
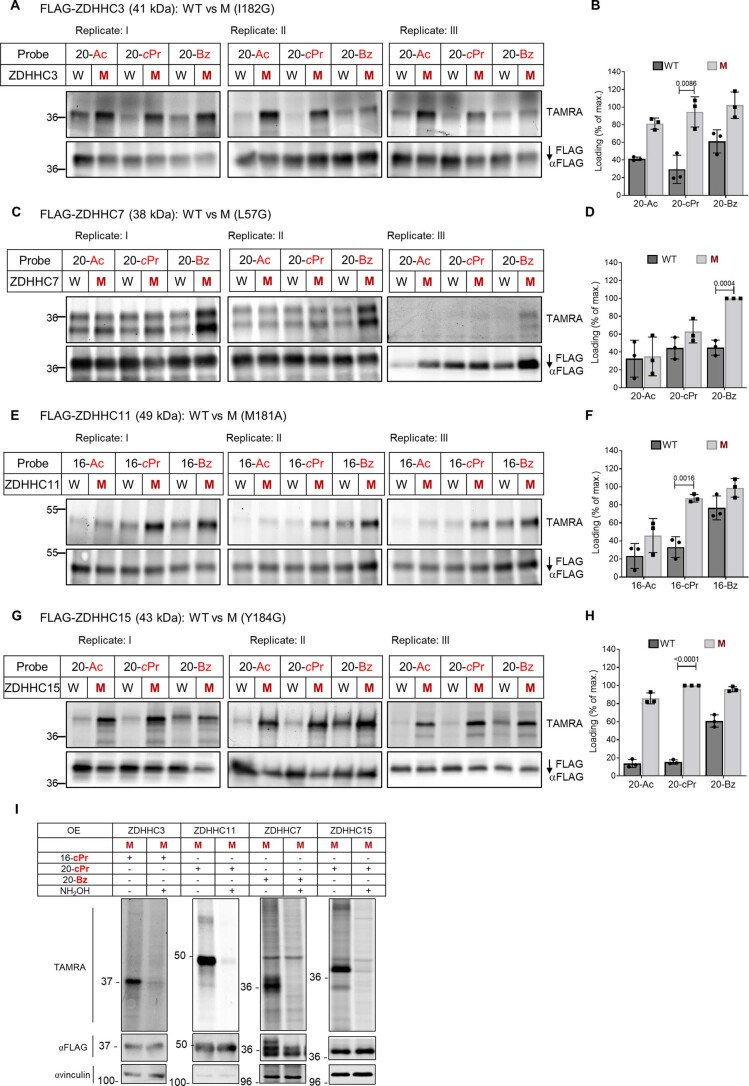
Bump optimization for mutants of ZDHHCs 3, 7, 11 and 15. (**a-h**) WT (W in tables) and the indicated mutants of N-FLAG-tagged ZDHHC family members, ZDHHC3 (**a-b**), ZDHHC7 (**c-d**), ZDHHC11 (**e-f**), and ZDHHC15 (**g-h**), were subjected to loading assays with probes containing optimal chain length and Ac, *c*Pr and Bz bump groups. WT and mutant constructs were transiently transfected into HEK293T cells and treated with 15 µM probe for 4 h. After cell lysis, constructs were immunoprecipitated on anti-FLAG resin, clicked with TAMRA-azide and separated by SDS-PAGE. Loading and input were visualized by in-gel fluorescence and anti-FLAG immunoblot, respectively. The average (n = 3 independent biological replicates) loading (**b**, **d**, **f**, and **h**) was reported as a percent of the maximal fluorescent: input ratios ± S.D. The two tailed unpaired t-test of Prism 9.0 was used to determine p-values and noted above relevant comparisons. (**i**) Thioester dependence of zDHHC7, zDHHC15, zDHHC3 and zDHHC11 labeling with bumped probes. HEK293T cells transiently expressing the acyltransferase mutants (M) were treated with C20-Bz (ZDHHC7), C20-cPr (ZDHHC15, ZDHHC11) or C16-cPr (ZDHHC3). Following CuAAC with TAMRA azide, lysates were treated with or without 0.8 M neutralized NH_2_OH. Representative images of 3 biological replicates (n = 3).

For several ZDHHCs, Gly mutation resulted in dramatically decreased expression (Supplementary Fig. 5), which in some cases could be restored by switching from Gly to a structurally more conservative Ala mutation or by adding strategically designed rescue mutations. For example, M181A in place of M181G improved ZDHHC11 expression, potentially by restoring backbone rigidity. ZDHHC15(Y184G) was also only modestly expressed; the reported crystal structure of zebrafish ZDHHC15 showed Ser35 hydrogen bonding to Tyr184, suggesting that Y184G may expose the Ser35 hydroxyl to the lipid-binding pocket. In support of this hypothesis, replacement of Ser35 with a less polar residue in a ZDHHC15(Y184G/S35C) double mutant improved expression. We identified several probes in the length screen displaying either selectivity or similar loading capacity for mutant versus WT ZDHHC; for example, 20-Ac was selectively loaded by ZDHHC3(I182G) and by ZDHHC15(Y184G/S35C), while 16-Ac was selectively loaded by ZDHHC11(M181A). Further refinement of probe selectivity for mutant over WT ZDHHC through bump screening at the optimal chain length led to the discovery of the following four additional optimized mutant/probe pairs: ZDHHC3(I182G)/20-*c*Pr, ZDHHC7(L57G)/20-Bz, ZDHHC11(M181A)/16-*c*Pr and ZDHHC15(Y184G)/20-*c*Pr (Fig. 6a–c and Extended Data Fig. 9), following optimization of expression to match WT ZDHHC. The thioester linkage introduced by these systems was further confirmed by labeling analysis with and without HA (Extended Data Fig. 9i). Taken together these data demonstrate that structure-guided design can expand chemical genetics across diverse ZDHHC family members and suggest that future generations of increasingly optimal ZDHHC mutant/probe pairs may be established by combining refined modeling approaches with wider-ranging mutant screens and additional bumped lipid designs.

### Comparative ZDHHC chemical–genetic substrate profiling

**Extended Data Fig. 10:**
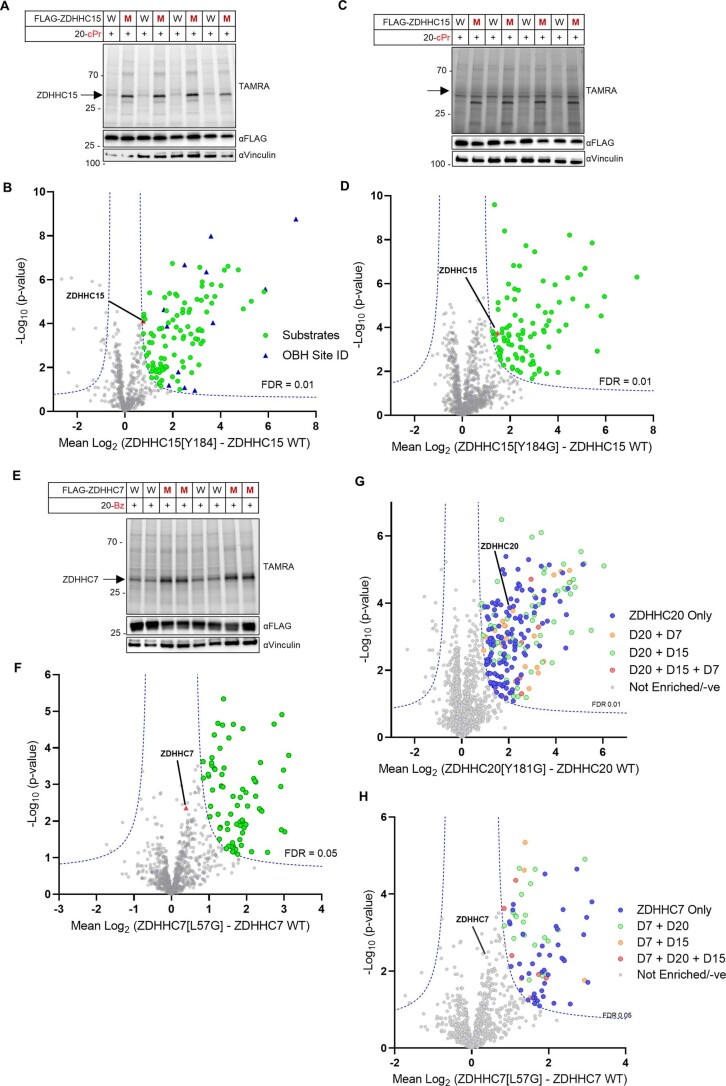
Proteomic analysis of ZDHHC15 and ZDHHC7 chemical genetic systems. (**a-b**) Gels and corresponding volcano plots for HEK293T cells treated with 15 μM 20-*c*Pr for 8 h in the presence of ZDHHC15 WT or ZDHHC15[Y184G]. (**a**) Lysates were subjected to CuAAC with TAMRA azide to assess probe incorporation and expression levels of FLAG-tagged ZDHHC and the loading control vinculin. (**b**) Volcano plot showing enrichment of putative ZDHHC15 substrates by ZDHHC15[Y184G] (Student’s two tailed unpaired T-test, S_0_ 0.5, adjusted FDR 0.01, n = 4 independent biological replicates) of matched lysates processed by OBH workflow and analyzed by LC-MS/MS. The positive control ZDHHC15 (red triangle) shows enrichment and many sites of modification (blue triangle) were identified through our OBH workflow. (**c-d**) Gels and corresponding volcano plots for PANC1 cells treated as described in (**a-b**). (**e-f**) Gels and corresponding volcano plots for HEK293T cells treated with 15 μM 20-*Bz* for 8 h in the presence of ZDHHC7 WT or ZDHHC7[L57G]. (**e**) Lysates were subjected to CuAAC with TAMRA azide to assess probe incorporation and expression levels of FLAG-tagged ZDHHCs and the loading control vinculin. (**f**) Volcano plot showing enrichment of putative ZDHHC7 substrates by ZDHHC7[L57G] (Student’s two tailed unpaired T-test, S_0_ 0.5, adjusted FDR 0.05, n = 4 independent biological replicates) of matched lysates processed by OBH workflow and analyzed by LC-MS/MS. (**g-h**) Overlap among ZDHHC substrate profiles for ZDHHC7, ZDHHC15, and ZDHHC20 (Student’s two tailed unpaired t-test, S_0_ 0.5, adjusted FDR 0.05, n = 4 independent biological replicates). (**g**) Volcano plot of ZDHHC20 OBH shown in Fig. 3b, with unique putative substrates; putative substrates shared with ZDHHC7 and/or ZDHHC15 highlighted. (**h**) Volcano plot of ZDHHC7 OBH shown in Supplementary Fig. 17 with unique putative substrates; putative substrates shared with ZDHHC15 and/or ZDHHC20 highlighted.

The identification of diverse ZDHHC mutant/probe pairs offers the opportunity to undertake comparative chemical–genetic substrate profiling between ZDHHCs. Chemical proteomic analyses of ZDHHC7(L57G)/20-Bz and ZDHHC15(Y184G)/20-cPr in HEK293T cells identified 74 and 107 substrates, respectively (Extended Data Fig. 10 and Supplementary Data 6 and 7), alongside 20 sites of modification across 13 proteins (Supplementary Data 2). Similarly, ZDHHC15 profiling in PANC1 cells rendered 91 substrates, 41 of which are in common with those identified in HEK293T cells (Extended Data Fig. 10 and Supplementary Data 7).

Among the 301 proteins identified across chemical proteomic substrate profiles, we observed common and distinct substrates between ZDHHCs 7, 15 and 20 consistent with a degree of redundancy across the DHHC family^29,50^ (Fig. 6d,f and Extended Data Fig. 9). However, common substrates were in the minority, with 55 (24%) shared substrates between closely related ZDHHC15 and ZDHHC20 (48% sequence identity), and only six (PTPN1, RHBDD2, SCAMP2, SLC7A1, TMEM161A, HMOX2) shared by all three *S*-acyltransferases. These data suggest that chemical genetics combined with chemical proteomics offers an approach to evaluate and compare substrate scope between ZDHHCs in intact cells.

## Discussion

Chemical genetics opens a ZDHHC-specific window on the expansive *S*-acylation network, enhancing the detection of substrates and *S*-acylation sites with low abundance or stoichiometry while simultaneously linking them to a cognate ZDHHC by circumventing ZDHHC redundancy. HEK293T cells express all 23 human ZDHHCs to a measurable extent^51^, and against this background, Ifitm3 *S*-acylation is essentially unaffected by ZDHHC20 KO, consistent with previous studies suggesting that IFITM3 may be *S*-acylated by ZDHHCs 3, 7, 15 and 20 (ref. ^21^). Indeed, a combination of traditional ZDHHC20 substrate identification strategies (KO, overexpression or proximity labeling with N- or C-terminal TurboID fusions) identified few significant hits and failed to identify known substrates at endogenous abundance (Supplementary Data 8). In contrast, chemical–genetic analyses readily identified endogenous IFITM3 as a high-confidence ZDHHC20 substrate, alongside several other examples (Figs. 3 and 5).

Extending chemical–genetic systems across the *S*-acyltransferase family has the potential to generate comprehensive contextual ZDHHC-specific substrate maps analogous to kinase-specific phosphorylation datasets, enabling elucidation of substrates and sites common and unique between ZDHHCs in varied cell and tissue types. The present study illustrates the potential of this approach through the identification of diverse partially overlapping substrate sets that exhibit a narrower spectrum of functional annotation than the wider *S*-acylated proteome. Validation of new and established ZDHHC20 substrates alongside high-fidelity recapitulation of known *S*-acylation site stoichiometry demonstrates that these datasets encompass bona fide ZDHHC-specific substrates, alongside a rich set of putative substrates for future validation. Chemical genetics also offers a unique approach for resolving ZDHHC isoform-dependent *S*-acylation at the level of specific PTM sites, while limiting or eliminating probe distribution into non-ZDHHC-dependent pathways (for example, membrane lipid biosynthesis, *O*- and *N*-linked acylation), which is an unavoidable liability of generic lipid analogs such as YnPal^41,52^. Lipidomic analyses suggest that bumped probes are not extensively processed into membrane lipid pools and do not alter endogenous lipid biosynthetic pathways.

We have demonstrated a systematic design and screening approach to establish effective ZDHHC chemical–genetic systems, including strategies for rescue mutations. In principle, we believe that most or all ZDHHCs should be amenable to chemical genetics through optimization beyond our preliminary screen through model refinement^49^, deeper mutational analysis, bumped lipid probe design and mutant/probe structure determination, for example, by cryogenic electron microscopy. Successful labeling of substrates by regulated low-level ZDHHC overexpression presents a promising direction for future optimization; however, initial experiments with gene-edited cell lines suggest that enrichment at endogenous ZDHHC expression may lie below the detection limit of first-generation designs. Next-generation designs may overcome this limitation while enabling new applications, for example, cell-type-specific analysis of ZDHHC activity in organoid or animal models^53^, or exploration of ZDHHC coregulation with multiplexed bioorthogonal tags^54^. Compatibility with cellular APT activity, which acts to reverse *S*-acylation, should also be investigated. Dysregulation of ZDHHC activity is associated with diverse pathologies including cancer, inflammation and neurodegeneration, and we envisage applications of chemical genetics for drug target validation and discovery in ZDHHC-associated disease models, and across diverse eukaryotes, for example, parasites, plants or fungi. Chemical proteomics also offers an ideal platform to analyze in-family selectivity of future small molecule ZDHHC inhibitors; conversely, it may prove possible to adapt bumped probes into chemical–genetic inhibitors, offering a general solution to the current lack of specific ZDHHC inhibitors for functional studies.

## Methods

### Synthesis of chemical probes

#### Materials

All chemicals and solvents were used as received from suppliers (Sigma-Aldrich (Merck), Thermo Fisher Scientific, Fluorochem or VWR) without further purification. Gases were from British Oxygen Company (BOC) Group and ultrapure water was used for all buffers.

#### Instrumentation

Hydrogen-1 nuclear magnetic resonance (^1^H-NMR) and carbon-13 nuclear magnetic resonance (^13^C-NMR) spectra were recorded on Bruker AV-400 (400 MHz) spectrometer, using residual isotopic solvent as an internal reference. Chemical shifts (*δ*) are given in units of parts per million (ppm). Each spectrum is corrected to the solvent reference signal. The multiplicity of each signal is given by singlet (s), doublet (d), triplet (t) or multiplet (m), and the number of protons (H) associated to a peak is indicated by nH. Coupling constants (*J*) are given in Hz and determined by analysis using MestReNova software.

Analytical LC–MS analysis was conducted on an Acquity UPLC BDH C18 column (50 mm × 2.1 mm, i.d. 1.7 µm packing diameter) at 40 °C. Flow rate was 0.5 ml min^−1^ and injection volume was 1 µl. The ultraviolet detection was a summed-up signal from wavelengths between 200 and 400 nm. UPLC retention times (*t*_*r*_) are reported in minutes. The following elution methods were used: method 1—(gradient of H_2_O and MeCN, supplemented with 0.1% formic acid) 3–100% MeCN for 0–1 min, 100% MeCN for 1–3.5 min, 100% to 3% MeCN for 3.5–3.6 min, 3% MeCN for 3.6–4 min; method 2—(gradient of 25 mM ammonium acetate (pH 8.0) and MeCN) 100% MeCN for 0–5 min, 100% MeCN for 5–5.5 min, 100% to 0% MeCN for 5.5–6.5 min, 0% MeCN for 6.5–9 min.

Chromatographic purifications were performed with a Biotage Isolera 4 using c-Hex/EtOAc gradient elution system. Final compounds were purified by PREP-LCMS (Agilent Technologies, 1260 series) equipped with a liquid chromatography/mass selective detector, an Agilent prep-C18 column (5 µm particle size, 21.2 × 50 mm) using water (containing 0.1% formic acid) and acetonitrile (containing 0.1% formic acid) in a gradient with a flow of 25 ml min^−1^.

#### Synthetic methods

Synthesis of compounds was performed according to Scheme 1 in Supplementary Information.

##### General method A (Jones oxidation)

The corresponding alcohol 1 (6.8 mmol) was dissolved in acetone (20 ml) and cooled to 0 °C, and 20 ml of chilled Jones reagent was added dropwise. The reaction was then allowed to warm up to room temperature and monitored by thin-layer chromatography (TLC) until completion. The reaction was quenched with 10% aqueous sodium thiosulfate, extracted with Et_2_O, dried and evaporated to give the target compound.

##### General method B (esterification)

The corresponding carboxylic acid (2a-c; 1 equiv.) was dissolved in MeOH (3 ml mmol^−1^) and heated to reflux. Concentrated H_2_SO_4_ (59 µl mmol^−1^) was added, and the reaction was monitored by TLC until completion. The reaction was then quenched with dH_2_O, extracted with Et_2_O, dried and evaporated to give compound 3 as a clear oil.

##### General method C (secondary amine formation)

The corresponding bromo methyl ester (3a-c; 1 equiv.) and propargylamine (10 equiv.) were dissolved in acetonitrile (30 ml g^−1^), and the reaction was set to reflux o/n and monitored by TLC. Upon completion, the solution was concentrated, cooled down and the resulting precipitate was collected by filtration, washed with cold acetonitrile and used in the next step without further purification.

##### General method D (amide bond formation)

The corresponding compound (4a-c; 1 equiv.) was dissolved in dry CH_2_Cl_2_ (3 ml mmol^−1^) under an inert argon atmosphere. *N*,*N*-Diisopropylethylamine (DIPEA) (3 equiv.) was added, and the reaction mixture was cooled to 0 °C. The corresponding acyl chloride was added (2 equiv.). The reaction was monitored until completion, upon which it was quenched with NaHCO_3_. The organic layer was extracted and dried, and the residue was purified by flash chromatography over silica gel using c-Hex/EtOAc (2:1→1:1) to yield the target compound.

##### General method E (ester hydrolysis)

The corresponding compound (5a-i) was dissolved in THF (1.5 ml mmol^−1^) and treated dropwise with 1 M LiOH (5 equiv.). The reaction was monitored until completion, quenched via addition of 1 M HCl to pH 1, extracted with EtOAc, dried and evaporated to give the product usually in quantitative yield.

##### Methyl 12-bromododecanoate (3a)

A solution was prepared by dissolving 12-bromododecanoic acid (1.0 g, 3.58 mmol) in 12 ml of H_2_O. Then, 200 µl of H_2_SO_4_ was added, and the resulting solution was refluxed for 4 h. The reaction mixture was diluted with 50 ml Et_2_O. The layers were separated, and the organic solution was washed with NaHCO_3_ (aq.), H_2_O and brine before it was dried on Na_2_SO_4_, filtered and concentrated under reduced pressure to yield compound 3a (1.05 g, 3.4 mmol, 95%). ^1^H-NMR (400 MHz, chloroform-d) *δ* 3.66 (s, 3H), 3.40 (t, *J* = 6.9 Hz, 2H), 2.29 (t, *J* = 7.5 Hz, 2H), 1.90–1.78 (m, 2H), 1.65–1.56 (m, 2H), 1.46–1.35 (m, 2H), 1.34–1.24 (m, 12H). ^13^C-NMR (101 MHz, chloroform-d) *δ* 174.28, 51.41, 34.07, 34.01, 32.80, 29.41, 29.35, 29.19, 29.10, 28.71, 28.14 and 24.91.

##### Methyl 12-(prop-2-yn-1-ylamino)dodecanoate (4a)

Compound 3a (500 mg, 1.7 mmol) was dissolved in MeCN (10 ml). Propargylamine (140 mg, 2.55 mmol) and K_2_CO_3_ (469 mg, 3.4 mmol) were added, and the solution was stirred o/n at 85 °C. The reaction mixture was concentrated under reduced pressure, and the dried crude was dissolved in 50 ml EtOAc, washed with NaHCO_3_ (2×) and brine before it was dried on Na_2_SO_4_, filtered and concentrated under reduced pressure. The crude was purified by flash chromatography over silica gel using c-Hex/EtOAc (1:1) + 1% 7 N NH_3_ in MeOH to yield 4a (190 mg, 0.71 mmol, 42%). ^1^H-NMR (400 MHz, chloroform-d) *δ* 3.65 (s, 3H), 3.41 (d, *J* = 2.4 Hz, 2H), and 2.71–2.62 (m, 2H), 2.28 (t, *J* = 7.5 Hz, 2H), 2.19 (t, *J* = 2.4 Hz, 1H), 1.66–1.56 (m, 2H), 1.50–1.41 (m, 2H), 1.34–1.20 (m, 14H). ^13^C-NMR (101 MHz, chloroform-d) *δ* 174.25, 82.35, 71.07, 51.36, 48.71, 38.16, 34.09, 29.81, 29.51, 29.47, 29.38, 29.20, 29.11, 27.27 and 24.93.

##### Methyl 12-(N-(prop-2-yn-1-yl)acetamido)dodecanoate (5a)

Compound 4a (30 mg, 0.11 mmol) was dissolved in dry CH_2_Cl_2_ (2 ml). DIPEA (30 µl, 0.22 mmol) was added, and the solution was cooled on ice. Acetyl chloride (17 µl, 0.22 mmol) in 1 ml CH_2_Cl_2_ was added dropwise. The reaction mixture was stirred on ice for 4 h. The reaction was quenched with 5 ml NaHCO_3_ (aq.), extracted with EtOAc (3×) and the combined organic layers were dried on Na_2_SO_4_, filtered and concentrated under reduced pressure. The residue was purified by flash chromatography over silica gel using c-Hex/EtOAc (2:1→1:1) to yield compound 5a (30 mg, 0.09 mmol, 86%). ^1^H- NMR (400 MHz, chloroform-d) *δ* 4.19 and 3.98 (d, *J* = 2.5 Hz, 2H), 3.65 (s, 3H), 3.38 (dt, *J* = 12.9, 7.5 Hz, 2H), 2.33–2.24 (m, 3H), 2.15 and 2.09 (s, 3H), 1.66–1.49 (m, 4H), 1.34–1.22 (m, 14H). ^13^C-NMR (101 MHz, chloroform-d) *δ* 174.25, 170.26, 170.00, 79.33, 72.29, 71.35, 51.39, 48.12, 46.27, 38.32, 34.09, 34.05, 29.47, 29.43, 29.36, 29.28, 29.20, 29.12, 28.42, 27.57, 26.88, 26.77, 24.94, 21.74 and 21.33.

##### 12-(N-(Prop-2-yn-1-yl)acetamido)dodecanoic acid (6a; 16-Ac)

Compound 5a (15 mg, 0.048 mmol) was dissolved in THF (5 ml) and lithium hydroxide monohydrate (LiOH·H_2_O; 42 mg, 0.92 mmol) in H_2_O (100 µl) was added dropwise to the reaction mixture. The reaction mixture was stirred at room temperature for 4 h. The reaction was quenched with 5 ml HCl (1 M), extracted with EtOAc (3×) and the combined organic layers were washed with brine, dried on Na_2_SO_4_ and concentrated under reduced pressure. The crude product was purified by flash chromatography over silica gel using c-Hex/EtOAc (1:2→1:5) to yield compound 6a (14 mg, 0.048 mmol, quantitative). ^1^H-NMR (400 MHz, chloroform-d) *δ* 4.21 and 4.00 (d, *J* = 2.5 Hz, 2H), 3.40 (dt, *J* = 15.1, 7.6 Hz, 2H), 2.34 (t, *J* = 7.5 Hz, 2H), 2.28 and 2.18 (m, 1H), 2.18 and 2.12 (s, 2H), 2.20–2.15 (m, 4H), 1.38–1.24 (m, 14H). ^13^C-NMR (101 MHz, CDCl3) *δ* 178.41, 170.27, 79.24, 78.54, 77.32, 77.00, 76.68, 72.40, 71.43, 48.13, 46.36, 38.34, 34.10, 33.85, 29.42, 29.36, 29.29, 29.25, 29.20, 29.14, 28.98, 28.86, 28.38, 27.49, 26.74, 24.67, 21.68 and 21.29. In LC–MS method 1, the retention time was 1.53 min and the observed *m*/*z* calc. for C_17_H_29_NO_3_ (M + H)^+^ was 296.29, which closely matches the calculated value of 296.21.

##### Methyl 12-(N-(prop-2-yn-1-yl)cyclopropanecarboxamido)dodecanoate (5b)

Compound 4a (59 mg, 0.22 mmol) was dissolved in dry CH_2_Cl_2_ (2 ml). DIPEA (77 µl, 0.44 mmol) was added, and the solution was cooled on ice. Cyclopropanecarbonyl chloride (40 µl, 0.44 mmol) in 1 ml CH_2_Cl_2_ was added dropwise. The reaction mixture was stirred on ice for 4 h. The reaction was quenched with 5 ml NaHCO_3_ (aq.), extracted with EtOAc (3×) and the combined organic layers were dried on Na_2_SO_4_, filtered and concentrated under reduced pressure. The residue was purified by flash chromatography over silica gel using c-Hex/EtOAc (2:1→1:1) to yield compound 5b (41 mg, 0.12 mmol, 55%). ^1^H-NMR (400 MHz, CDCl_3_) *δ* 4.19 (dd, *J* = 9.6, 2.6 Hz, 2H), 3.65 (s, 3H), 3.58–3.52 (m, 1H), 2.27 (d, *J* = 7.5 Hz, 2H), 1.72–1.50 (m, 5H), 1.26 (q, *J* = 8.0 Hz, 16H), 0.99 (qd, *J* = 5.3, 4.8, 1.8 Hz, 2H) and 0.77 (tt, *J* = 7.2, 3.3 Hz, 2H). ^13^C-NMR (101 MHz, CDCl3) *δ* 179.34, 174.34, 173.18, 79.58, 72.12, 71.28, 51.44, 47.42, 35.01, 34.09, 29.50, 29.45, 29.37, 29.32, 29.22, 29.12, 28.93, 26.85, 24.93, 20.47, 12.65, 11.20, 8.89, 7.97, 7.75 and 7.71.

##### 12-(N-(Prop-2-yn-1-yl)cyclopropanecarboxamido)dodecanoic acid (6b; 16-*c*Pr)

Compound 5b (41 mg, 0.12 mmol) was dissolved in THF (3 ml). In total, 610 µl of a 1 M LiOH solution in H_2_O was added dropwise. The reaction mixture was stirred at room temperature for 4 h. The reaction was quenched with 5 ml HCl (1 M), extracted with EtOAc (3×) and the combined organic layers were washed with brine, dried on Na_2_SO_4_ and concentrated under reduced pressure to yield compound 6b (13 mg, 0.040 mmol, 33%). ^1^H-NMR (400 MHz, CDCl3) *δ* 4.27–4.15 (m, 2H), 3.57 and 3.43 (t, *J* = 7.6 Hz, 1H), 2.34 (t, *J* = 7.5 Hz, 2H), 2.18 (d, *J* = 2.6 Hz, 1H), 1.83–1.51 (m, 5H), 1.40 – 1.18 (m, 14H), 1.02 (dt, *J* = 8.0, 4.2 Hz, 2H), 0.78 (dp, *J* = 7.2, 4.3 Hz, 2H). ^13^C-NMR (101 MHz, CDCl_3_) *δ* 178.96, 173.25, 79.58, 72.15, 71.30, 47.43, 37.61, 35.04, 33.93, 29.49, 29.41, 29.33, 29.29, 29.18, 29.01, 28.93, 27.62, 26.83, 24.68, 11.71, 11.24, 7.98 and 7.75. In LC–MS method 1, the retention time was 1.61 min and the observed *m*/*z* calc. for C_19_H_31_NO_3_ (M + H)^+^ was 322.33, which closely matches the calculated value of 322.23.

##### Methyl 12-(N-(prop-2-yn-1-yl)benzamido)dodecanoate (5c)

Compound 4a (59 mg, 0.22 mmol) was dissolved in dry CH_2_Cl_2_ (2 ml). DIPEA (77 µl, 0.44 mmol) was added and the solution was cooled on ice. Benzoyl chloride (51 µl, 0.44 mmol) in 1 ml CH_2_Cl_2_ was added dropwise. The reaction mixture was stirred on ice for 4 h. The reaction was quenched with 5 ml NaHCO_3_ (aq.), extracted with EtOAc (3×) and the combined organic layers were dried on Na_2_SO_4_, filtered and concentrated under reduced pressure. The residue was purified by flash chromatography over silica gel using c-Hex/EtOAc (2:1→1:1) to yield compound 5c (43 mg, 0.12 mmol, 52%). ^1^H- NMR (400 MHz, CDCl_3_) *δ* 7.49–7.31 (m, 4H), 4.37 (s, 1H), 3.99 (s, 1H), 3.70 (s, 3H) 3.64 (s, 1H), 3.38 (s, 1H), 2.30 (td, *J* = 7.7, 2.6 Hz, 3H), 1.61 (tt, *J* = 8.0, 4.3 Hz, 4H), 1.41–1.02 (m, 14H). ^13^C-NMR (101 MHz, CDCl_3_) *δ* 174.28, 136.04, 129.69, 128.43, 126.78, 78.99, 51.41, 34.09, 29.44, 29.36, 29.21, 29.16, 29.12 and 24.93.

##### 12-(N-(Prop-2-yn-1-yl)benzamido)dodecanoic acid (6c; 16-Bz)

Compound 5c (43 mg, 0.12 mmol) was dissolved in THF (3 ml). In total, 580 µl of a 1 M LiOH solution in H_2_O was added dropwise. The reaction mixture was stirred at room temperature for 4 h. The reaction was quenched with 5 ml HCl (1 M), extracted with EtOAc (3×) and the combined organic layers were washed with brine, dried on Na_2_SO_4_ and concentrated under reduced pressure to yield compound 6c (18 mg, 0.050 mmol, 44%). ^1^H-NMR (400 MHz, CDCl3) *δ* 7.49 (s, 1H), 7.41 (d, *J* = 3.9 Hz, 4H), 4.37 (s, 1H), 3.99 (s, 1H), 3.60 (s, 1H), 3.39 (s, 1H), 2.35 (t, *J* = 7.4 Hz, 2H), 2.29 (s, 1H), 1.64 (p, *J* = 7.3 Hz, 4H), 1.40 – 1.16 (m, 14H). In LC–MS method 1, the retention time was 1.66 min and the observed *m*/*z* calc. for C22H31NO3 (M + H)^+^ was 358.31, which closely matches the calculated value of 358.23.

##### Methyl 14-bromotetradecanoate (2b)

14-Bromododecan-1-ol (2.0 g, 6.8 mmol) was dissolved in acetone (50 ml). Jones reagent (10 ml) was added dropwise, and the reaction mixture was stirred for 2 h on ice. Half of the acetone was removed by rotary evaporation and H_2_O (50 ml) was added. The aqueous mixture was extracted with Et_2_O (3 × 75 ml). The combined organic layers were washed with H_2_O (2 × 25 ml), and brine before it was dried on Na_2_SO_4_ and concentrated under reduced pressure to yield compound 2a (2.1 g, 6.8 mmol, quantitative). ^1^H-NMR (400 MHz, chloroform-*d*) *δ* 3.40 (t, *J* = 6.9 Hz, 2H), 2.34 (t, *J* = 7.5 Hz, 2H), 1.85 (p, *J* = 7.0 Hz, 2H), 1.63 (q, *J* = 7.3 Hz, 2H), 1.43–1.26 (m, 20H). ^13^C-NMR (101 MHz, CDCl3) *δ* 180.15, 77.32, 77.00, 76.68, 34.40, 34.04, 33.95, 32.83, 29.52, 29.48, 29.40, 29.37, 29.19, 29.02, 28.74, 28.16, 25.00 and 24.64.

##### Methyl 14-bromotetradecanoate (3b)

Compound 2a (1.0 g, 3.4 mmol) was dissolved in dry MeOH (10 ml), H_2_SO_4_ (200 µl) was added and the mixture was stirred at 75 °C for 2 h. The mixture was then concentrated under reduced pressure until ~80% of the MeOH was evaporated. Water (50 ml) was added, and the aqueous mixture was extracted with Et_2_O (3×). The combined organic layers were washed with NaHCO_3_ (aq.), and brine before it was dried on Na_2_SO_4_, filtered and concentrated under reduced pressure to yield compound 3b (1.05 g, 3.4 mmol, quantitative). ^1^H-NMR (400 MHz, chloroform-*d*) *δ* 3.65 (s, 3H), 3.39 (t, *J* = 6.9 Hz, 2H), 2.28 (t, *J* = 7.5 Hz, 2H), 1.84 (dt, *J* = 14.4, 6.9 Hz, 2H), 1.61 (q, *J* = 7.4 Hz, 2H), 1.45–1.34 (m, 2H), 1.33–1.20 (m, 18H). ^13^C-NMR (101 MHz, CDCl_3_) *δ* 174.23, 77.32, 77.00, 76.68, 51.34, 34.06, 33.90, 32.81, 29.50, 29.46, 29.37, 29.19, 29.10, 28.71, 28.13, 25.71 and 24.91.

##### Methyl 14-(Prop-2-yn-1-ylamino)tetradecanoate (4b)

Compound 3b (0.5 g, 1.63 mmol) was dissolved in MeCN (15 ml), propargylamine (1.05 ml, 16.3 mmol) was added and the reaction mixture was stirred at 85 °C o/n. The solution was concentrated to ~4 ml, and the precipitate was collected by filtration. The filtrate was washed with cold MeCN to yield compound 4b (280 mg, 95 mmol, 58%). ^1^H-NMR (400 MHz, CDCl_3_) *δ* 3.86 (d, *J* = 2.6 Hz, 2H), 3.66 (s, 3H), 3.18–3.04 (m, 2H), 2.59 (t, *J* = 2.6 Hz, 1H), 2.30 (t, *J* = 7.6 Hz, 2H), 1.90 (p, *J* = 7.7 Hz, 2H), 1.61 (t, *J* = 7.3 Hz, 2H), 1.47–1.18 (m, 18H). ^13^C-NMR (101 MHz, CDCl_3_) *δ* 174.36, 78.44, 77.32, 77.00, 76.68, 72.43, 51.44, 46.26, 35.97, 34.11, 29.52, 29.46, 29.40, 29.35, 29.23, 29.13, 28.95, 26.75, 25.72 and 24.94.

##### 14-(N-(Prop-2-yn-1-yl)acetamido)tetradecanoic acid (6d; 18-Ac)

Compound 4b (60 mg, 0.2 mmol) was dissolved in dry CH_2_Cl_2_ (5 ml). Acetyl chloride (21 µl, 0.3 mmol) was added, and the solution was cooled on ice. DIPEA (41 µl, 0.3 mmol) in CH_2_Cl_2_ (1 ml) was added dropwise. The reaction mixture was stirred for 2 h before NaHCO_3_ (5 ml) was added. The layers were separated, and the aqueous layer was extracted with CH_2_Cl_2_. The combined organic layers were washed with brine, dried on MgSO_4_ and concentrated under reduced pressure. The residue was purified by flash chromatography over silica gel using c-Hex/EtOAc (2:1→1:3) to yield compound 5d (50 mg, 0.015 mmol, 75%). Compound 5d was used directly for ester hydrolysis without further purification.

Compound 5d (40 mg, 0. 11 mmol) was dissolved in THF (5 ml). In total, 1.2 ml of a 1 M LiOH solution in H_2_O was added dropwise. The reaction mixture was stirred at room temperature o/n. The reaction was quenched with 5 ml HCl (1 M), extracted with EtOAc (3×) and the combined organic layers were washed with brine, dried on Na_2_SO_4_ and concentrated under reduced pressure. The residue was purified by flash chromatography over silica gel using c-Hex/EtOAc (1:2→1:5) to yield compound 6d (25 mg, 0.077 mmol, 70%). ^1^H-NMR (400 MHz, CDCl_3_) *δ* 4.20 and 3.99 (d, *J* = 2.5 Hz, 2H), 3.46–3.34 (m, 2H), 2.33 (t, *J* = 7.5 Hz, 2H), 2.18 and 2.12 (m, 4H), 1.68–1.50 (m, 4H), 1.37–1.22 (m, 18H). ^13^C-NMR (101 MHz, CDCl_3_) *δ* 178.93, 178.84, 170.69, 170.35, 79.17, 78.61, 77.32, 77.00, 76.68, 72.41, 71.44, 48.14, 46.37, 38.34, 34.10, 33.98, 29.67, 29.50, 29.48, 29.45, 29.44, 29.39, 29.35, 29.29, 29.25, 29.19, 29.10, 29.02, 28.97, 28.34, 27.48, 26.81, 26.72, 24.69, 21.66 and 21.26. In LC–MS method 1, the retention time was 1.67 min and the observed *m*/*z* calc. for C19H33NO3 (M + H)^+^ was 324.27, which closely matches the calculated value of 324.26.

##### 14-(N-(Prop-2-yn-1-yl)cyclopropanecarboxamido)tetradecanoic acid (6e; 18-*c*Pr)

Compound 4b (40 mg, 0.13 mmol) was dissolved in dry CH_2_Cl_2_ (4 ml). Cyclopropanecarbonyl chloride (24 µl, 0.26 mmol) was added, and the solution was cooled on ice. DIPEA (45 µl, 0.26 mmol) in CH_2_Cl_2_ (1 ml) was added dropwise. The reaction mixture was stirred for 2 h before NaHCO_3_ (5 ml) was added. The layers were separated, and the aqueous layer was extracted with CH_2_Cl_2_. The combined organic layers were washed with brine, dried on MgSO_4_ and concentrated under reduced pressure. The residue was purified by flash chromatography over silica gel using petroleum ether/EtOAc (2:1→1:3) to yield compound 5e (41 mg, 0.112 mmol, 92%). Compound 5e was used directly for ester hydrolysis without further purification.

Compound 5e (41 mg, 0.112 mmol) was dissolved in THF (5 ml). In total, 1.2 ml of a 1 M LiOH solution in H_2_O was added dropwise. The reaction mixture was stirred at room temperature o/n. The reaction was quenched with 5 ml HCl (1 M), extracted with EtOAc (3×) and the combined organic layers were washed with brine, dried on Na_2_SO_4_ and concentrated under reduced pressure. The residue was purified by flash chromatography over silica gel using c-Hex/EtOAc (1:2→1:5) to yield compound 6e (31 mg, 0.088 mmol, 68%). ^1^H-NMR (400 MHz, CDCl_3_) *δ* 4.22 and 4.19 (d, 2.6 Hz, 2H), 3.56 and 3.42 (t, *J* = 7.6 Hz, 2H), 2.33 (t, *J* = 7.5 Hz, 2H), 2.29 and 2.17 (s, 1H), 1.83–1.49 (m, 5H), 1.37–1.20 (m, 18H), 1.04–0.97 (m, 2H), 0.82–0.74 (m, 2H). ^13^C-NMR (101 MHz, CDCl_3_) *δ* 179.26, 173.29, 79.56, 77.35, 77.04, 76.72, 72.16, 71.31, 47.45, 47.11, 37.62, 35.05, 34.01, 29.56, 29.53, 29.51, 29.39, 29.33, 29.23, 29.15, 29.05, 28.93, 27.66, 26.86, 24.71, 11.71, 11.24, 8.00 and 7.77. In LC–MS method 1, the retention time was 1.72 min and the observed *m*/*z* calc. for C_21_H_35_NO_3_ (M + H)^+^ was 350.36, which closely matches the calculated value of found 350.26.

##### 14-(N-(Prop-2-yn-1-yl)benzamido)tetradecanoic acid (6f; 18-Bz)

Compound 4b (85 mg, 0.29 mmol) was dissolved in dry CH_2_Cl_2_ (4 ml). Benzoyl chloride (31 µl, 0.29 mmol) was added, and the solution was cooled on ice. DIPEA (101 µl, 0.58 mmol) in CH_2_Cl_2_ (1 ml) was added dropwise. The reaction mixture was stirred for 2 h before NaHCO_3_ (5 ml) was added. The layers were separated, and the aqueous layer was extracted with CH_2_Cl_2_. The combined organic layers were washed with brine, dried on MgSO_4_ and concentrated under reduced pressure. The residue was purified by flash chromatography over silica gel using c-Hex/EtOAc (2:1→1:3) to yield compound 5f (68 mg, 0.17 mmol, 59%). Compound 5f was used directly for the ester hydrolysis without further purification.

Compound 5f (40 mg, 0.112 mmol) was dissolved in THF (5 ml). In total, 1.2 ml of a 1 M LiOH solution in H_2_O was added dropwise. The reaction mixture was stirred at room temperature o/n. The reaction was quenched with 5 ml HCl (1 M), extracted with EtOAc (3×) and the combined organic layers were washed with brine, dried on Na_2_SO_4_ and concentrated under reduced pressure. The residue was purified by flash chromatography over silica gel using c-Hex/EtOAc (1:2→1:5) to yield compound 6f (28 mg, 0.072 mmol, 67%). ^1^H-NMR (400 MHz, CDCl_3_) *δ* 7.48 (s, 1H), 7.41 (s, 4H), 4.37 (s, 1H), 3.98 (s, 1H), 3.60 (s, 1H), 3.37 (s, 1H), 2.33 (t, *J* = 7.5 Hz, 2H), 2.28 (s, 1H), 1.72–1.50 (m, 4H), 1.43–1.10 (m, 18H). ^13^C-NMR (101 MHz, CDCl_3_) *δ* 179.40, 135.91, 129.70, 128.46, 126.81, 78.92, 72.42, 71.95, 34.05, 29.53, 29.52, 29.39, 29.21, 29.05 and 24.71. In LC–MS method 1, the retention time was 1.66 min and the observed *m*/*z* calc. for C_24_H_35_NO_3_ (M + H)^+^ was 386.35, which closely matches the calculated value of 386.26.

##### Methyl 16-(prop-2-yn-1-ylamino)hexadecanoate (4c)

Methyl 16-bromohexadecanoate (0.5 g, 1.43 mmol) was dissolved in MeCN (15 ml), propargylamine (916 µl, 14.3 mmol) was added and the reaction mixture was stirred at 85 °C o/n. The solution was concentrated, cooled down and the resulting precipitate was collected by filtration, then washed with cold MeCN to yield compound 4c (391 mg, 1.21 mmol, 85%). ^1^H-NMR (400 MHz, CDCl3) *δ* 3.86 (d, *J* = 2.6 Hz, 2H), 3.67 (s, 3H), 3.17–3.09 (m, 2H), 2.59 (t, *J* = 2.6 Hz, 1H), 2.30 (t, *J* = 7.6 Hz, 2H), 1.89 (q, *J* = 7.9 Hz, 2H), 1.61 (d, *J* = 7.4 Hz, 2H), 1.41 (t, *J* = 7.7 Hz, 2H), 1.35–1.23 (m, 22H). ^13^C-NMR (101 MHz, CDCl_3_) *δ* 174.39, 78.38, 72.54, 51.46, 46.31, 35.99, 34.14, 29.63, 29.59, 29.51, 29.45, 29.39, 29.27, 29.17, 29.00, 26.79, 25.81 and 24.97.

##### 16-(N-(Prop-2-yn-1-yl)acetamido)hexadecanoic acid (6g; 20-Ac)

Compound 4c (50 mg, 0.15 mmol) was dissolved in dry CH_2_Cl_2_ (3 ml). Acetyl chloride (21 µl, 0.3 mmol) was added, and the solution was cooled on ice. DIPEA (54 µl, 0.3 mmol) in CH_2_Cl_2_ (1 ml) was added dropwise. The reaction mixture was stirred for 2 h before NaHCO_3_ (5 ml) was added. The layers were separated, and the aqueous layer was extracted with CH_2_Cl_2_. The combined organic layers were washed with brine, dried on MgSO_4_ and concentrated under reduced pressure. The residue was purified by flash chromatography over silica gel using c-Hex/EtOAc (2:1→1:3) to yield compound 5g (10 mg, 0.027 mmol, 18%).

Compound 5g (10 mg, 0.027 mmol) was dissolved in THF (1 ml). In total, 140 µl of a 1 M LiOH solution in H_2_O was added dropwise. The reaction mixture was stirred at room temperature for 4 h. The reaction was quenched with 1 ml HCl (1 M), extracted with EtOAc (3×) and the combined organic layers were washed with brine, dried on Na_2_SO_4_ and concentrated under reduced pressure to yield compound 6g (6 mg, 0.017 mmol, 60%). ^1^H-NMR (400 MHz, CDCl_3_) *δ* 4.21 (d, *J* = 2.5 Hz, 2H), 4.00 (d, *J* = 2.5 Hz, 1H), 3.41–3.34 (m, 2H), 2.18 (d, *J* = 1.9 Hz, 2H), 2.12 (s, 3H), 1.62 (q, *J* = 7.3 Hz, 4H), 1.26 (d, *J* = 2.9 Hz, 22H). ^13^C-NMR (101 MHz, CDCl_3_) *δ* 178.09, 169.78, 72.40, 71.43, 48.15, 38.36, 34.09, 29.57, 29.28, 29.22, 26.74, 24.72, 21.33. In LC–MS method 1, the retention time was 1.61 min and the observed *m*/*z* calc. for C_21_H_37_NO_3_ (M + H)^+^ was 352.36, which closely matches the calculated value of 352.28.

##### Methyl 16-(N-(prop-2-yn-1-yl)cyclopropanecarboxamido)hexadecanoate (5h)

Compound 4c (90 mg, 0.28 mmol) was dissolved in dry CH_2_Cl_2_ (5 ml). Cyclopropanecarbonyl chloride (50 µl, 0.56 mmol) was added, and the solution was cooled on ice. DIPEA (97 µl, 0.56 mmol) in CH_2_Cl_2_ (1 ml) was added dropwise. The reaction mixture was stirred for 2 h before NaHCO_3_ (5 ml) was added. The layers were separated, and the aqueous layer was extracted with CH_2_Cl_2_. The combined organic layers were washed with brine, dried on MgSO_4_ and concentrated under reduced pressure. The residue was purified by flash chromatography over silica gel using c-Hex/EtOAc (2:1→1:3) to yield compound 5h (53 mg, 0.14 mmol, 49%). ^1^H-NMR (400 MHz, CDCl_3_) *δ* 4.26–4.19 (m, 2H), 3.68 (s, 3H), 3.57 (d, *J* = 7.8 Hz, 1H), 2.32 (t, *J* = 7.5 Hz, 2H), 1.77–1.56 (m, 5H), 1.29 (d, *J* = 11.8 Hz, 23H), 1.03 (dq, *J* = 8.5, 4.5, 3.7 Hz, 2H), 0.80 (dt, *J* = 7.8, 3.4 Hz, 2H). ^13^C-NMR (101 MHz, CDCl3) *δ* 174.36, 173.13, 79.64, 71.24, 51.45, 47.41, 34.98, 34.13, 29.64, 29.59, 29.57, 29.45, 29.35, 29.26, 29.16, 28.95, 26.87, 24.97, 11.20, 7.96 and 7.70.

##### 16-(N-(Prop-2-yn-1-yl)cyclopropanecarboxamido)hexadecanoic acid (6h; 20-*c*Pr)

Compound 5h (53 mg, 0.14 mmol) was dissolved in THF (3 ml). In total, 700 µl of a 1 M LiOH solution in H_2_O was added dropwise. The reaction mixture was stirred at room temperature for 4 h. The reaction was quenched with 1 ml HCl (1 M), extracted with EtOAc (3×) and the combined organic layers were washed with brine, dried on Na_2_SO_4_ and concentrated under reduced pressure to yield compound 6h (32 mg, 0.085 mmol, 61%). ^1^H-NMR (400 MHz, CDCl3) *δ* 4.21 (dd, *J* = 11.6, 2.5 Hz, 2H), 3.60–3.51 (m, 1H), 3.43 (t, *J* = 7.6 Hz, 1H), 2.34 (t, *J* = 7.5 Hz, 2H), 2.18 (t, *J* = 2.5 Hz, 1H), 1.85–1.56 (m, 5H), 1.36–1.24 (m, 22H), 1.01 (td, *J* = 6.3, 5.4, 2.7 Hz, 2H), 0.78 (dt, *J* = 7.9, 3.4 Hz, 2H). ^13^C-NMR (101 MHz, CDCl_3_) *δ* 178.88, 173.24, 79.59, 72.13, 71.29, 47.44, 35.03, 33.92, 29.61, 29.54, 29.42, 29.34, 29.24, 29.06, 28.93, 26.86, 24.71, 11.24, 8.00 and 7.76. In LC–MS method 1, the retention time was 1.61 min and the observed *m*/*z* calc. for C_23_H_39_NO_3_ (M + H)^+^ was 378.40, which closely matches the calculated value of 378.29.

##### 16-(N-(Prop-2-yn-1-yl)benzamido)hexadecanoic acid (6i; 20-Bz)

Compound 4c (70 mg, 0.22 mmol) was dissolved in dry CH_2_Cl_2_ (2 ml). Benzoyl chloride (50 µl, 0.44 mmol) was added, and the solution was cooled on ice. DIPEA (76 µl, 0.44 mmol) in CH_2_Cl_2_ (1 ml) was added dropwise. The reaction mixture was stirred for 2 h before NaHCO_3_ (5 ml) was added. The layers were separated, and the aqueous layer was extracted with CH_2_Cl_2_. The combined organic layers were washed with brine, dried on MgSO_4_ and concentrated under reduced pressure. The residue was purified by flash chromatography over silica gel using c-Hex/EtOAc (2:1→1:3) to yield compound 5i (58 mg, 0.14 mmol, 63%). Compound 5i (40 mg, 0.094 mmol) was dissolved in THF (3 ml). In total, 470 µl of a 1 M LiOH solution in H_2_O was added dropwise. The reaction mixture was stirred at room temperature for 4 h. The reaction was quenched with 1 ml HCl (1 M), extracted with EtOAc (3×) and the combined organic layers were washed with brine, dried on Na_2_SO_4_ and concentrated under reduced pressure to yield compound 6i (12 mg, 0.029 mmol, 31%). ^1^H-NMR (400 MHz, CDCl_3_) *δ* 7.48 (s, 1H), 7.41 (d, *J* = 3.6 Hz, 4H), 4.37 (s, 1H), 3.99 (s, 1H), 3.61 (s, 1H), 3.37 (s, 1H), 2.33 (t, *J* = 7.5 Hz, 2H), 2.28 (s, 1H), 1.62 (p, *J* = 7.4 Hz, 4H), 1.30–1.14 (m, 20H). ^13^C-NMR (101 MHz, CDCl_3_) *δ* 179.62, 136.39, 130.20, 128.90, 127.25, 79.42, 72.26, 34.43, 30.05, 30.03, 29.99, 29.85, 29.67, 29.50 and 25.16. In LC–MS method 1, the retention time was 1.86 min and observed *m*/*z* calc. for C26H39NO3 (M + H)^+^ was 414.34, which closely matches the calculated value of 414.29.

##### C18-Bz-CoA probe

To a suspension of 14-(*N*-(prop-2-yn-1-yl)benzamido)tetradecanoic acid (30 mg, 78 μmol, 2 equiv.) in dry THF (1.2 ml) was added a solution of 1,1′-carbonyl-diimidazole (15 mg, 94 μmol, 2.4 equiv.) in CH_2_Cl_2_ (1.2 ml), under nitrogen atmosphere. The clear reaction mixture was stirred for 45 min at room temperature. The reaction mixture was concentrated under reduced pressure. The residue was dissolved in dry THF (1.2 ml). Coenzyme A hydrate (30 mg, 39 μmol, 1 equiv.) was dissolved in an aqueous solution of NaHCO_3_ (0.5 M, 4 ml) and added dropwise to the solution of activated acid. The reaction mixture was stirred at room temperature for 3 h under nitrogen atmosphere, flash frozen in liquid N_2_ and lyophilized overnight. The samples were then dissolved in 1 ml H_2_O, and the product was purified by preparative RP-HPLC over a gradient of 25 mM ammonium acetate pH 8 in MeCN. C18-Bz-CoA 19 was obtained as a white lyophilized solid (22 mg, 47% yield). ^1^H-NMR (400 MHz, D_2_O) *δ* 8.51 (s, 1H), 8.20 (s, 1H), 7.35 (dd, *J* = 13.8, 7.6 Hz, 4H), 7.27 (d, *J* = 7.4 Hz, 1H), 6.08 (d, *J* = 6.1 Hz, 1H), 4.80–4.70 (m, 2H), 4.53 (p, *J* = 2.8 Hz, 1H), 4.23–4.16 (m, 2H), 3.98 (s, 1H), 3.84 (dd, *J* = 9.8, 5.0 Hz, 1H), 3.52 (dd, *J* = 9.8, 4.7 Hz, 1H), 3.37 (p, *J* = 6.7 Hz, 2H), 3.22 (dtd, *J* = 11.1, 7.8, 7.3, 3.4 Hz, 4H), 2.89 (t, *J* = 6.7 Hz, 2H), 2.64 (d, *J* = 2.5 Hz, 1H), 2.42 (t, *J* = 7.4 Hz, 2H), 2.35 (t, *J* = 6.9 Hz, 2H), 1.44 (p, *J* = 7.3 Hz, 4H), 1.14–0.91 (m, 18H), 0.87 (s, 3H), 0.70 (s, 3H). ^13^C-NMR (151 MHz, D_2_O) *δ* 174.65, 173.41, 148.71, 140.74, 134.73, 128.69, 126.36, 118.34, 86.96, 82.43, 78.89, 74.06, 73.91, 65.16, 50.96, 43.67, 38.72, 38.44, 38.39, 35.47, 35.37, 29.32, 29.16, 28.98, 28.57, 27.99, 25.36, 21.05 and 18.13. In LC–MS method 2, the retention time was 4.39 min and observed *m*/*z* calc. for C_45_H_69_N_8_O_18_P_3_S (M + H) ^+^ was 1135.5, which closely matches the calculated value of 1135.37.

### Cell culture and compound preparation

HEK293T, HEK293-FT, MDA-MB-231 and PANC1 cell lines were cultured in Dulbecco’s modified Eagle medium (DMEM) supplemented with GlutaMAX (Thermo Fisher Scientific, 10566016), 10% vol/vol FBS, 100 U ml^−1^ penicillin and 0.1 mg ml^−1^ streptomycin (Thermo Fisher Scientific, 15140122) in a 37 °C, 5% CO_2_ incubator. Cells were selected with puromycin dihydrochloride (Thermo Fisher Scientific, A1113803), blasticidin S hydrochloride (Thermo Fisher Scientific, A1113903) and hygromycin B (Thermo Fisher Scientific, 10687010) at final concentrations noted below. Synthetic lipid-based loading and transfer probes and alkyne palmitic acid (2BScientific, BCAL-015-25), palmostatin B (Sigma-Aldrich, 178501), TAMRA or biotin PEG_3_ azide (Sigma-Aldrich, 760757 or 762024), tris((1-benzyl-1*H*-1,2,3-triazol-4-yl)methyl)amine (TBTA; Sigma-Aldrich, 678937) and palmitoyl coenzyme A (Sigma-Aldrich, P9716; ≥90%) were dissolved in DMSO and stored at −20 °C. Sealed ampules containing a 0.5 M aqueous solution of tris (2-carboxyethyl) phosphine (TCEP) and TCEP HCl (C4706) were purchased from Sigma-Aldrich, and n-dodecyl-β-d-maltopyranoside (DDM) was purchased from Generon (D310LA). TCEP HCl was prepared fresh as 50 mM stock in Milli-Q H_2_O, DDM was prepared as a 10% stock solution in Milli-Q H_2_O and stored at −20 °C and cOmplete, EDTA-free protease inhibitor cocktail tablets were used according to the manufacturer’s instructions (Sigma-Aldrich, 11873580001).

#### Antibodies and western analysis

Mouse-derived monoclonal antibodies for FLAG M2 (F1804), HA-epitope (HA-7, H3663) and α-tubulin (T5168) and rabbit-derived polyclonal antibodies for ZDHHC20 (Atlas Antibodies, HPA014702), BCAP31 (Atlas Antibodies, HPA003906) and V5-epitope (SAB1306079) were purchased from Sigma-Aldrich. Mouse monoclonal anti-GFP (GF28R) antibody was purchased from Generon, rabbit monoclonal anti-TOMM20 (ab186735) antibody was purchased from Abcam, rabbit polyclonal anti-TMX1 (HPA003085) antibody was purchased from Atlas Antibodies, rabbit polyclonal anti-NCAM1/CD56 (14255-1-AP) and anti-PI4K2A (15318-1-AP) antibodies were bought from ProteinTech and rabbit-derived polyclonal antibodies against vinculin (42H89L44) and calnexin (ab22595) were purchased from Thermo Fisher Scientific and Abcam, respectively. Secondary antibodies were horseradish peroxidase (HRP)-conjugated, polyclonal goat-derived antibodies against mouse (Dako, P0447) and rabbit (Dako, P0448), or fluorophore-conjugated IRDye 680CW goat anti-mouse (Licor, 926-68072) and IRDye 680CW goat anti-rabbit (Licor, 926-32211). Western blot analysis was accomplished through SDS–PAGE of cell lysates or affinity-resin eluates in 1× Laemmli loading buffer (Bio-Rad, 1610747) containing 2.5% β-mercaptoethanol and transfer of protein onto polyvinylidene fluoride (PVDF) or nitrocellulose using the Trans-Blot Turbo System (Bio-Rad). Secondary HRP-conjugates were visualized after addition of Clarity Western ECL Substrate (Bio-Rad, 1705061) and chemiluminescent detection in an Amersham Imager 680 (GE Life Sciences) or fluorescence detection using a LICOR Odyssey CLx. Quantitation of western blot protein intensity was performed by densitometry using ImageJ 1.50c or Image Studio Lite (GE Life Sciences), and data were plotted using Prism 9.0.

#### ZDHHC structural modeling

Human ZDHHC family protein sequences were aligned using the ‘Create Alignment’ module of CLC Sequence Viewer 7. Regions of sequence similarity that also overlap with ZDHHC20 transmembrane helices (TMs) 1–4 and the DHHC-containing cysteine-rich domain were identified and selected for homology modeling. To generate homology models for ZDHHCs 1–19 and 21–24, selected sequences were individually submitted to the Protein Homology/analogY Recognition Engine V 2.0 (Phyre^2^) using the ‘Normal’ modeling mode^55^. To identify putative bump-hole mutations, homology models were structurally aligned to the ZDHHC20-2BP crystal structure (Protein Data Bank (PDB) ID: 6BML) using MacPyMOL: PyMOL V1.5.0.4. Residues located on TM3 and spatially overlapping with or proximal to ZDHHC20-Y181 were prioritized for bump-hole analysis; however, several ZDHHC models did not present residues meeting these criteria. In this case, strict rules were adopted including (1) selection of ZDHHC20-Y181 or (2) -F65 proximal residues located on TMs 2 and 3 overlapping with ZDHHC20 residues having B-factor values <100 and (3) when rules 1 and 2 fail, residues on TM1 proximal to the ω-position of the 2BP fatty acid chain and with lowest ZDHHC20 B-factor value were selected for bump-hole screening. Residues presented by TM1 were given least priority as their side chains have access to the lipid-binding pocket and the bilayer. Potentially, mutations on this helix could generate a hole in the pocket, leading to structural instability or loss of lipid-probe binding affinity.

### Molecular biology and cloning

#### Plasmids and subcloning

For a complete list of vectors used in this study, refer to Supplementary Table 2. The preparation of new plasmids generated for this study will be described herein. Human C-terminally Myc-FLAG-tagged ZDHHC20 (C-FLAG-D20) was purchased from Origene Technologies (MR205665). Plasmids for expression of N-terminally 3xFLAG-tagged human ZDHHCs 1–24 (N-FLAG-DX, *X* = 1–24), HA-tagged GCP16 (GOLGA7, ZDHHC9 cofactor) and empty pEF-1α vector were a generous gift from Y. Ohno (Hokkaido University). The C-FLAG-pcDNA3 expression vector (Addgene, 20011) was also used as a negative control for FLAG-tagged ZDHHC expression. C-terminally Myc-HA-tagged ZDHHC20 (C-HA-D20) PCR fragment was subcloned into the PmeI and AsiSI linearized C-FLAG-D20 vector using the NEBuilder HiFi Assembly Kit (NEB, E5520S). C-HA-D20 fragment, with HA-tag sequence spacer (bold), was generated by PCR using Phusion DNA Polymerase and the primer set in Supplementary Table 3. C-FLAG-D20 was subcloned into the pLVX-TetOne-Puro (Clontech) and in-house attb vectors, expressing mouse ZDHHC20 and containing a blasticidin resistance marker (attb-ZDHHC20-BSDr), using the same strategy. Empty pLVX-TetOne-Puro and attb-ZDHHC20-BSDr were linearized using EcoRI and BamHI and XhoI and MfeI, respectively, and human C-FLAG-D20 fragments generated with primer sets indicated in Supplementary Table 3. V5-tagged TurboID (promiscuous BirA mutant, V5-Turbo-NES-pCDNA3; Addgene, 107169) and C-FLAG-D20 or EGFP (pEGFP-N1-FLAG; Addgene, 60360) chimeras were also subcloned into the XhoI- and MfeI-linearized attb-BSDr vector using the NEBuilder HiFi Assembly Kit, and C-FLAG-D20 WT and C-FLAG-D20(Y181G) were subcloned into the BamHI- and NotI-linearized pcDNA5/FRT (see Supplementary Table 4 for primer sets). Plasmids for the expression of PI4K2A (pDONR223-PI4K2A; Addgene, 23503), TOMM20 (mCherry-TOMM20-N-10; Addgene, 55146) and TFAM (pcDNA3-TFAM-mCLOVER; Addgene, 129574) were subcloned into the EcoRI- and XhoI-linearized N-HA-Ifitm3 vector using the NEBuilder HiFi Assembly Kit (see Supplementary Table 3 for primer sets used to create N-HA-tagged plasmids). pcDNA3.1 XXYLT1-HA was kindly provided by H. Bakker. All new plasmids were sent to GATC Biotech for Sanger sequencing to confirm sequences of entire inserts and junctions between backbone and insert and backbone and PCR fragment. Primers used to produce PCR fragments were purchased from Sigma-Aldrich.

#### Molecular cloning

Lentiviral plasmid pLVX-C-FLAG-D20, packaging and viral envelope plasmids pCMV-Delta-8.2 (Addgene, 12263) and pCMV-VSV-G (Addgene, 8454) and HEK293-FT cells were used to prepare lentiviral particles according to the instructions in the Lenti-X Lentiviral Expression System Manual (Clontech). Doxycycline-inducible ZDHHC20 expressing HEK293T cells were prepared by transduction of 2.5 × 10^5^ low-passage cells with lentivirus, followed by puromycin selection at 1.0 μg ml^−1^ for 1 week. After 1 week, cells were cultured in normal media containing 0.5 μg ml^−1^ puromycin and, after four or more passages, sent to the Flow Cytometry Science Technical Platform for single-cell sorting into 96-well plates. Briefly, single cells were sorted on the Beckman Coulter MoFlo XDP, using the 488 nm forward scatter (FSC) LASER signal to trigger events. Cells were identified and separated from debris using side scatter (SSC) height versus FSC height. Doublets were then removed using SSC height versus SSC width (Supplementary Fig. 6). Single cells were sorted into 96-well plates using the single-cell sort mask with a drop envelope of 0.5. Clones were expanded into 12-well plates and induced with 2 μg ml^−1^ doxycycline for 2 d before anti-FLAG western blot screening. Jump-in TurboID cell lines were prepared by cotransfecting HEK293T cells with 1.0 μg and 1.5 μg of attb-BSDr containing the gene of interest and pCMV-Int (ΦC31-integrase; Addgene, 18935) plasmids, respectively, followed by selection with 10 μg ml^−1^ blasticidin for 1 week. For transfection conditions, see Cellular and biochemical analysis—Cellular ZDHHC-autoacylation and substrate transfer. After selection, single cells were sorted into 96-well plates, as described above, expanded into 12-well plates and then screened by immunoblot with antibodies against GFP, V5 and FLAG epitopes.

#### Subcloning BCAP31 into mammalian expression vector

After extracting total RNA from HEK293T cells using the GenElute Total RNA Purification Kit (Merck, RNB100), 500 ng of total RNA was used to amplify BCAP31 (isoform 1, P51572) cDNA using the SuperScript III One-Step RT-PCR System with Platinum Taq DNA Polymerase (Thermo Fisher Scientific, 12574018) and the following primer set: forward primer ATG AGT CTG CAG TGG ACT GCA GTT G and reverse primer TTA CTC TTC CTT CTT GTC CAT G. BCAP31 cDNA was purified by agarose gel electrophoresis, extracted and shuttled into a TOPO-TA vector. After blue–white screening, white colonies were selected, amplified in the presence of ampicillin and then collected to prepare DNA minipreps. Miniprepped DNA was digested with KpnI and SmaI and analyzed by agarose gel electrophoresis to confirm the presence of BCAP31. All inserts were oriented in the reverse sense, and Sanger sequencing verified the sequence of human BCAP31, isoform 1. To generate an HA-tagged BCAP31 construct in a mammalian expression vector, BCAP31 PCR fragment was subcloned into SalI- and BalI-linearized pcDNA3.1(+)-HA-Ifitm3 vector using the NEBuilder HiFi Assembly kit. PCR primers used to generate the HA-BCAP31 PCR fragment are listed in Supplementary Table 3.

#### Mutagenesis

All mutagenesis reactions were carried out using the QuikChange II Site-Directed or Lightning MultiSite-Directed Mutagenesis Kits (Agilent, 200523) according to the manufacturer’s instructions. See Supplementary Tables 5 and 6 for a list of mutations and mutagenic primer sets. All plasmids were sent to GATC Biotech or Genomics Equipment Park (The Francis Crick Institute) for Sanger sequencing to confirm mutations. Mutagenic primers were purchased from Sigma-Aldrich.

### Cellular and biochemical analysis

#### Click reactions

Click reactions were performed with 100 μM of the described azide, 1 mM CuSO_4_, 1 mM TCEP and 100 μM TBTA for 1 h at room temperature and were quenched with 5 mM EDTA or the reaction solution removed when performed on-bead.

#### Cellular ZDHHC-autoacylation and substrate transfer

##### Pulldown

FLAG-tagged ZDHHC was singly or cotransfected with affinity-tagged substrate (FLAG or HA epitopes) in HEK293T cells. For a single, reverse-transfection mixture, 1–2 μg of ZDHHC plasmid, alone (autoacylation) or in combination with 0.5 μg substrate (transfer) plasmid, was mixed with three volumes of FuGene HD (3:1 FuGene (μl)/plasmid (μg); Promega, E2311) in 100 μl of Opti-MEM. After 15 min, the transfection mixtures were added to 1–2 × 10^6^ cells in 900 μl of culture media and cells incubated at 37 °C o/n. Cells were treated with DMSO or probe in 10% FBS (YnPal) or 0.5% FBS (bumped probes) at the indicated concentrations for 4 h at 37 °C, after which they were dislodged by manual pipetting, pelleted at 200*g* for 5 min and the media was discarded. Cells were washed 2× ice-cold phosphate buffered saline (PBS) and pelleted each time to remove the supernatant. Cells were lysed with 0.5 ml DDM lysis buffer—50 mM Tris–HCl (pH 7.5), 150 mM NaCl, 10% glycerol, 1% DDM, 0.5 mM TCEP, 10 μM palmostatin B, 1× protease inhibitor tablet, 2 mM MgCl_2_ and 0.05 U μl^−1^ benzonase. After shaking for 10 min at room temperature, lysates were clarified at 17,000*g* for 10 min at 4 °C. The clarified lysates were treated with the appropriate affinity resins, anti-FLAG M2 (Sigma-Aldrich, M8823) and/or Pierce anti-HA (Life Technologies, 88836) magnetic beads, or diluted with 4× Laemmli buffer for analysis. FLAG- and/or HA-tagged proteins were immunoprecipitated for 2 h at room temperature or o/n at 4 °C and then washed with wash buffer (1% NP-40 in PBS). A click reaction was performed with TAMRA-azide in wash buffer for 1 h, then beads were washed with wash buffer, treated with 1× Laemmli loading buffer and then eluates subjected to SDS–PAGE. For samples used to demonstrate thioester-dependent labeling, before 1× Laemmli addition, beads were incubated with 0.8 M neutralized NH_2_OH in PBS (pH 7.4) for 1 h at room temperature and then diluted with 4× Laemmli buffer. After protein separation, TAMRA-labeled proteins were visualized using a Typhoon FLA 9500 instrument. The Typhoon resolution was set to 25 μm and the photomultiplier tube (PMT) value varied from 500 to 1,000 depending on signal intensity. Finally, protein was transferred to PVDF/nitrocellulose and analyzed via western analysis to reveal ZDHHC and substrate input. For loading and transfer, the fluorescent signals were normalized against input as determined by western blot analysis.

#### Cell lysate

These follow the same protocol as the pull-down assay with the following modifications. Cells were lysed in a PBS RIPA buffer supplemented with 1× protease inhibitor and clarified at 17,000*g* and 4 °C. Lysates were clicked with TAMRA-azide for 1 h and then precipitated using chloroform/methanol. Protein pellets were washed in MeOH and then dissolved in 1× Laemmli buffer diluted in 1% NP-40 in PBS by shaking for 1 h at room temperature. For samples used to demonstrate thioester-dependent labeling, the dissolution solution was supplemented with 0.8 M neutralized NH_2_OH and lysates were then analyzed by in-gel fluorescence and western blot.

#### TREX HEK293 assays

TREX HEK293T cells were seeded and left overnight in an incubator at 37 °C in standard media supplemented with 1 μg ml^−1^ blasticidin and 50 μg ml^−1^ hygromycin B. On the day before the treatment of cells with the appropriate probes, media was supplemented with 1 μg ml^−1^ of doxycycline or water alongside any transfection reagents that were applied as described above. All assays then followed the same methods as described above.

#### ZDHHC20 enzyme kinetic mutant and probe analysis

##### Protein purification

For each FLAG-ZDHHC20 construct, two dishes of HEK293T cells were prepared and transfected with calcium phosphate transfection mix. To a 10 cm tissue culture dish, 6 × 10^6^ HEK293T cells in 8 ml DMEM culture media were added. Cells were allowed to settle o/n in a 37 °C incubator. To prepare the calcium phosphate transfection mix, 10 μg of C-FLAG-D20 construct and water were added to a 1.5 ml Eppendorf tube to a volume of 436 μl. To this was added 64 μl of 2 M CaCl_2_ to give a final volume of 500 μl. This solution was slowly added dropwise and with continuous bubbling to 500 μl 2× Hanks’ balanced salt solution (HBSS;273.8 mM NaCl, 9.4 mM KCl, 1.5 mM Na_2_HPO_4_-7H_2_O, 15 mM glucose, 42 mM HEPES (free acid) pH 7.05 in Milli-Q H_2_O, filter sterilized) in a sterile 30 ml polystyrene tube. This solution was incubated at room temperature for 5 min before use. To a 10 cm plate of HEK293T cells, 1 ml of the DNA mixture was added. The mix was added dropwise and evenly across the media surface to ensure maximal cell coverage with precipitated DNA. The tissue culture dish was returned to the 37 °C incubator and left to incubate for 72 h before collecting cells for protein purification.

After 72 h, cells were dislodged manually using an automatic pipette, collected in a 50 ml falcon tube and pelleted at 200*g* for 5 min. The media was decanted, and cells were washed 3× with cold PBS. The cell pellet was dislodged and lysed with 5 ml 2% DDM buffer (50 mM Tris–HCl (pH 7.5), 150 mM NaCl, 5% glycerol, 2% DDM, 0.5 mM TCEP, 10 μM palmostatin B and 1× protease inhibitor tablet), vortexed thoroughly and allowed to incubate at 4 °C with constant rotation. After 4 h, lysate was centrifuged for 20 min at 20,000*g* and 4 °C. In total, 1 ml anti-FLAG M2 Affinity Gel suspension was equilibrated in 5 ml 1% DDM buffer before use. The clarified lysate was diluted with 1 volume (~5 ml) of the described buffer containing no DDM, to give a final concentration of 1% DDM, before being added to cold resin and allowed to mix o/n at 4 °C. After incubation, the lysate–resin mix was added to an Econo-Pac gravity-flow chromatography column (Bio-Rad, 7321010). The lysate container was rinsed thoroughly with 1% DDM buffer to transfer all residual resin to the column. The resin was washed sequentially with ice-cold buffer W1 (50 mM Tris–HCl (pH 7.5), 150 mM NaCl, 0.2% DDM, 2 mM TCEP and 1× protease inhibitors), W2 (25 mM HEPES (pH 7.5), 500 mM NaCl, 0.2% DDM, 2 mM TCEP and 1× protease inhibitors) and W3 (W2 with 25 mM NaCl). After washing, FLAG-ZDHHC20 was eluted with 5 × 0.5 ml portions of buffer W3 containing 0.25 mg ml^−1^ 3× FLAG-peptide (MDYKDHDGDYKDHDIDYKDDDDK, Crick Peptide Chemistry STP stock peptide). FLAG-ZDHHC20 containing fractions were pooled, and buffer was exchanged with W3 simultaneously with concentration using an Amicon Ultra 15 ml Spin-Column with 50 kDa cut-off.

#### Enzyme-coupled ZDHHC20-autoacylation assay

Autoacylation reactions were carried out as previously described with a few exceptions^3^. Reactions were prepared in a Corning 96-well black half-area plate (3686). In one well was prepared a prestart mix with the indicated concentration of fatty acid- or probe-CoA, 2 mM α-ketoglutaric acid, 0.25 mM NAD (β-nicotinamide adenine dinucleotide, oxidized) and 0.2 mM thiamine pyrophosphate in 25 μl reaction buffer (25 mM MES (pH 6.8), 50 mM NaCl, 1 mM DTT, 1 mM EDTA, 0.2 mM DDM and 1× protease inhibitors). The reaction was started by addition of 25 μl ZDHHC master mix containing 20 nM ZDHHC20 and 2 μl of α-ketoglutarate dehydrogenase (KDH; Sigma-Aldrich, K1502). In the first reaction step, ZDHHC20 autoacylation leads to the production of free CoA-SH, which is then converted to NADH in the next step by KDH. NADH production was monitored using the fluorescence module (Ex. 360 nm/Em. 465 nm) of the Tecan Spark Multimode Microplate Reader. Note that 18-Bz-CoA exhibited weak, but measurable, activity in KDH reactions without ZDHHC20 enzyme. Therefore, all rates from reactions with ZDHHC20 and 18-Bz-CoA were adjusted by subtraction of the ZDHHC20-independent rates from the corresponding total rates of reactions with ZDHHC20 (Extended Data Fig. 3g).

### Confocal microscopy and colocalization analysis

For transient expression, 4 × 105 cells were plated per well in a six-well in 1.5 polylysine-l (Sigma, P4707) coated 1.5 borosilicate glass coverslips (Zeiss). The next day, cells were cotransfected with 0.5 μg of HA-tagged ZDHHC20 WT and 0.5 μg of FLAG-tagged ZDHHC20 Y181G bump-hole mutant plasmid DNA in 200 μl of Opti-MEM (Thermo Fisher Scientific, 31985062) and mixed with FuGene HD with a 3:1 ratio (DNA). Cells were left overnight in the incubator. Twenty-four hours after the transfection, media containing the transfection mix was removed and washed twice with PBS. Samples were then incubated with 2 ml of ice-cold methanol for 15 min over ice, then removed and cells washed thrice with PBS. Coverslips were blocked with 2 ml of 5% Donkey serum (Sigma, D9663) in PBS for 1 h at room temperature. Coverslips were then incubated overnight in 500 μl of 1% BSA/PBS at 4 °C with 1/500 anti-FLAG (Sigma, F1804) and 1/500 anti-HA (Proteintech, NB600-362), adding to specific samples 1/250 Gm130 (Abcam, ab52649) or 1/250 anti-pan-cadherin (Abcam, ab51034). The day after, coverslips were washed with PBS and incubated with 500 μl of 1/1,000 Donkey anti-mouse Alexa 488 (Thermo Fisher Scientific, A32766), 1/1,000 donkey anti-rabbit Alexa 594 nm (Thermo Fisher Scientific, A21207), 1/1,000 donkey anti-goat Alexa 647 nm (Thermo Fisher Scientific, A-21447) and 0.5 μg ml^−1^ DAPI (Sigma, D9452) in 1% BSA/PBS solution for 1 h at room temperature protected from light. Finally, coverslips were washed with PBS and mounted using Prolong Glass Antifade Mountant (Thermo Fisher Scientific, P36982) for 24 h at room temperature. Images were produced with VisiTech iSIM to produce *Z*-stacks of at least 17 stacked images of 0.125 μm width per field.

Fluorescence signal of each channel in the region of interest was measured using built-in tools in Fiji software, then normalized and plotted using Prism 9.0 (ref. ^56^).

### Metabolomics

#### Acyl- and probe-coenzyme A analysis

HEK293T cells were seeded in six-well plates, grown to 70% confluency in media containing 0.5% FBS and treated with 30 μM YnPal or 18-Bz for 2 h. Cells were dislodged into their growth media by pipetting and pelleted by centrifugation (500*g*, 5 min). The cell pellet was washed twice by resuspending in ice-cold PBS and pelleting by centrifugation.

#### Sample extraction

To each sample, 400 µl chloroform was added and vortexed for ~1 min, followed by addition of 200 µl methanol and a repeated vortex. Samples were incubated in a water bath sonicator (4 °C, 1 h), with 3 × 8 min sonication pulses, followed by centrifugation (4 °C, 10 min, 17,000*g*). The supernatant was transferred to a new Eppendorf 1.5 ml tube (E1). The pellet was re-extracted with 450 µl methanol:water (2:1 vol/vol, containing internal standard, ^13^C_3_-Malonyl-CoA), sonicated (8 min, 4 °C) and centrifuged, as above. The supernatant was added to the first extract (E1). Combined extracts were dried using a SpeedVac concentrator, resuspended in 350 µl chloroform:methanol:water (1:3:3, vol/vol) and centrifuged, as above. The upper, aqueous phase containing the polar metabolites (including probe, probe-CoA and acyl-CoA molecules) was dried using the SpeedVac concentrator and resuspended in 100 µl acetonitrile/ammonium carbonate 20 mM (7:3, vol/vol) for LC–MS injection.

#### LC–MS

##### Chromatography conditions

Chromatography before all MS was performed using an adaptation of a method described previously^57^. Samples were injected into a Dionex UltiMate 3000 LC system (Thermo Fisher Scientific) with a Phenomenex Luna C18(2) 100 Å (100 × 2 mm, 3 μm) column coupled with a SecurityGuard C18 guard column (4 × 2 mm). Analytes were separated using 20 mM ammonium carbonate in water (Optima HPLC grade; Sigma-Aldrich) as solvent A and acetonitrile (Optima HPLC grade; Sigma-Aldrich) as solvent B at 0.3 ml min^−1^ flow rate. Elution began at 5% solvent B, maintained for 3 min, increased to 100% B over 12 min, followed by a 3 min wash of 100% B and subsequent 3 min re-equilibration to 5% B. Other parameters were as follows: column temperature, 30 °C; injection volume, 10 μl; needle wash, 50% methanol; autosampler temperature, 4 °C.

#### High-resolution MS

Postchromatography, high-resolution MS was performed with positive and negative polarity switching using a Q-Exactive Orbitrap (Thermo Fisher Scientific) with a heated electrospray ionization (HESI-II) probe. MS parameters were as follows: spray voltage, 3.5 kV and 3.2 kV (for positive and negative modes, respectively); probe temperature, 320 °C; sheath and auxiliary gases, 30 and 5 arbitrary units (a.u.), respectively; full scan range: 100–1,300 *m*/*z* with settings of AGC target and resolution as balanced and high (3 × 10^6^ and 70,000), respectively. Data were recorded using Xcalibur 3.0.63 software (Thermo Fisher Scientific). Mass calibration was performed for both electrospray ionsation (ESI) polarities before analysis using the standard Thermo Fisher Scientific Calmix solution. Qualitative analysis was performed using Xcalibur FreeStyle 1.8 SP1 and Tracefinder 5.1 software (Thermo Fisher Scientific) according to the manufacturer’s workflows. Masses, retention times and fragmentation of all relevant sample-derived molecules were compared to authentic chemical standards.

#### MS/MS

MS parameters were optimized by direct infusion of 16 μM acyl-CoAs dissolved in 10 mM MeOH/ammonium acetate at 5 μl min^−1^ into a TSQ Quantiva triple quadrupole MS (Thermo Fisher Scientific). The heated electrospray was set in positive mode with the following parameters: capillary voltage, 3,472 V; sheath gas, 60 a.u.; aux gas, 10 a.u.; sweep gas, 1 a.u.; ion transfer tube temperature, 325 °C; vaporizer temperature, 275 °C. A selected reaction monitoring function was applied for the simultaneous detection of acyl-CoA and probe-CoA molecules with RF lens and collision energies as shown in Supplementary Table 7. Data were recorded using Xcalibur 4.0.27.10 software and analyzed using QuanBrowser 4.5.445.18 (Thermo Fisher Scientific).

### Lipidomics methods

#### Lipid extraction

HEK293T cells were seeded in six-well plates, grown to 70% confluency and treated with bumped fatty acid probes (15 µM) for 4 h. Cells were dislodged into their growth media by pipetting and pelleted by centrifugation (500*g*, 5 min). The cell pellet was washed 2× with ice-cold PBS and pelleted by centrifugation. Subsequently, the cells were resuspended in 500 µl of ice-cold 150 mM ammonium bicarbonate. An aliquot (10%) was kept aside for protein concentration determination, and the remaining sample was snap-frozen in liquid nitrogen and stored at −80 °C until further processing. For protein concentration determination, cells were lysed in M-PER Mammalian Protein Extraction Reagent (Thermo Fisher Scientific, 78501), and protein content was determined using the Pierce BCA Protein Assay Kit (Thermo Fisher Scientific, 23227) as per the manufacturer’s instructions. An aliquot equivalent to 100 µg protein per sample was used for lipid extraction. Lipids were extracted by the methyl-tert-butyl ether (MTBE) method with minor modifications^58^. Extractions were performed in glass vials fitted with Teflon-lined caps using MS-grade solvents and water. Glass pipettes were used to handle any MTBE-containing solutions or lipid extracts. Methanol (1.5 ml) was added, and the protein sample was vortexed. MTBE (5 ml) was added, and the mixture was incubated for 1 h at room temperature on a shaker. Phase separation was induced by the addition of water (1.25 ml) followed by incubation for 10 min at room temperature. The sample was centrifuged (1,000*g*, 10 min), and the upper organic phase was collected. The lower aqueous phase was re-extracted by addition of 1.67 ml of solvent mixture comprising MTBE/methanol (10:3, vol/vol) and 0.32 ml water. The samples were vortexed, incubated for 10 min and centrifuged (1,000*g*, 10 min). The upper phase was recovered, and the combined organic phases were evaporated at 37 °C under a stream of nitrogen and stored at −20 °C. Before analysis, lipid extracts were reconstituted in 100 µl loading buffer (isopropanol/water/acetonitrile, 2:1:1, vol/vol/vol). Blank control extraction was performed on a 200 µl aliquot of 150 mM ammonium bicarbonate solution. Quality control (QC) samples were prepared by pooling a small aliquot of all experimental samples after resuspension in the loading buffer.

#### Ultrahigh-performance LC–MS (UHPLC–MS) analysis of lipid extracts

UHPLC–MS analysis was performed on a 1290 Infinity II UHPLC system coupled to a 6550 iFunnel quadrupole time-of-flight (QTOF) mass spectrometer (Agilent Technologies). The reversed-phase chromatography protocol was optimized with minor modifications from Cajka and Fiehn^59^. Extracted lipids were separated on an Acquity UPLC CSH C18 column (130 Å, 1.7 μm, 2.1 × 100 mm) fitted with an Acquity UPLC CSH C18 VanGuard precolumn (130 Å, 1.7 µm, 2.1 mm × 5 mm; both waters). The column was maintained at 65 °C at a flow rate of 0.6 ml min^−1^. The mobile phases used were 60:40 (vol/vol) acetonitrile/H_2_O (solvent A) and 10:90 (vol/vol) acetonitrile/isopropanol (solvent B). Solvents A and B were supplemented with 10 mM ammonium formate and 0.1% formic acid for ESI-positive mode and with 10 mM ammonium acetate for ESI-negative-mode analysis. UHPLC gradient elution was carried out as follows: 15–30% solvent B for 0−2 min; 30–48% solvent B for 2–2.5 min; 48–82% solvent B for 2.5−11 min; 82–99% solvent B for 11–11.5 min; 99% solvent B for 11.5–14.50 min. The gradient was returned to initial conditions over 0.5 min, and the column was equilibrated for 3 min before subsequent runs. Between injections a 100% isopropanol needle wash was performed. For negative mode, 5 µl (MS mode) or 10 µl (MS/MS mode) of the sample was injected, and for positive mode, 4 µl (MS mode) or 8 µl (MS/MS mode) of the sample was injected. Samples were injected in randomized order, with QC sample injections added to the start, middle and end of each sample sequence to ensure consistency and reproducibility of all acquisition parameters. Samples were loaded in random order by blinded selection from pooled anonymously labeled samples.

Electrospray parameters were set as follows: gas and sheath gas temperature, 200 °C; drying gas flow, 14 l min^−1^; sheath gas flow, 11 l min^−1^; sheath gas temperature, 350 °C; nebulizer pressure, 35 psig; capillary voltage, 3,000 V; nozzle voltage, 1,000 V. MS-TOF fragmentor and Oct 1 RF Vpp radio voltage were set to 350 and 750 V, respectively. The QTOF was calibrated and operated in the extended dynamic range mode (∼2 GHz) in the mass range of 50–1,700 *m*/*z*. Spectra were acquired in centroid mode with an acquisition rate of 2 spectra per second for MS mode acquisition. Data were acquired in MS mode for quantitative analysis of the natural lipidome and in MS/MS mode to obtain data for lipid structure assignment.

MS/MS data were acquired in auto-MS/MS mode (data-dependent). Spectra were acquired in centroid mode with an acquisition rate of 1 and 5 spectra per second for MS and MS/MS acquisition, respectively. Collision energy was adjusted to −35 eV and 30 eV for negative and positive modes, respectively. Mass range for precursor selection was 300–1,650 *m/z* (negative) and 250–1,680 *m/z* (positive). Fragmentation was triggered if the precursor reached 5,000 (negative) or 2,000 (positive) counts, and the maximum precursors per cycle was set to 5. MS/MS isolation width for precursors was selected as narrow (1.3 *m/z*). Active exclusion was enabled, set to exclude after 3 spectra and release after 0.1 min. To improve precursor selection, background ions were added to an exclusion list. For structure determination of probe-derived lipids, a list of preferred precursor ions was generated for each probe to improve MS/MS coverage of features originating from probe metabolism. MS/MS analysis of DMSO control samples was used to confirm the assignment of natural lipids.

#### Quantitative analysis of natural lipidome

Lipid annotations and quantifications were performed following the guidelines of the Lipidomics Standard Initiative (https://lipidomics-standards-initiative.org/). Feature extraction was carried out in Mass Hunter Profinder (v. 10.0, Agilent Technologies) using the ‘Batch Targeted Feature Extraction’ option. Features were matched to an in-house library containing mass and retention time information of lipid species including glycerophospholipids, sphingolipids, fatty acids and glycolipids. All lipids in the database were previously assigned from MS/MS data using MS-DIAL^60^ followed by manual curation. H^+^, Na^+^ and NH_4_^+^ adducts were selected for positive mode, and H^−^, C_2_H_3_O_2_^−^ and CHO_2_^−^ adducts were selected for negative-mode data. Both mass and retention time were required for feature matching. Match tolerance was set to 5 ppm for mass and 0.15 min for retention time. The EIC extraction range was limited to ± 0.3 min of the expected retention time. An overall score of >70 was required for feature matching, with the contribution to overall score set as follows: mass score 100, isotope abundance score 60, isotope spacing score 50 and retention time score 100. Features over 20% of the saturation limit were excluded from the dataset. Matched features were manually inspected and re-integrated where required and checked for the correct adduct pattern for the relevant lipid class. Data were exported as .csv files containing the identity, peak area and the retention time of each lipid species. Further data analysis and data representation were performed in Excel and GraphPad Prism. The relative abundance of each lipid species within a class was calculated as a percentage of the summed peak areas of all species identified within the class. Triglyceride (TG) species were quantified from data acquired in positive mode, while all other species were quantified from data acquired in negative mode (*n* = 5 for each experimental condition).

#### Assignment of probe-derived lipids

Feature extraction of data acquired in MS mode was carried out in Mass Hunter Profinder (v. 10.0, Agilent Technologies) using the ‘Batch Recursive Feature Extraction (small molecule/peptide)’ option. Samples were grouped according to experimental conditions. All parameters except those detailed below were used as preset by the program. Peak heights were set to a minimum of 3,000 counts. H^+^, Na^+^ and NH_4_^+^ adducts were selected for positive mode, and H^−^, C_2_H_3_O_2_^−^ and CHO_2_^−^ adducts were selected for negative mode. For compound binning and alignment, retention time tolerance was set to (±0% + 0.15 min) and mass tolerance to (±5 ppm + 2 mDa). A minimum free energy score of at least 70 was required in at least 4 of 6 samples per group. For match tolerance, the mass was set to ±10 ppm and retention time to ±0.15 min. The EIC extraction range was limited to ±0.15 min of the expected retention time. An overall score of >75 was required for feature matching, with the contribution to the overall score set as follows: mass score 100, isotope abundance score 60, isotope spacing score 50 and retention time score 100. Features over 20% of the saturation limit were excluded from the dataset. Postprocessing filters were set to require a score (Tgt) of at least 50 in 4 of 6 samples per experimental group.

Manual filtering was performed to remove features present in the blank extraction samples. To create a list of features originating from probe metabolism, only features unique to each probe condition were selected. All features present in DMSO control samples were discarded. Features were manually inspected and re-integrated where required. The feature lists were used to create inclusion lists for MS/MS analysis and peak lists for lipid annotations as described below.

LipidMatch (v. 3.5)^61^ was used for assignment of probe-derived lipids. Lipid libraries containing theoretical fragments of probe-derived lipid species of different classes (PC, PE, PC-O, PE-O, PC-P, PE-P, Cer-NS, Cer-NDS, diglyceride (DG) and TG) were constructed and added to the existing library folder. Agilent .d files were converted to .ms2 format using MSConvertGUI (ProteoWizard)^62^. The search was performed using the feature tables created above. Search parameters were set as follows: retention time window, ±0.15 min; ppm window for matching experimental and in silico fragments, ±5 ppm; mass accuracy window for matching experimental and in silico precursors, ±0.005 Da; MS/MS isolation window, 1 Da; minimum signal intensity for MS/MS ion, 1; minimum number of scans for confirmation, 1. All lipid assignments were manually curated by inspecting MS/MS spectra and ensuring correct adduct formation for the relevant lipid class. Additionally, a small number of lipids were assigned manually from MS/MS spectra. The retention time of all probe-derived lipid species matched the expected retention time window for the relevant lipid class. Note that in contrast to natural DG and TG species, which preferentially form NH_4_^+^ and Na^+^ adducts, probed-derived DG and TG species preferentially formed the (M + H)^+^ ion, presumably through protonation at the amide moiety.

### Quantitative MS-based proteomics

#### On-bead hydrolysis proteomics

Cells were concurrently plated and transfected with 1 µg ml^−1^ of the appropriate ZDHHC construct using 3 µl of FuGene per 1 µg of DNA in a 10 cm dish. After 6 h, the media was refreshed and cells were incubated overnight. Cells were treated with 15 µM of the appropriate probe in 0.5% FBS media for 8 h. Cells were then washed 3× with ice-cold PBS then lysed in SDS lysis buffer (50 mM HEPES (pH 7.4), 0.5% NP-40, 0.25% SDS, 10 mM NaCl, 2 mM MgCl_2_, EDTA-free cOmplete protease inhibitor (Roche,11873580001) and 0.05 U μl^−1^ benzonase (Merck, E1014). Lysates were adjusted to 2 mg ml^−1^, using ~2 mg per condition, and then subjected to a click reaction using biotin-PEG3-azide as described above before EDTA quenching followed by chloroform/methanol precipitation.

For those samples where no site ID was performed, protein samples were suspended in 50 mM HEPES (pH 7.4) containing 1% SDS and then precipitated with chloroform/methanol. For experiments including site identification of lipidation, proteins were solubilized in 1 ml 50 mM triethanolamine (pH 7.5), 4% SDS and 5 mM EDTA. TCEP (10 mM) was added to samples and incubated for 20 min at room temperature with agitation. To these solutions, 25 mM *N*-ethylmaleimide (NEM, from 1 M stock in EtOH) was added and incubated for 2 h at room temperature with agitation. Samples were then precipitated with chloroform/methanol.

All protein samples were then suspended in 50 mM HEPES (pH 7.4) containing 2% SDS and then diluted to 0.2% SDS using 50 mM HEPES (pH 7.4). Labeled proteins were enriched using NeutrAvidin agarose beads (Thermo Fisher Scientific, 29200) using 40 μl (50% slurry) per condition. Beads were washed with 0.2% SDS in 50 mM HEPES (pH 7.4) before incubation with the samples for 3 h at room temperature with agitation. Beads were then washed 2× with 0.2% SDS in 50 mM HEPES (pH 7.4) and 4× with 50 mM HEPES (pH 7.4). Beads were then suspended in 20 µl of 50 mM triethanolamine (pH 7.5), 4 mM EDTA and 0.5% ProteaseMAX (Promega, V2071). To this was added 10 µl of the NH_2_OH cleavage solution (100 µl of 8.16 M NH_2_OH in 50 mM TEA (pH 8.0), 30 µl of 500 mM triethanolamine (pH 7.5), 2.6 µl of 500 mM EDTA and 197.4 µl water) giving a final NH_2_OH concentration of 0.82 M, and the samples were incubated at room temperature for 2 h with agitation. Following this, 100 µl of 50 mM HEPES (pH 8.0) containing 5 mM TCEP was added, the beads were pelleted and 120 µl of supernatant was taken. In total, 20 mM chloroacetamide was added to the supernatant, and they were incubated at room temperature for 15 min. Samples were then diluted with 400 µl HEPES (50 mM, pH 8.0) and digested with 0.3 µg of trypsin (Promega, V5111) o/n at 37 °C. Samples were acidified with 0.5% (vol/vol) trifluoroacetic acid (TFA), flash frozen and lyophilized. Samples were dissolved in water containing 0.5% TFA and loaded onto stage tips containing three polystyrenedivinyl-benzene (SDB-XC) copolymer discs (Merck, 66884-U). The stage tipping procedure was carried out as described in ref. ^63^. Peptide samples were eluted in 55% acetonitrile in water, and the solvent was removed by incubation in an Eppendorf Concentrator plus at 45 °C.

Samples in which site ID was not performed were dissolved in water containing 0.1% TFA, ready for LC–MS/MS analysis. For samples where site ID was performed, before LC–MS/MS analysis samples were 3× SCX fractionation using stage tips loaded with three layers SDB–RPS discs (3M Empore). Peptides were loaded on the solid phase in 0.5% TFA and subsequently washed 3× with 60 µl 0.2% TFA. Peptides were eluted using the following three elution buffers (60 µl): buffer 1 (100 mM ammonium formate, 40% (vol/vol) MeCN, 0.5% (vol/vol) formic acid), buffer 2 (150 mM ammonium formate, 60% (vol/vol) MeCN, 0.5% (vol/vol) formic acid) and buffer 3 (5% (vol/vol) ammonium hydroxide, 80% (vol/vol) MeCN). Samples were dried in an Eppendorf Concentrator plus at 45 °C and then dissolved in water containing 0.1% TFA, ready for LC–MS/MS analysis. Peptides were analyzed on a Q-Exactive mass spectrometer (Thermo Fisher Scientific) coupled to an Ultimate 3000 LC (Thermo Fisher Scientific) using an Easy Spray Nano-source. The instrument was operated in data-dependent acquisition mode selecting the ten most intense precursor ions for fragmentation.

#### YnPal KO proteomics

A total of 6 × 10^6^ cells from HEK293T, HEK293T ZDHHC20 CRISPR knockdown cell line and two HEK293T ZDHHC20 CRISPR KO clonal lines were seeded in triplicate into 10 cm dishes and left o/n at 37 °C. Cells were then treated with 15 μM YnPal or 15 μM palmitic acid and incubated for 8 h at 37 °C. Cells were then washed 3× with PBS and then lysed in SDS lysis buffer. Lysates were adjusted to 2 mg ml^−1^, using 2 mg per condition, and then subjected to a click reaction using biotin-PEG3-azide as described above. The reaction was quenched with 5 mM EDTA followed by chloroform/methanol precipitation. Protein pellets were washed and sonicated 2× with 1 ml MeOH, and samples were processed via on-bead digestion.

#### YnPal ZDHHC20 proteomics

Cells were transfected as described above and then left overnight. Cells were then treated with 25 µM YnPal in full media for 4 h. Cells were then washed 3× with ice-cold PBS, lysed in SDS lysis buffer, spun at 14 kG for 10 min at 4 °C and then the clarified lysates were equalized and adjusted to 2 mg ml^−1^. Lysates were clicked as described above using biotin-PEG3-azide, quenched with EDTA and then precipitated using methanol/chloroform. Samples were processed as described for on-bead digestion with the following addition. Samples were dissolved in 1% SDS, 150 mM NaCl, 1 mM EDTA and 50 mM HEPES (pH 7). Samples were then diluted with buffer or buffer plus neutralized NH_2_OH to a concentration of 1 M and were then shaken at room temperature for 1 h. Samples were precipitated using methanol/chloroform; then dissolved again in 1% SDS, 150 mM NaCl, 1 mM EDTA, and 50 mM HEPES (pH 7); then diluted with buffer or buffer plus neutralized NH_2_OH to a concentration of 0.66 M and then heated to 90 °C for 5 min. Samples were precipitated using methanol/chloroform then followed by the methodology described in the on-bead digestion section and followed by labeling with a TMT-6plex and high pH reversed-phase fractionation (both described below).

#### TREX ZDHHC20 proteomics

TREX HEK293T cells were seeded and left overnight in an incubator at 37 °C in standard media supplemented with 1 μg ml^−1^ blasticidin and 50 μg ml^−1^ hygromycin B in quadruplicate. On the day before the treatment of cells with the appropriate probes, media were supplemented with 1 μg ml^−1^ of doxycycline or water. Cells were treated with 15 µM of C18-Bz in 0.5% FBS media for 8 h.

#### On-bead digestion

All samples were then dissolved in 1% SDS in PBS and then diluted to 0.2% SDS with PBS. Biotinylated proteins were then enriched on a 1:1 mixture of dimethylated NeutrAvidin agarose beads^64^ and control agarose beads (Thermo Fisher Scientific, 20333), which had been prewashed 2× 0.2% SDS PBS, for 3 h at room temperature. Beads were washed 3× with 1 ml SDS (0.2%) in PBS followed by 2× with 1 ml HEPES (50 mM, pH 7.4) and finally 1× with 1 ml HEPES (50 mM, pH 8.0). Beads were then suspended in 50 µl HEPES (50 mM, pH 8.0) with 400 ng of LysC (Promega, VA1170) for 2 h at 37 °C with agitation. The supernatant was removed and reduced with 5 mM TCEP and alkylated using 15 mM chloroacetamide for 15 min then digested with 100 ng of trypsin o/n at 37 °C (Promega).

Samples were acidified with 0.5% (vol/vol) TFA, and the solvent was removed in an Eppendorf Concentrator plus at 45 °C. Samples were dissolved in water containing 0.5% TFA and after stage tipping using Oasis HLB μElution Plate 30 μm following the manufacturer’s procedure, and elutions were dried in an Eppendorf Concentrator plus at 45 °C. Samples were dissolved in 2% MeCN, 97.9% water containing 0.1% TFA ready for LC–MS/MS analysis. Peptides were analyzed on a Q-Exactive mass spectrometer (Thermo Fisher Scientific) or an Eclipse mass spectrometer coupled to an Ultimate 3000 LC (Thermo Fisher Scientific) using an Easy Spray Nano-source. The instrument was operated in data-dependent acquisition mode selecting the ten most intense precursor ions for fragmentation.

#### Proteomics searches and data analysis for LFQ proteomics

RAW files were uploaded into MaxQuant (version 1.6.7.0) and searched against Uniprot curated human proteome (As of 2019) using the built-in Andromeda search engine. Cysteine carbamidomethylation was selected as a fixed modification and methionine oxidation and acetylation of protein N terminus as variable modifications. For site ID experiments, cysteine NEM modification and carbamidomethylation were selected as variable modifications. Trypsin/P was set as the digestion enzyme, up to two missed cleavages were allowed and a false discovery rate (FDR) of 0.01 was set for peptides, proteins and sites with match between runs selected. Data were quantified using LFQ with a minimum ratio count = 2.

Data analysis was performed using Perseus (version 1.6.2.1). MaxQuant proteingroups.txt output files were uploaded and filtered against contaminants, reverse and proteins identified by site. A base 2 logarithm was applied to all LFQ intensities. On-bead hydrolysis datasets were filtered to contain ≥3 valid values in the positive (mutant) condition. Missing values were imputed from a normal distribution (width = 0.3 and downshift = 1.8). YnPal KO samples data were filtered for valid values in at least 2/3 of each condition for all analyses except when comparing against palmitic acid, where only 2/3 YnPal-treated samples were considered and then missing values were imputed from a normal distribution (width = 0.3 and downshift = 1.8; Extended Data Fig. 7g). TREX samples were filtered for ≥3 valid values in the positive (mutant) condition with no imputation performed. Within the YnPal datasets, the data were normalized by subtracting the median value from each column. A two-tailed unpaired Student’s *t* test was performed comparing the various sets of condition groupings (*S*_0_ = 0.1/0.5, adjusted FDR = 0.01/0.05) for all proteins remaining in the dataset, and the results were analyzed according to their statistical significance.

#### TurboID proximity labeling

A total of 6 × 10^6^cells stably expressing TurboGFP clones 1 and 2 and ZDHHC20 clones with C- or N-terminally fused TurboID were plated in duplicate in 10 cm dishes. After reaching 80% confluency, cells were treated with 500 μM biotin for 3 h, cooled on ice, collected and lysed in SDS lysis buffer (described above). A BCA assay was performed, and two 1 mg portions of each lysate (*n* = 4 independent biological replicates for each cell line) at 1 mg ml^−1^ were added to dimethylated neutravidin-agarose beads and agitated at room temperature for 3 h. Beads were washed 3× with 0.2% SDS HEPES (50 mM, pH 7.4) and 3× with HEPES (50 mM, pH 7.4). Beads were then suspended in 50 mM HEPES (pH 8.0) containing 400 ng LysC for 1 h at 37 °C. The supernatant was reduced with 5 mM TCEP and alkylated using 15 mM chloroacetamide for 15 min and then digested with 100 ng of trypsin o/n at 37 °C (Promega). Samples were then concentrated in an Eppendorf Concentrator plus at 45 °C for TMT-10Plex labeling and SCX fractionation.

#### TMT labeling

Samples were labeled with a TMT-6Plex or TMT-10Plex as described here^24^, and the combined solvent was removed in an Eppendorf Concentrator plus at 45 °C. Samples were then fractionated either using high pH reversed-phase fractionation (Pierce) following the manufacturer’s protocol or by SCX fractionation with the following method. Samples were redissolved in 1% TFA and loaded on pre-activated three layers of SCX membranes and fractionated six times. Membranes were washed 3× with 60 µl 0.2% TFA. Peptides were eluted using the following six elution buffers (60 µl): buffer 1 (75 mM ammonium formate, 20% (vol/vol) MeCN, 0.5% (vol/vol) formic acid), buffer 2 (125 mM ammonium formate, 20% (vol/vol) MeCN, 0.5% (vol/vol) formic acid), buffer 3 (200 mM ammonium formate, 20% (vol/vol) MeCN, 0.5% (vol/vol) formic acid), buffer 4 (300 mM ammonium formate, 20% (vol/vol) MeCN, 0.5% (vol/vol) formic acid), buffer 5 (400 mM ammonium formate, 20% (vol/vol) MeCN, 0.5% (vol/vol) formic acid) and buffer 6 (5% (vol/vol) ammonium hydroxide, 80% (vol/vol) MeCN). Samples were dried in an Eppendorf Concentrator plus at 45 °C and then dissolved in water containing 0.1% TFA, before analysis on a Q-Exactive mass spectrometer (Thermo Fisher Scientific) coupled to an Ultimate 3000 LC (Thermo Fisher Scientific) using an Easy Spray Nano-source. The instrument was operated in data-dependent acquisition mode selecting the ten most intense precursor ions for fragmentation.

#### TMT proteomic analysis

Data analysis was performed using Perseus (version 1.6.2.1). MaxQuant proteingroups.txt output files were uploaded and filtered against contaminants, reverse and proteins identified by site. A base 2 logarithm was applied to all reporter intensity corrected values, and data were filtered for where valid values were found in at least 8/10 or 5/6 channels. Data were normalized across all samples by subtracting the median across replicates within each TMT multiplex followed by normalizing across the conditions by subtracting the mean value from each column. A two-tailed unpaired Student’s *t* test was performed comparing the various sets of condition groupings (*S*_0_ = 0.1, adjusted FDR = 0.01) for all proteins remaining in the dataset, and the results were analyzed according to their statistical significance.

### Generation of ZDHHC20 KO cell lines

Two guide sequences (gRNA1 and 2) targeting exon 9 or 4, respectively (Supplementary Table 8), were designed using the online tool CHOPCHOP (https://chopchop.cbu.uib.no)^65^ and separately cloned into a plasmid containing Cas9 and the sgRNA scaffold, pSpCas9(BB)-2A-Puro (PX459), using a Fast Digest BbsI restriction strategy coupled with T7 DNA ligase ligation. Plasmids were sequenced by GATC Biotech to confirm subcloning of the gRNA guides. In total, 1 μg of each plasmid was mixed with 250 μl of Opti-MEM before being combined with another mixture containing 5 μl of TransIT-X2 (5 µl per well) in 250 µl Opti-MEM. The combined mixtures were allowed to incubate at room temperature for 20 min before being added to a cell suspension containing 6 × 10^5^ HEK293T cells in a six-well plate. The cells were cultured for 3 d before selection in 1 μg ml^−1^ puromycin for 1 week. Cells were then single-cell sorted into 96-well plates and allowed to expand into 12-well plates before screening by anti-ZDHHC20 and anti-calnexin (loading control) immunoblot. For single-cell sorting, see Molecular cloning.

### Generation of ZDHHC20 T-REx cell lines

Flp-In T-REx 293 cells were transfected with 100 ng of pcDNA/FRT/C-FLAG-D20 WT or C-FLAG-D20(Y181G) alongside 0.9 μg of pOG44 plasmid using FuGene HD replacing with fresh media after 24 h. Cells were then grown for 4 d before selecting resistant clones in media containing 15 μg ml^−1^ blasticidin and 200 μg ml^−1^ hygromycin B over 10 d when resistant clones were pooled. Cells were then grown in standard media supplemented with 1 μg ml^−1^ blasticidin and 50 μg ml^−1^ hygromycin B for 14 d before colonies were pooled and expanded.

### Bioinformatic PANTHER overrepresentation analysis

The online bioinformatic tool PANTHER^66^ was used to perform statistical overrepresentation analysis of ZDHHC20 substrates enriched in at least 2 of 3 cell lines, which are as follows: HEK293T, PANC1 and MDA-MB-231. Two separate analyses were performed using gene ontology (GO) terms cellular compartment and protein class. The cellular compartment (GO-Slim) analysis was performed using the default list of genes from the human genome, whereas the protein class analysis was done with a manually curated list representing the human *S*-acylated proteome. Statistical analysis and *P* values were determined using an FDR-adjusted two-tailed Fisher’s exact test. Results were filtered for those with a −log_10_(*P* value) > 9. The human *S*-acylome contains the combined unique hits between the following filtered SwissPalm lists: the first list was collated by setting the ‘nber_palmitoyl_proteome_hits’ and ‘nber_technique_categories’ ≥2 and the second list was generated by setting the ‘nber_palmitoyl_proteome_hits’ ≤1 and ‘nber_targeted_study_hits’ ≥1. After conversion of Uniprot AC IDs to gene names, the combined list of unique genes totaled 2,429. Results were filtered for those with a −log_10_(*P* value) > 1.5.

### Generation of knock-in ZDHHC20(Y181G) mutant cell line by CRISPR–Cas9

To generate ZDHHC20 Y181G knock-in mutant polyclonal cell line, custom sgRNA and donor ssDNA were synthesized by Thermo Fisher Scientific (A35534), and recombinant Cas9 protein was used, following the manufacturer’s recommendations. In short, 4 × 10^5^ HEK293T cells were seeded per well in a six-well plate a day before transfection. To transfect cells with an assembled ribonucleoprotein (RNP) complex, the following reagents were mixed: 6.25 μg of TrueCut Cas9 v2 protein (Thermo Fisher Scientific, A36496), 1.2 μg of sgRNA (5′-CACGAAAAGGCAAUAUAAUA-3′), 50 pmol of ssDNA (5′- AAATTCTTCCTGCTGTTTTTATTGTATTCCCTA[CTAGGTTGT]CTTTTCGTGGCTGCAACAGTTTTAGAGTACTT-3′, knock-in sequence in brackets), 12.5 μl of Lipofectamine Cas9 Plus Reagent and 7.5 μl of Lipofectamine CRISPRMAX Reagent (Thermo Fisher Scientific, CMAX00001) in 250 μl of Opti-MEM. The RNP mix was added to cells with 2.5 ml of fresh media and placed back in the incubator at 37 °C and 5% CO_2_. Two days after transfection, cells were trypsinized and seeded at 0.8 cells per well density on a 96-well plate to increase the probability of isolating single-cell clones. Cells were then placed back in the incubator, and 2 million cells from the remaining trypsinized cells were pelleted and flash frozen to be used as a control for downstream analyses. Three weeks after seeding the cells, wells that showed only one colony were further expanded in 24-well plates. When monoclonal populations reached 90% confluency, cells were trypsinized and half of them were frozen for storage. From the remaining cells, along with the transfected parental polyclonal cell population, genomic DNA (gDNA) was isolated using Monarch Genomic DNA Purification Kit (NEB, T3010S) following the manufacturer’s recommendations.

To assess if the Y181G sequence had successfully replaced the endogenous sequence in at least one allele, gDNA from each monoclonal cell line was probed by PCR with GoTaq G2 (Promega, M7841) using manufacturer recommendations and with the following program: 95 °C for 2 min for initial denaturation, 30 cycles of denaturation at 95 °C for 20 s, annealing at 55 °C for 30 s, extension at 72 °C for 1 min and final step of extension at 72 °C for 5 min. Forward primer 5′-TGTATTCCCTA [CTAGGTTGT]-3′ (knock-in sequence in brackets) and reverse primer 5′-CCCTATCTGTCCTCTGAT-3′ were used to produce an amplicon of 213 bp, which was resolved in a 2% agarose gel.

After selecting positive clones, a second PCR was carried out in these to confirm by Sanger sequencing that the Y181G mutation sequence had been successfully integrated and that the ORF of ZDHHC20 exon 7 was intact with no indels present. The PCR amplicon was produced using Q5 master mix (NEB, M0492S) and the following program: 98 °C for 30 s for initial denaturation, 33 cycles of denaturation at 98 °C for 10 s, annealing at 68 °C for 30 s, extension at 72 °C for 20 s and a final step of extension at 72 °C for 20 s. The primers used (forward 5′-GGCAGCCTCCATCCTACTTT-3′ and reverse 5′-GCCCTATCTGTCCTCTGATGG-3′) produced an amplicon of 348 bp, which was resolved in a 1.5% agarose gel, recovered using Monarch DNA gel extraction kit (NEB, T1020) following manufacturer’s recommendations and submitted to Genewiz for Sanger sequencing.

### Reporting summary

Further information on research design is available in the Nature Portfolio Reporting Summary linked to this article.

## Online content

Any methods, additional references, Nature Portfolio reporting summaries, source data, extended data, supplementary information, acknowledgements, peer review information; details of author contributions and competing interests; and statements of data and code availability are available at 10.1038/s41587-023-02030-0.

## Supplementary information

Supplementary Information Supplementary Note (synthetic methods and characterization data), Tables 1–9 and Figs. 1–6 and uncropped scans.

Reporting Summary

Supplementary Data Supplementary Data 1–8 including proteomics data.

## Source data

Source Data Fig. 1 Uncropped images of gels and blots.

## Data Availability

The MS proteomics data have been deposited to the ProteomeXchange Consortium via the PRIDE partner repository with the dataset identifiers PXD032373 and PXD032378, and made available. The lipidomics (10.25418/crick.24279541) and metabolomics (10.25418/crick.24279838) datasets have been uploaded to Figshare. PDB ID 6BML was used for Fig. 1. Uncropped gel data are shown in a separately attached supplementary file. An earlier version of this paper has been uploaded to bioRxiv (10.1101/2023.04.18.537386). Source data are provided with this paper.
