# Supplementary material for: A palmitoyl transferase chemical–genetic system to map ZDHHC-specific S-acylation
Source: Nat Biotechnol. 2024 Jan 8;42(10):1548–58. doi: 10.1038/s41587-023-02030-0 (PMC11471619; doi:10.1038/s41587-023-02030-0)
Supplement: Supplementary file 1 — Supplementary Note (synthetic methods and characterization data), Tables 1–9 and Figs. 1–6 and uncropped scans. [file 41587_2023_2030_MOESM1_ESM.pdf]

nature biotechnology

Article https://doi.org/10.1038/s41587-023-02030-0

A palmitoyl transferase chemical–genetic system to map ZDHHC-specific *S*-acylation

In the format provided by the authors and unedited

Table of contents **Page**

1. Synthetic Methods and Characterization Data	2
2. Supplementary Tables	18
3. Supplementary Figures	25
4. Supplementary Information – References	35
5. Uncropped Scans of Supplementary Information blots	36

Synthetic Methods and Characterization Data

Scheme 1. Synthetic scheme to bumped fatty acid probes.

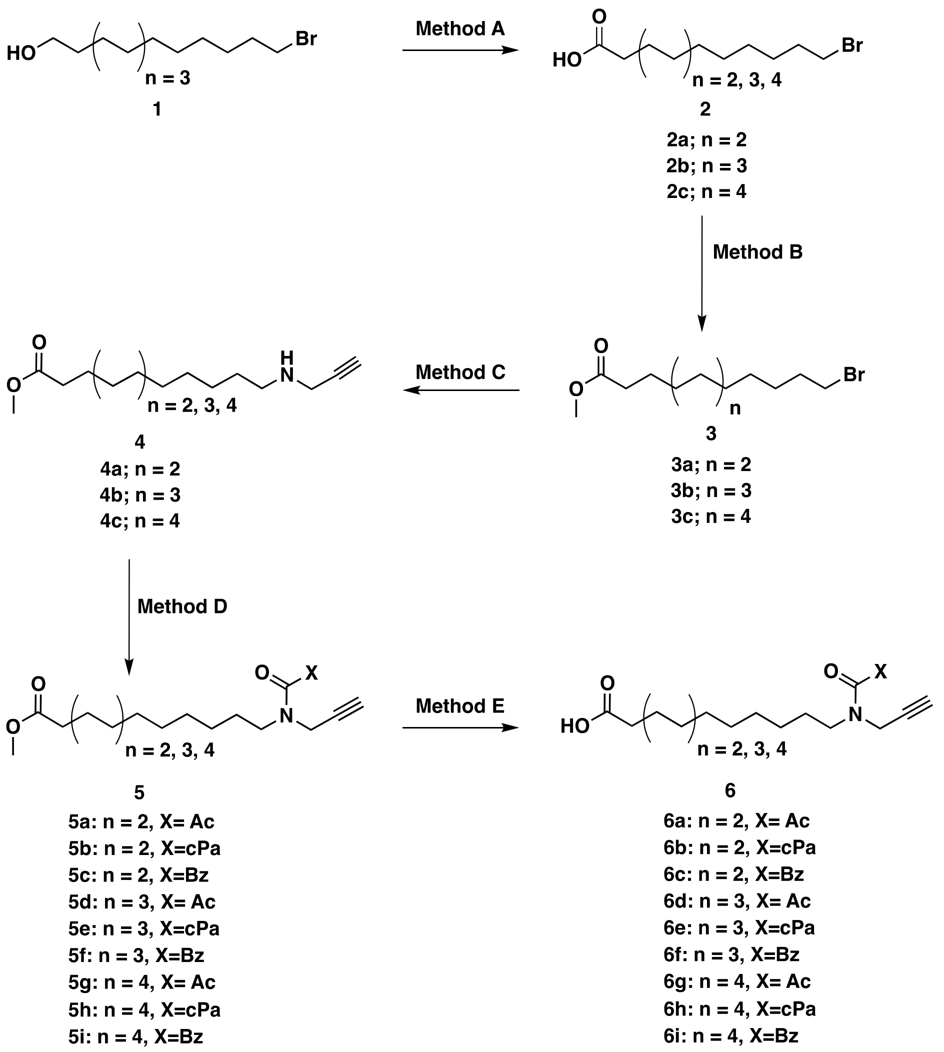

12-(N-(prop-2-yn-1-yl)acetamido)dodecanoic acid (6a; 16-Ac)

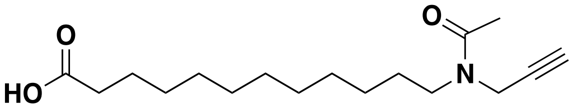

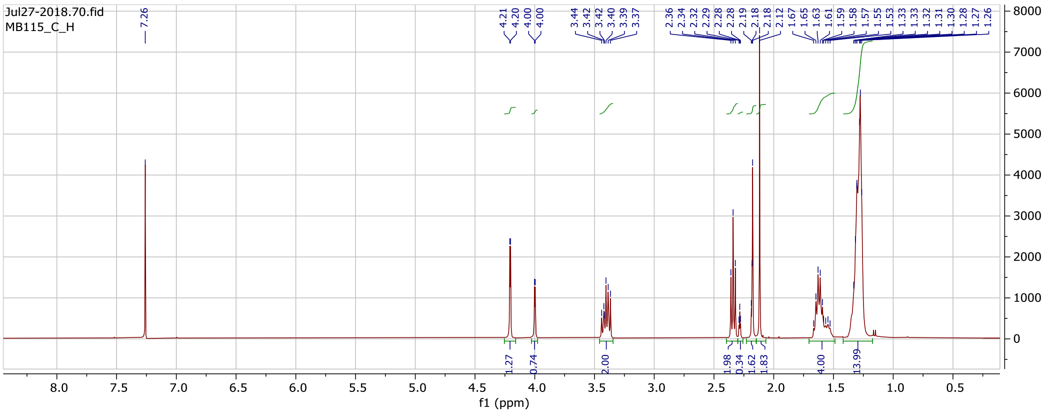

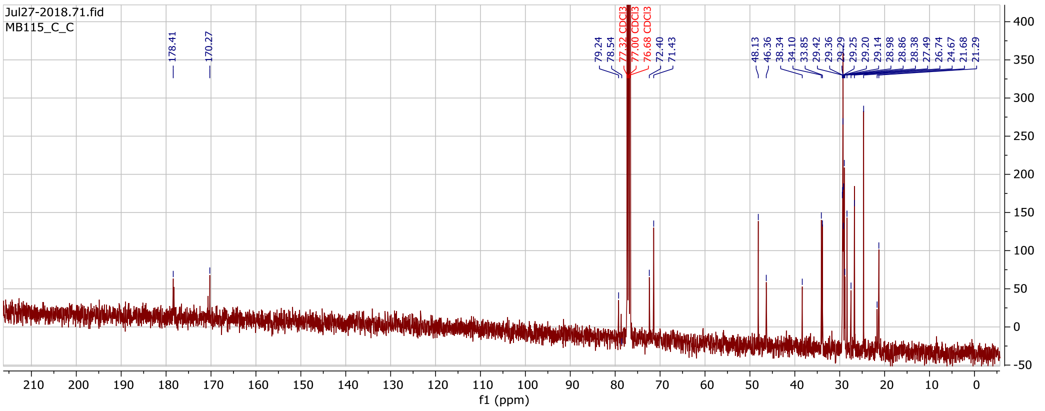

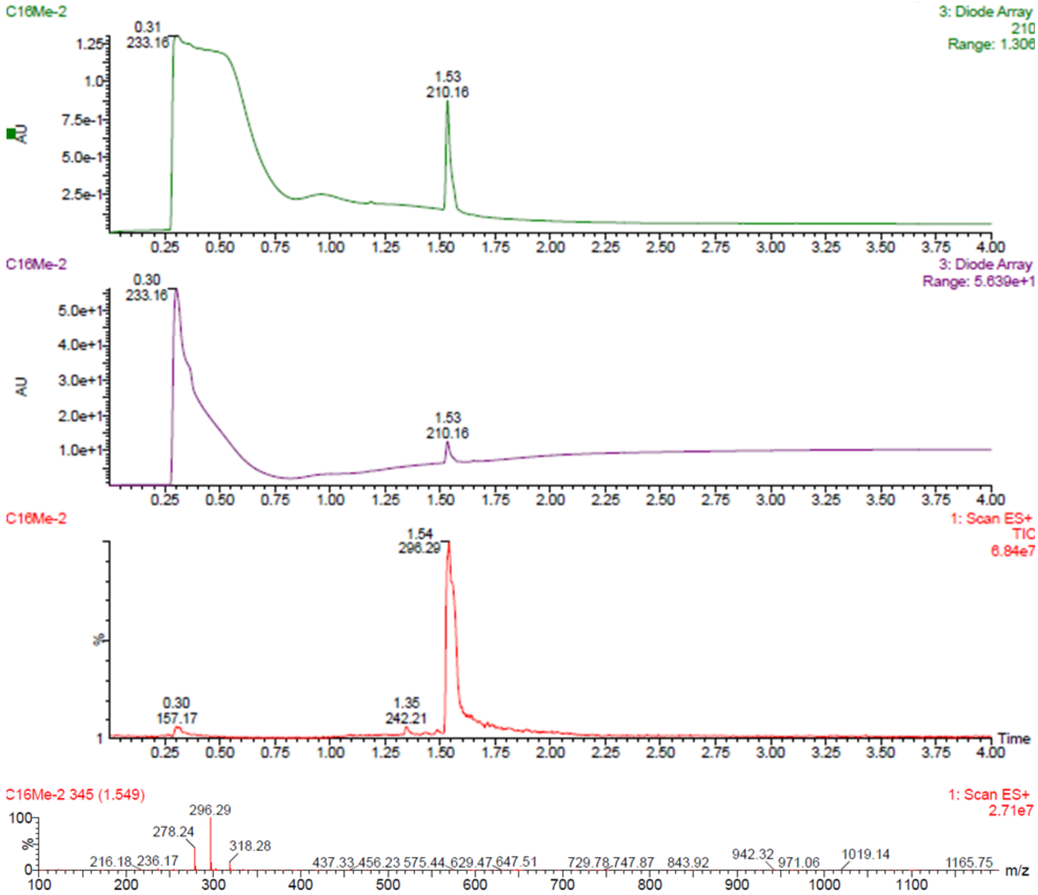

12-(N-(prop-2-yn-1-yl)cyclopropanecarboxamido)dodecanoic acid (6b; 16-cPr)

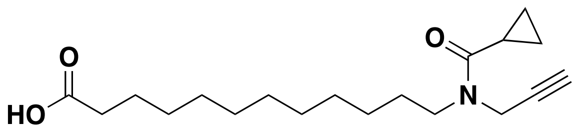

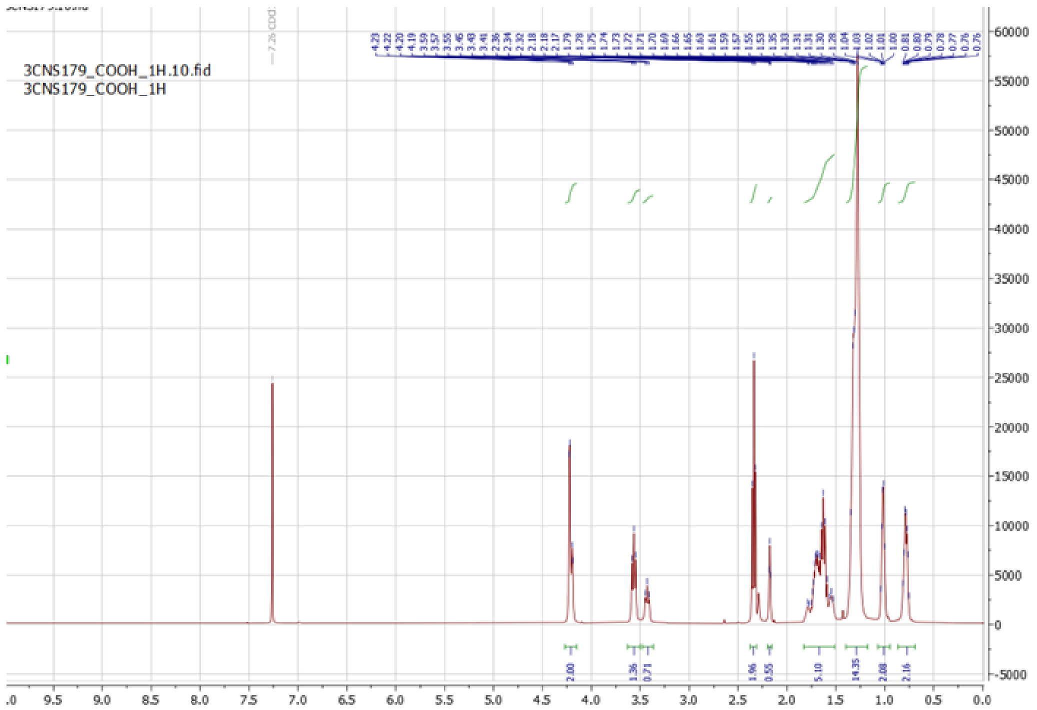

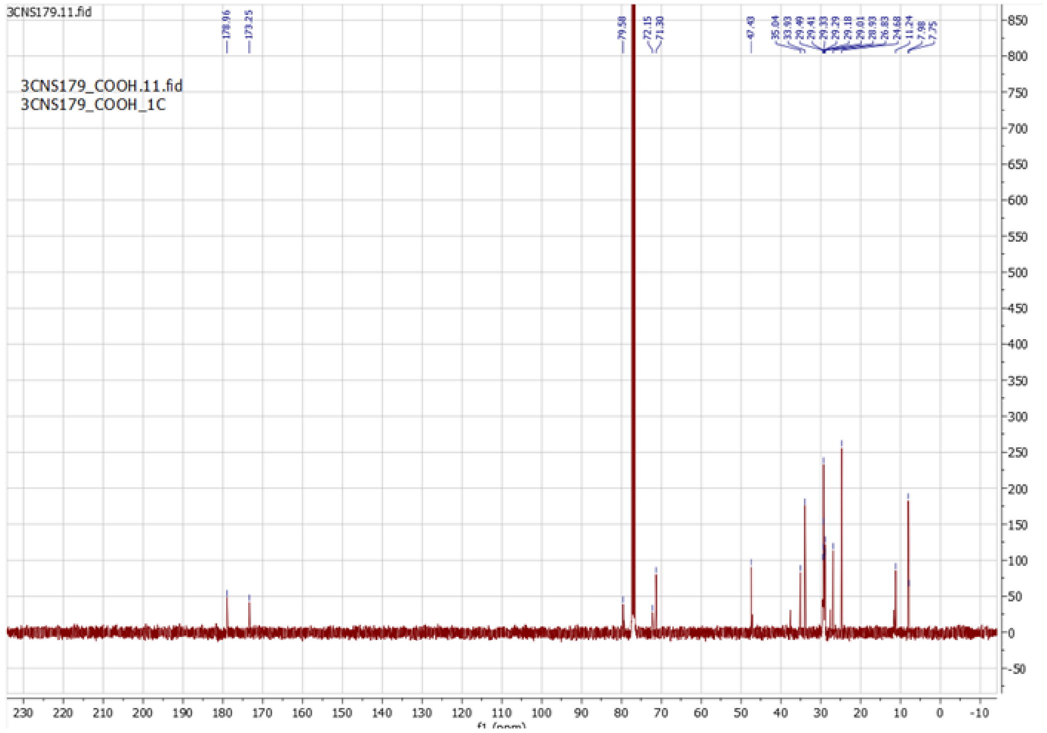
3CNS179.11.fid
3CNS179_COOH.11.fid
3CNS179_COOH_1C
178.96
173.25
79.58
72.15
71.30
47.43
f1 (ppm)

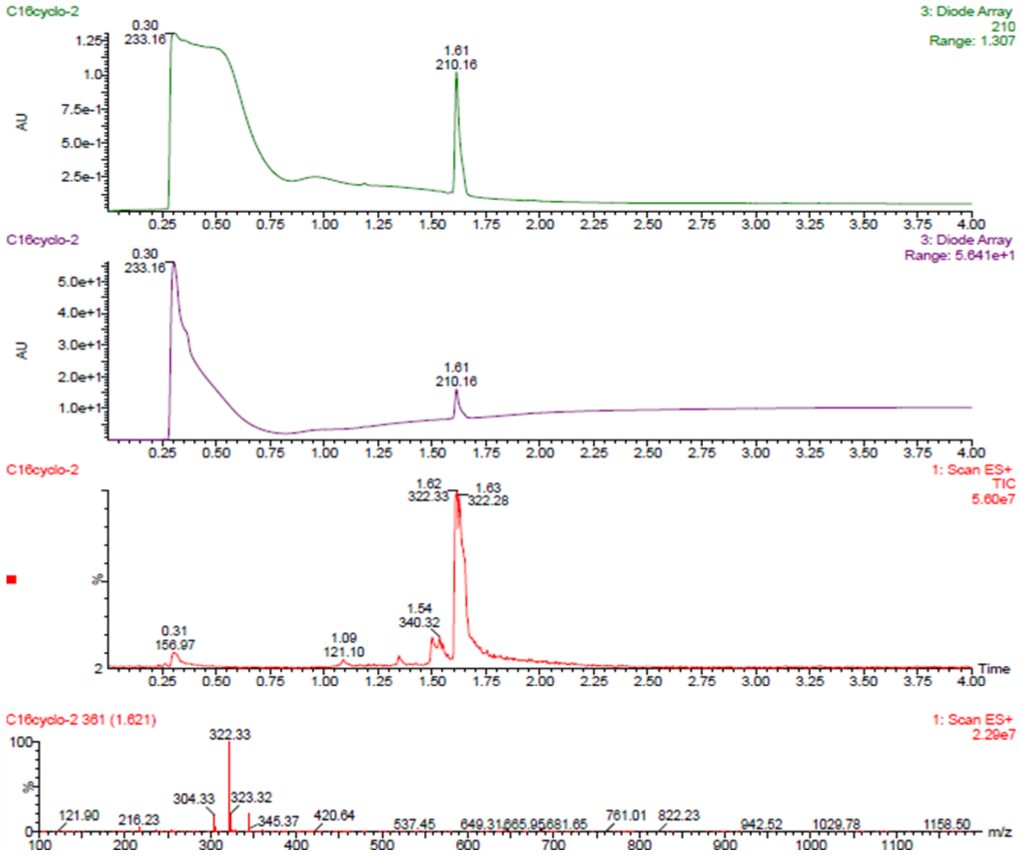
C16cyclo-2
3: Diode Array
210
Range: 1.307
AU
0.30
233.16
1.61
210.16
C16cyclo-2
3: Diode Array
Range: 5.641e+1
AU
0.30
233.16
1.61
210.16
C16cyclo-2
1: Scan ES+
TIC
5.60e7
1.62
322.33
1.63
322.28
1.54
340.32
0.31
156.97
1.09
121.10
Time
C16cyclo-2 361 (1.621)
1: Scan ES+
2.29e7
322.33
304.33
323.32
345.37
420.64
121.90
216.23
537.45
761.01
822.23
942.52
1029.78
1158.50
m/z

12-(N-(prop-2-yn-1-yl)benzamido)dodecanoic acid (6c; 16-Bz)

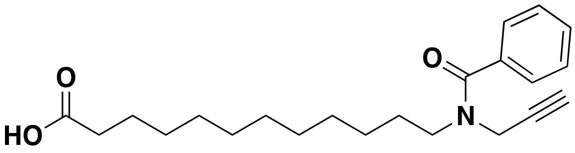

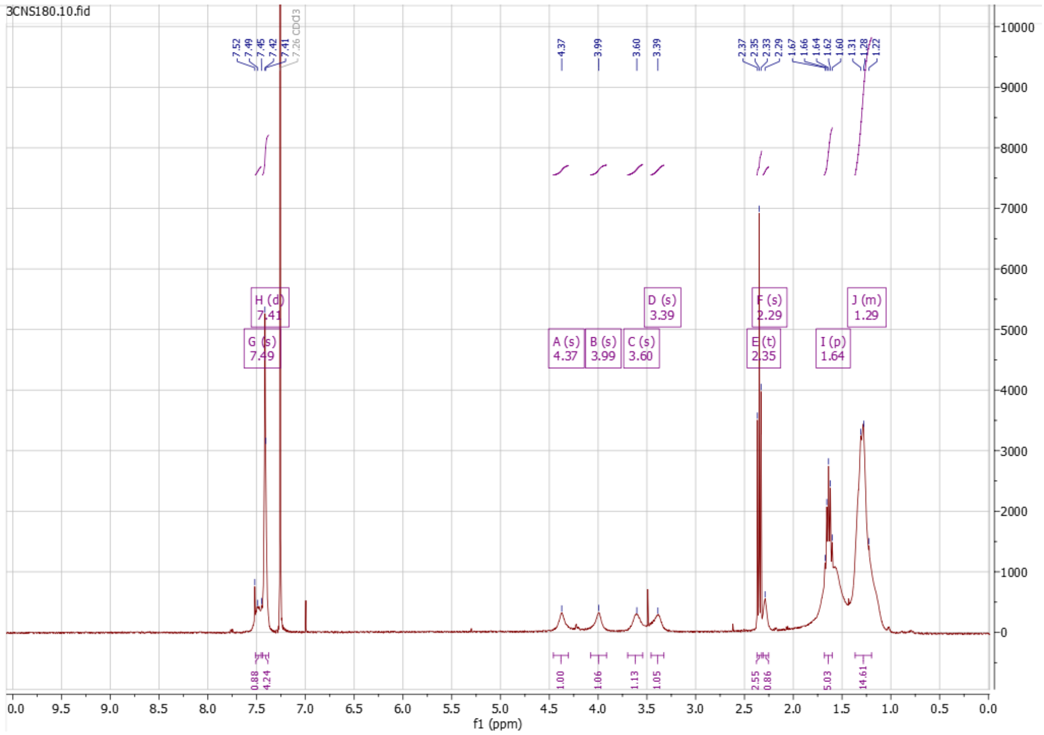

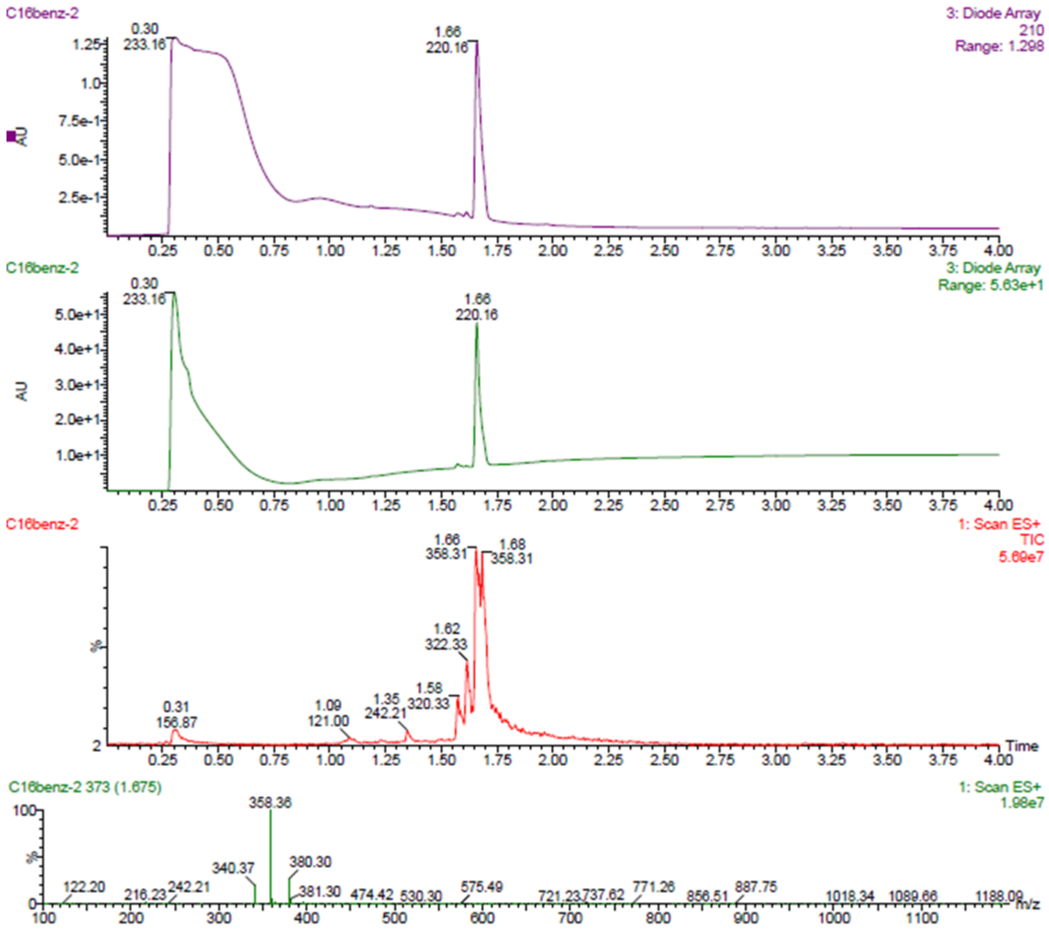

14-(N-(prop-2-yn-1-yl)acetamido)tetradecanoic acid (6d; 18-Ac)

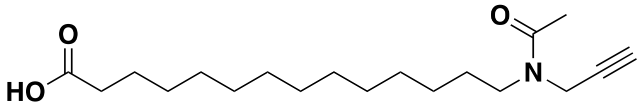

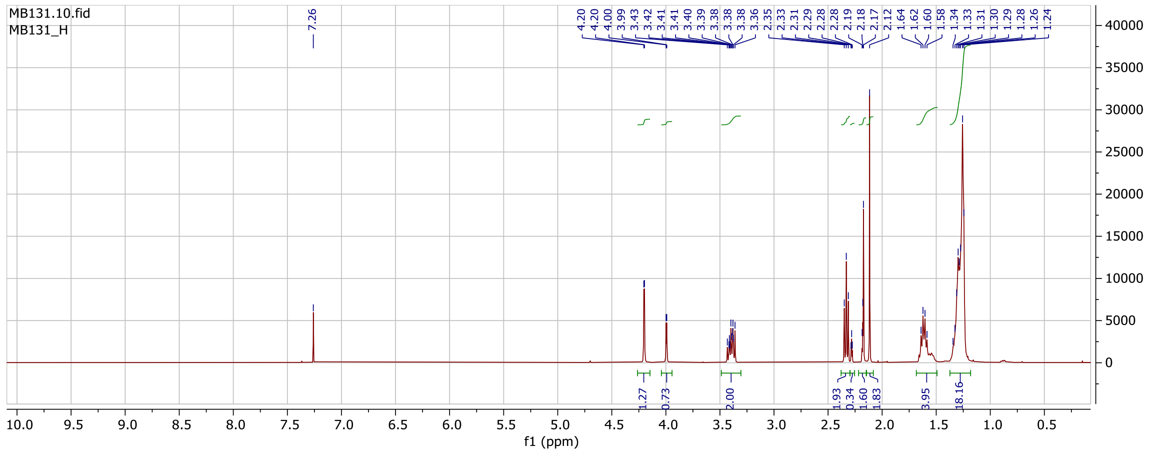

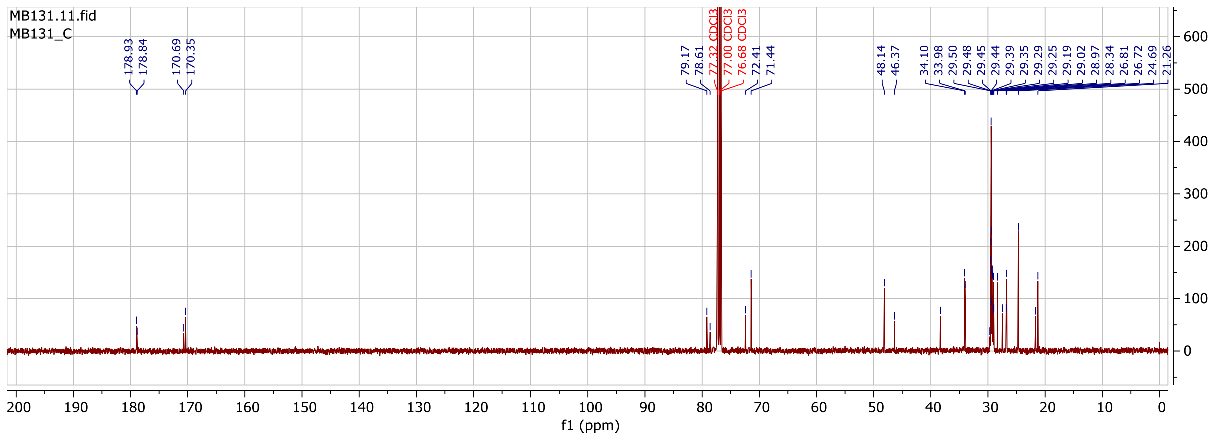

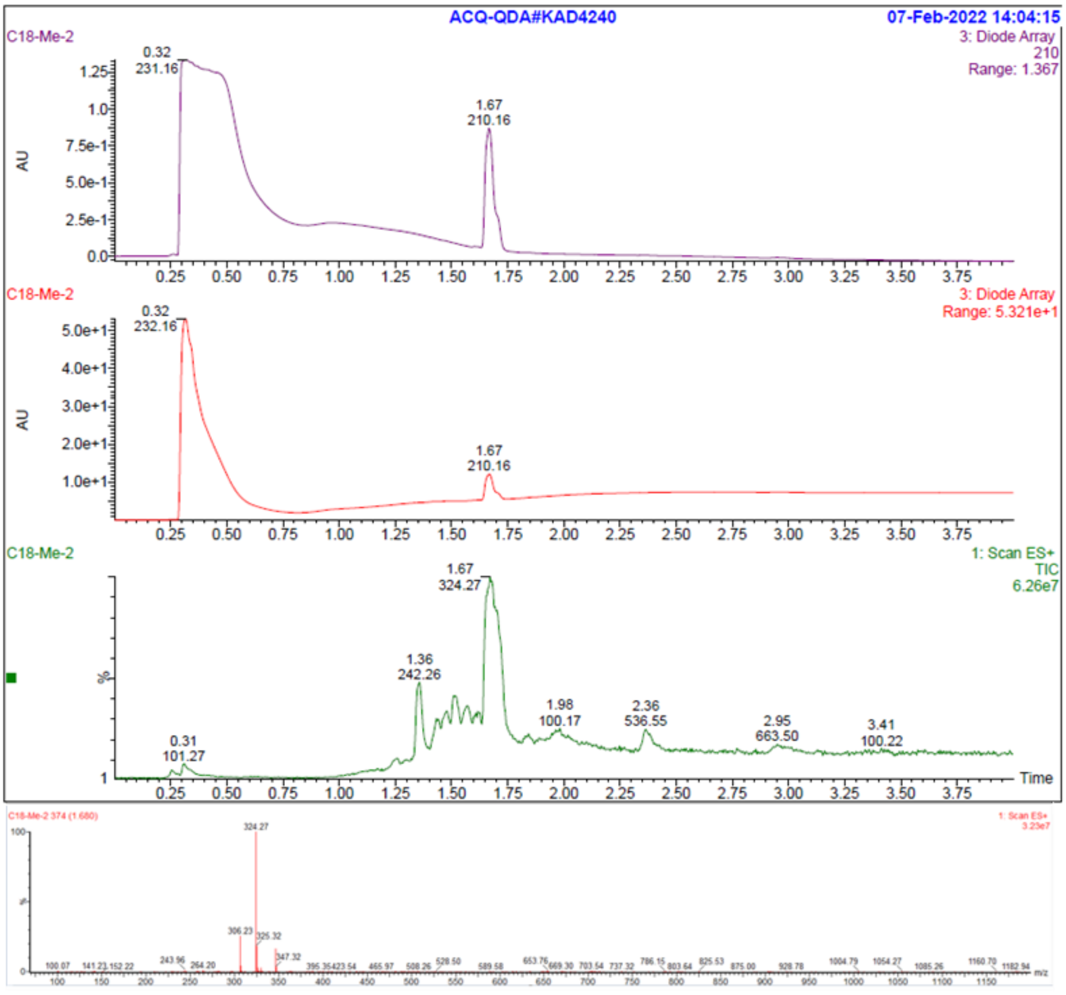

14-(N-(prop-2-yn-1-yl)cyclopropanecarboxamido)tetradecanoic acid (6e; 18-cPr)

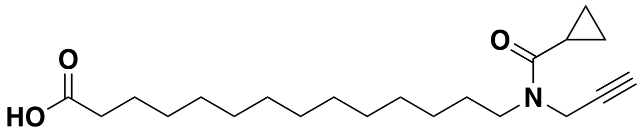

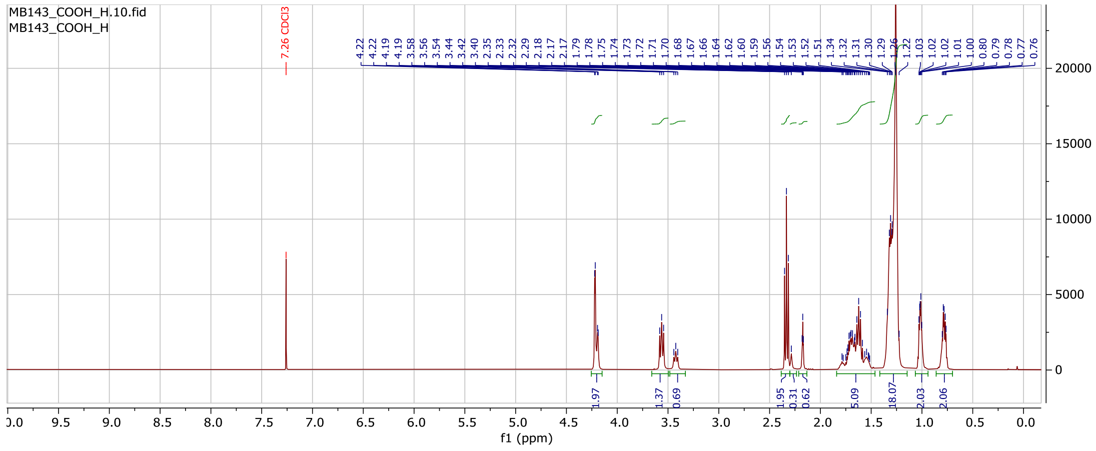
MB143_COOH_H.10.fid
MB143_COOH_H
7.26 CDCl3
4.22
4.22
4.19
4.19
3.58
3.56
3.54
3.44
3.42
3.40
2.35
2.33
2.32
2.29
2.18
2.17
2.17
1.79
1.78
1.75
1.74
1.73
1.72
1.71
1.70
1.68
1.67
1.66
1.64
1.62
1.60
1.59
1.56
1.54
1.53
1.53
1.52
1.51
1.34
1.32
1.31
1.30
1.29
1.26
1.22
1.03
1.02
1.02
1.01
1.00
0.80
0.79
0.78
0.77
0.76
1.97
1.37
0.69
1.95
0.31
0.62
5.09
18.07
2.03
2.06
f1 (ppm)

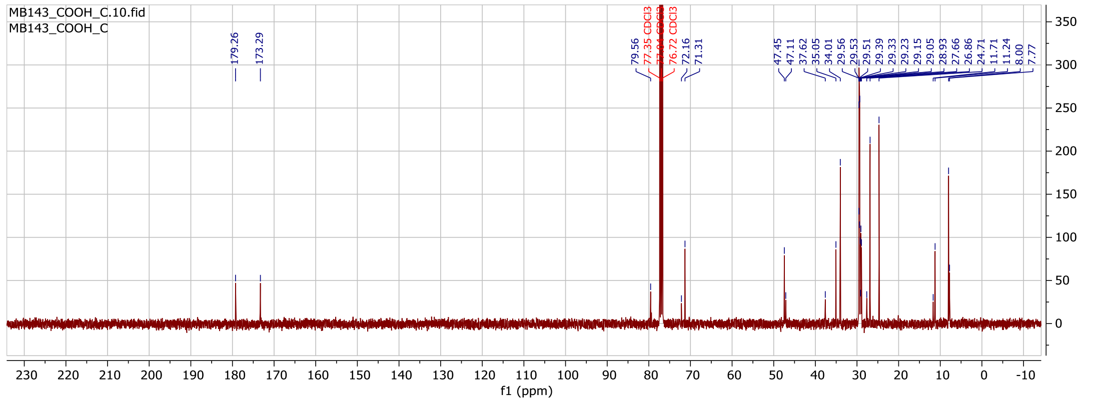
MB143_COOH_C.10.fid
MB143_COOH_C
179.26
173.29
79.56
77.35 CDCl3
76.72 CDCl3
72.16
71.31
47.45
47.11
37.62
35.05
34.01
29.56
29.53
29.51
29.39
29.33
29.23
29.15
29.05
28.93
27.66
26.86
24.71
11.71
11.24
8.00
7.77
f1 (ppm)

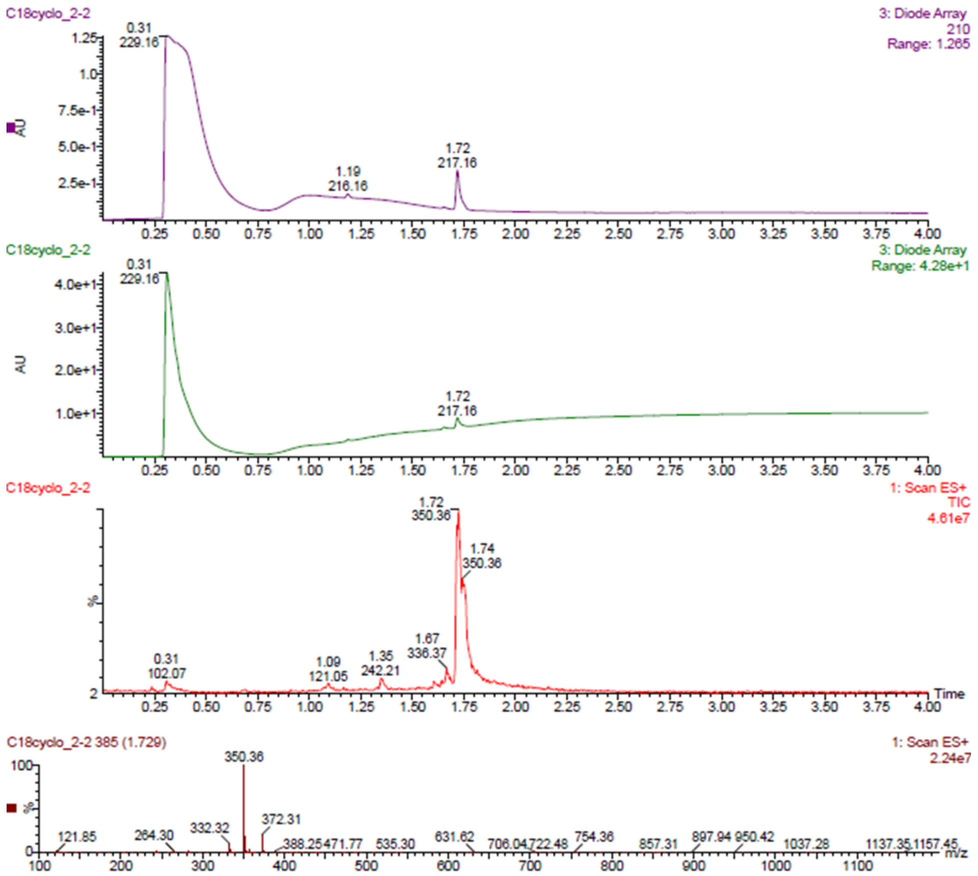

14-(N-(prop-2-yn-1-yl)benzamido)tetradecanoic acid (6f; 18-Bz)

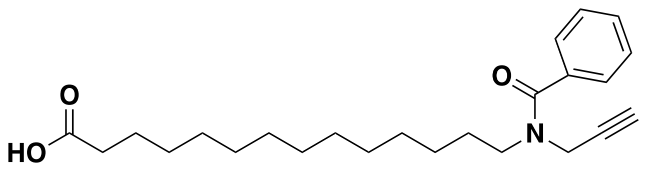

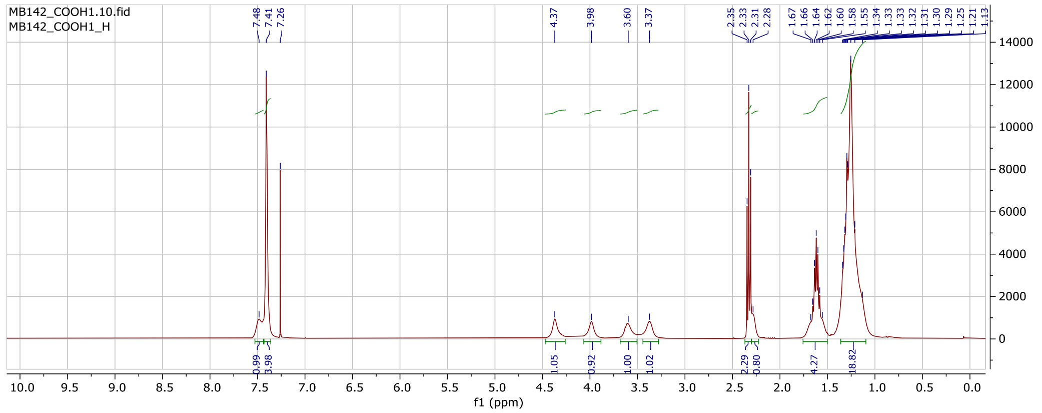
MB142_COOH1.10.fid
MB142_COOH1_H
f1 (ppm)

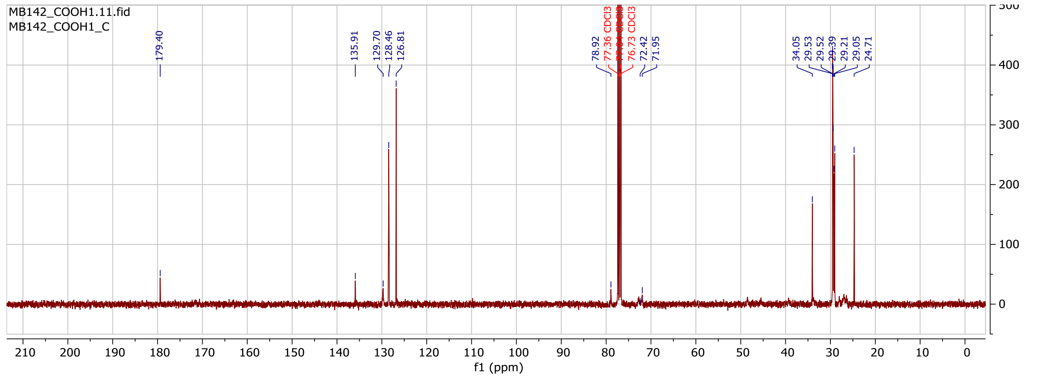
MB142_COOH1.11.fid
MB142_COOH1_C
179.40
135.91
129.70
128.46
126.81
78.92
77.36 CDCl3
76.73 CDCl3
72.42
71.95
34.05
29.53
29.52
29.39
29.21
29.05
24.71
f1 (ppm)

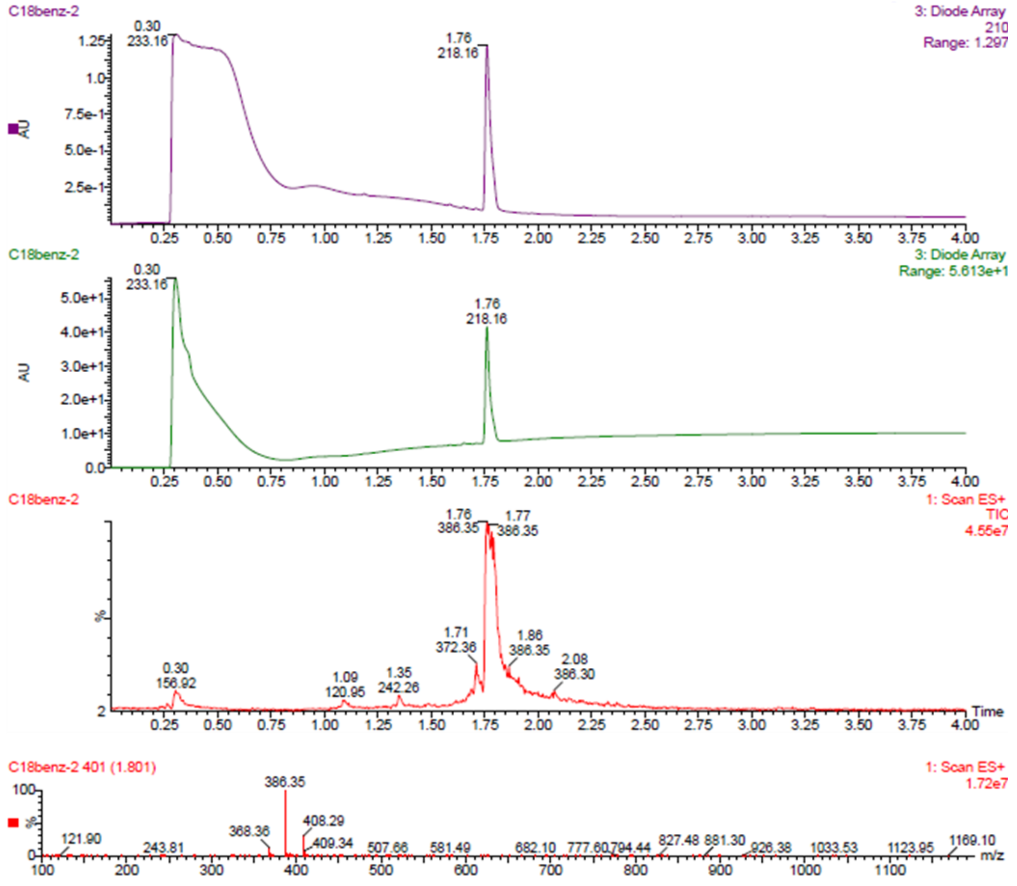
C18benz-2
3: Diode Array
210
Range: 1.297
C18benz-2
3: Diode Array
Range: 5.613e+1
C18benz-2
1: Scan ES+
TIC
4.55e7
C18benz-2 401 (1.801)
1: Scan ES+
1.72e7
386.35
408.29
368.36
409.34
m/z

16-(N-(prop-2-yn-1-yl)acetamido)hexadecanoic acid (6g; 20-Ac)

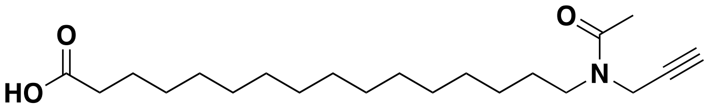

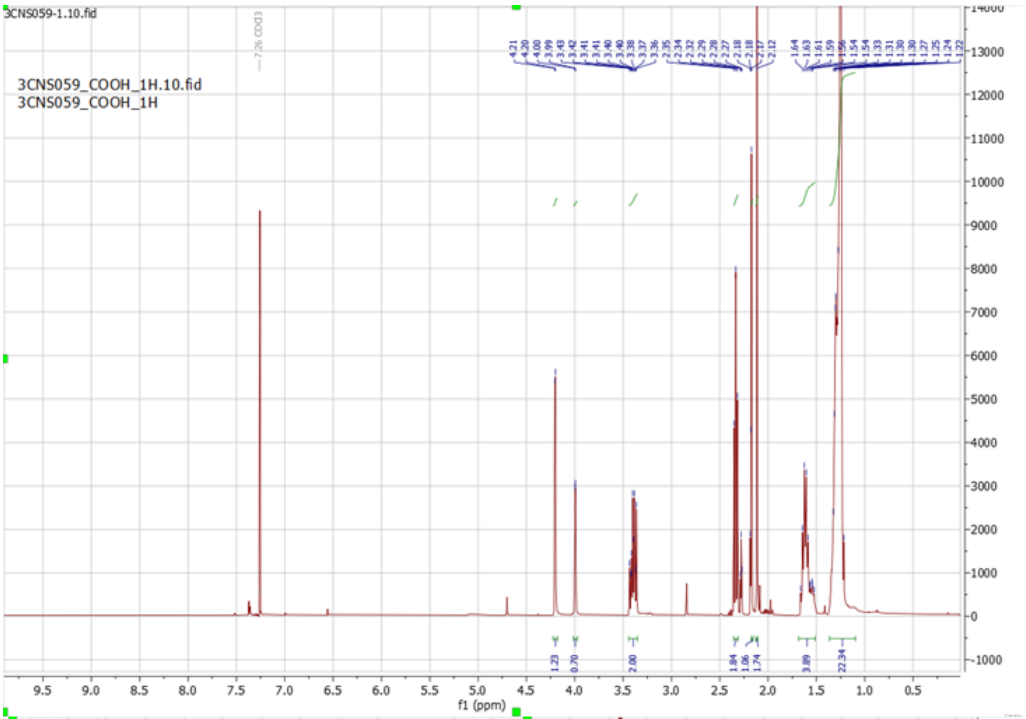

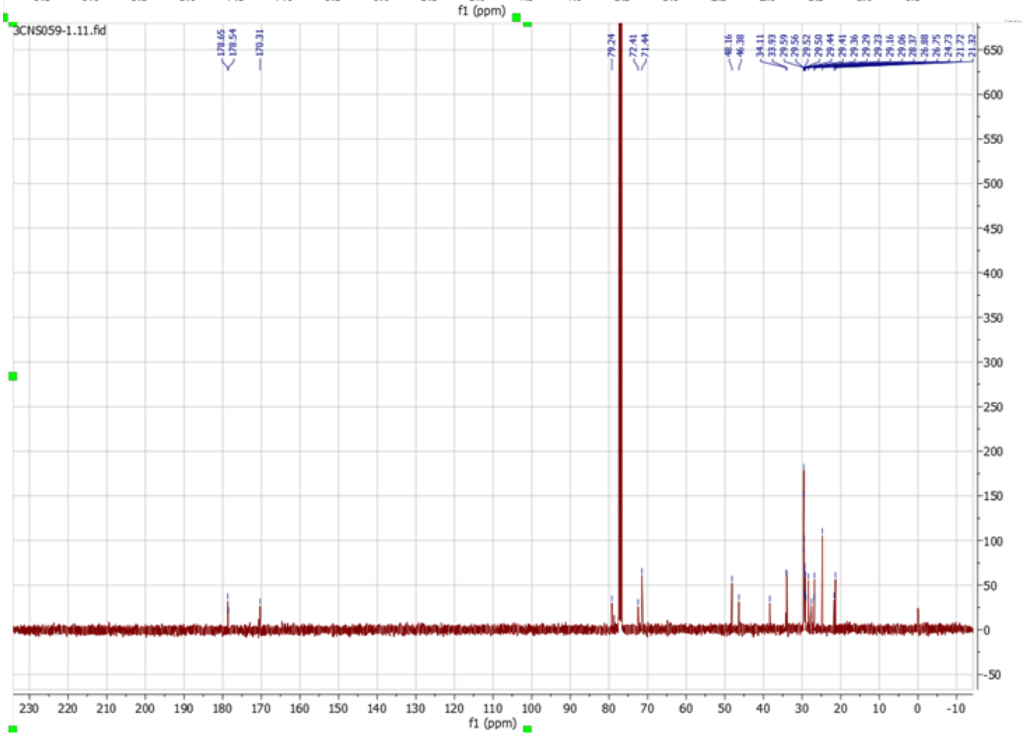

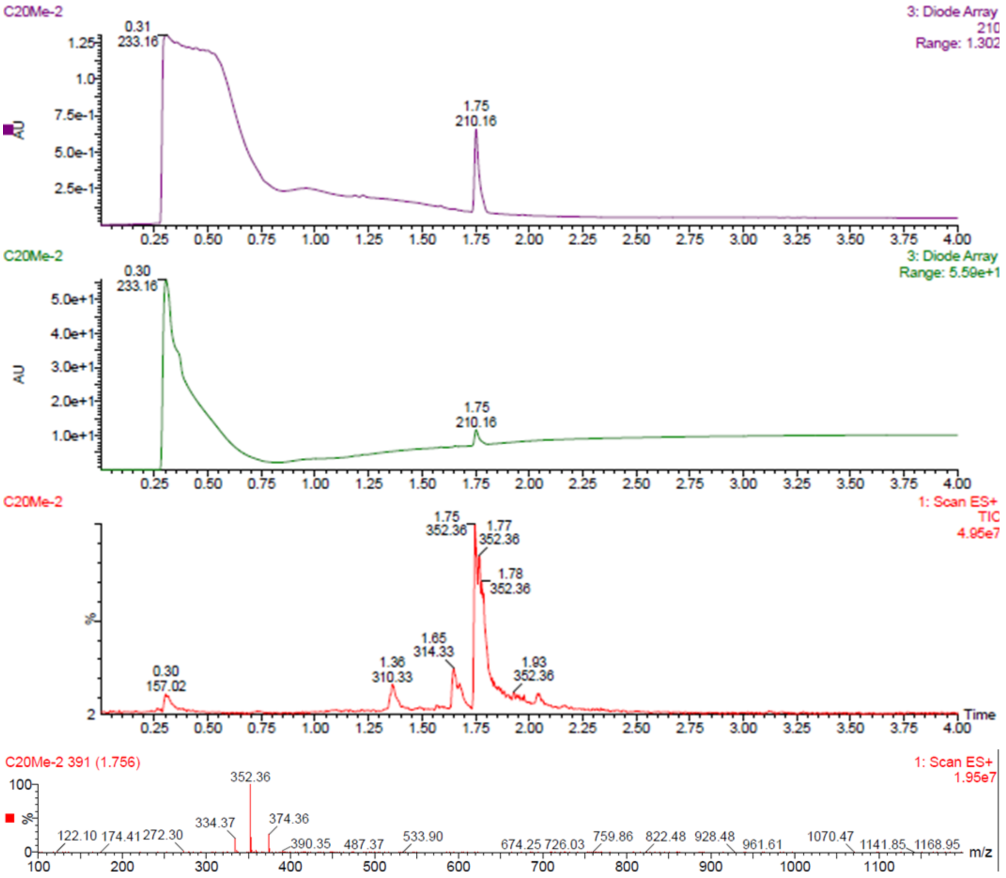

16-(N-(prop-2-yn-1-yl)cyclopropanecarboxamido)hexadecanoic acid (6h; 20-cPr)

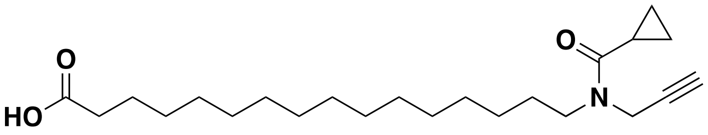

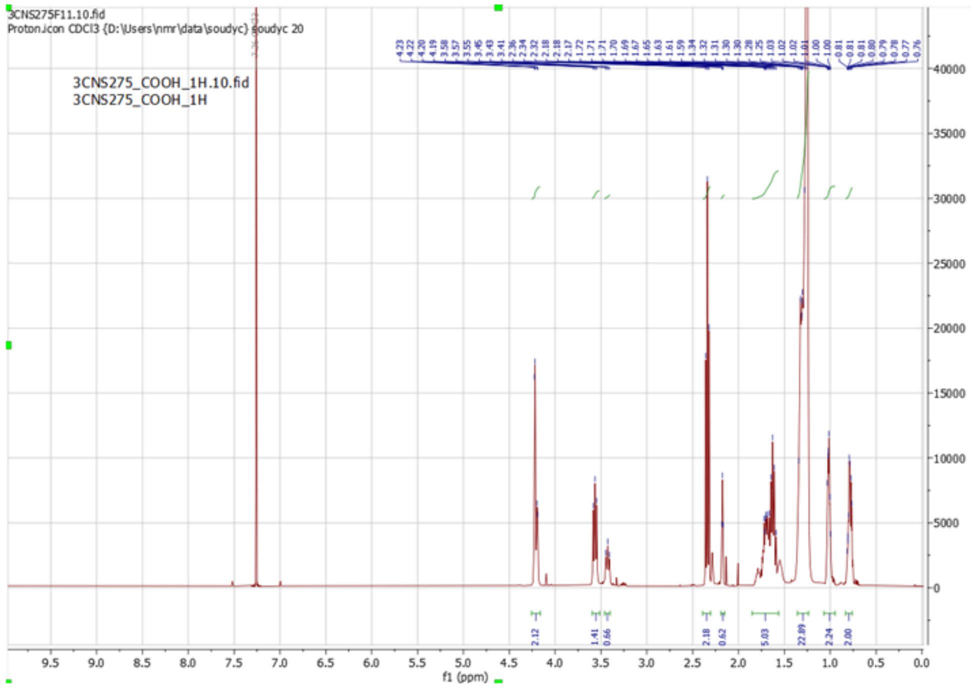

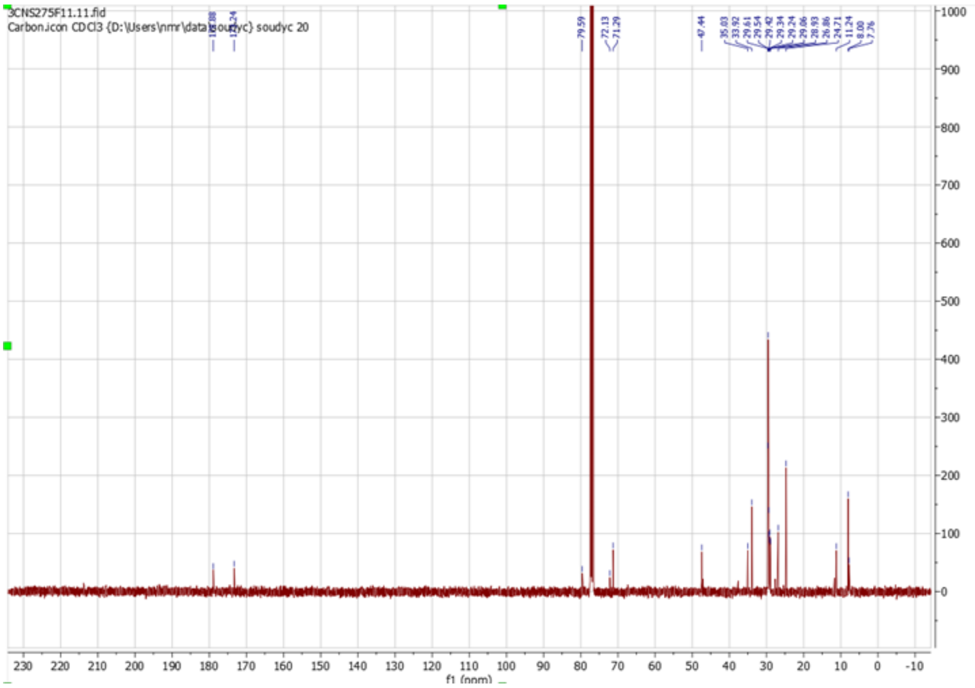

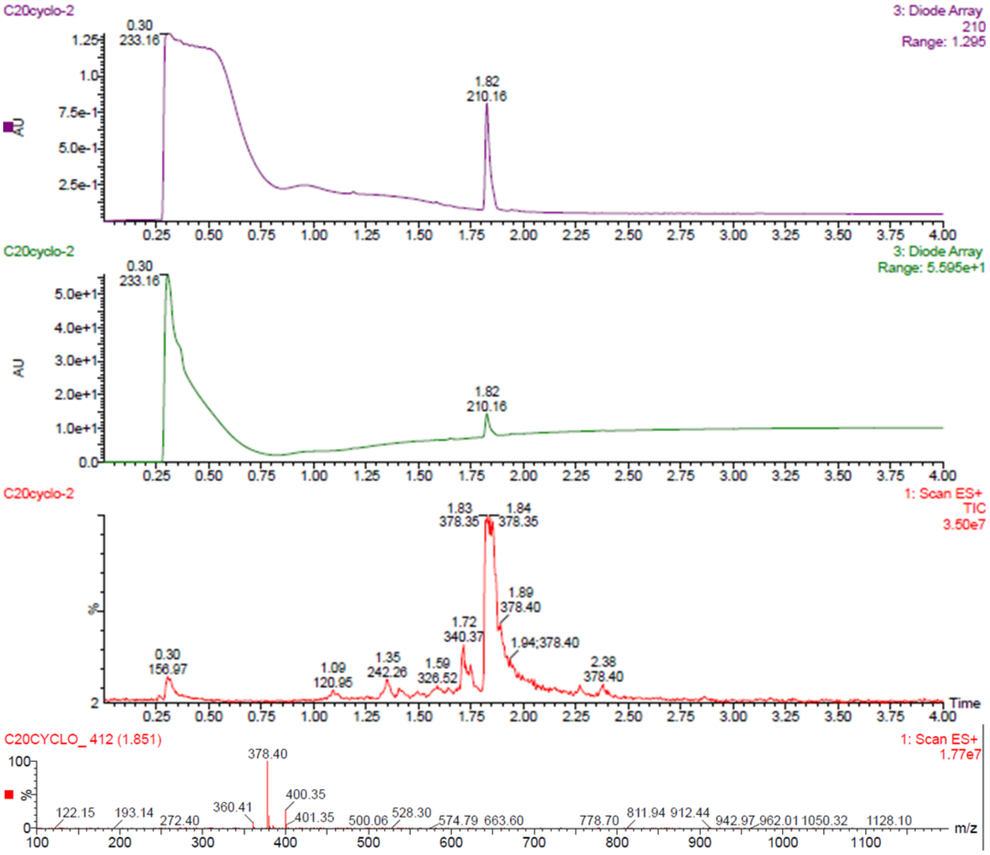

16-(N-(prop-2-yn-1-yl)benzamido)hexadecanoic acid (6i; 20-Bz)

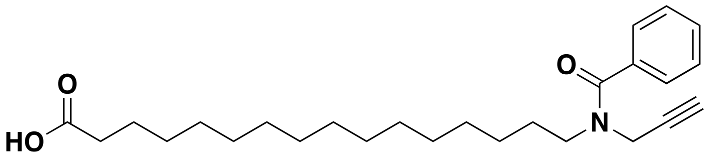
O
HO
O
N

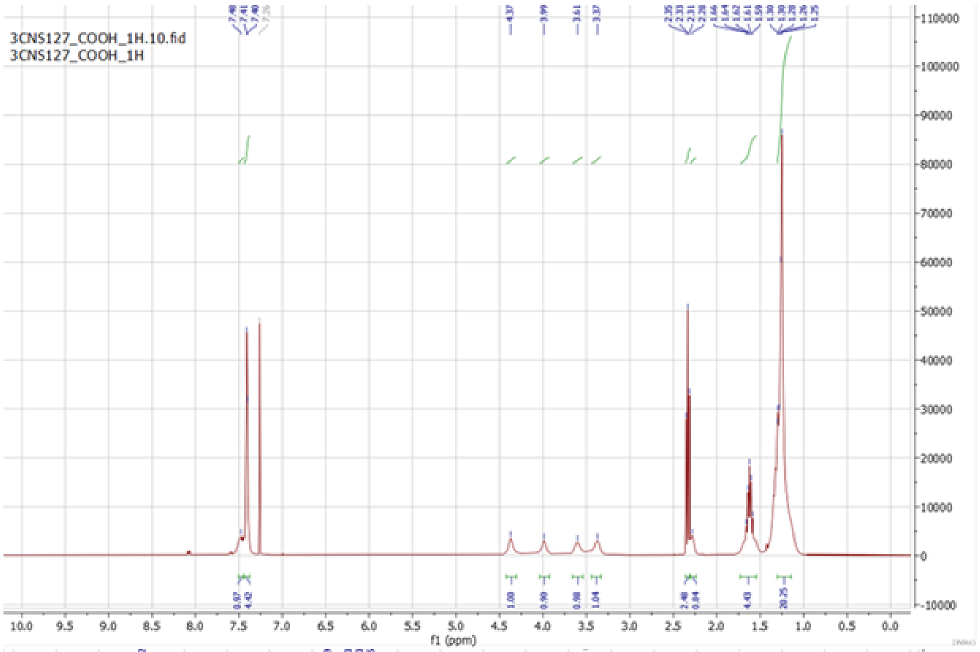
3CNS127_COOH_1H.10.fid
3CNS127_COOH_1H
f1 (ppm)

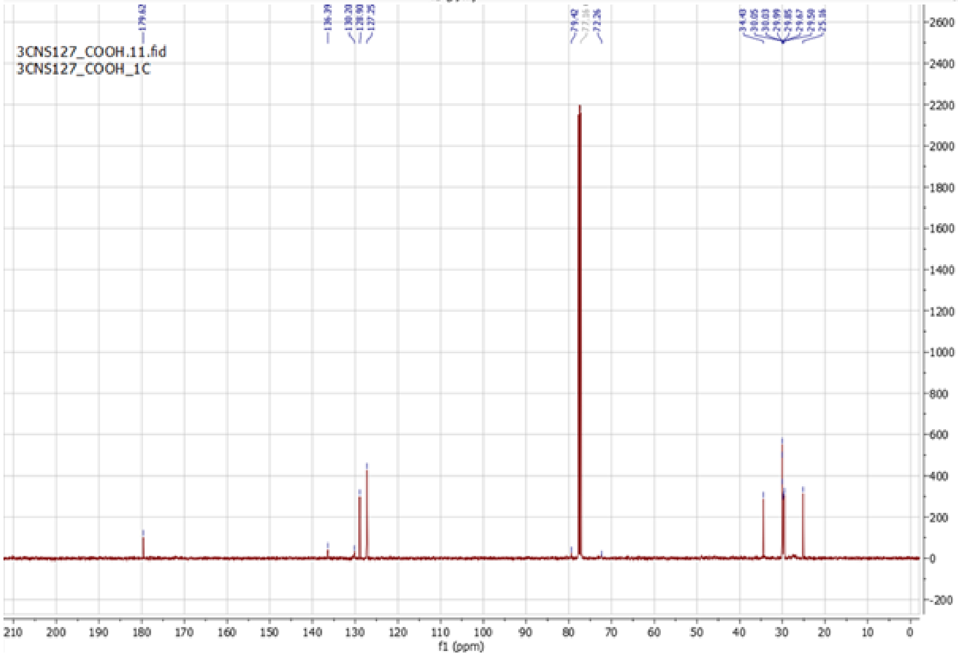
3CNS127_COOH.11.fid
3CNS127_COOH_1C
f1 (ppm)

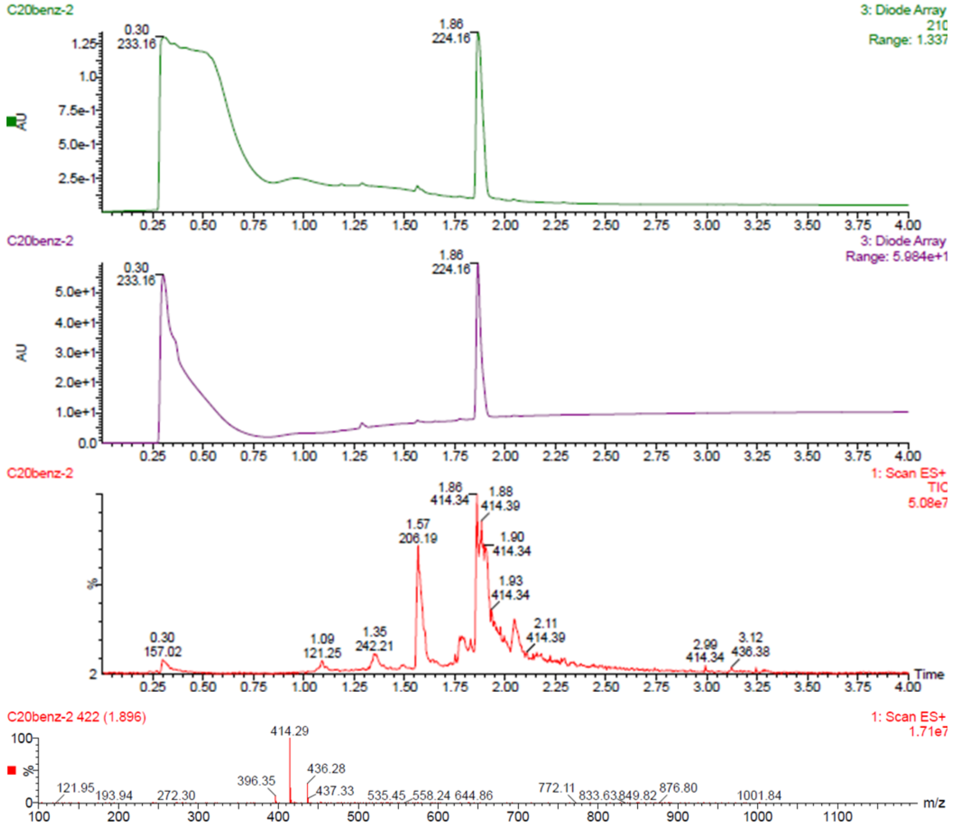

C18-Bz-CoA probe

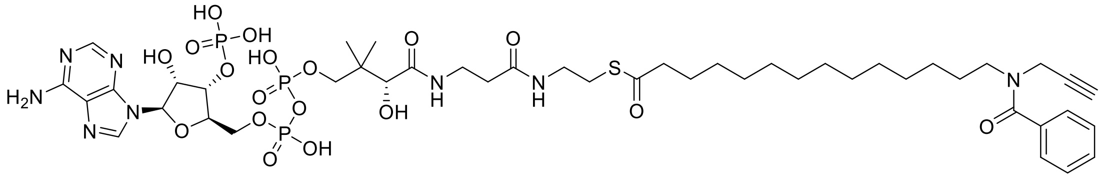

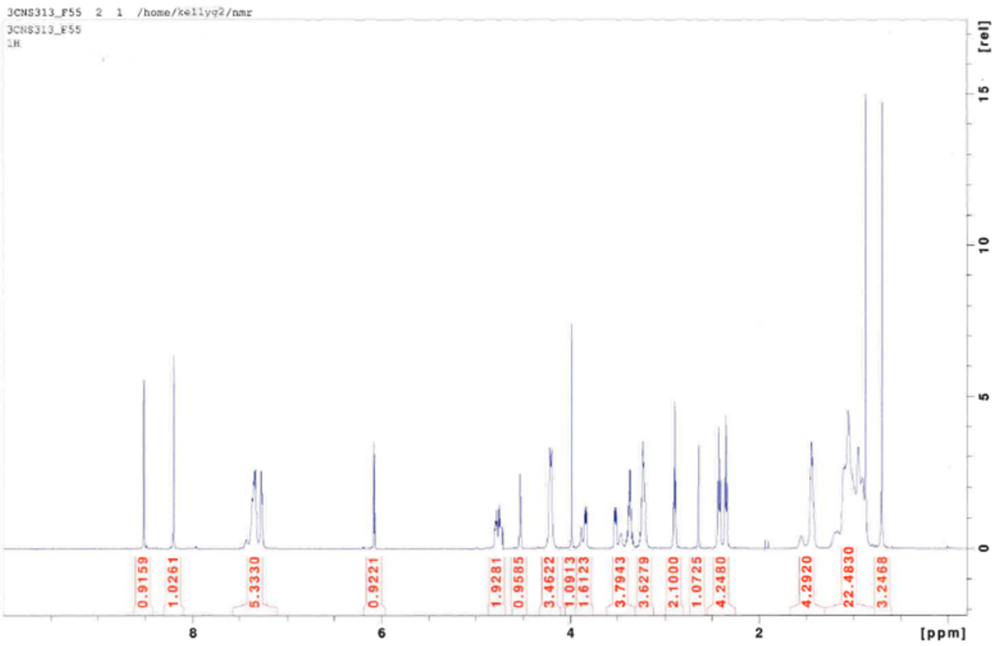

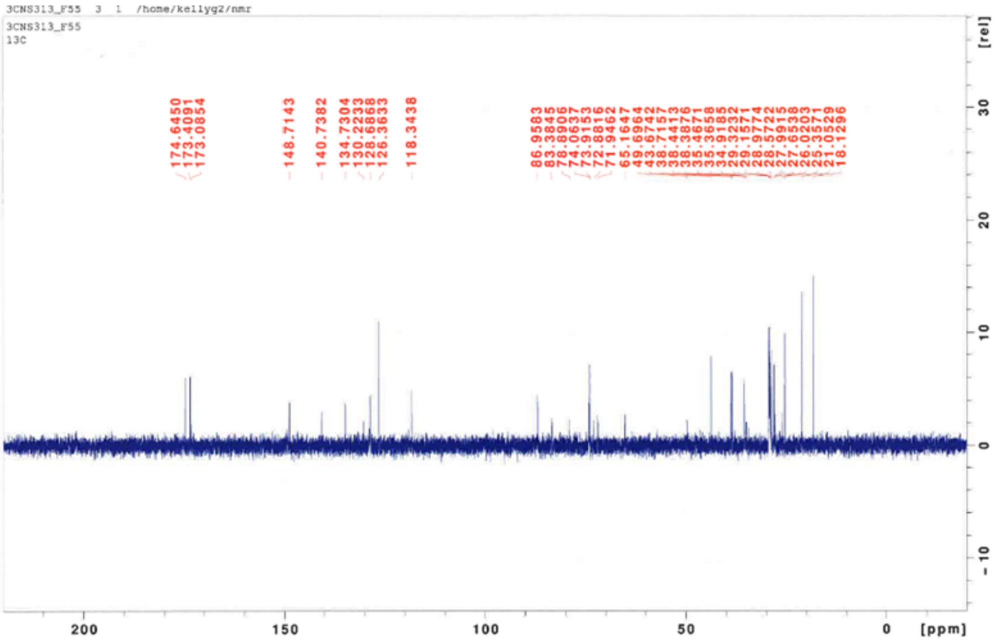
3CNS313_F55 3 1 /home/kellyg2/nmr
3CNS313_F55
13C
174.6450
173.4091
173.0854
148.7143
140.7382
134.7304
130.2233
128.6868
126.3633
118.3438
[rel]
[ppm]

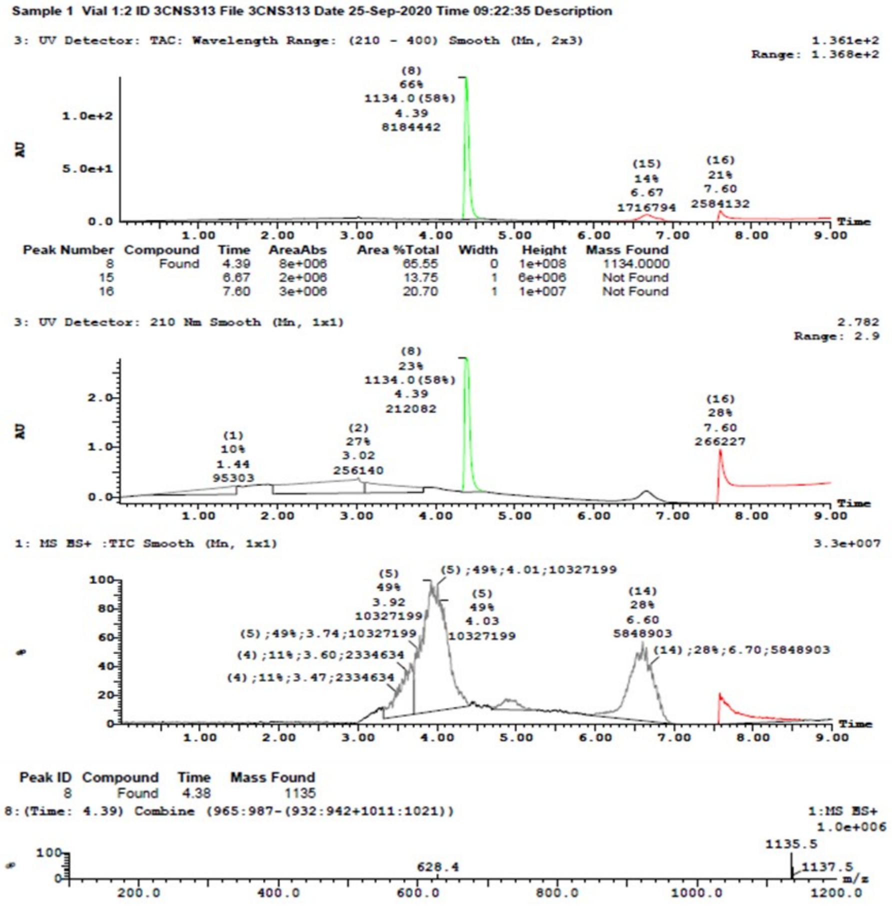
Sample 1 Vial 1:2 ID 3CNS313 File 3CNS313 Date 25-Sep-2020 Time 09:22:35 Description
3: UV Detector: TAC: Wavelength Range: (210 - 400) Smooth (Mn, 2x3)
1.361e+2
Range: 1.368e+2
(8)
66%
1134.0 (58%)
4.39
8184442
(15)
14%
6.67
1716794
(16)
21%
7.60
2584132
Peak Number Compound Time AreaAbs Area %Total Width Height Mass Found
8 Found 4.39 8e+006 65.55 0 1e+008 1134.0000
15 6.67 2e+006 13.75 1 6e+006 Not Found
16 7.60 3e+006 20.70 1 1e+007 Not Found
3: UV Detector: 210 Nm Smooth (Mn, 1x1)
2.782
Range: 2.9
(8)
23%
1134.0 (58%)
4.39
212082
(1)
10%
1.44
95303
(2)
27%
3.02
256140
(16)
28%
7.60
266227
1: MS ES+ :TIC Smooth (Mn, 1x1)
3.3e+007
(5);49%;4.01;10327199
(5)
49%
3.92
10327199
(5)
49%
4.03
10327199
(5);49%;3.74;10327199
(4);11%;3.60;2334634
(4);11%;3.47;2334634
(14)
28%
6.60
5848903
(14);28%;6.70;5848903
Peak ID Compound Time Mass Found
8 Found 4.38 1135
8: (Time: 4.39) Combine (965:987-(932:942+1011:1021))
1:MS ES+
1.0e+006
1135.5
628.4
1137.5
m/z

Supplementary Tables

Supplementary Table 1. Kinetic analysis of WT, Y181G, C156S and Y181G/C156S ZDHHC20 expression constructs with YnPal-CoA and 18-Bz-CoA.

	**WT-ZDHHC20**	
**Kinetic Parameters**	**Pal-CoA**	**C18-Bz-CoA**
Vmax (μM NADH•min^{-1})	0.45 ± 0.006	na
Kcat (min^{-1})	22.5 ± 0.3	na
Km (μM)	3.3 ± 0.2	na
Kcat/Km (μM•min)$^{-1}$	6.8 ± 0.3	na
	**YG-ZDHHC20**	
**Kinetic Parameters**	**Pal-CoA**	**C18-Bz-CoA**
Vmax (μM NADH•min^{-1})	0.45 ± 0.03	0.24 ± 0.01
Kcat (min^{-1})	22.3 ± 1.5	11.8 ± 0.6
Km (μM)	2.9 ± 0.1	0.74 ± 0.02
Kcat/Km (μM •min)$^{-1}$	7.6 ± 0.3	16.0 ± 1.0

Supplementary Table 2. List of vectors used in this study

**Plasmid**	**Associated Projects**	**Source**
ZDHHC20 (MYC-FLAG-tagged)-Human (CFlagD20)	ZDHHC Mammalian Expression	Origene Technologies #RC215317
N-3XFLAG-ZDHHC01	ZDHHC Mammalian Expression	Gifted by Dr Yusuke Ohno, Faculty of Pharmaceutical Sciences, Hokkaido University
N-3XFLAG-ZDHHC02	ZDHHC Mammalian Expression	Gifted by Dr Yusuke Ohno, Faculty of Pharmaceutical Sciences, Hokkaido University
N-3XFLAG-ZDHHC03	ZDHHC Mammalian Expression	Gifted by Dr Yusuke Ohno, Faculty of Pharmaceutical Sciences, Hokkaido University
N-3XFLAG-ZDHHC04	ZDHHC Mammalian Expression	Gifted by Dr Yusuke Ohno, Faculty of Pharmaceutical Sciences, Hokkaido University
N-3XFLAG-ZDHHC05	ZDHHC Mammalian Expression	Gifted by Dr Yusuke Ohno, Faculty of Pharmaceutical Sciences, Hokkaido University
N-3XFLAG-ZDHHC06	ZDHHC Mammalian Expression	Gifted by Dr Yusuke Ohno, Faculty of Pharmaceutical Sciences, Hokkaido University
N-3XFLAG-ZDHHC07	ZDHHC Mammalian Expression	Gifted by Dr Yusuke Ohno, Faculty of Pharmaceutical Sciences, Hokkaido University
N-3XFLAG-ZDHHC08	ZDHHC Mammalian Expression	Gifted by Dr Yusuke Ohno, Faculty of Pharmaceutical Sciences, Hokkaido University
N-3XFLAG-ZDHHC09	ZDHHC Mammalian Expression	Gifted by Dr Yusuke Ohno, Faculty of Pharmaceutical Sciences, Hokkaido University
N-HA-GCP16 (GOLGA7)	GCP16 Mammalian Expression	Gifted by Dr Yusuke Ohno, Faculty of Pharmaceutical Sciences, Hokkaido University

N-3XFLAG-ZDHHC11	ZDHHC Mammalian Expression	Gifted by Dr Yusuke Ohno, Faculty of Pharmaceutical Sciences, Hokkaido University
N-3XFLAG-ZDHHC12	ZDHHC Mammalian Expression	Gifted by Dr Yusuke Ohno, Faculty of Pharmaceutical Sciences, Hokkaido University
N-3XFLAG-ZDHHC13	ZDHHC Mammalian Expression	Gifted by Dr Yusuke Ohno, Faculty of Pharmaceutical Sciences, Hokkaido University
N-3XFLAG-ZDHHC14	ZDHHC Mammalian Expression	Gifted by Dr Yusuke Ohno, Faculty of Pharmaceutical Sciences, Hokkaido University
C-3XFLAG-ZDHHC15	ZDHHC Mammalian Expression	Gifted by Dr Yusuke Ohno, Faculty of Pharmaceutical Sciences, Hokkaido University
N-3XFLAG-ZDHHC16	ZDHHC Mammalian Expression	Gifted by Dr Yusuke Ohno, Faculty of Pharmaceutical Sciences, Hokkaido University
N-3XFLAG-ZDHHC17	ZDHHC Mammalian Expression	Gifted by Dr Yusuke Ohno, Faculty of Pharmaceutical Sciences, Hokkaido University
N-3XLAG-ZDHHC18	ZDHHC Mammalian Expression	Gifted by Dr Yusuke Ohno, Faculty of Pharmaceutical Sciences, Hokkaido University
N-3XFLAG-ZDHHC19	ZDHHC Mammalian Expression	Gifted by Dr Yusuke Ohno, Faculty of Pharmaceutical Sciences, Hokkaido University
N-3XFLAG-ZDHHC21	ZDHHC Mammalian Expression	Gifted by Dr Yusuke Ohno, Faculty of Pharmaceutical Sciences, Hokkaido University
C-3XFLAG-ZDHHC22	ZDHHC Mammalian Expression	Gifted by Dr Yusuke Ohno, Faculty of Pharmaceutical Sciences, Hokkaido University
N-3XFLAG-ZDHHC23	ZDHHC Mammalian Expression	Gifted by Dr Yusuke Ohno, Faculty of Pharmaceutical Sciences, Hokkaido University
N-3XFLAG-ZDHHC24	ZDHHC Mammalian Expression	Gifted by Dr Yusuke Ohno, Faculty of Pharmaceutical Sciences, Hokkaido University
3XFLAG Vector (EF-1α)	Negative Control for ZDHHC Expression	Gifted by Dr Yusuke Ohno, Faculty of Pharmaceutical Sciences, Hokkaido University
C-FLAG-pcDNA3	Negative Control for ZDHHC Expression	Addgene #20011
V5-Turbo-NES-pCDNA3	Proximity Based Labelling, BirA mutant	BirA mutant, promiscuous biotin ligase, Addgene #107169
pEGFP-N1-FLAG	GFP Fluorescence	Addgene #60360
Attb-ZDHHC20-BSDr-Vector (Mus Musculus)	Jump-In, Stable Targeted Integration of cDNA	Gifted by the Downward lab (The Francis Crick Institute), Generated In-House
pCMV-Int (PhiC31)	Jump-In, Stable Targeted Integration of cDNA	Gifted by the Downward lab (The Francis Crick Institute), Generated In-House, Addgene #18935
pLVX-TetOne-Puro Vector	Lentiviral Transduction	Gifted by the Downward lab (The Francis Crick Institute), Generated In-House, Clontech #631849
pCMV-VSV-G	Lentiviral Production	Gifted by the Downward lab (The Francis Crick Institute), Generated In-House, Addgene #8454
pCMV-Delta-8.2	Lentiviral Packaging	Gifted by the Downward lab (The Francis Crick Institute), Generated In-House, Addgene #12263
pcDNA3.1(+)-HA-Ifitm3 (Mus Musculus)	Iftim3 Mammalian Expression	Gifted by Dr Emmanuelle Thinon (Centre National de la Recherche Scientifique)
pcDNA3.1(+)-HA-VAMP3	VAMP3 Mammalian Expression	Gifted by Dr Emmanuelle Thinon (Centre National de la Recherche Scientifique)

pcDNA3.1(+)-HA-VAMP3 (C76A)	VAMP3 Mammalian Expression	Gifted by Dr Emmanuelle Thinon (Centre National de la Recherche Scientifique)
pcDNA3.1(+)-XXYLT1-HA	XXYLT1 Mammalian Expression	Gifted by Dr Hans Bakker(Hannover Medical School)
pSpCas9(BB)-2A-Puro (PX459) V2.0	Production of Gene Knock-Out Cells	Addgene #62988

Supplementary Table 3. Primer sets used to generate PCR fragments for NEBuilder HiFi Assembly reactions.

Plasmid	Forward Primer (5' → 3')	Reverse Primer (5' → 3')	Backbone
C-HA-D20	AGGAGATCTGCCGCCGCGA TATGGCGCCCTGGACGCTG	ATGACCGCGGCCGGCCGTT TTTAACCAGAACCTGA**AGCG TAATCTGGAACATCGTATGG GTA**CAGGATATCATTTGCTG CCAGATCCTCTTC	C-FLAG-D20
pLVX-C-FLAG-D20	CTCGCAGGGGAGGTGGTCT GTTAAACCTTATCGTCGTCAT CCTTGTAATCCAGG	CACTTCCTACCCTCGTAAAG ATGGCGCCCTGGACGCTG	pLVX-TetOne-Puro
attb-C-FLAG-D20-BSDr	CTCATCTCCGGGCCTTTCGC ATGGCGCCCTGGACGCTG	TGGCAACTAGAAGGCACAGC TTAAACCTTATCGTCGTCATC CTTGTAATCCAGG	attb-ZDHHC20-BSDr
pcDNA3.1(+)-HA-BCAP31	TCCAGATTACGCTCTTATGG GGATGAGTCTGCAGTGGACT G	GGTACCTCGAGAGATCTCGG TTACTCTTCCTTCTTGTCCAT G	pcDNA3.1(+)-HA-Ifitm3
N-HA-PI4K2A	CTTATGGCCATGGAGGCCC GAATTCCCATGGACGAGAC	ATCCCCGCGGCCGCGGTAC CCTACCACCATGAAAAGAAG	pDONR223-PI4K2A Addgene #23503
N-HA-TOMM20	CTTATGGCCATGGAGGCCC GAATTCCCATGGTGGGTCG	ATCCCCGCGGCCGCGGTAC CTTATTCCACATCATCTTCAG CC	mCherry-TOMM20-N-10 Addgene #55146
N-HA-TFAM	CTTATGGCCATGGAGGCCC GAATTCCCATGGCGTTTCTC	ATCCCCGCGGCCGCGGTAC CTTAACACTCCTCAGCACC	pcDNA3-TFAM-mCLOVER Addgene #129574

Supplementary Table 4. Primer sets used to generate Turbo, EGFP and ZDHHC20 PCR fragments for NEBuilder HiFi Assembly reactions.

Plasmid	Forward Primer (5' → 3')	Reverse Primer (5' → 3')	Backbone
attb-N-Turbo-EGFP-BSDr	CTCATCTCCGGGCCTTTCGC A TGGGCAAGCCCATCCCC	CCTTGCTCACGTCCAGGGTC AGGCGCTC	V5-Turbo-NES-pCDNA3
	GACCCTGGACGTGAGCAAG GGCGAGGAG	TGGCAACTAGAAGGCACAGC TTACTTGTACAGCTCGTCCAT G	pEGFP-N1-FLAG
attb-N-D20-Turbo-BSDr	CTCATCTCCGGGCCTTTCGC ATGGCGCCCTGGACGCTG	TGGGCTTGCCGAGCGGCCG CGTACGCGT	C-FLAG-D20
	GCGGCCGCTCGGCAAGCCC ATCCCCAAC	TGGCAACTAGAAGGCACAGC TTAGTCCAGGGTCAGGCG	V5-Turbo-NES-pCDNA3
attb-N-Turbo-D20-BSDr	CTCATCTCCGGGCCTTTCGC ATGGGCAAGCCCATCCCC	AGGGCGCCATGTCCAGGGT CAGGCGCTC	V5-Turbo-NES-pCDNA3
	GACCCTGGACATGGCGCCC TGGACGCTG	TGGCAACTAGAAGGCACAGC TTAAACCTTATCGTCGTCATC CTTGTAATCCAGG	C-FLAG-D20
pcDNA5 FRT/ ZDHHC20	TAAGCTTGGTACCGAGCTCG CATGGCGCCCTGGACGCT	CGGGCCCTCTAGACTCGAG CTTTAAACCTTATCGTCGTCA TCCTTGTAATCCAGG	TREX-ZDHHC20 WT & ZDHHC20[Y181G]

Supplementary Table 5. Primer sets used for ZDHHC mutagenesis.

Mutant	Forward Primer (5' → 3')	Reverse Primer (5' → 3')	Templates
C156S	TCTTAAGATGGATCATCACAGT CCTTGGGTGAATAACTG	CAGTTATTCACCCAAGGACTGTGAT GATCCATCTTAAGA	C-Flag-D20, C-HA-D20, pLVX-C-Flag-D20
Y181G	CCTGCTGTTTTTATTGTATTCCC TATTAGGTTGCCTTTTCGTGGC TG	CAGCCACGAAAAGGCAACCTAATA GGGAATACAATAAAAACAGCAGG	C-Flag-D20, pLVX-C-Flag-D20
Y181A	CCTGCTGTTTTTATTGTATTCCC TATTAGCTTGCCTTTTCGTGGC TG	CAGCCACGAAAAGGCAAGCTAATA GGGAATACAATAAAAACAGCAGG	C-Flag-D20
I22A	GTGCCGGTGCTCTTCGCCACCT TCGTGGTCGT	ACGACCACGAAGGTGGCGAAGAG CACCGGCAC	C-Flag-D20
F65A	GCTTTCCATCTGTTCTTTGTTAT GGCTGTATGGTCCTATTGGATG ACAAT	ATTGTCATCCAATAGGACCATACAG CCATAACAAAGAACAGATGGAAAG C	C-Flag-D20
F174A	GGATTTTCTAATTACAAATTCTT CCTGCTGGCTTTATTGTATTCC CTATTATATTGCCTTTT	AAAAGGCAATATAATAGGGAATACA ATAAAGCCAGCAGGAAGAATTTGTA ATTAGAAAATCC	C-Flag-D20
F220A	TTTTCTTTGTGTCTGCAATGGC CTTCATCAGCGTCCTCTCAC	GTGAGAGGACGCTGATGAAGGCCA TTGCAGACACAAAGAAAA	C-Flag-D20
L193G	GGGCGTCCTGCTCGGGGTGCT GGTGGCC	GGCCACCAGCACCCCGAGCAGGA CGCCC	N-Flag-D01
Y182G	CTTGGCTTATTCTCTGCTCGGC TGCCTTTTTATTGCGGCA	TGCCGCAATAAAAAGGCAGCCGAG CAGAGAATAAGCCAAG	N-Flag-D02
I182G	TACAATGTACATAGCTCTCGGT TCCTTGCACGCCCTCATC	GATGAGGGCGTGCAAGGAACCGA GAGCTATGTACATTGTA	N-Flag-D03
L108G	CTTCTTCTGCCCTATCTGCTGG GAGGTGTAAACCTGTTTTTTTT	AAAAAAAACAGGTTTACACCTCCCA GCAGATAGGGCAGAAGAAG	N-Flag-D04
H159G	CTCCTTTCCCTGACAGCCGGCA TTATGGGTGTGTTTGG	CCAAACACACCCATAATGCCGGCT GTCAGGGAAAGGAG	N-Flag-D05
I168G	CATCATGTTGATAAATTGGACT GTCATGGGTCTTTATAATTACTT CAATGCCATGTTT	AAACATGGCATTGAAGTAATTATAA AGACCCATGACAGTCCAATTTATCA ACATGATG	N-Flag-D06
L57G	CTGTCATGACGTGGCTTGGGGT CGCCTATGCAGA	TCTGCATAGGCGACCCCAAGCCAC GTCATGACAG	N-Flag-D07
M160G	CACTCAGTGCACACGGGGTGG GCGTCGTGG	CCACGACGCCCACCCCGTGTGCAC TGAGTG	N-Flag-D08
L194G	CATCCTTTCTCTCTCCCTCGGC ACAATCTATGTCTTCGCC	GGCGAAGACATAGATTGTGCCGAG GGAGAGAGAAAGGATG	N-Flag-D09
M181G	CAGCACTGTGGCCTCGGCCAC AGCTGGCGGGCTCTGCC	CATACAGCAGGATGGCGATCAGGC AGAGCCCGCCAGCTG	N-Flag-D11
M181A	CGGCCACAGCTGGCGCGCTCT GCCTGATCG	CGATCAGGCAGAGCGCGCCAGCT GTGGCCG	N-Flag-D11
L153G	CTACCTGGCGCTGCAGCTGGT GGTGGGTCTGTGGGGCCTGTA C	GTACAGGCCCCACAGACCCACCAC CAGCTGCAGCGCCAGGTAG	N-Flag-D12
L478G	CTATTACATATTCTTCTTGTTTTT CGGTTCCATGGTATGTGGCTGG ATTATA	TATAATCCAGCCACATACCATGGAA CCGAAAAACAAGAAGAATATGTAAT AG	N-Flag-D13
L220G	AACTACAGATTTTTTTATATGTT TATTTTATCTCTGTCTTTTGGGA CAGTCTTTATATTTGCATTC	GAATGCAAATATAAAGACTGTCCCA AAAGACAGAGATAAAATAAACATAT AAAAAAATCTGTAGTT	N-Flag-D14
Y184G	CTTAGCTTACTCTGTTCTCGGC TGCCTGTACATTGCTACG	CGTAGCAATGTACAGGCAGCCGAG AACAGAGTAAGCTAAG	N-Flag-D15
C159S	TGTTAAAAATGGATCATCACAG CCCTTGGGTTAATAACTGC	GCAGTTATTAACCCAAGGGCTGTG ATGATCCATTTTTAACA	N-Flag-D15
S35C	CGTCGTGCTCTGGTGCTACTAT GCCTACG	CGTAGGCATAGTAGCACCAGAGCA CGACG	N-Flag-D15
L126G	TTCTTCTATAGCCACTGGAATG GGATCCTGATTGTCTTCCAC	GTGGAAGACAATCAGGATCCCATT CCAGTGGCTATAGAAGAA	N-Flag-D16
M492G	TATTTTATGGGCTACCTATTCTT CTTGCTTTTTGGGATCTGCTGG ATGATTTA	TAAATCATCCAGCAGATCCCAAAAA GCAAGAAGAATAGGTAGCCCATAA AATA	N-Flag-D17
L247G	CTTCTTCTACGCGTTTATTCTCT CCCTCTCATTCGGGACGGCCTT C	GTGACCACACAGGCGAAGATGAAG GCCGTCCCGAATGAGAG	N-Flag-D18
Y167G	GTCCCTGTGCCTCGGCTCGGG CGCCATG	CATGGCGCCCGAGCCGAGGCACA GGGAC	N-Flag-D19

L145G	CTGCAGTTGTGTTTCTACACTG AACTTGGTACTTGCTACGCAC	CAGAAAGAAAACATCAGTGCGTAG CAAGTACCAAGTTCAG	N-Flag-D21
L49G	CGCCCGCCCTGGGCCACGGGG CGC	GCGCCCCGTGGCCCAGGGCGGGC G	N-Flag-D22
I181G	TACCTGCGGGTTATTTCTGGGA CTCTTAGCCTTGCACAGA	TCTGTGCAAGGCTAAGAGTCCCAG AAATAACCCGCAGGTA	N-Flag-D23
L150G	GCCGCCGGCGTCGGGCTCCAC GTCTC	GAGACGTGGAGCCCGACGCCGGC GGC	N-Flag-D24
TREX Kozak	GTACCGAGCTCGCGCCGCCACCATGGC GCCCTGGA	TCCAGGGCGCCATGGTGGCGGCGCGAGCTC GGTAC	TREX-ZDHHC20 WT & ZDHHC20[Y181G]

Supplementary Table 6. Primer sets used for ZDHHC substrate mutagenesis

Mutant	Forward Primer (5' → 3')	Reverse Primer (5' → 3')	Templates
C71A	GCTATGAAGCCCAGGCAGGCG AAGTTCATGAAGAGTGT	ACACTCTTCATGAACTTCGCCTGCC TGGGCTTCATAGC	N-HA-Ifitm3
C72A	TAGGCTATGAAGCCCAGGGCG CAGAAGTTCATGAAGAG	CTCTTCATGAACTTCTGCGCCCTG GGCTTCATAGCCTA	N-HA-Ifitm3
C105A	GGTGCTGATGTTCAGGGCCTTA GCAGTGGAGGCG	CGCCTCCACTGCTAAGGCCCTGAA CATCAGCACC	N-HA-Ifitm3
C71/72A	GGCATAGGCTATGAAGCCCAG GGCGGCGAAGTTCATGAAGAG TGTATTG	CAATACACTCTTCATGAACTTCGCC GCCCTGGGCTTCATAGCCTATGCC	N-HA-Ifitm3
C71/72/105A	GGCATAGGCTATGAAGCCCAG GGCGGCGAAGTTCATGAAGAG TGTATTG	CAATACACTCTTCATGAACTTCGCC GCCCTGGGCTTCATAGCCTATGCC	N-HA-Ifitm3 C105A
C174/175A	GGCCAAAGCAGCAAGGAGCGG CCAGCTTCTGCAGCCACT	AGTGGCTGCAGAAGCTGGCCGCTC CTTGCTGCTTTGGCC	N-HA-PI4K2A
C177/178A	GGCAGTCACGGCCAAAGGCGG CAGGACAGCACAGCTTCT	AGAAGCTGTGCTGTCCTGCCGCCT TTGGCCGTGACTGCC	N-HA-PI4K2A
C174/175/177/ 178A	GACAAGGCAGTCACGGCCAAA GGCGGCAGGAGCGGCCAGCTT CTGCAGCCACTTGGTC	GACCAAGTGGCTGCAGAAGCTGGC CGCTCCTGCCGCCTTTGGCCGTGA CTGCCTTGTC	N-HA-PI4K2A
C23A	GTCTTTGTTGTGTTGCTTCTCG CCATTCCCTTCATTTCTCCTAA	TTAGGAGAAATGAAGGGAATGGCG AGAAGCAACACAACAAAGAC	pcDNA3.1(+)-HA-BCAP31

Supplementary Table S7. MS parameters were optimized by direct infusion of 16 μM acyl-CoAs dissolved in 10 mM MeOH/ammonium acetate at 5 μL/min into an TSQ Quantiva triple quadrupole MS (Thermo Scientific). A selected reaction monitoring (SRM) function was applied for the simultaneous detection of acyl-CoA and probe-CoA molecules with RF lens and collision energies as shown in the table below.

**Compound**	**Start Time (min)**	**End Time (min)**	**Polarity**	**Precursor (*m/z*)**	**Product (*m/z*)**	**Collision Energy (V)**	**RF Lens (V)**
$^{13}C_3$-Malonyl-CoA	0	20	Positive	857.06	305.169	37.15	118.36
$^{13}C_3$-Malonyl-CoA	0	20	Positive	857.06	350.169	27.8	118.36
$^{13}C_3$-Malonyl-CoA	0	20	Positive	857.06	410.071	24.11	118.36
$^{13}C_3$-Malonyl-CoA	0	20	Positive	857.06	428.04	27.04	118.36
$^{13}C_3$-Malonyl-CoA	0	20	Positive	857.06	448.151	22.19	118.36
YnP-CoA	0	20	Positive	1002.32	410.111	30	130
YnP-CoA	0	20	Positive	1002.32	428.11	30	130
YnP-CoA	0	20	Positive	1002.32	495.36	30	130

YnP-CoA	0	20	Positive	1002.32	593.37	30	130
C16:0-CoA	0	20	Positive	1006.33	397.333	30.98	144
C16:0-CoA	0	20	Positive	1006.33	410.111	26.48	144
C16:0-CoA	0	20	Positive	1006.33	428.111	29.97	144
C16:0-CoA	0	20	Positive	1006.33	499.444	33.46	144
18-Bz-CoA	0	20	Positive	1135.4	410.111	30	130
18-Bz-CoA	0	20	Positive	1135.4	428.111	30	130
18-Bz-CoA	0	20	Positive	1135.4	628.41	30	130
18-Bz-CoA	0	20	Positive	1135.4	726.43	30	130

Supplementary Table 8. Guide RNAs for generation of ZDHHC20 knock-out cell lines

Guide RNA	Forward Oligo (5' → 3')	Reverse Oligo (5' → 3')	Exon Targeted
gRNA1	CACCGCGCGCACCCACGTTTTC ATA	AAACTATGAAAACGTGGGTGCGCG C	9
gRNA2	CACCGGAAGCTGATGTGGTATA GAT	AAACATCTATACCACATCAGCTTCC	4

Supplementary Table 9. Comparison of -Log Student's t-test values in significantly enriched substrates in the inducible Flp-In™ T-REx™ ZDHHC20[Y181G] system and observed in the overexpressed ZHHC20[Y181G] system.

Gene Names	Flp-In™ T-REx™ ZDHHC20[Y181G]	Overexpressed ZHHC20[Y181G in HEK293T]
NCAM1	3.99684	0.236981386
CD81	2.35145	1.624676404
SCARB1	2.06502	2.728360518
LMAN2	3.12472	2.276817726
GPX8	1.65215	5.035153985
SLC3A2	2.7649	2.196013052
BET1;DKFZp781C0425	2.57035	2.667884292
PTRH2	2.72686	4.359553832
SCAMP3	2.0937	3.237179303
SCAMP1	3.31211	3.52992149
STX7	3.31363	6.099594143
CYB5B	3.8324	2.221461403
CPD	4.25644	1.161774621
TFRC	3.83118	2.757314377
IGF2R	2.75487	2.633142222
CANX	4.15653	0.658001813
VAMP7	2.04805	0.849621078

CKAP4	4.12575	5.967218567
MLEC	4.52191	0.072233846
SLC1A5	3.31353	4.645900762
VAMP3	3.29348	1.821914871
MTDH	4.01342	2.289114481
LSR	2.99476	1.185664251
TSPAN14	1.99335	4.011930624
SLC35B2	1.76436	4.44602433
NCSTN	2.90123	0.518187726
GLG1	1.82921	0.296333714
TMX3	4.15625	0.007419209
SLC38A2	1.74294	2.659194662
SORT1	3.46093	0.401372241
TMX4	2.48304	1.186091139
TMX1	2.3788	2.953057551
DNAJC5	3.06901	1.162369768
PTGFRN	4.03202	0.466283984
TMX2	2.19996	3.190539947
SLC30A1	3.30438	1.216217635

Supplementary Figures

A

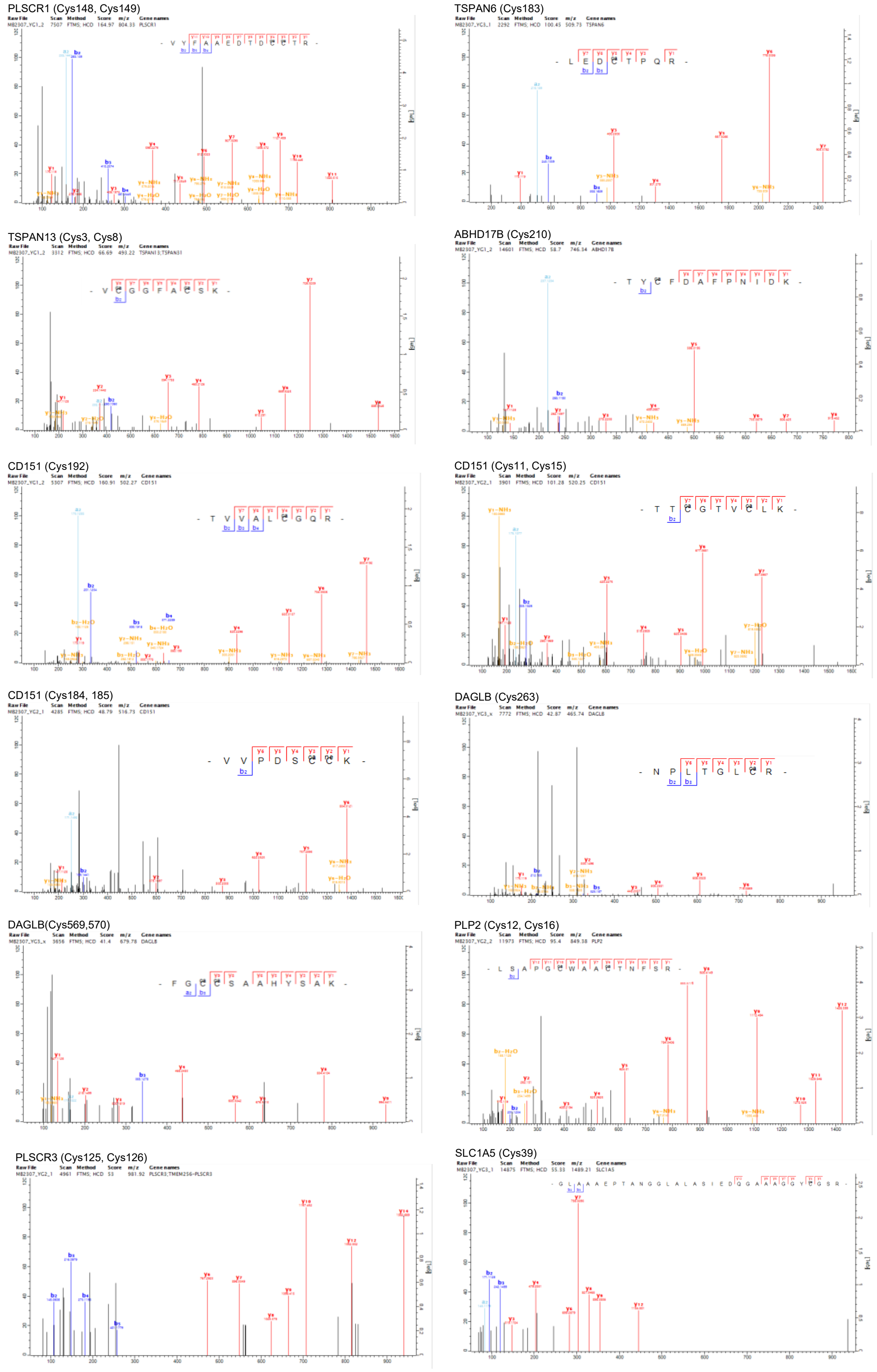

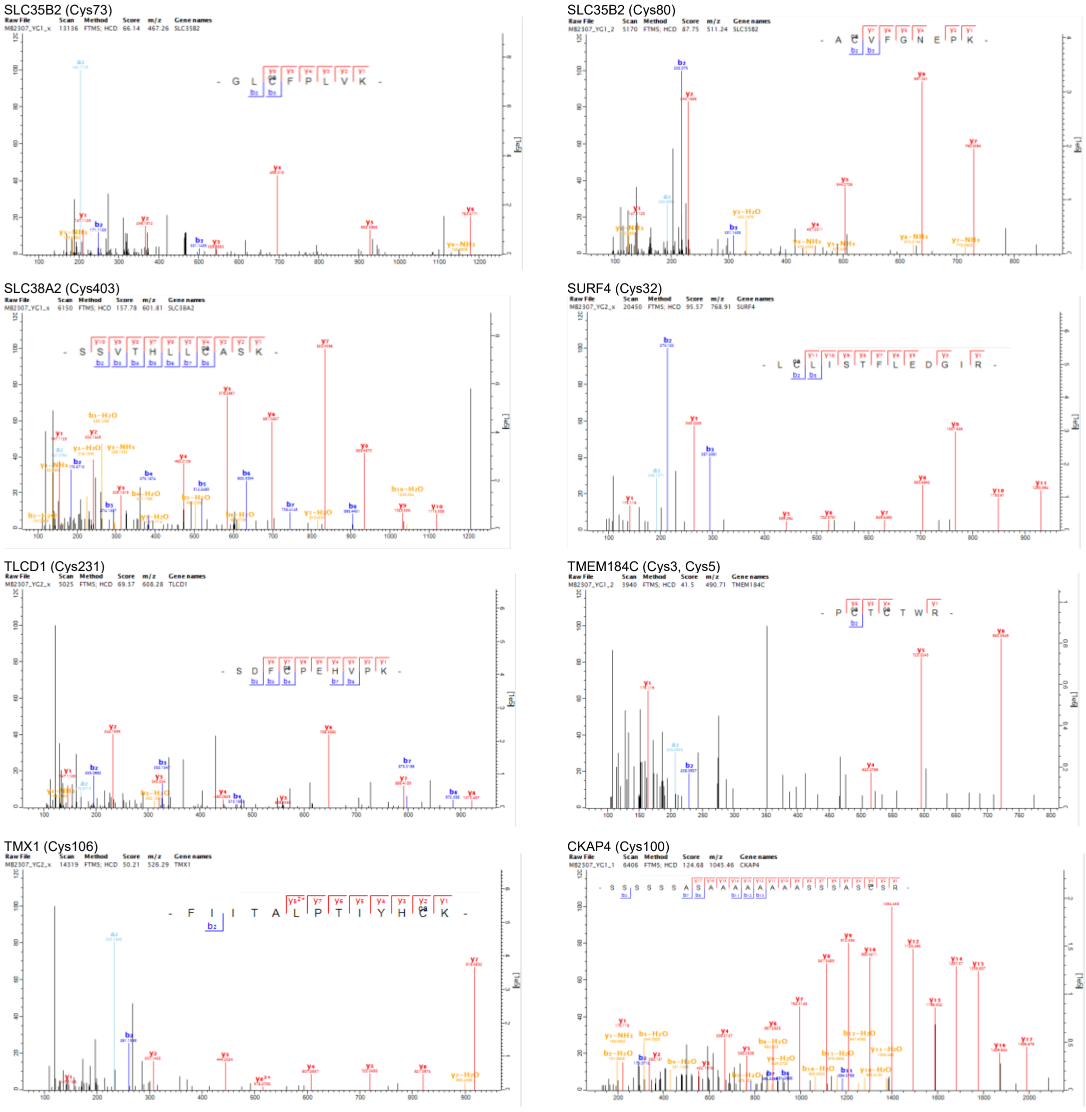

SLC35B2 (Cys73)
SLC35B2 (Cys80)
SLC38A2 (Cys403)
SURF4 (Cys32)
TLCD1 (Cys231)
TMEM184C (Cys3, Cys5)
TMX1 (Cys106)
CKAP4 (Cys100)

B

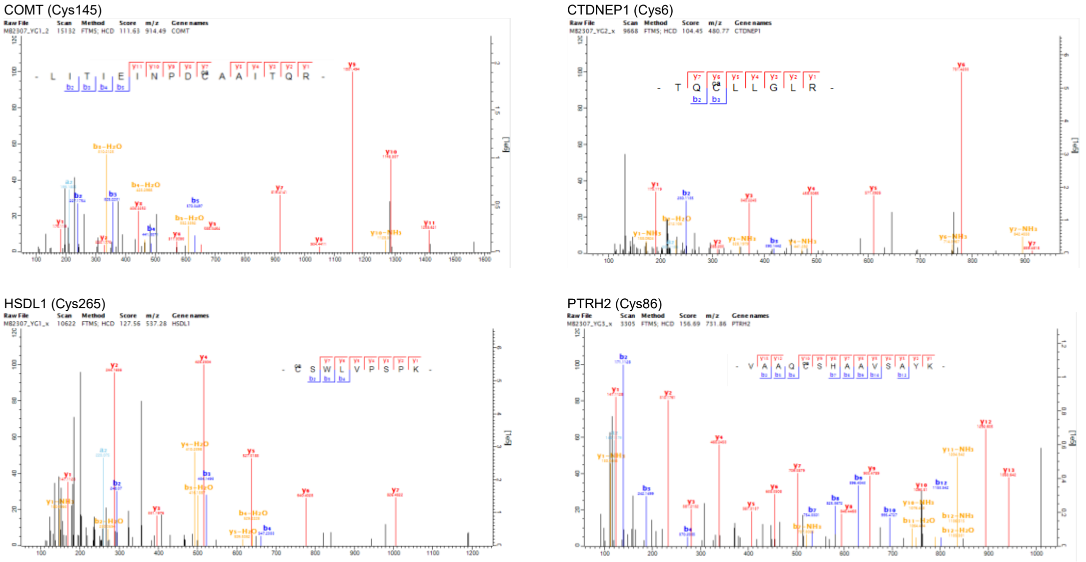

COMT (Cys145)
CTDNEP1 (Cys6)
HSDL1 (Cys265)
PTRH2 (Cys86)

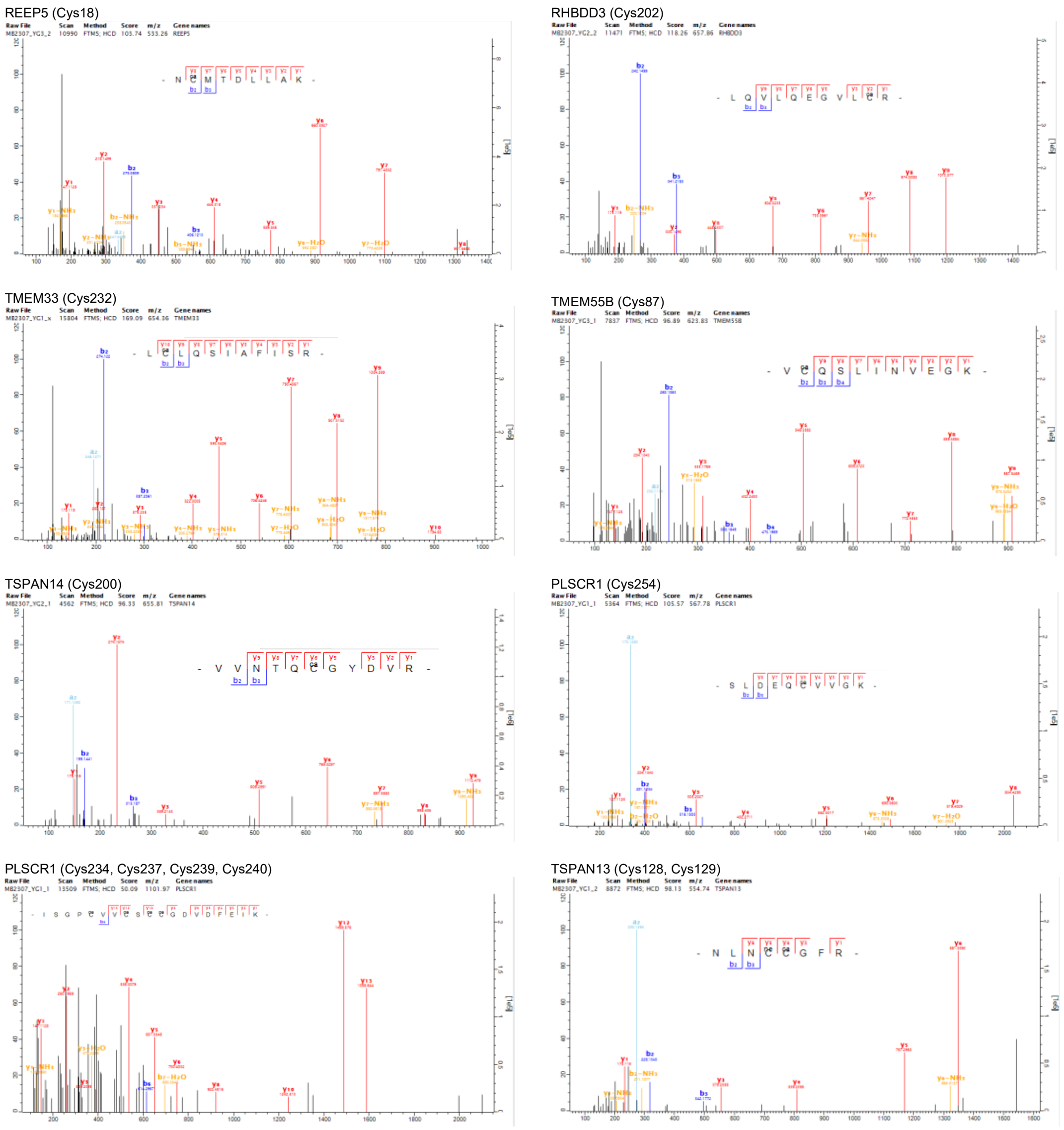

Supplementary Figure 1. MS/MS spectra of putative previously and newly identified ZDHHC20 mediated S-acylation sites in HEK293T cells. MS/MS spectra of sites identified to be carbamidomethylated during on-bead hydrolysis workflow of HEK293T cells. All the sites identified were from proteins enriched within the ZDHHC20[Y181G] expressing cells when treated with 18-Bz and therefore are indicative of a ZDHHC20 *S*-acylation site. Sites were curated as being detected within the mutant expressing samples and spectra inspected by eye. (**A**) These sites correspond to those either identified as being *S*-acylated in palmitoyl-proteome experiments or validated as sites of *S*-acylation[6]. (**B**) The sites identified correspond to those not previously identified through other studies which could correspond to novel sites of *S*-acylation.

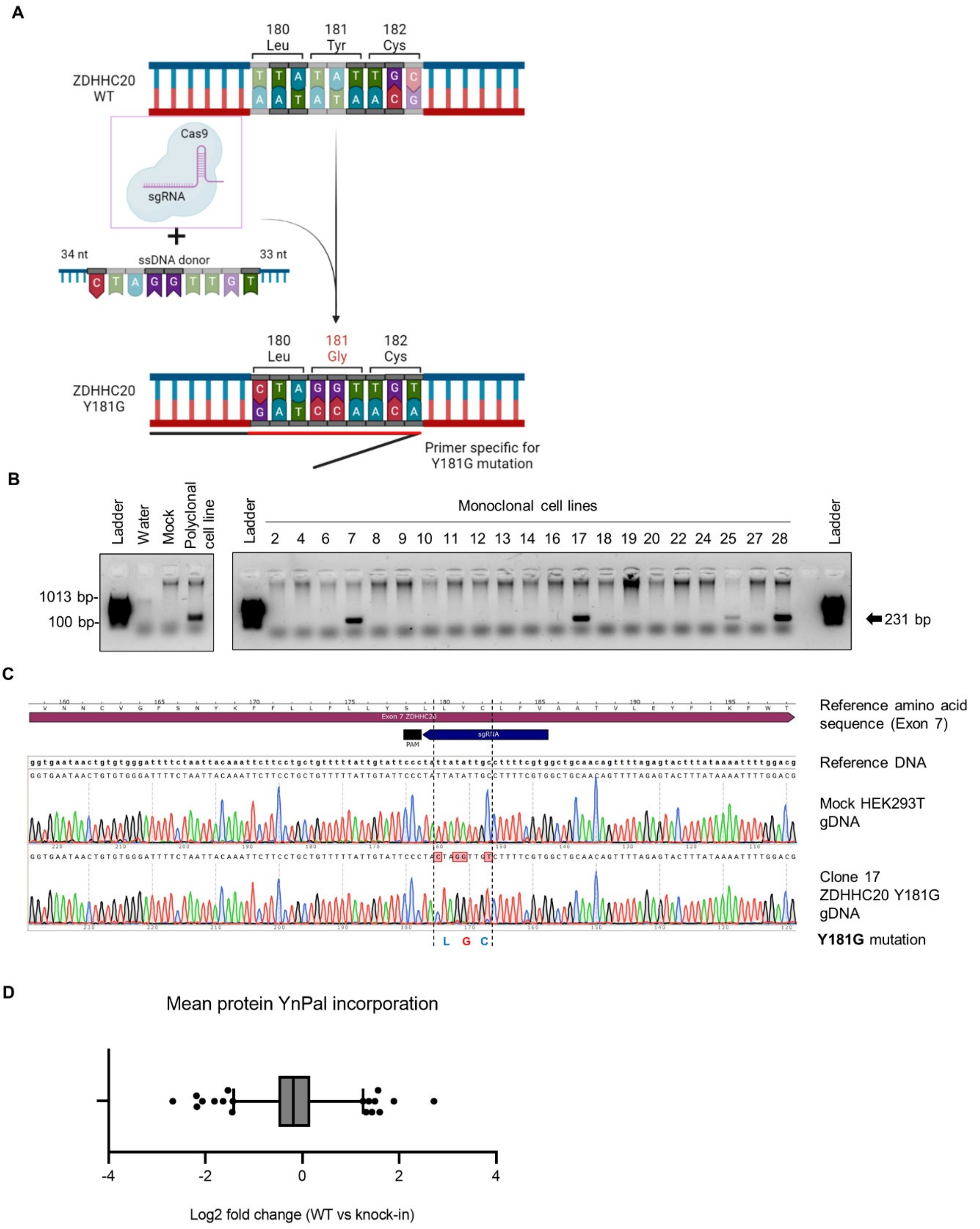

Supplementary Figure 2. Knock-in ZDHHC20Y181G mutant design and validation in HEK293T cell line. (**A**) Knock-in design strategy to generate ZDHHC20Y181G mutant via homologous double strand recombination using single strand DNA as template (**B**) Validation of *Left:* PCR controls in 2% agarose gels to probe for Y181G genomic integration using water, mock (negative) and parental polyclonal cell line (positive). *Right:* As in left panel, but showing PCR products in mono clonal cell lines (1 biological replicate). (**C**) Alignment of Sanger sequencing traces of mock and positive homozygous clone 17 with wild type sequence of Exon 7 in ZDHHC20. In maroon, coding sequence of exon 7 of ZDHHC20. In blue, target sequence and direction of sgRNA used in this study. (**D**) Box and whisker plots represent median values (center lines) and 25th and 75th percentiles (box limits) with Tukey whiskers

graph showing LC-MSMS (nanoElute2 and timstofHT) data of mean incorporation log2 fold-change between knock-in ZDHHC[20Y181G] vs ZDHHC20 WT treated with YnPal (n= 4 independent biological replicates).

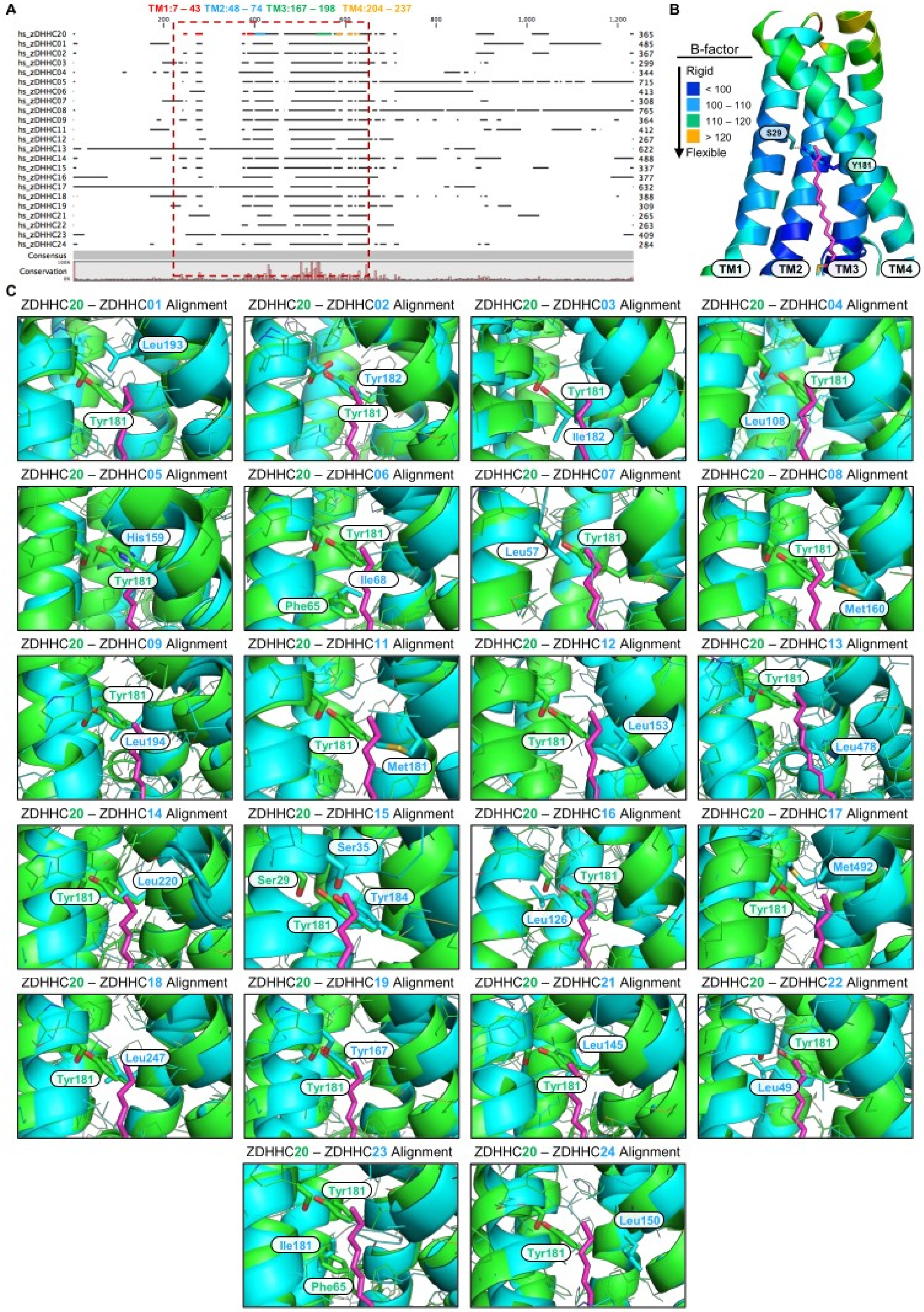

Supplementary Figure 3. **Expansion of ZDHHC chemical genetics.** (**A**) Multiple protein sequence alignments of all ZDHHC family members. The transmembrane helices (TMs) 1 - 4

of ZDHHC20 are indicated in red, cyan, blue and green, respectively. The ZDHHC protein sequences overlapping with ZDHHC20 TMs 1 - 4 are within the dotted, dark red border. (**B**) The transmembrane domain of ZDHHC20 (PDB entry 6BML). The B-factor coloring scheme (*/ca) was applied to secondary structures. Note that Ser29 and Tyr181 pinch helices TM1 and TM3 together through hydrogen-bonding. (**C**) ZDHHC homology models (blue) were generated using Phyre2 and structurally aligned to ZDHHC20 (green). Residue labels for ZDHHC20 Ser29, Tyr181 and Phe65 (green) and all others (ZDHHCs 1-19 and 21-24, blue) are positioned adjacent to their respective residues. Blue labels highlight residues that serve as putative sites for mutagenesis and bump-hole engineering.

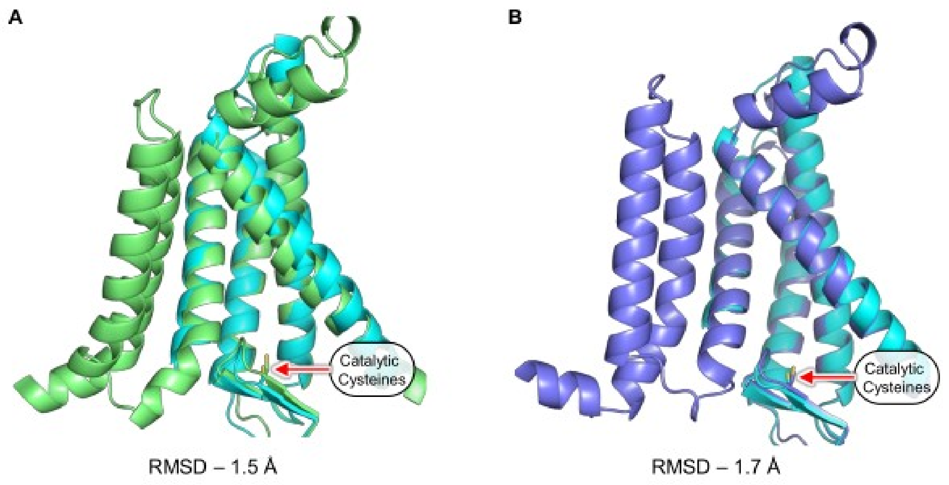

Supplementary Figure 4. Structural alignments of full and core ZDHHC13 and 17 homology models. (**A-B**) Homology models of complete ZDHHC13 (**A**) and ZDHHC17 (**B**) were downloaded from the AlphaFold Protein Structure Database or generated by PHYRE2 using sequences of the four-TM helical core. Each set of models were aligned using Pymol and agreement reported as the root-mean-square deviation between protein backbones.

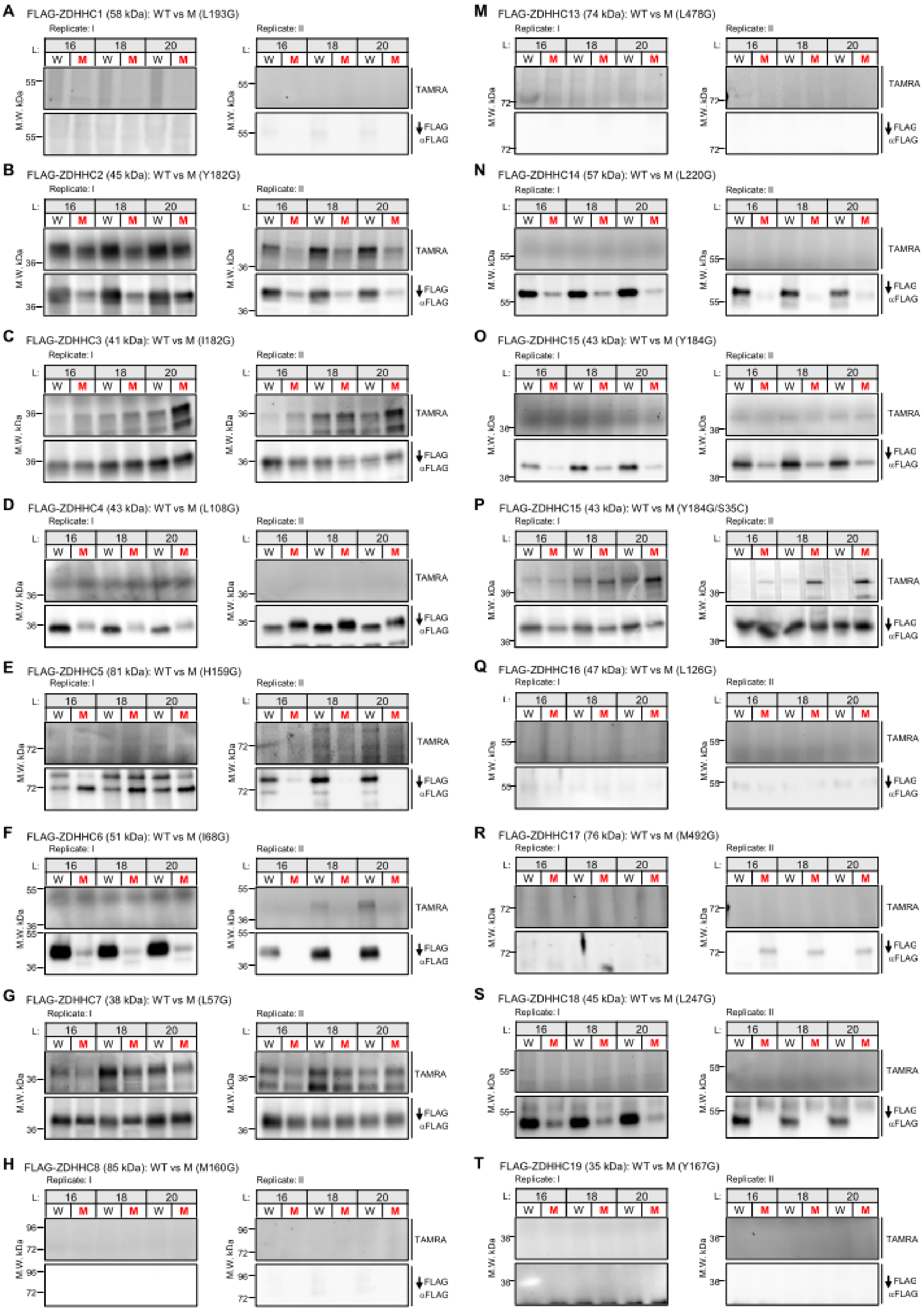
A FLAG-ZDHHC1 (58 kDa): WT vs M (L193G)
B FLAG-ZDHHC2 (45 kDa): WT vs M (Y182G)
C FLAG-ZDHHC3 (41 kDa): WT vs M (I182G)
D FLAG-ZDHHC4 (43 kDa): WT vs M (L108G)
E FLAG-ZDHHC5 (81 kDa): WT vs M (H159G)
F FLAG-ZDHHC6 (51 kDa): WT vs M (I68G)
G FLAG-ZDHHC7 (38 kDa): WT vs M (L57G)
H FLAG-ZDHHC8 (85 kDa): WT vs M (M160G)
M FLAG-ZDHHC13 (74 kDa): WT vs M (L478G)
N FLAG-ZDHHC14 (57 kDa): WT vs M (L220G)
O FLAG-ZDHHC15 (43 kDa): WT vs M (Y184G)
P FLAG-ZDHHC15 (43 kDa): WT vs M (Y184G/S35C)
Q FLAG-ZDHHC16 (47 kDa): WT vs M (L126G)
R FLAG-ZDHHC17 (76 kDa): WT vs M (M492G)
S FLAG-ZDHHC18 (45 kDa): WT vs M (L247G)
T FLAG-ZDHHC19 (35 kDa): WT vs M (Y167G)
Replicate: I
Replicate: II
L:
16
18
20
W
M
M.W. kDa
TAMRA
FLAG
αFLAG

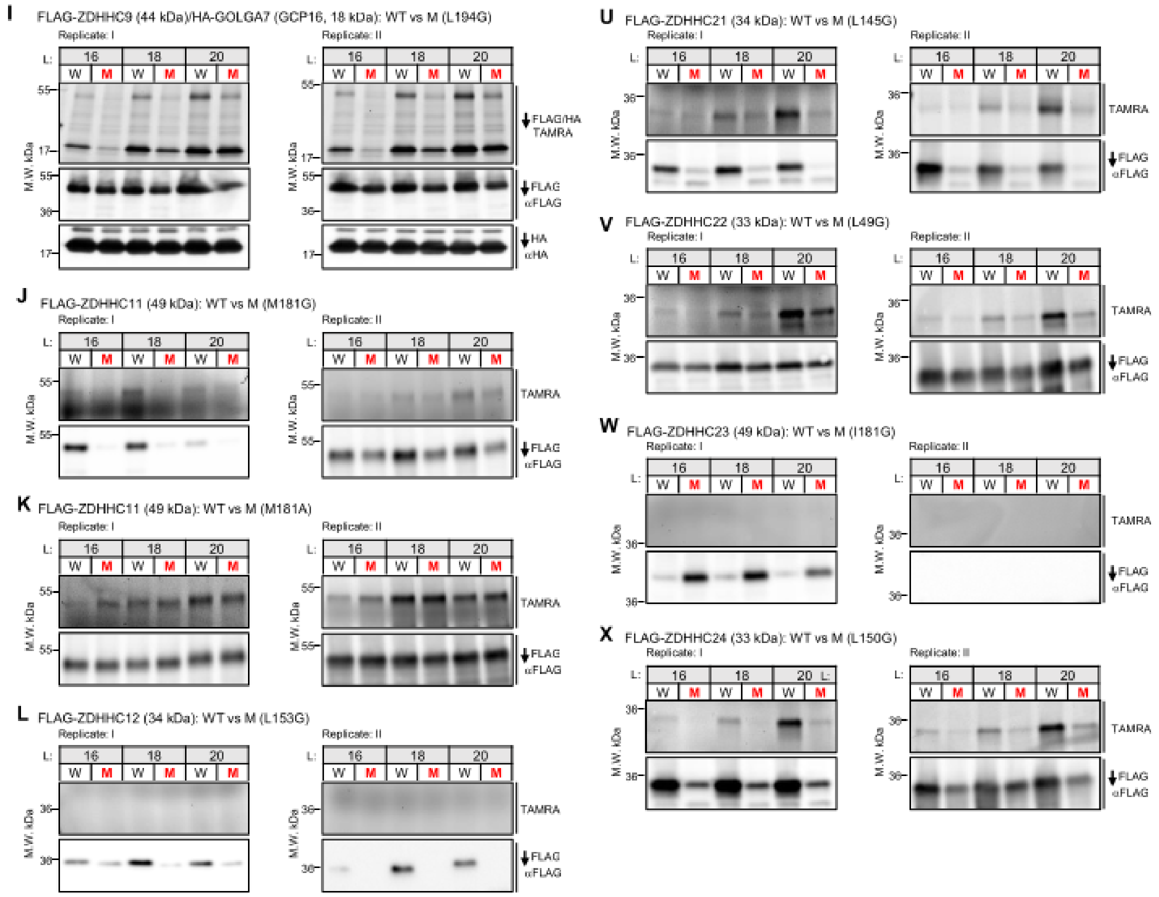

Supplementary Figure 5. Chain length optimization against putative chemical genetics enabling ZDHHC mutants. (**A-X**) The indicated single or double mutants of FLAG-tagged ZDHHC family members were generated and subjected to loading assays with probes containing chain-lengths of 16, 18 and 20 Acetyl bump (denoted 16, 18, and 20). Note that for ZDHHC9 (**I**) loading assays, cells were co-transfected with ZDHHC9 and HA-tagged GOLGA7 (GCP16). Wild-type (WT or W in tables) and mutant (M) constructs were transiently transfected into HEK293T cells and treated with 15 μM probe for 4 h. After cell lysis, constructs were immunoprecipitated on anti-FLAG resin, clicked with TAMRA-azide and separated by SDS-PAGE. Assays were conducted in duplicate and loading and input were visualized by in-gel fluorescence and anti-FLAG or -HA immunoblot, respectively (n=2 independent biological replicates).

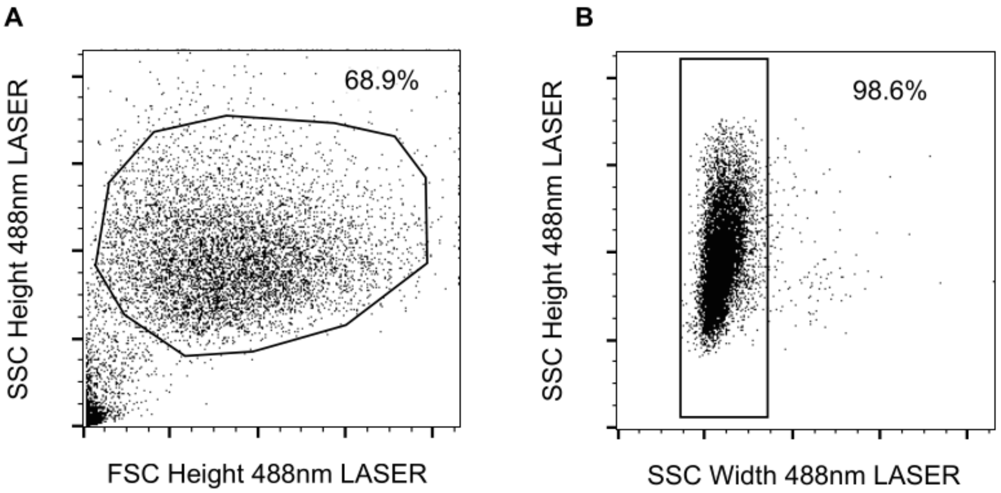

Supplementary Figure 6. **Gating strategy for single cell sorting.** (**A-B**) Example of data analyzed in FlowJo. (**A**) Cells were separated from the debris using side scatter (SSC) height vs. forward scatter (FSC) height. (**B**) Single cells were then identified using SSC height vs. SSC width. Single cells were sorted into 96-well plates on the Beckman Coulter MoFlo XDP using the Single Cell sort mask with a drop envelope of 0.5.

Supplementary Information – References

6159 Yang, X., Ma, Y., Li, N., Cai, H. & Bartlett, M. G. Development of a Method for the Determination of Acyl-CoA Compounds by Liquid Chromatography Mass Spectrometry to Probe the Metabolism of Fatty Acids. *Anal Chem* **89**, 813-821, doi:10.1021/acs.analchem.6b03623 (2017).

60 Matyash, V., Liebisch, G., Kurzchalia, T. V., Shevchenko, A. & Schwudke, D. Lipid extraction by methyl-tert-butyl ether for high-throughput lipidomics. *J Lipid Res* **49**, 1137-1146, doi:10.1194/jlr.D700041-JLR200 (2008).

61 Cajka, T. & Fiehn, O. Increasing lipidomic coverage by selecting optimal mobile-phase modifiers in LC–MS of blood plasma. *Metabolomics* **12**, doi:10.1007/s11306-015-0929-x (2016).

62 Tsugawa, H. *et al.* MS-DIAL: data-independent MS/MS deconvolution for comprehensive metabolome analysis. *Nat Methods* **12**, 523-526, doi:10.1038/nmeth.3393 (2015).

63 Koelmel, J. P. *et al.* LipidMatch: an automated workflow for rule-based lipid identification using untargeted high-resolution tandem mass spectrometry data. *BMC Bioinformatics* **18**, 331, doi:10.1186/s12859-017-1744-3 (2017).

64 Kessner, D., Chambers, M., Burke, R., Agus, D. & Mallick, P. ProteoWizard: open source software for rapid proteomics tools development. *Bioinformatics* **24**, 2534-2536, doi:10.1093/bioinformatics/btn323 (2008).

Uncropped Scans of Supplementary Information blots

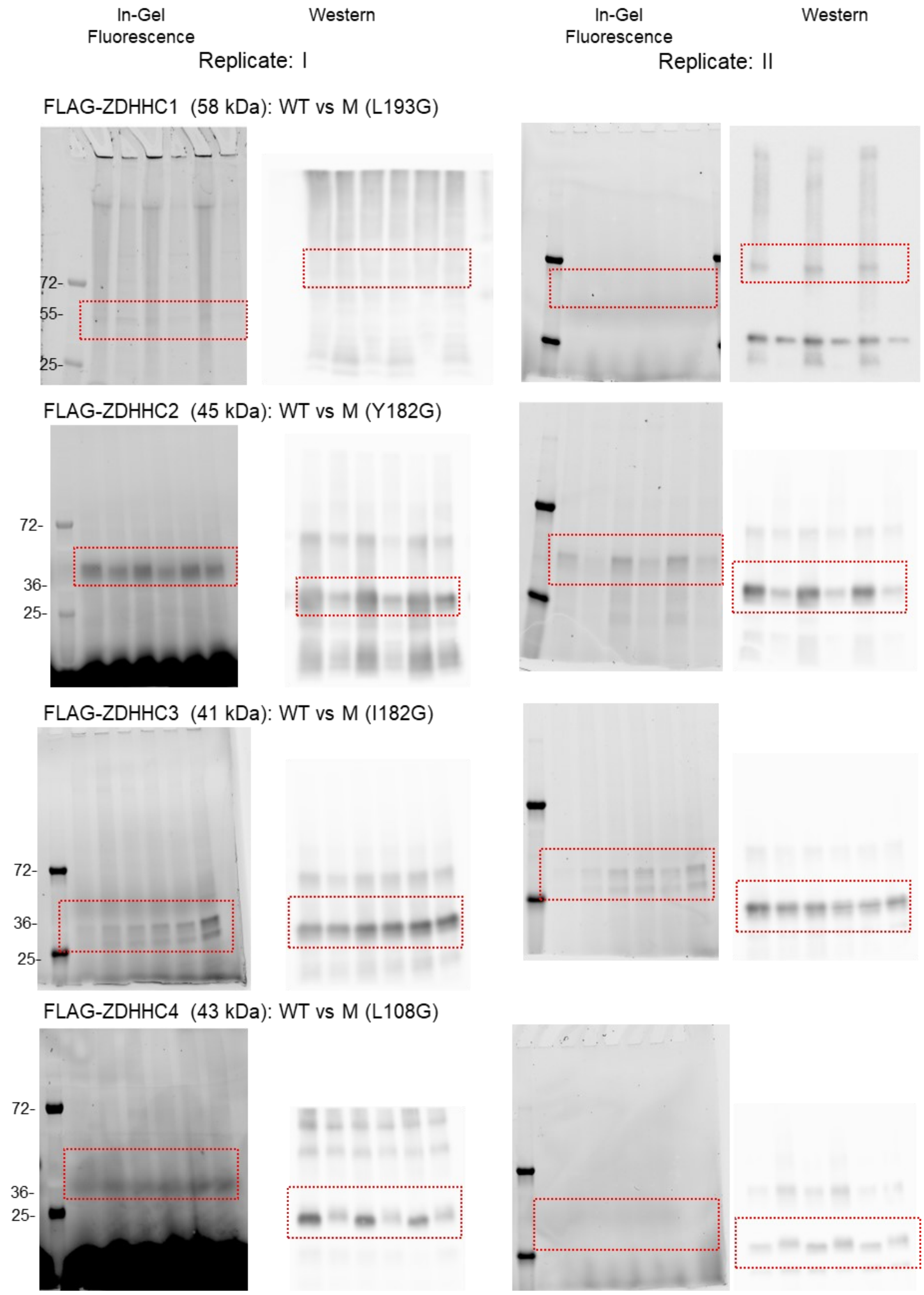

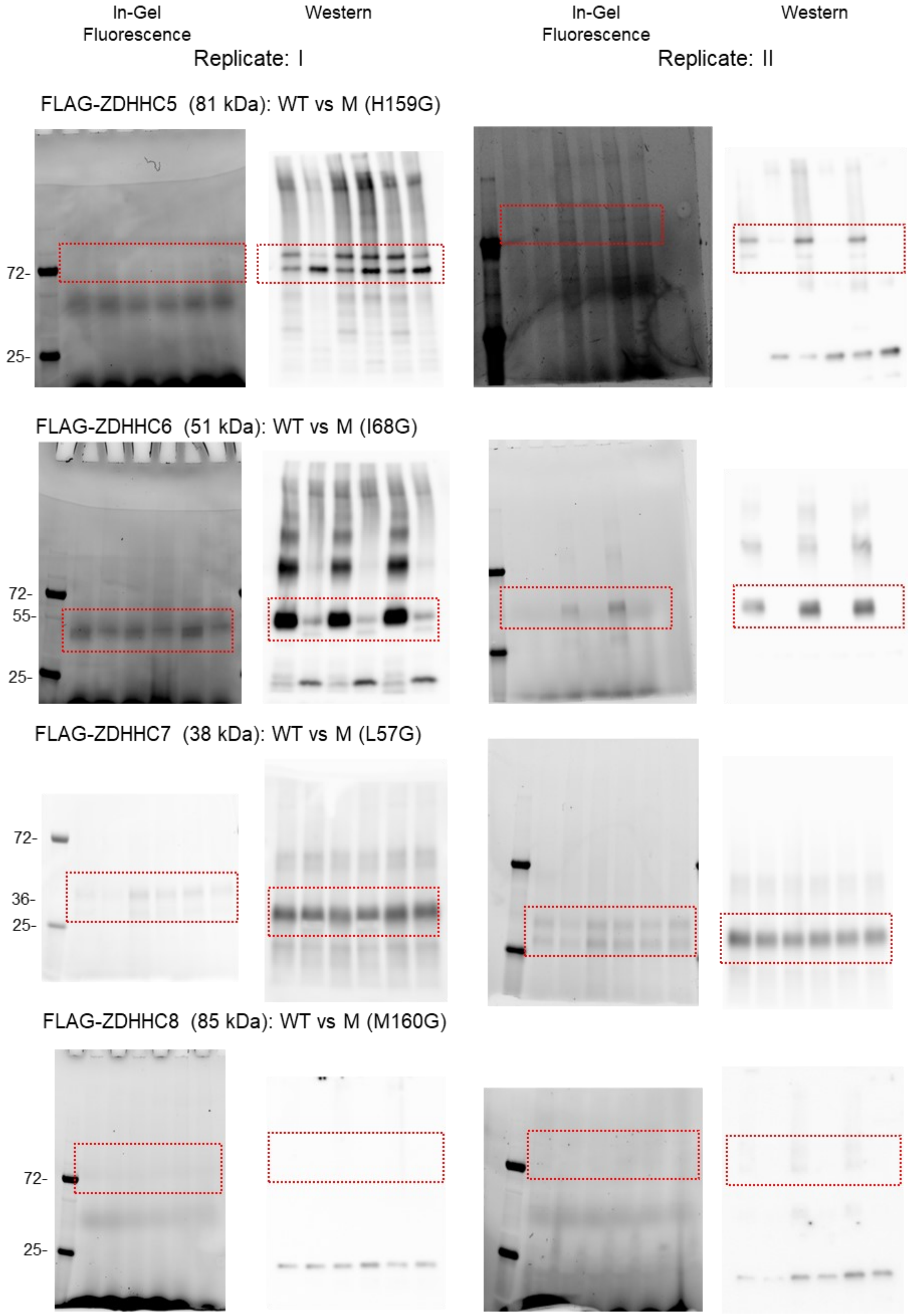
In-Gel Fluorescence
Western
Replicate: I
In-Gel Fluorescence
Western
Replicate: II
FLAG-ZDHHC5 (81 kDa): WT vs M (H159G)
72-
25-
FLAG-ZDHHC6 (51 kDa): WT vs M (I68G)
72-
55-
25-
FLAG-ZDHHC7 (38 kDa): WT vs M (L57G)
72-
36-
25-
FLAG-ZDHHC8 (85 kDa): WT vs M (M160G)
72-
25-

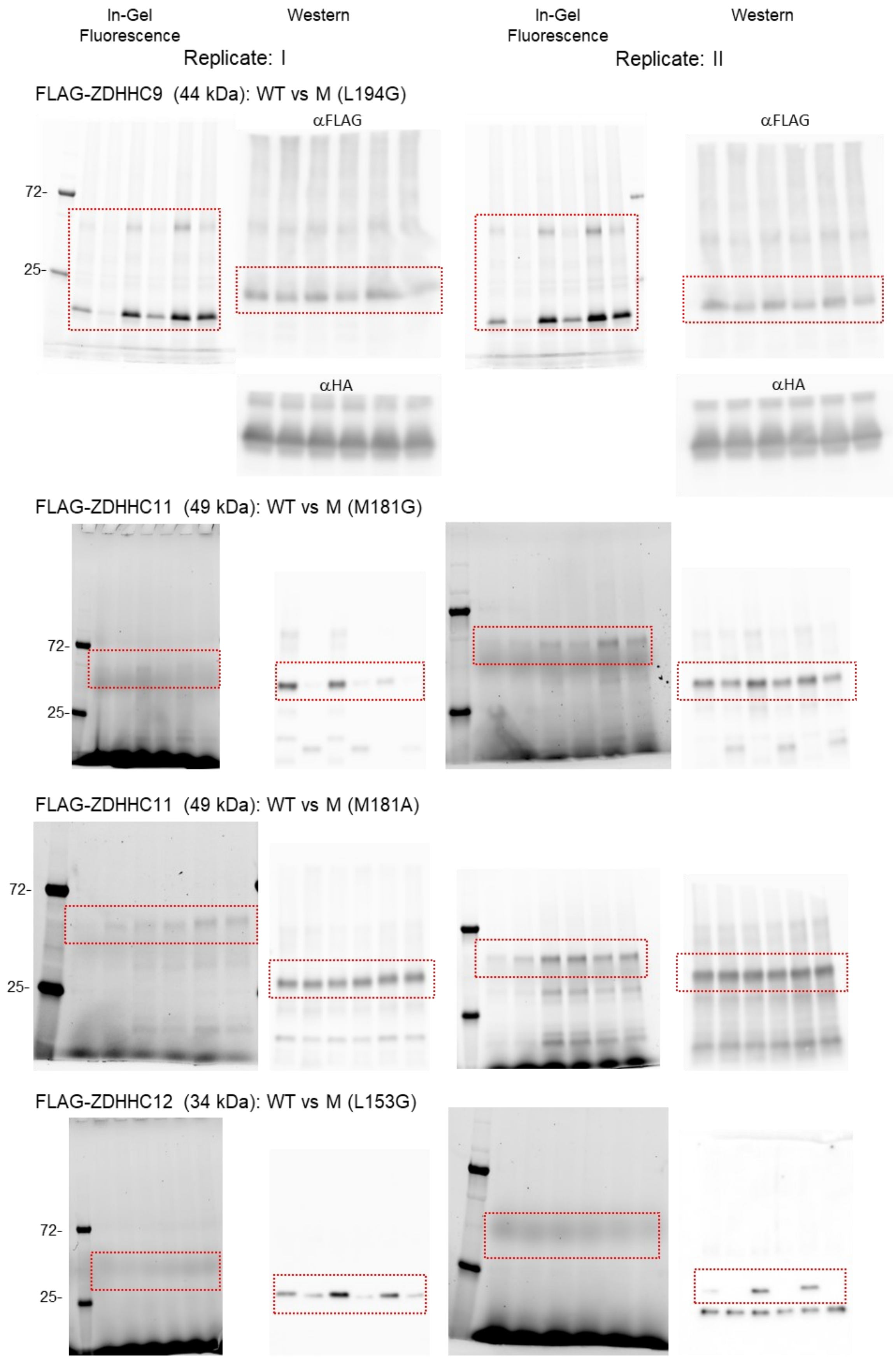
In-Gel Fluorescence
Western
Replicate: I
In-Gel Fluorescence
Western
Replicate: II
FLAG-ZDHHC9 (44 kDa): WT vs M (L194G)
αFLAG
αFLAG
72-
25-
αHA
αHA
FLAG-ZDHHC11 (49 kDa): WT vs M (M181G)
72-
25-
FLAG-ZDHHC11 (49 kDa): WT vs M (M181A)
72-
25-
FLAG-ZDHHC12 (34 kDa): WT vs M (L153G)
72-
25-

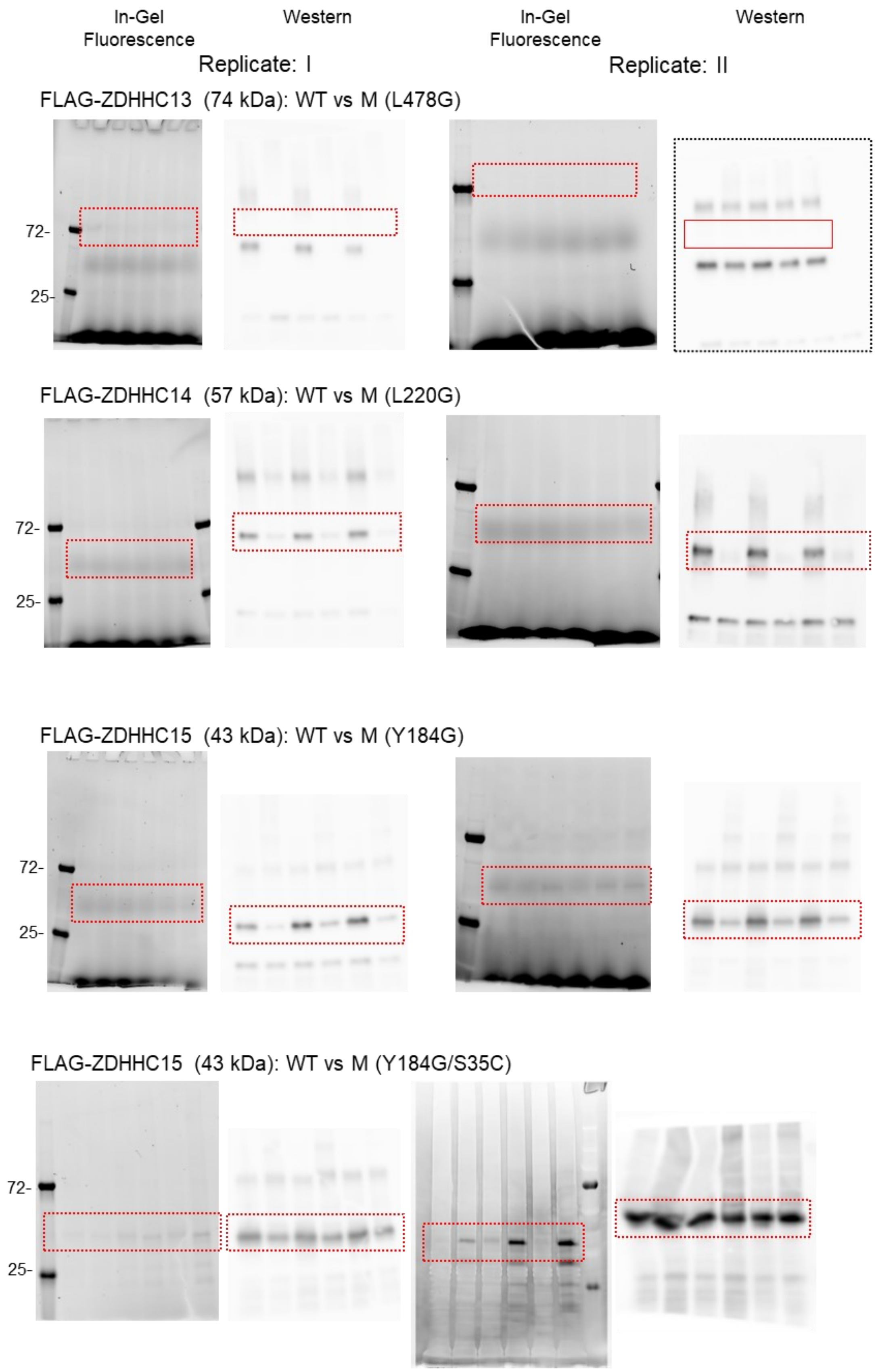
In-Gel Fluorescence
Western
Replicate: I
In-Gel Fluorescence
Western
Replicate: II
FLAG-ZDHHC13 (74 kDa): WT vs M (L478G)
72-
25-
FLAG-ZDHHC14 (57 kDa): WT vs M (L220G)
72-
25-
FLAG-ZDHHC15 (43 kDa): WT vs M (Y184G)
72-
25-
FLAG-ZDHHC15 (43 kDa): WT vs M (Y184G/S35C)
72-
25-

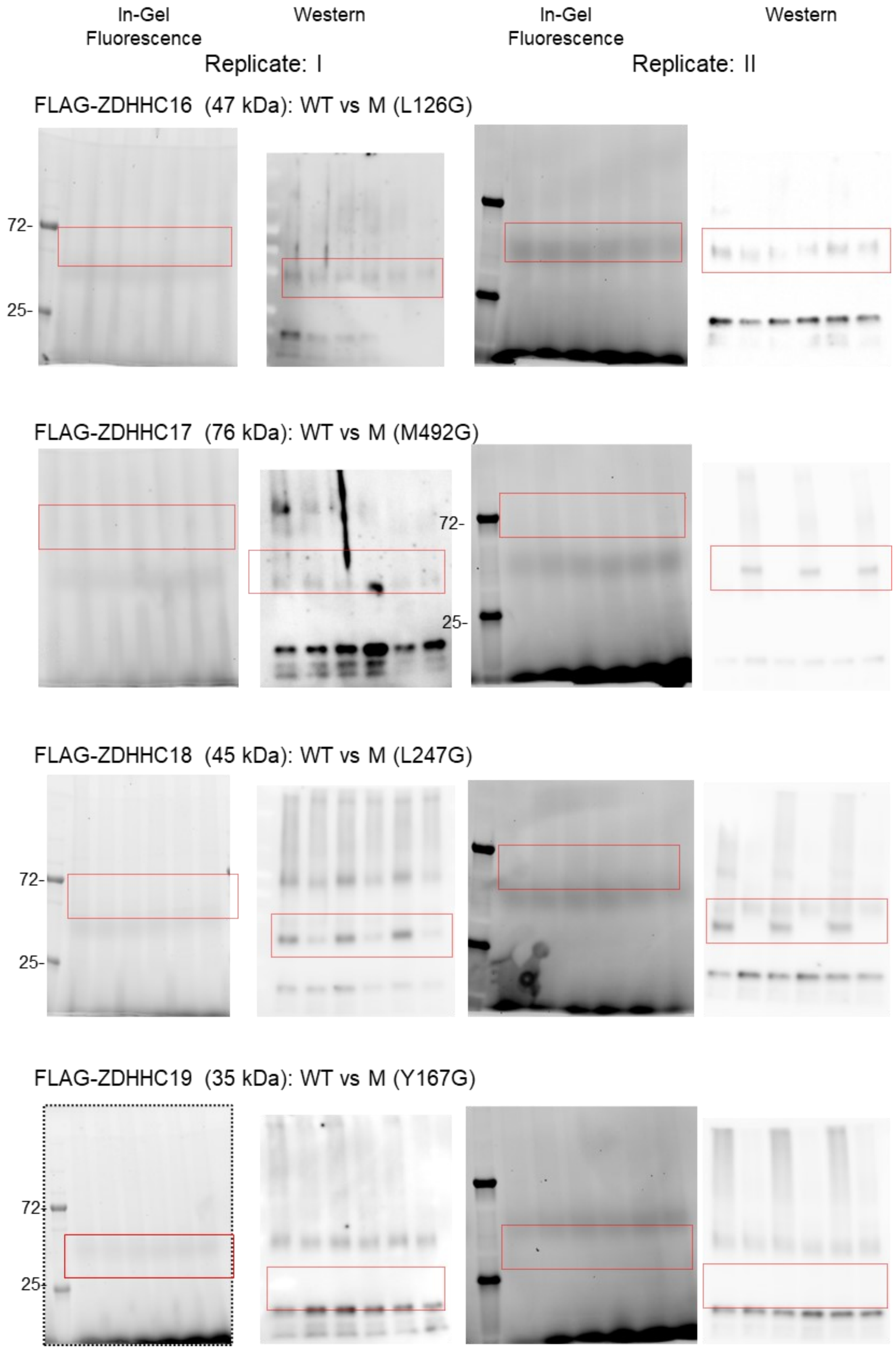
In-Gel Fluorescence
Western
In-Gel Fluorescence
Western
Replicate: I
Replicate: II
FLAG-ZDHHC16 (47 kDa): WT vs M (L126G)
72-
25-
FLAG-ZDHHC17 (76 kDa): WT vs M (M492G)
72-
25-
FLAG-ZDHHC18 (45 kDa): WT vs M (L247G)
72-
25-
FLAG-ZDHHC19 (35 kDa): WT vs M (Y167G)
72-
25-

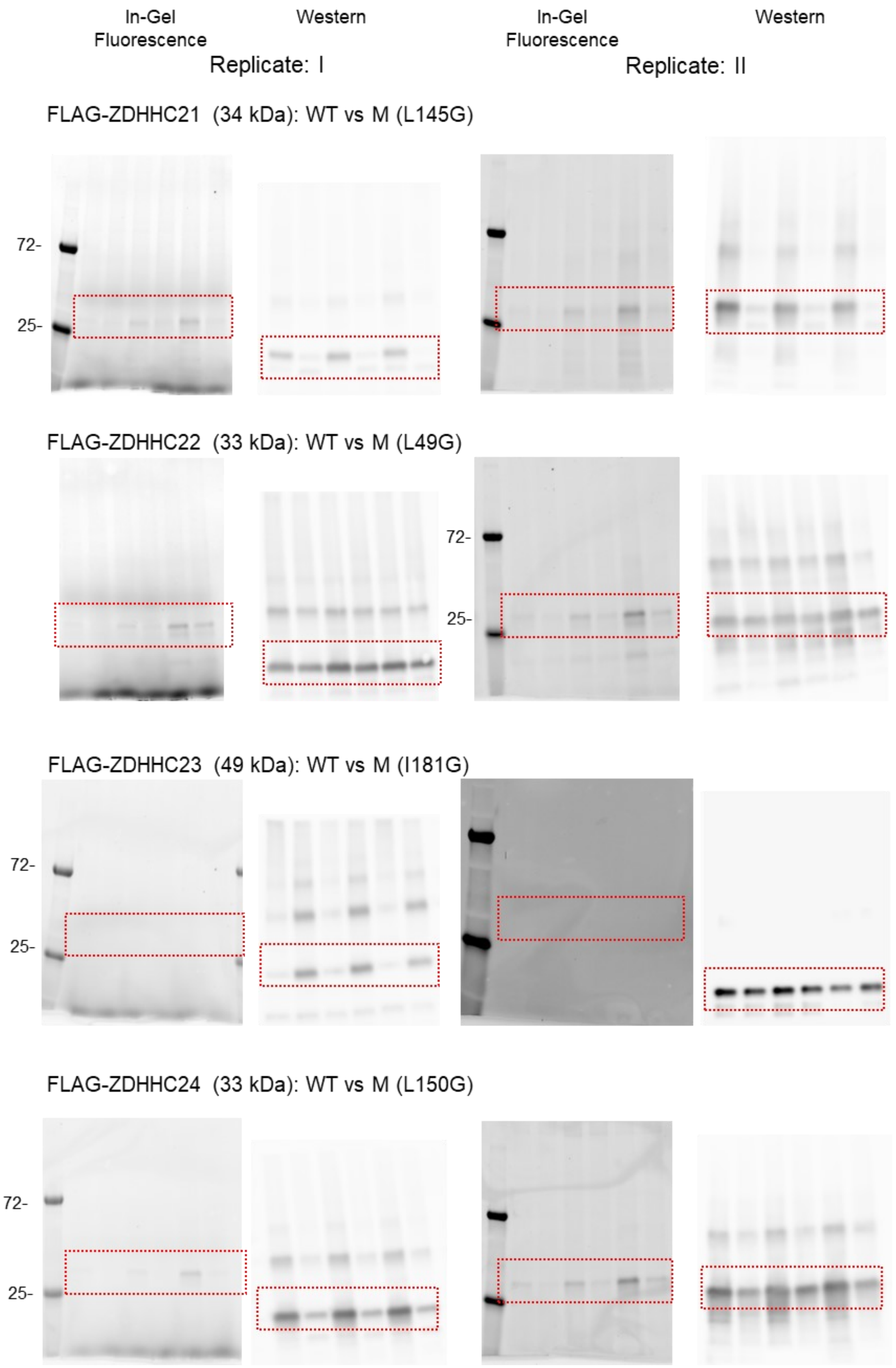

Uncropped western blots for Supplementary Figure 5. Areas within red borders are in the supplementary figure.
