# Supplementary material for: A palmitoyl transferase chemical–genetic system to map ZDHHC-specific S-acylation
Source: Nat Biotechnol. 2024 Jan 8;42(10):1548–58. doi: 10.1038/s41587-023-02030-0 (PMC11471619; doi:10.1038/s41587-023-02030-0)
Supplement: Supplementary file 2 — Reporting Summary [file 41587_2023_2030_MOESM2_ESM.pdf]

nature portfolio

Corresponding author(s): Edward Tate

Last updated by author(s): Oct 10, 2023

Reporting Summary

Nature Portfolio wishes to improve the reproducibility of the work that we publish. This form provides structure for consistency and transparency in reporting. For further information on Nature Portfolio policies, see our Editorial Policies and the Editorial Policy Checklist.

Statistics

For all statistical analyses, confirm that the following items are present in the figure legend, table legend, main text, or Methods section.

n/a	Confirmed	
☐	☒	The exact sample size (*n*) for each experimental group/condition, given as a discrete number and unit of measurement
☐	☒	A statement on whether measurements were taken from distinct samples or whether the same sample was measured repeatedly
☐	☒	The statistical test(s) used AND whether they are one- or two-sided<br>*Only common tests should be described solely by name; describe more complex techniques in the Methods section.*
☒	☐	A description of all covariates tested
☒	☐	A description of any assumptions or corrections, such as tests of normality and adjustment for multiple comparisons
☐	☒	A full description of the statistical parameters including central tendency (e.g. means) or other basic estimates (e.g. regression coefficient) AND variation (e.g. standard deviation) or associated estimates of uncertainty (e.g. confidence intervals)
☐	☒	For null hypothesis testing, the test statistic (e.g. *F*, *t*, *r*) with confidence intervals, effect sizes, degrees of freedom and *P* value noted<br>*Give P values as exact values whenever suitable.*
☒	☐	For Bayesian analysis, information on the choice of priors and Markov chain Monte Carlo settings
☒	☐	For hierarchical and complex designs, identification of the appropriate level for tests and full reporting of outcomes
☒	☐	Estimates of effect sizes (e.g. Cohen's *d*, Pearson's *r*), indicating how they were calculated

Our web collection on statistics for biologists contains articles on many of the points above.

Software and code

Policy information about availability of computer code

Data collection	Typhoon FLA 9500 version 1.1 (GE Healthcare), MaxQuant v.1.6.7.0 (free, https://maxquant.net/maxquant/), Tecan SPARK, Odyssey ClX1, Amersham imager 600, Xcalibur 4.0.27.10 (V4.5.445.18 and V3.0.63) (Thermo Fisher Scientific)
Data analysis	Perseus v.1.6.2.1 (free, https://maxquant.net/perseus/), Graphpad Prism (Version 9.0), Image Studio Lite 5.2 (Li-COR), Microsoft Excel, ImageJ FiJi 1.50c, QuanBrowser 4.5.445.18 (Thermo Fisher Scientific), Mass Hunter Profinder (v. 10.0, Agilent Technologies), LipidMatch (v. 3.5), MSConvertGUI (ProteoWizard), PANTHER GO version 16 and version 18 analysis software, The PyMOL Molecular Graphics System (Version 2.0 Schrödinger, LLC)

For manuscripts utilizing custom algorithms or software that are central to the research but not yet described in published literature, software must be made available to editors and reviewers. We strongly encourage code deposition in a community repository (e.g. GitHub). See the Nature Portfolio guidelines for submitting code & software for further information.

Data

Policy information about availability of data

All manuscripts must include a data availability statement. This statement should provide the following information, where applicable:

- Accession codes, unique identifiers, or web links for publicly available datasets
- A description of any restrictions on data availability
- For clinical datasets or third party data, please ensure that the statement adheres to our policy

The mass spectrometry proteomics data have been deposited to the ProteomeXchange Consortium via the PRIDE partner repository with the dataset identifier PXD032373 and PXD032378. Lipidomics (10.25418/crick.24279541) and metabolomics (10.25418/crick.24279838) datasets have been uploaded to Figshare.

PDB entry 6BML3 was used for Figure 1.
Uncropped gel data is shown in a separately attached supplementary file.

Field-specific reporting

Please select the one below that is the best fit for your research. If you are not sure, read the appropriate sections before making your selection.

☒ Life sciences ☐ Behavioural & social sciences ☐ Ecological, evolutionary & environmental sciences

For a reference copy of the document with all sections, see nature.com/documents/nr-reporting-summary-flat.pdf

Life sciences study design

All studies must disclose on these points even when the disclosure is negative.

Sample size	No sample size-calculations were performed. All cell-based experiments were performed in triplicate. For chemical proteomics analysis, all biological conditions were tested in triplicates or quadruplicate. For each cell line based assay experiment the sample size was appropriately adjusted based on the experiment itself.
Data exclusions	No data were excluded
Replication	All replicates were succesful, performed in triplicate or quadruplicate.
Randomization	For lipodomics samples: Samples were loaded in a random order by blinded selection from pooled anonymously labelled samples.
Blinding	Blinding of samples were not suitable for the nature of the samples. Only cell-based experiments were performed for this study.

Reporting for specific materials, systems and methods

We require information from authors about some types of materials, experimental systems and methods used in many studies. Here, indicate whether each material, system or method listed is relevant to your study. If you are not sure if a list item applies to your research, read the appropriate section before selecting a response.

Materials & experimental systems

n/a	Involved in the study	
☐	☒	Antibodies
☐	☒	Eukaryotic cell lines
☒	☐	Palaeontology and archaeology
☒	☐	Animals and other organisms
☒	☐	Human research participants
☒	☐	Clinical data
☒	☐	Dual use research of concern

Methods

n/a	Involved in the study	
☒	☐	ChIP-seq
☒	☐	Flow cytometry
☒	☐	MRI-based neuroimaging

Antibodies

Antibodies used

mouse monoclonal anti-FLAG M2 antibody (F1804,), mouse monoclonal anti-HA-epitope antibody (clone HA-7, H3663), mouse monoclonal anti-alpha-tubulin antibody (clone B-5-1-2, T5168), rabbit polyclonal anti-ZDHHC20 antibody (Atlas Antibodies, HPA014702), rabbit polyclonal anti-BCAP31 antibody (Atlas Antibodies, HPA003906) and rabbit polyclonal anti-V5 antibody (SAB1306079) were purchased from Sigma Aldrich.
mouse monoclonal anti-GFP antibody (GF28R) was purchased from Generon LTD
rabbit monoclonal anti-vinculin antibody (42H89L44) was purchased from Thermo Fisher Scientific
rabbit monoclonal anti-pan cadherin antibody(EPR1792Y, ab51034), rabbit monoclonal anti-Gm130 antibody (EP892Y, ab52649), rabbit polyclonal anti-calnexin antibody (ab22595) and rabbit monoclonal anti-TOMM20 antibody (EPR15581-54, ab186735) were purchased from Abcam
rabbit polyclonal anti-TMX1 antibody (HPA003085) was purchased from Atlas Antibodies
rabbit polyclonal anti-NCAM1/CD56 antibody (14255-1-AP) and rabbit polyclonal anti-PI4K2A antibody (15318-1-AP) were purchased from proteintech
goat polyclonal anti-rabbit Immunoglobulins/HRP secondary antibody (P044801-2) and goat polyclonal anti-mouse Immunoglobulins/HRP secondary antibody (P044701-2) were purchased from Agilent Dako
goat polyclonal anti-mouse/IRDye 800CW secondary antibody (ab216772) was purchased from abcam

Validation

The following antibodies have been validated by the manufacturer (from the manufacturer's website):
https://www.sigmaaldrich.com/GB/en/product/sigma/f1804
https://www.sigmaaldrich.com/GB/en/product/sigma/h3663
https://www.sigmaaldrich.com/GB/en/product/sigma/t5168
enhanced validation, independent (Antibodies)

https://www.sigmaaldrich.com/GB/en/product/sigma/hpa014702
https://www.sigmaaldrich.com/GB/en/product/sigma/hpa003906
enhanced validation, orthogonal RNAseq
https://www.sigmaaldrich.com/GB/en/product/sigma/sab1306079
https://www.thermofisher.com/antibody/product/Vinculin-Antibody-clone-42H89L44-Recombinant-Monoclonal/700062
Advanced Verification: This Antibody was verified by Knockout to ensure that the antibody binds to the antigen stated.
https://www.abcam.com/calnexin-antibody-er-marker-ab22595.html
KO validated
https://www.abcam.com/products/primary-antibodies/tomm20-antibody-epr15581-54-mitochondrial-marker-ab186735.html
https://www.atlasantibodies.com/products/antibodies/primary-antibodies/triple-a-polyclonals/tmx1-antibody-hpa003085/
Validated in Western blot using relevant lysates
https://www.ptglab.com/products/NCAM1-Antibody-14255-1-AP.htm
Variouslysates were subjected to SDS PAGE followed by western blotwith 14255-1-AP (NCAM1/CD56 antibody) at dilution of 1:15000 incubated at room temperature for 1.5 hours.
https://www.ptglab.com/products/PI4K2A-Antibody-15318-1-AP.htm
HepG2 cells were subjected to SDS PAGE followed by western blotwith 15318-1-AP (PI4K2A antibody) at dilution of 1:800 incubated at room temperature for 1.5 hours.
https://www.abcam.com/products/primary-antibodies/pan-cadherin-antibody-epr1792y-intercellular-junction-marker-ab51034.html
https://www.abcam.com/products/primary-antibodies/gm130-antibody-ep892y-cis-golgi-marker-ab52649.html

For the ZDHHC20 antibody, this was verified by CRISPR/CAS9 KO disclosed in the manuscript.

Eukaryotic cell lines

Policy information about cell lines

Cell line source(s)	All cell lines used within this study were provided by The Francis Crick Institute Cell Services facility, namely HEK293T, HEK293-FT, MDA-MB-231, PANC1 and Flp-In™T-REx™ 293 cell lines.
Authentication	All the cell lines used in the publication were verified via STR by The Francis Crick Institute Cell Services.
Mycoplasma contamination	All cell lines were routinely screened for Mycoplasma by The Francis Crick Institute Cell Services facility, and tested negative.
Commonly misidentified lines (See ICLAC register)	No commonly misidentified cell lines were used in the study. All lines verified by STR analysis.
