# Supplementary material for: A palmitoyl transferase chemical–genetic system to map ZDHHC-specific S-acylation
Source: Nat Biotechnol. 2024 Jan 8;42(10):1548–58. doi: 10.1038/s41587-023-02030-0 (PMC11471619; doi:10.1038/s41587-023-02030-0)
Supplement: Supplementary file 4 — Uncropped images of gels and blots. [file 41587_2023_2030_MOESM4_ESM.pdf]

Appendix

A palmitoyl transferase chemical genetic system to map ZDHHC-specific *S*-acylation

Cory A. Ocasio[1,†], James Sipthorp[1,2,†], Marc P. Baggelaar[1,2,‡,†], Ana Losada de la Lastra[1,2], Manuel Tavares[2], Jana Volarić[2], Christelle Soudy[1], Elisabeth M. Storck[3], Jack W. Houghton[1], Susana A. Palma-Duran[1], James I. MacRae[1], Goran Tomic[1], Lotte Carr[1], Julian Downward[1], Ulrike S. Eggert[3] and Edward W. Tate[1,2,*]

Affiliations:

[1]The Francis Crick Institute; London, NW1 1AT (UK)

[2]Imperial College London; Department of Chemistry, Molecular Sciences Research Hub, London, W12 0BZ (UK)

[3]King's College London; Randall Centre for Cell and Molecular Biophysics, School of Basic and Medical Biosciences and Department of Chemistry, London, SE1 1UL (UK)

‡: Current address: Utrecht University; Biomolecular Mass Spectrometry & Proteomics Group, 3584 CH Utrecht (NL)

†: These authors contributed equally

Correspondence to: e.tate@imperial.ac.uk

Table of contents **Page**

1.	Uncropped gels, blots and replicates from Main Figures.	3 – 19
2.	Uncropped gels, blots and replicates from Extended Figures.	20 – 31

Appendix – Main Figures

Uncropped Gels and Blots

Main Figure 2C

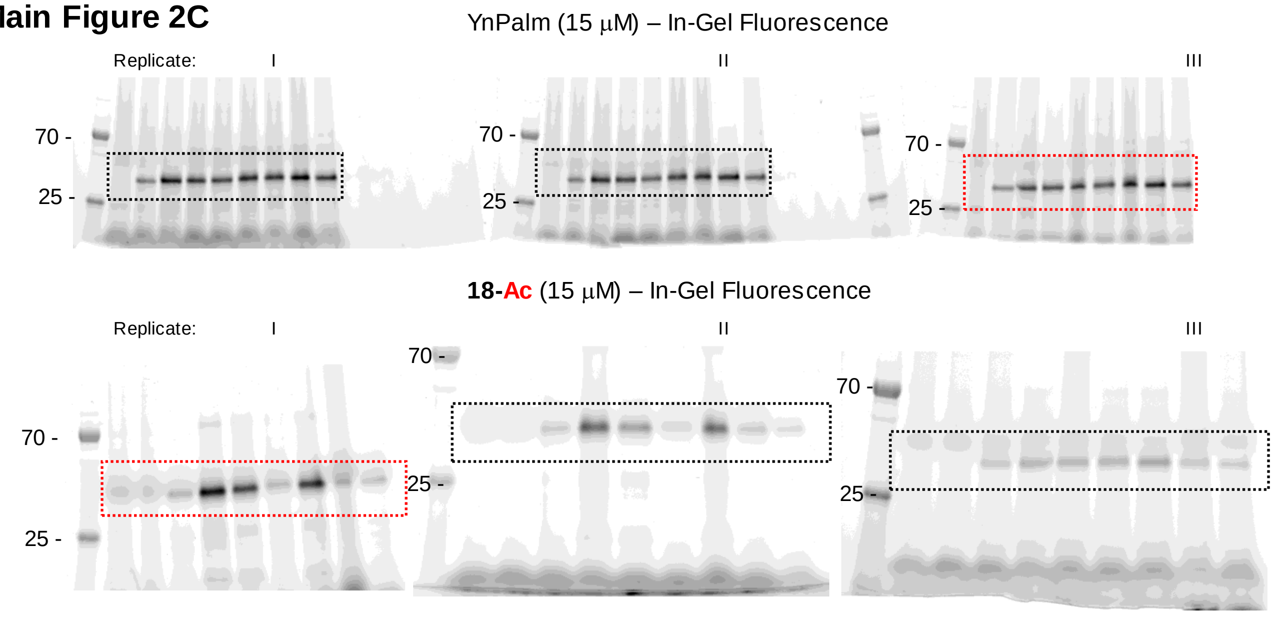

Main Figure 2E

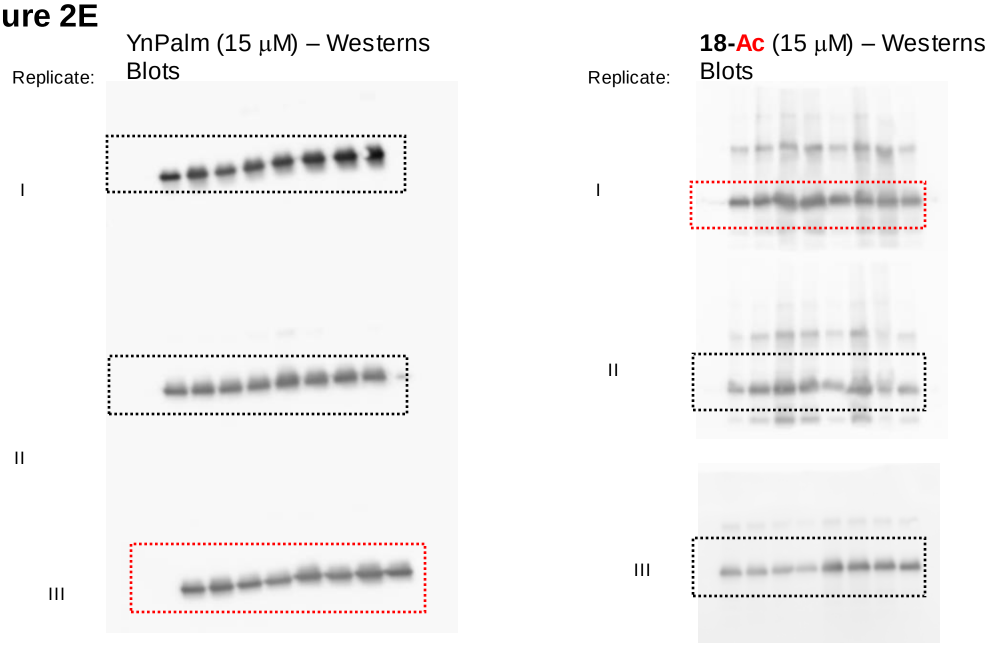

Figure A1. Uncropped gels and blots for Main Figure 2C,E. Areas within red borders are in the main figure

Main Figure 2G

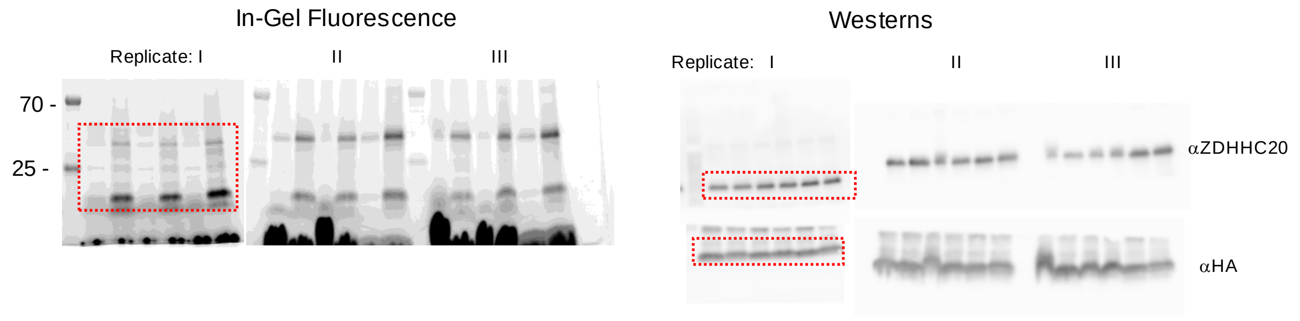

Figure A2. Uncropped gels and blots for Main Figure 2G. Areas within red borders are in the main figure

Main Figure 3F

C18Bz – Westerns Blots for ZDHHC(wt) and ZDHHC (M)

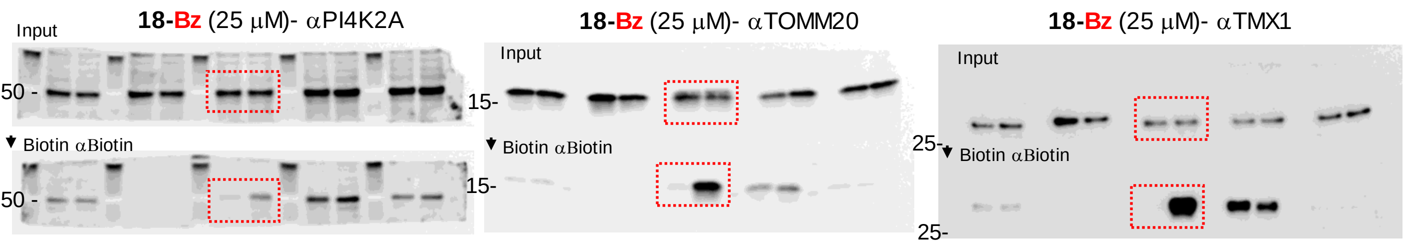

Main Figure 3G

YnPal – Westerns Blots for ZDHHC(wt) and ZDHHC (M)

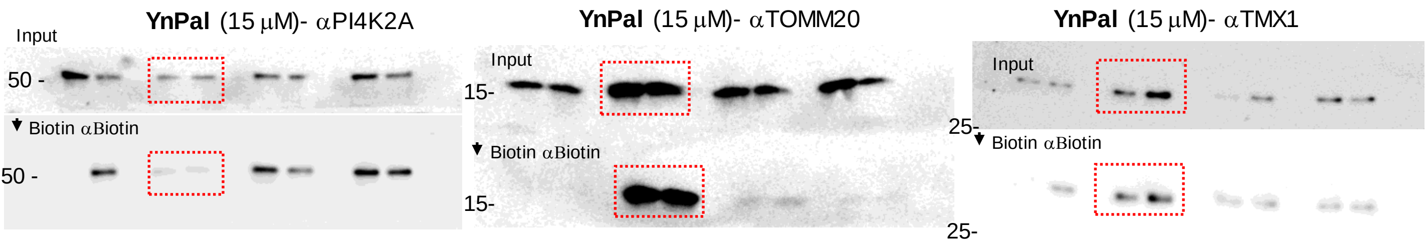

Figure A3. Uncropped gels and blots for Main Figure 3F and 3G. Areas within red borders are in the main figure

Main Figure 4A

IFITM3 Rep 1

IFITM3 Rep 1 TAMRA

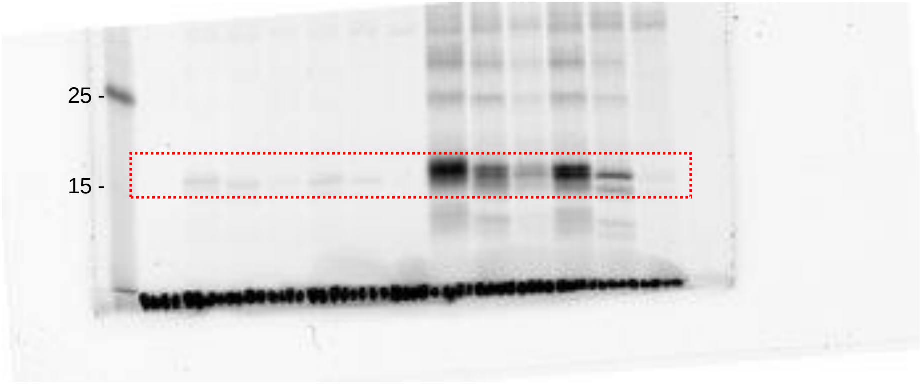

IFITM3 Rep 1 HA IP

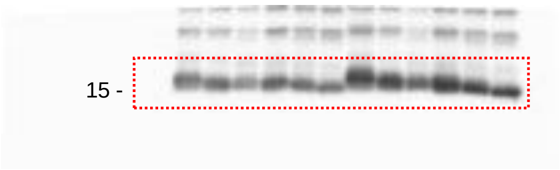

IFITM3 Rep 1 HA input

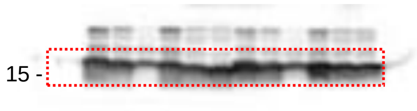

IFITM3 Rep 1 tubulin

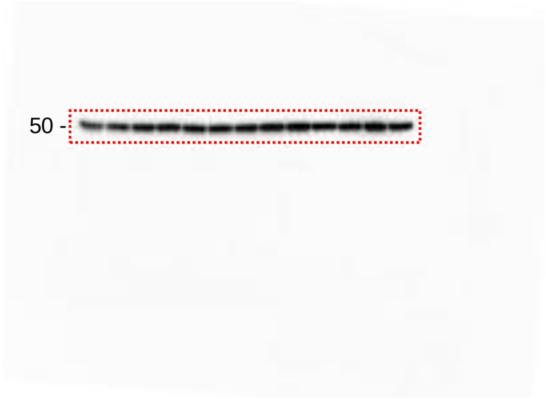

IFITM3 Rep 1 FLAG input

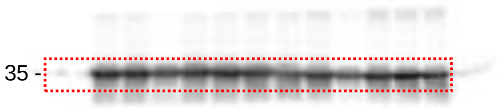

Figure A4. Uncropped gels and westerns for Main Figure 4A, replicate set 1. Areas within red borders are in the main figure.

Main Figure 4A

IFITM3 Rep 2

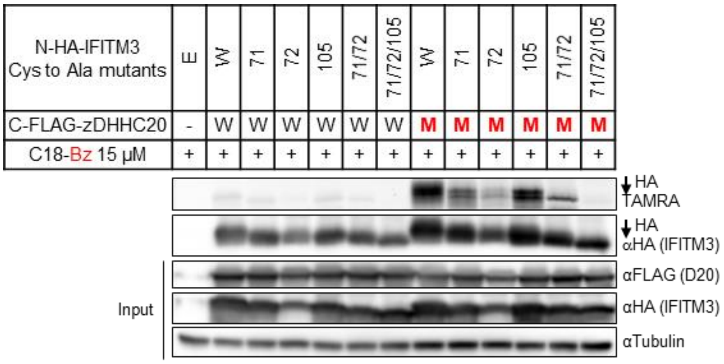

IFITM3 Rep 2 HA input

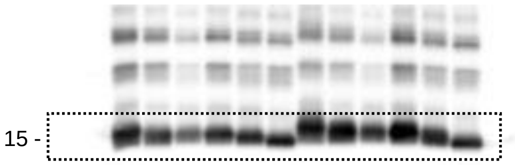

IFITM3 Rep 2 HA IP

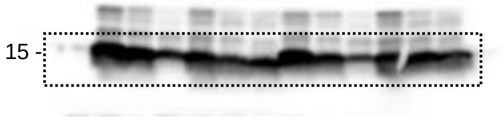

IFITM3 Rep 2 TAMRA

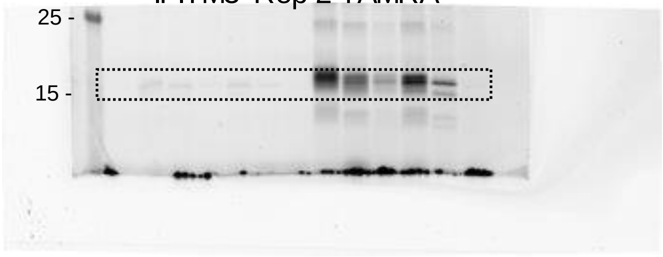

IFITM3 Rep 2 tubulin

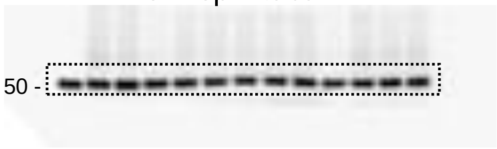

IFITM3 Rep 2 FLAG input

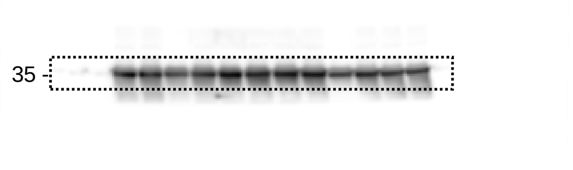

Figure A5. Uncropped gels and westerns for Main Figure 4A, replicate sets 2 – 3. Areas within red borders are in the main figure.

Main Figure 4A

IFITM3 Rep 3

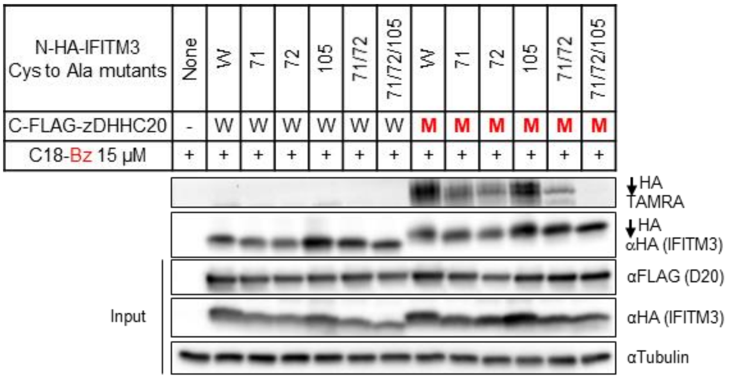

IFITM3 Rep 3 HA IP

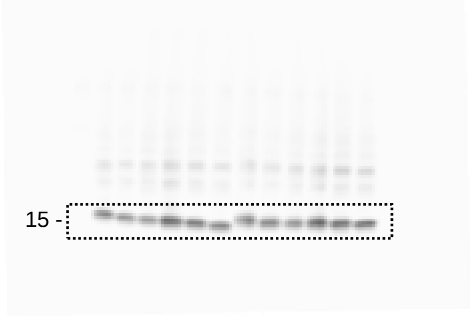

IFITM3 Rep 3 TAMRA

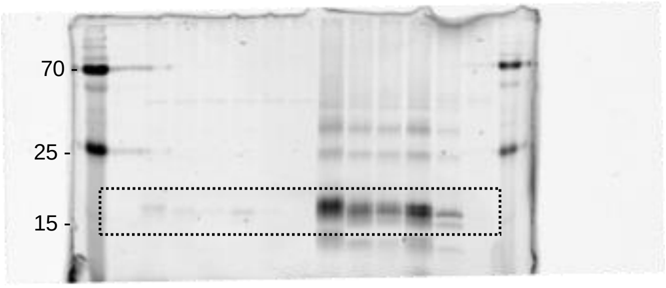

IFITM3 Rep 3 FLAG input

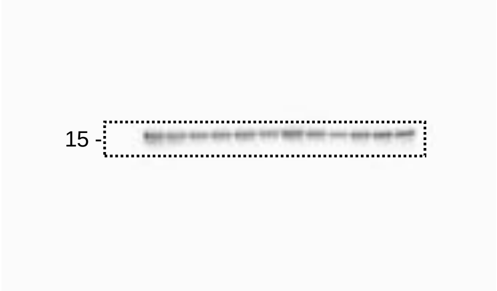

IFITM3 Rep 3 HA input

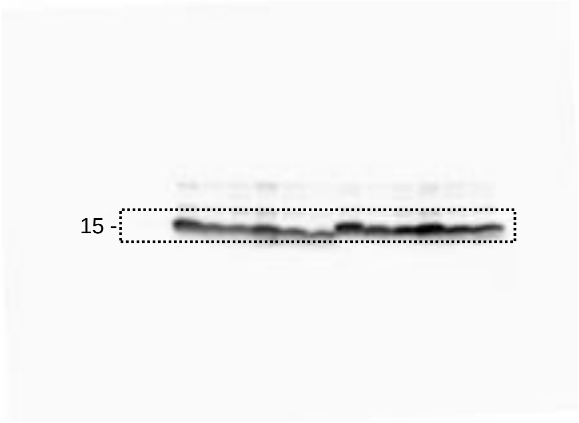

IFITM3 Rep 1 tubulin

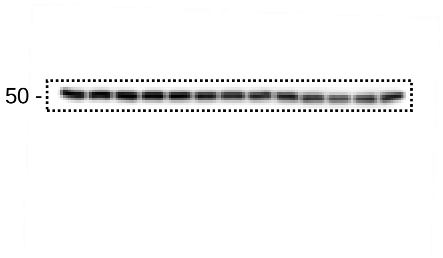

Figure A6 cont. Uncropped gels and westerns for Main Figure 4A, replicate sets 2 – 3. Areas within red borders are in the main figure.

Main Figure 4B

PI4K2A Rep 1

PI4K2A Rep 1 TAMRA

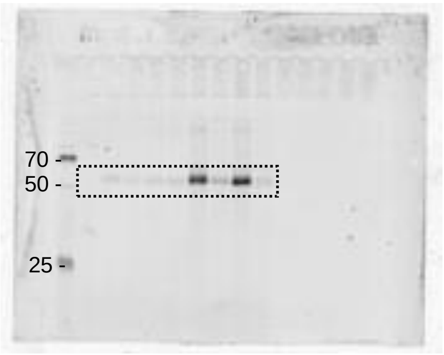

PI4K2A Rep 1 Tubulin input

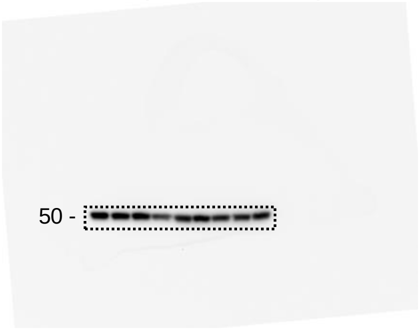

PI4K2A Rep 1 HA IP

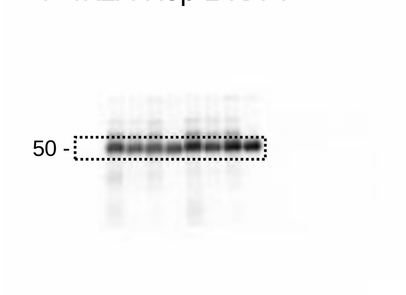

PI4K2A Rep 1 FLAG input

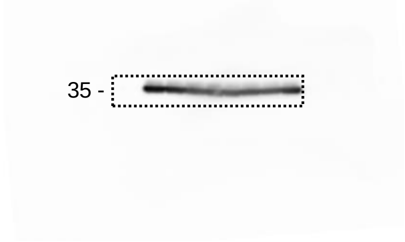

PI4K2A Rep 1 HA input

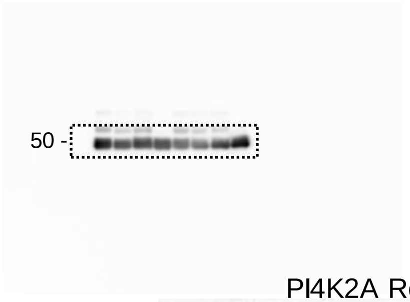

PI4K2A Rep 2

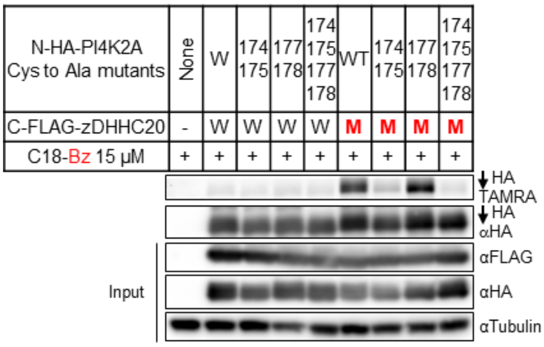

PI4K2A Rep 2 TAMRA

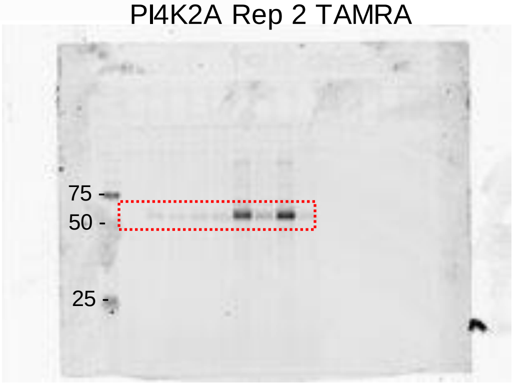

PI4K2A Rep 2 HA input

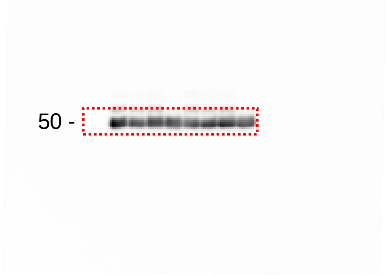

PI4K2A Rep 2 HA IP

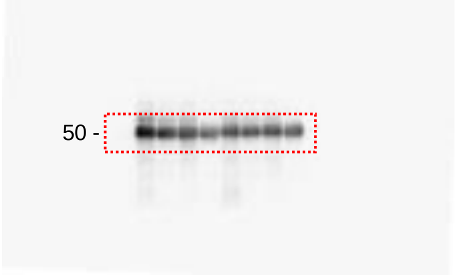

PI4K2A Rep 2 FLAG input

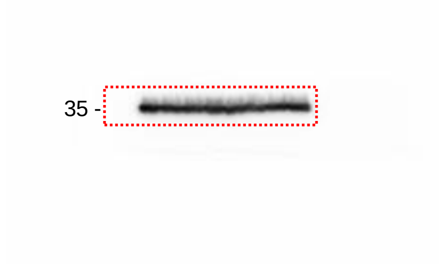

PI4K2A Rep 2 Tubulin input

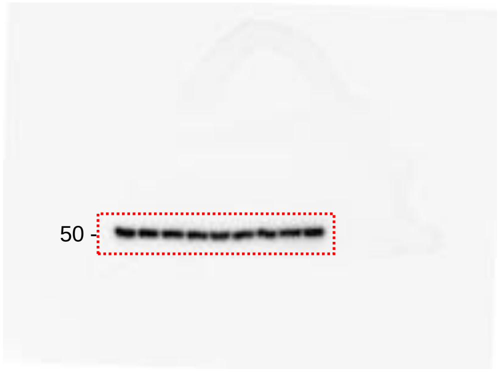

Figure A7. Uncropped gels and westerns for Main Figure 4B, replicate sets 1 – 2. Areas within red borders are in the main figure.

Main Figure 4B

PI4K2A Rep 3

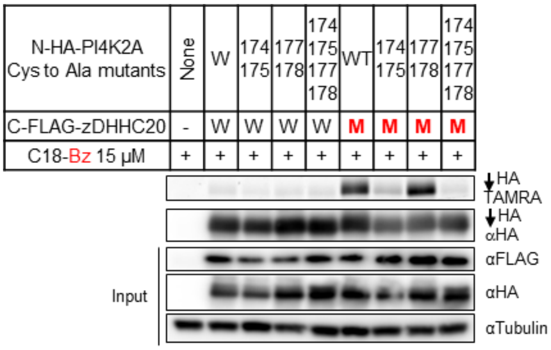

PI4K2A Rep 3 TAMRA

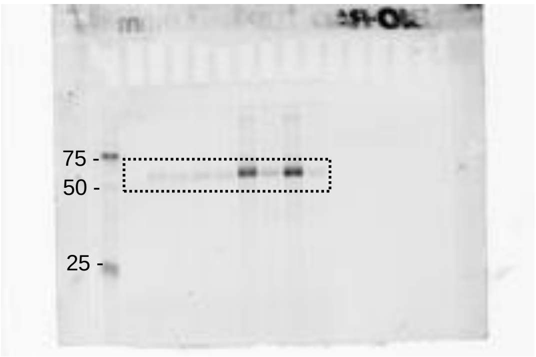

PI4K2A Rep 3 HA input

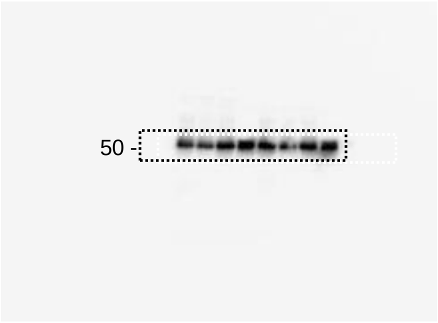

PI4K2A Rep 3 FLAG input

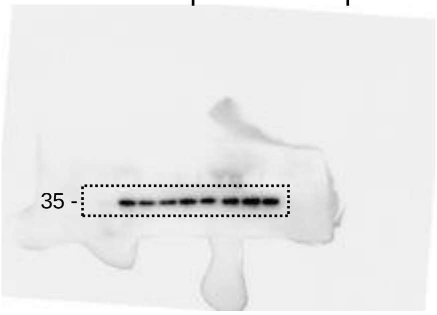

PI4K2A Rep 3 tubulin input

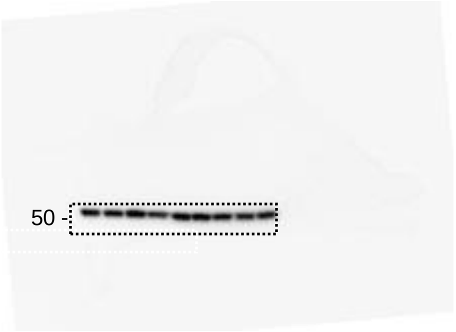

PI4K2A Rep 3 HA IP

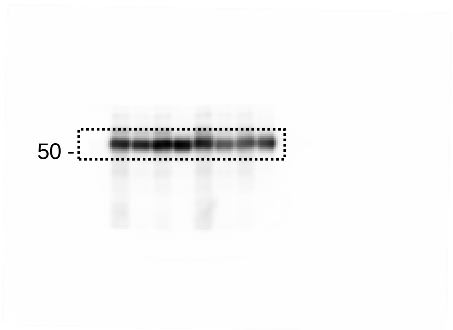

Figure A8. Uncropped gels and westerns for Main Figure 4B, replicate set 3. Areas within red borders are in the main figure.

Main Figure 4C

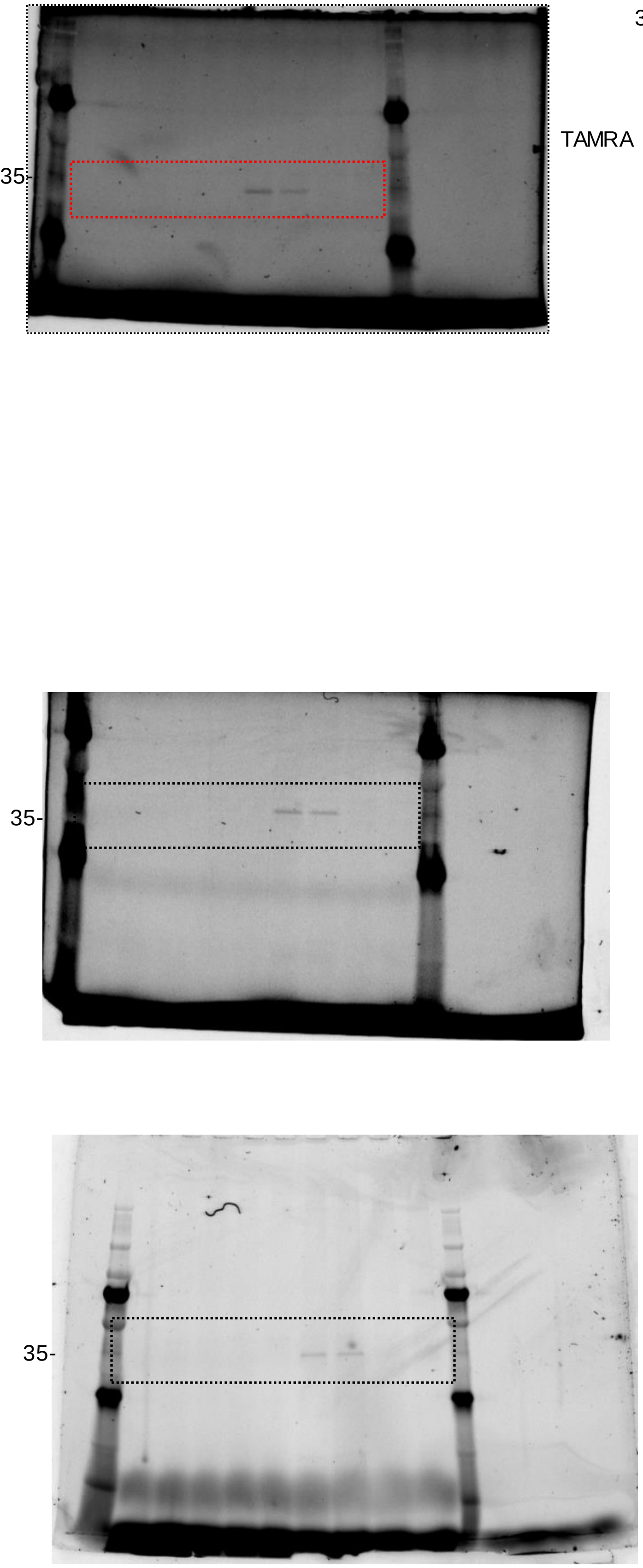

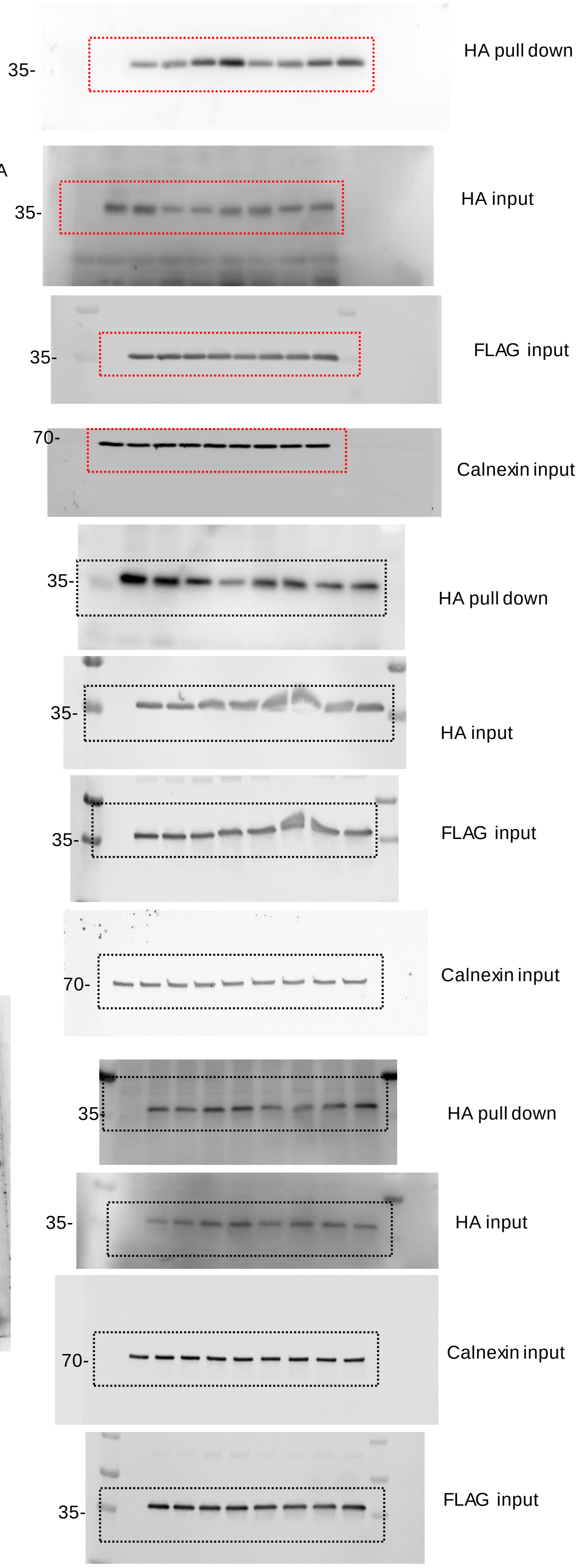

Figure A9.Uncropped gels and westerns for Main Figure 4c. Areas within red borders are in the main figure.

Main Figure 4D

VAMP 3 HA

Rep1 Rep2 Rep3

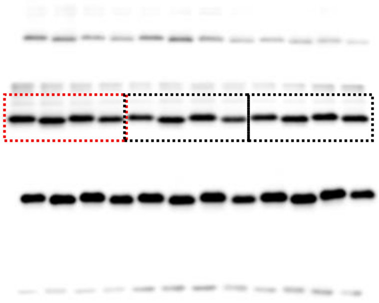

VAMP 3 TAMRA

Rep1 Rep2 Rep3

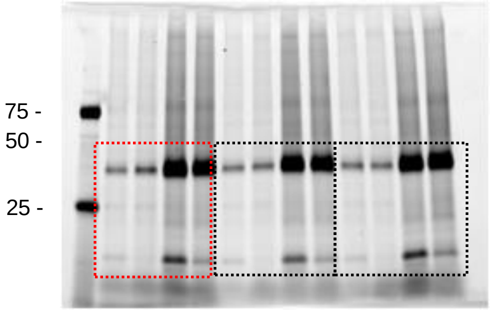

VAMP 3 Vinculin

Rep1 Rep2 Rep3

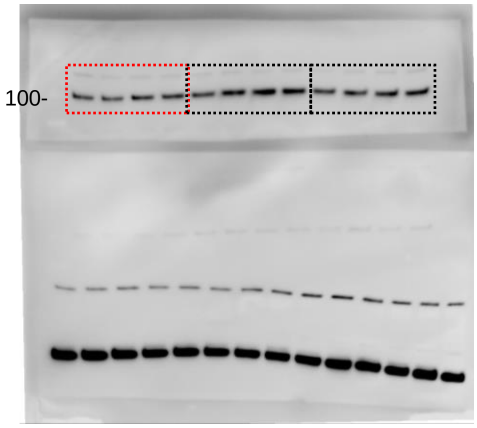

VAMP 3 FLAG

Rep1 Rep2 Rep3

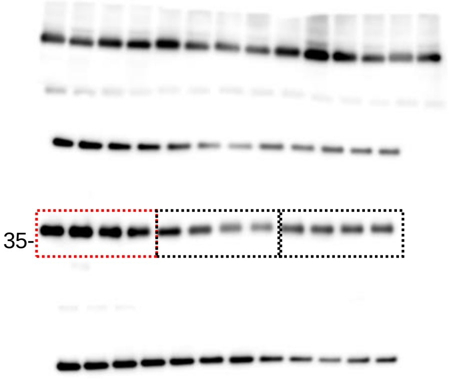

Figure A10. Uncropped gels and westerns for Main Figure 4D. Areas within red borders are in the main figure.

Main Figure 4E

BCAP31 – Replicates 1 - 3

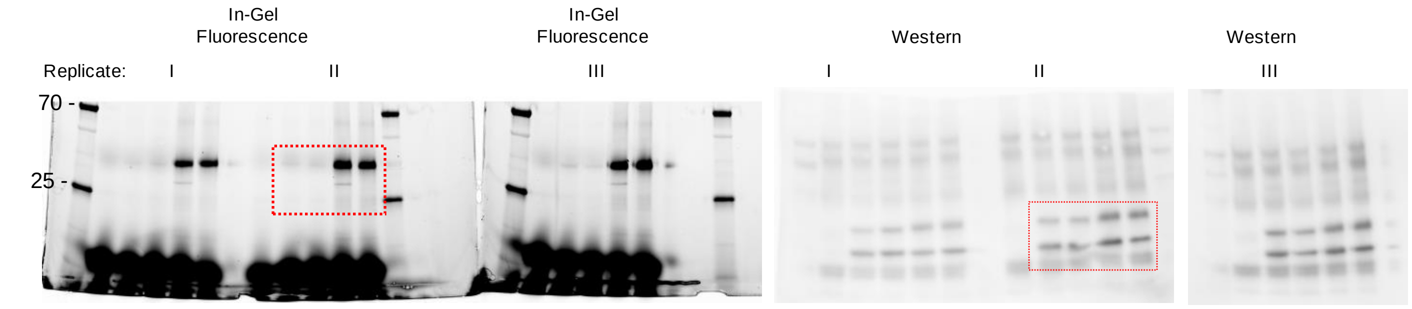

Figure A11. Uncropped gels and westerns for Main Figure 4E. Areas within red borders are in the main figure.

Main Figure 4F

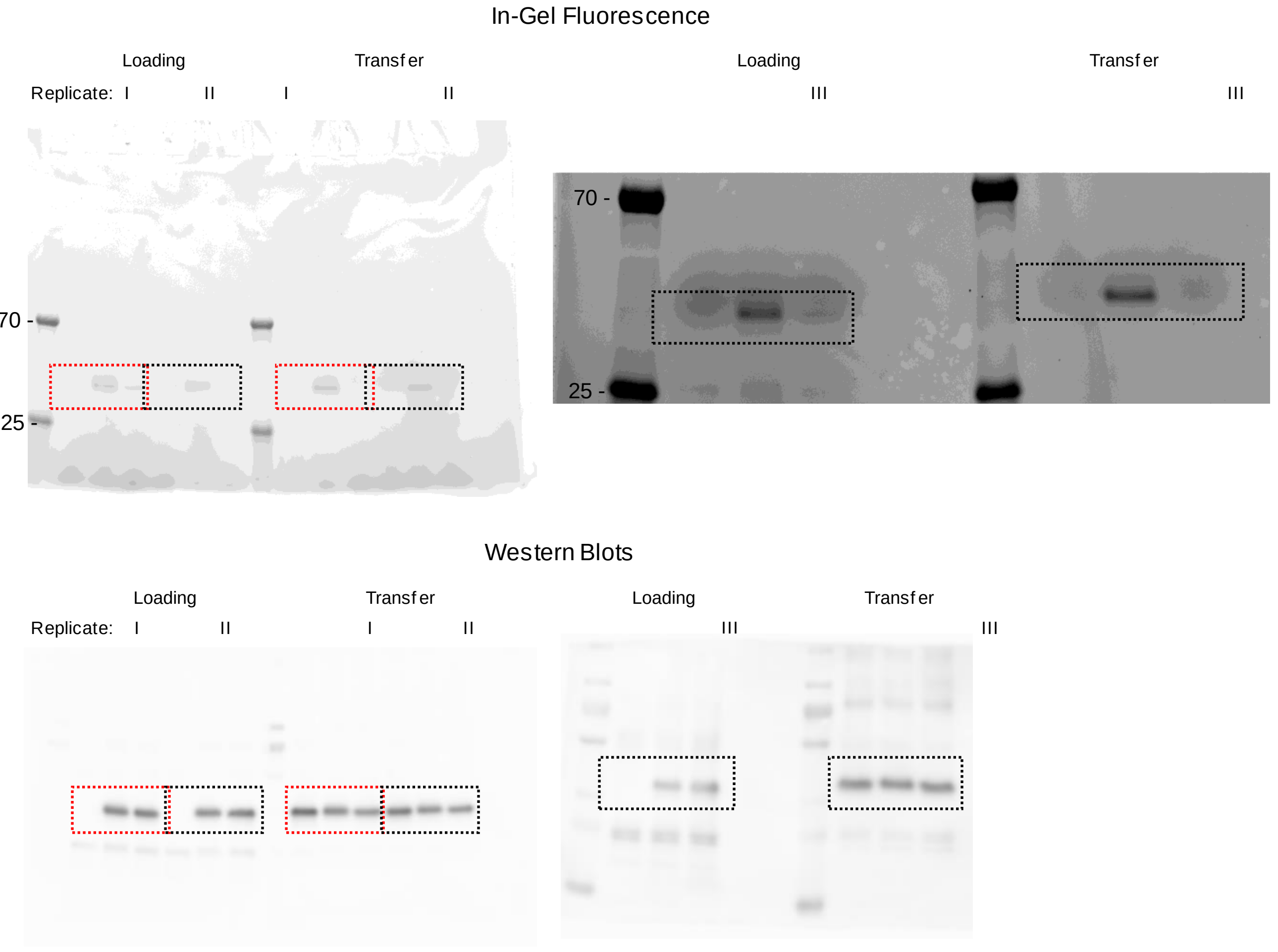

Figure A12. Uncropped gels and westerns for Main Figure 4F, replicate sets 1 - 3. Areas within red borders are in the main figure.

Main Figure 5A

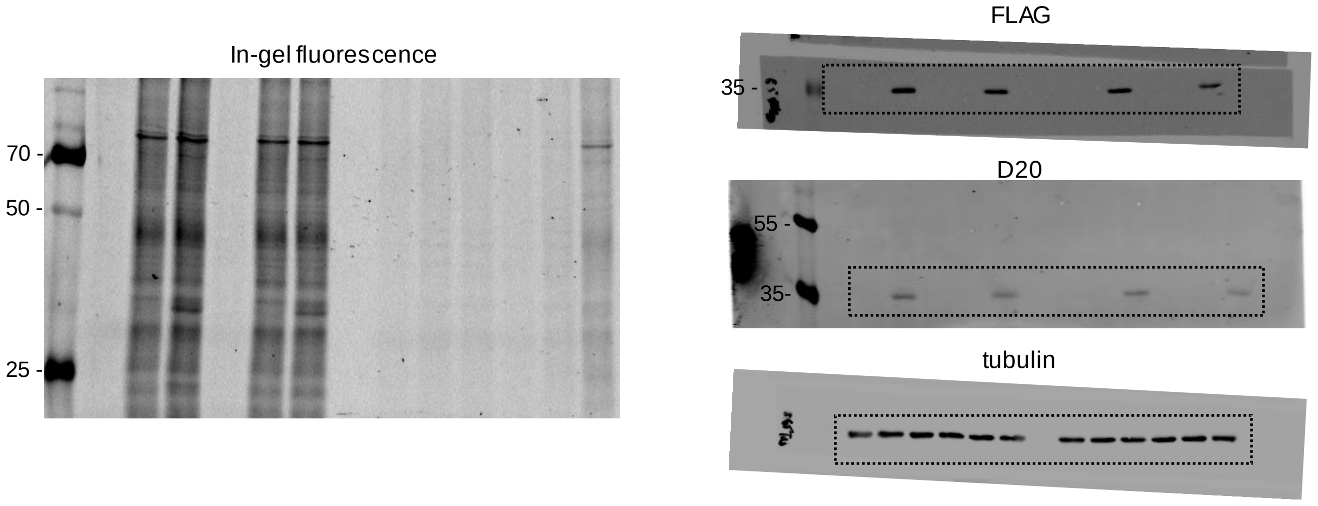

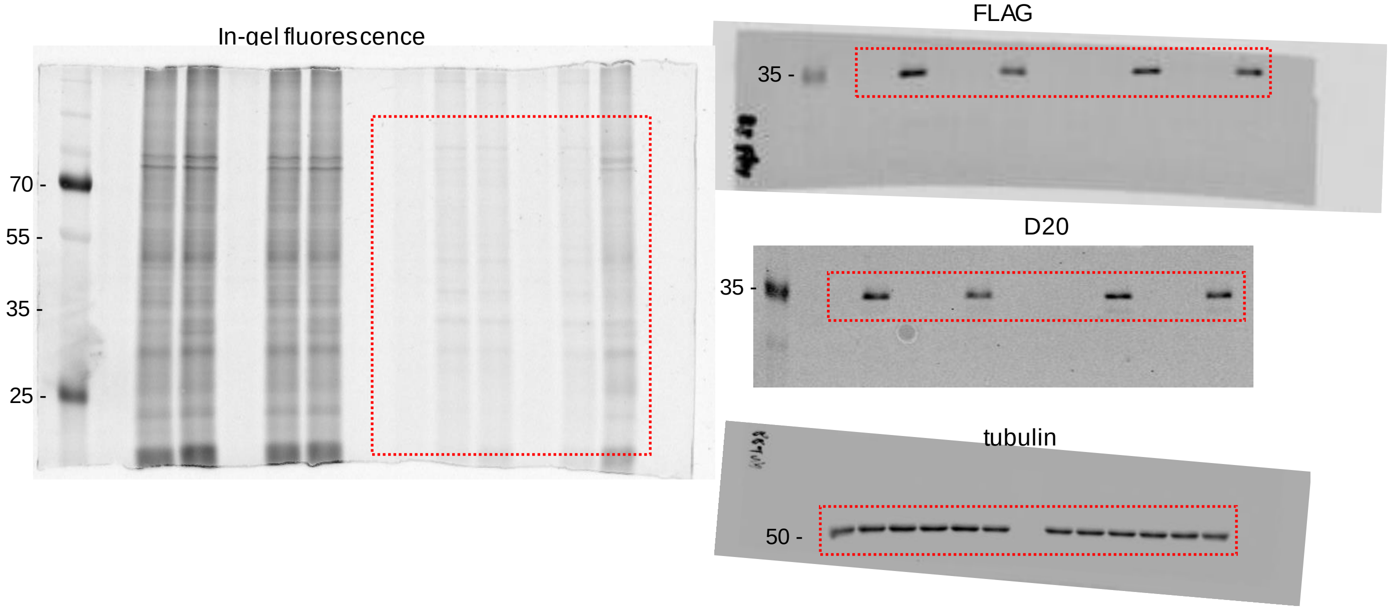

Figure A13. Uncropped gels and westerns for Main Figure 5F. Areas within red borders are in the main figure.

Main Figure 5B rep1

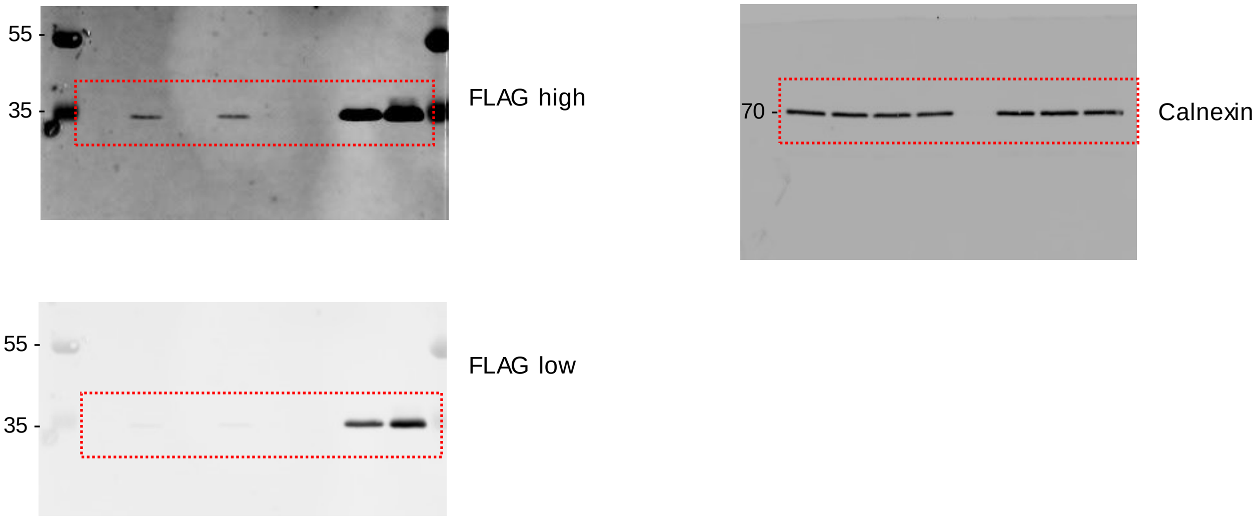

Main Figure 5B rep2

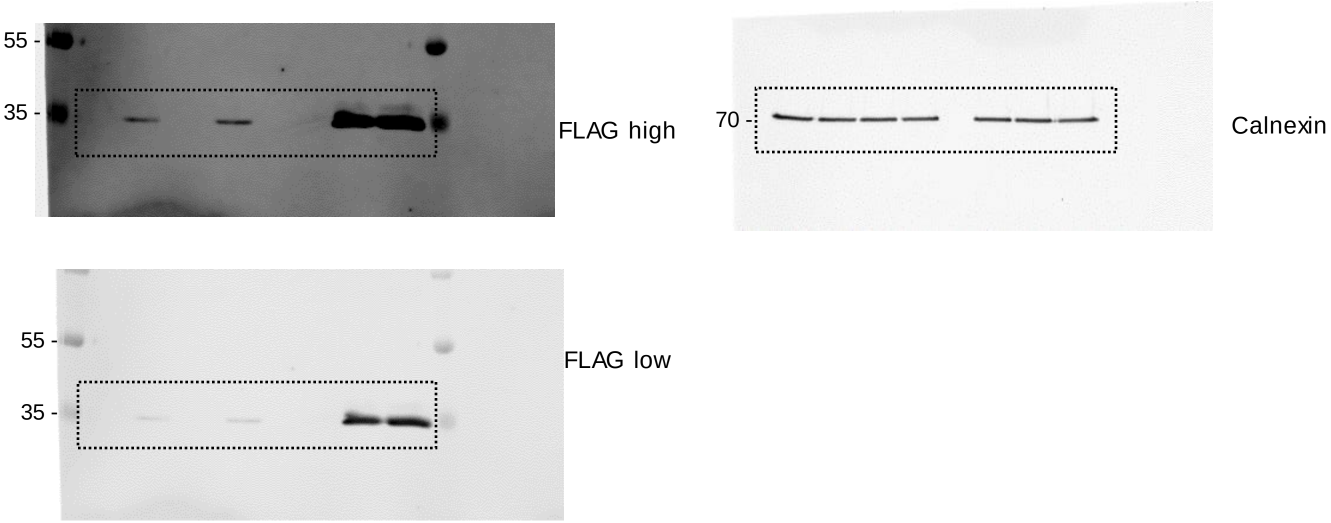

Main Figure 5B rep3

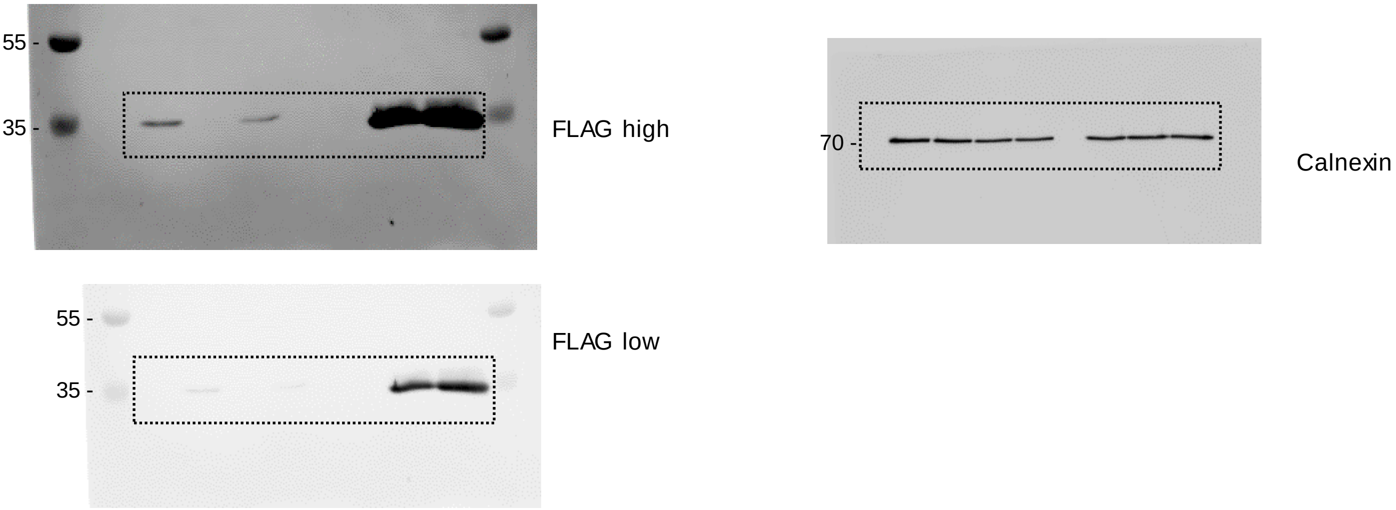

Figure A14. Uncropped gels and westerns for Main Figure 5B. Areas within red borders are in the main figure.

Main Figure 5C Replicate 1

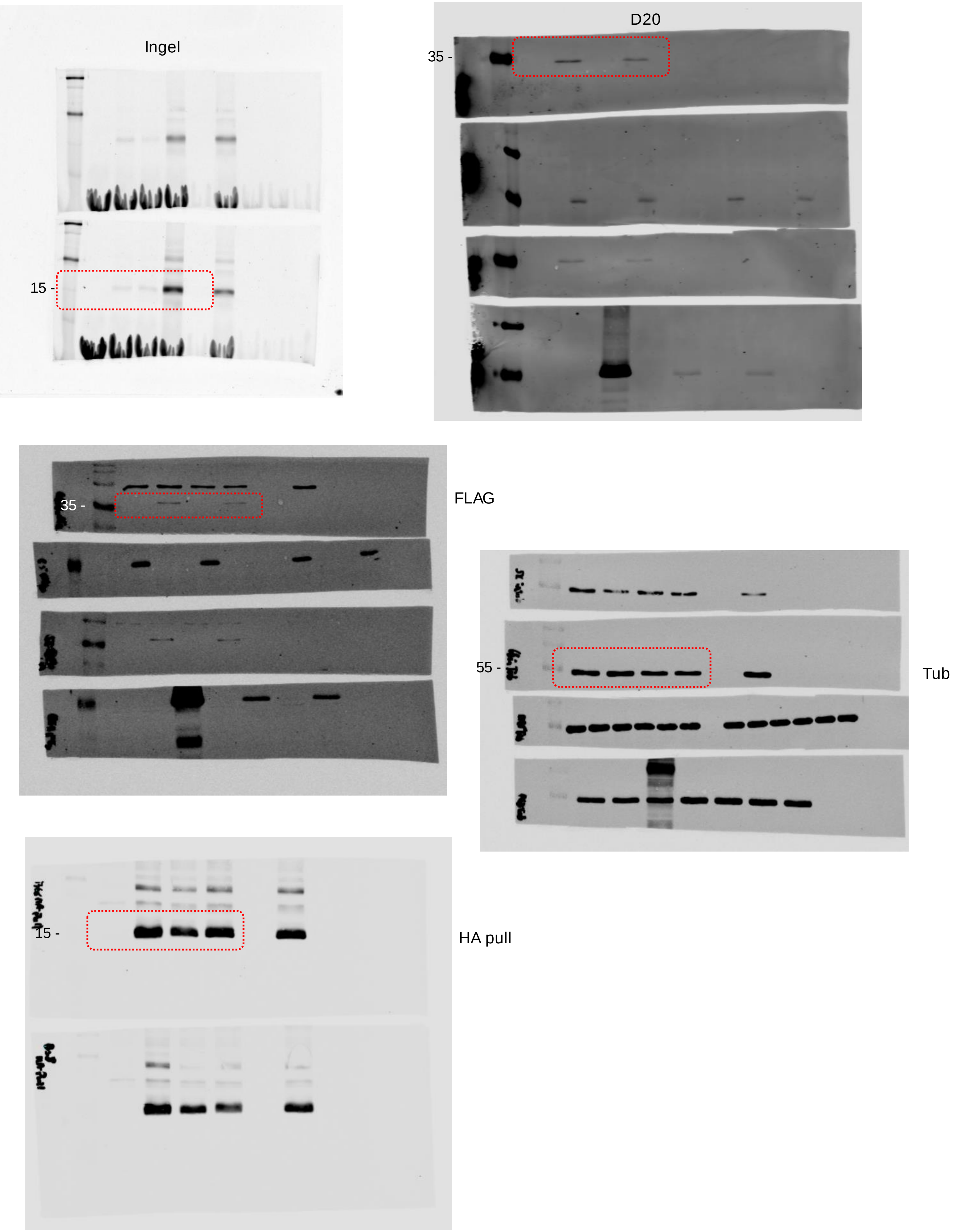

Figure A15. Uncropped gels and westerns for Main Figure 5C. Areas within red borders are in the main figure.

Main Figure 5C

Replicate 2

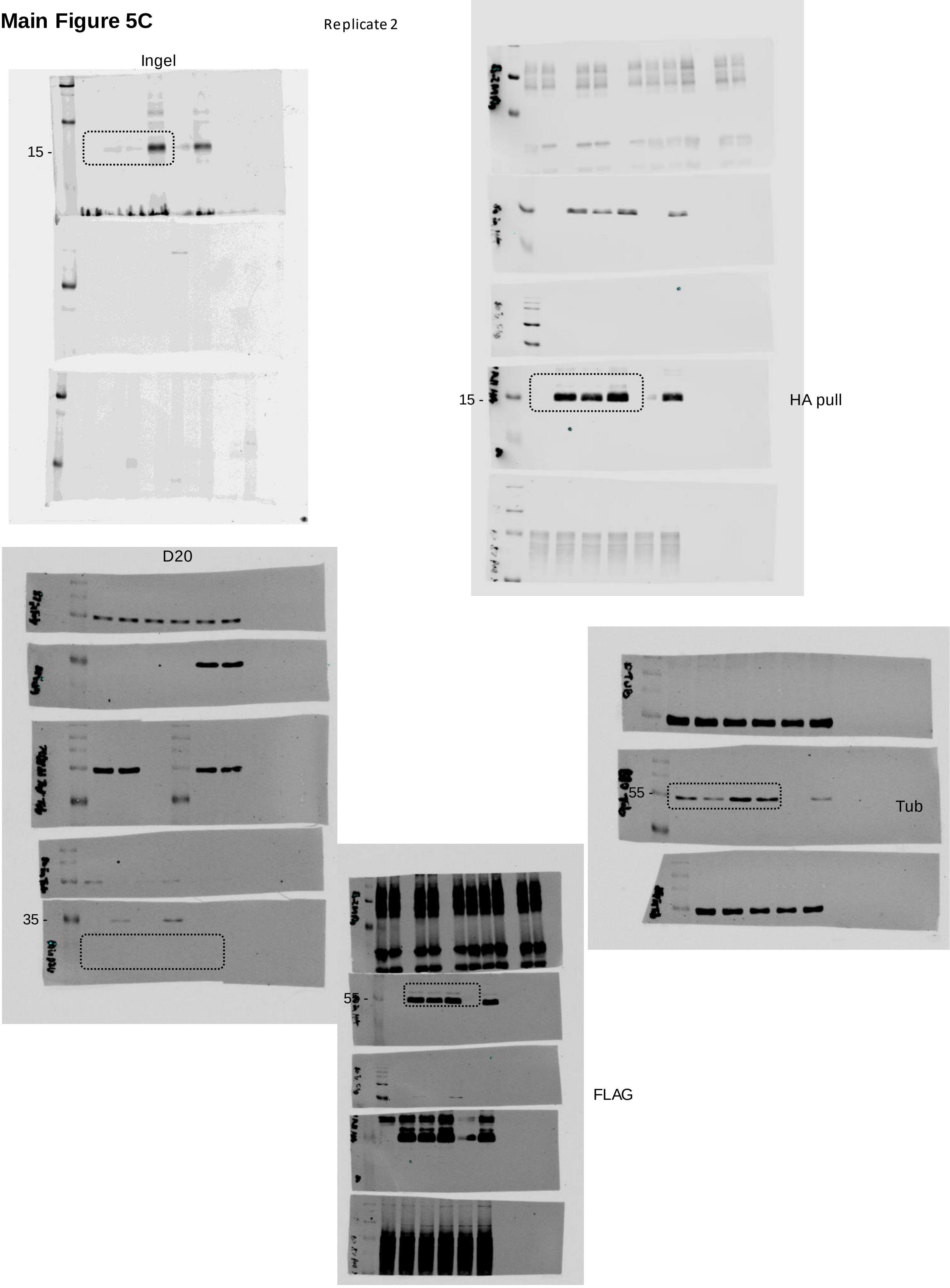

Figure A16. Uncropped gels and westerns for Main Figure 5C. Areas within red borders are in the main figure.

Main Figure 5C

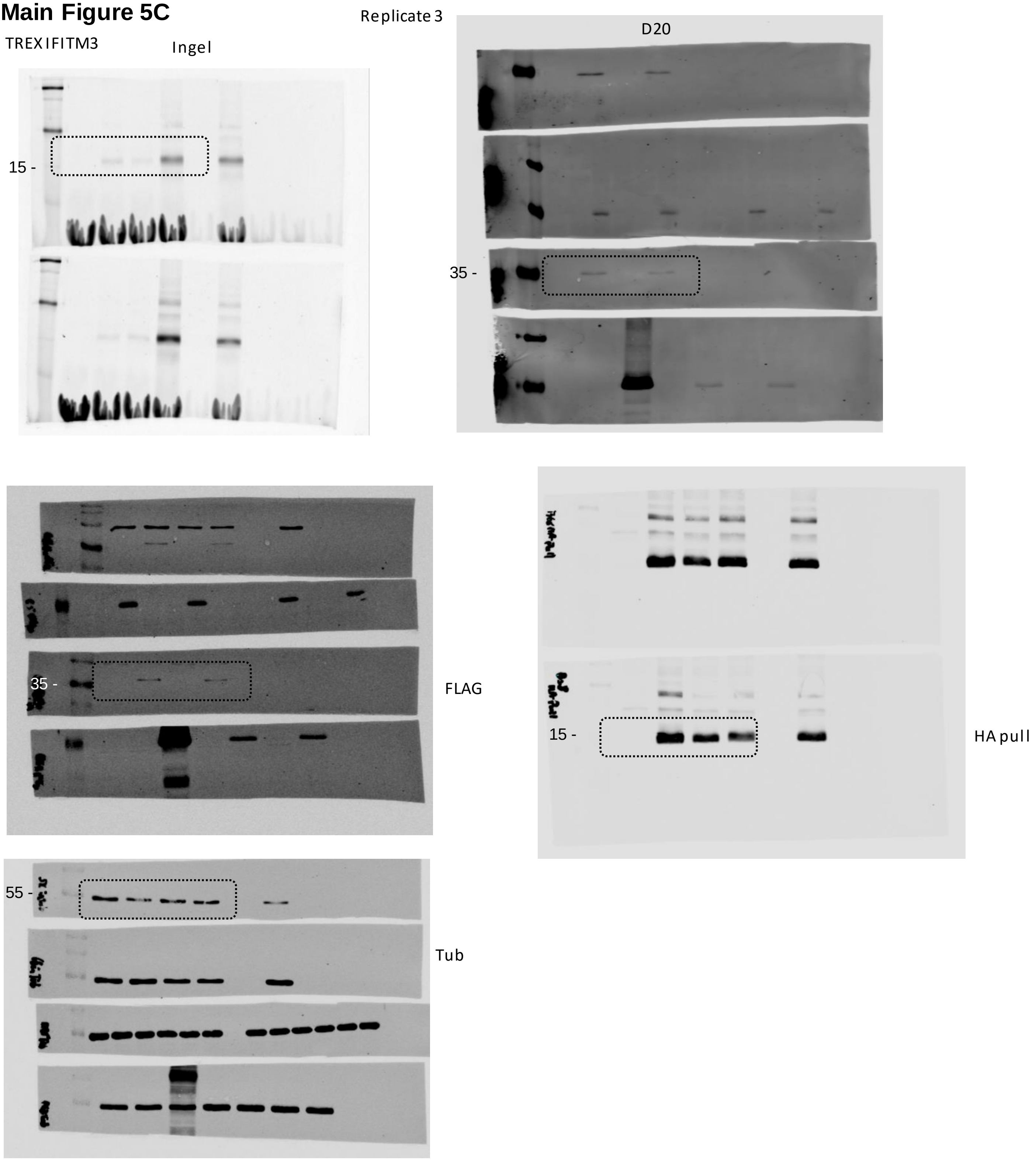

Figure A17. Uncropped gels and westerns for Main Figure 5C. Areas within red borders are in the main figure.

Main Figure 5E

C18Bz – Westerns Blots for Trex ZDHHC(wt) and ZDHHC (M)

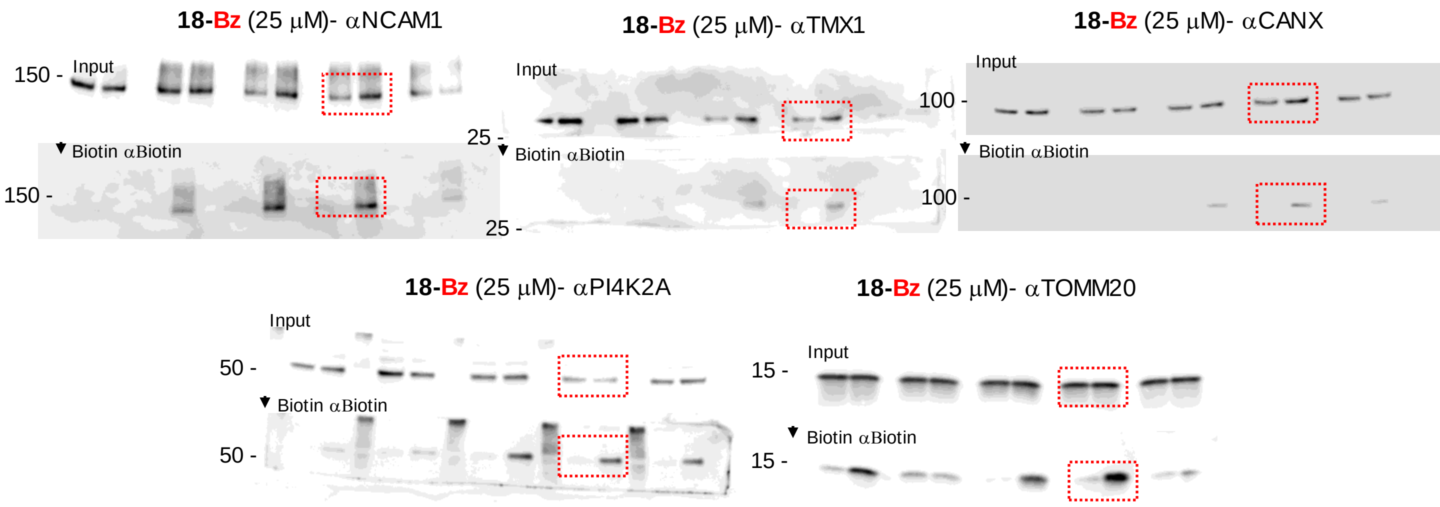

Main Figure 5F

YnPal – Westerns Blots for for Trex ZDHHC(wt) and ZDHHC (M)

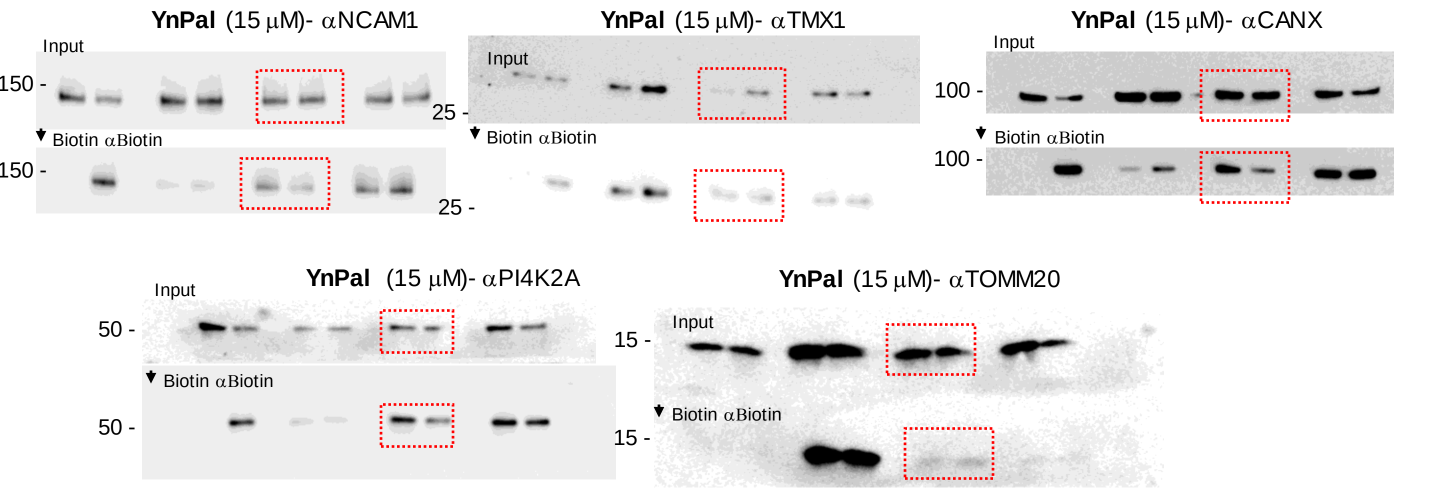

Figure A18. Uncropped gels and westerns for Main Figure 5E and 5F. Areas within red borders are in the main figure.

Main Figure 6B

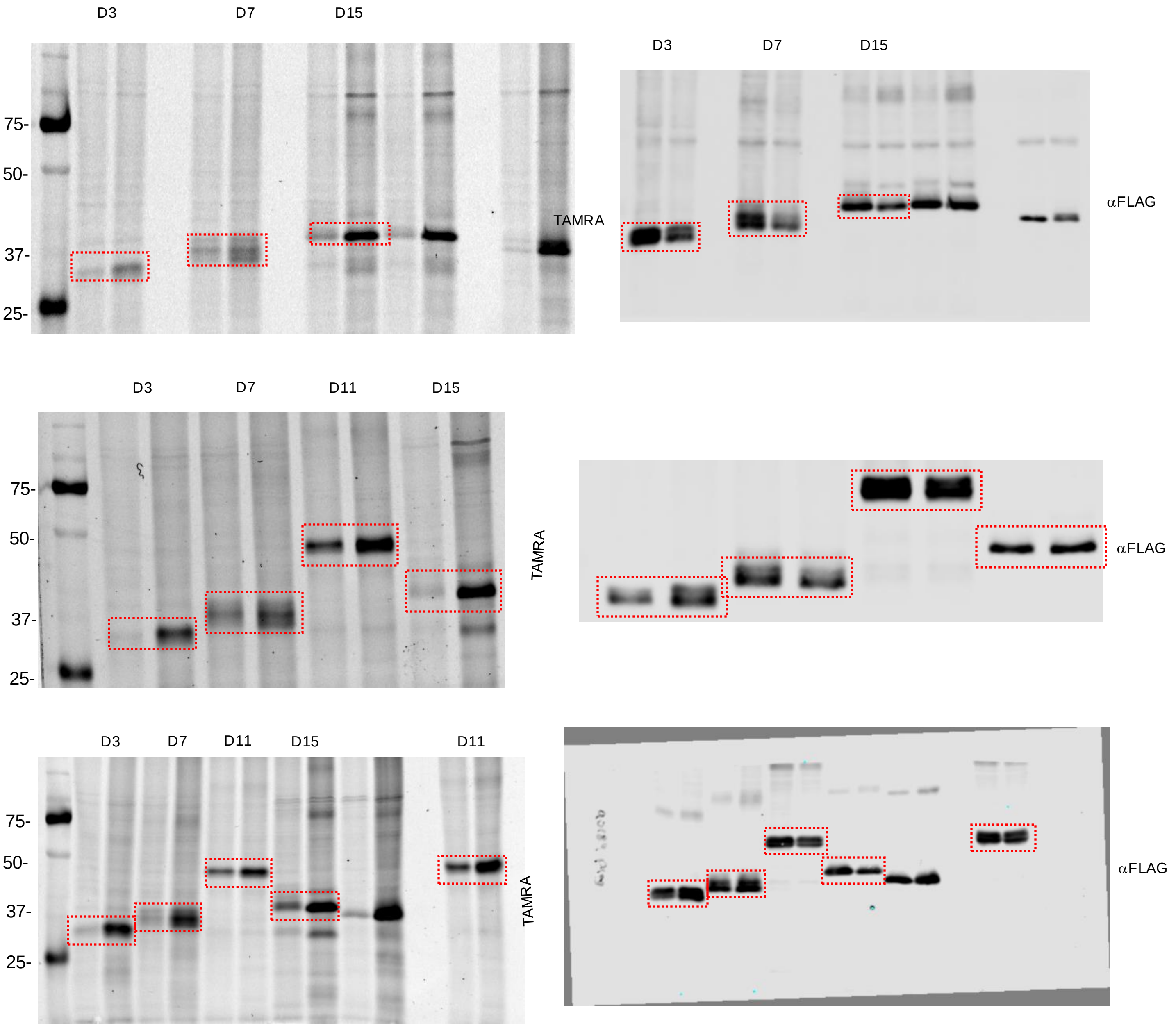

Figure A19. Uncropped gels and westerns for Main Figure 6B. Areas within red borders are in the supplementary figure.

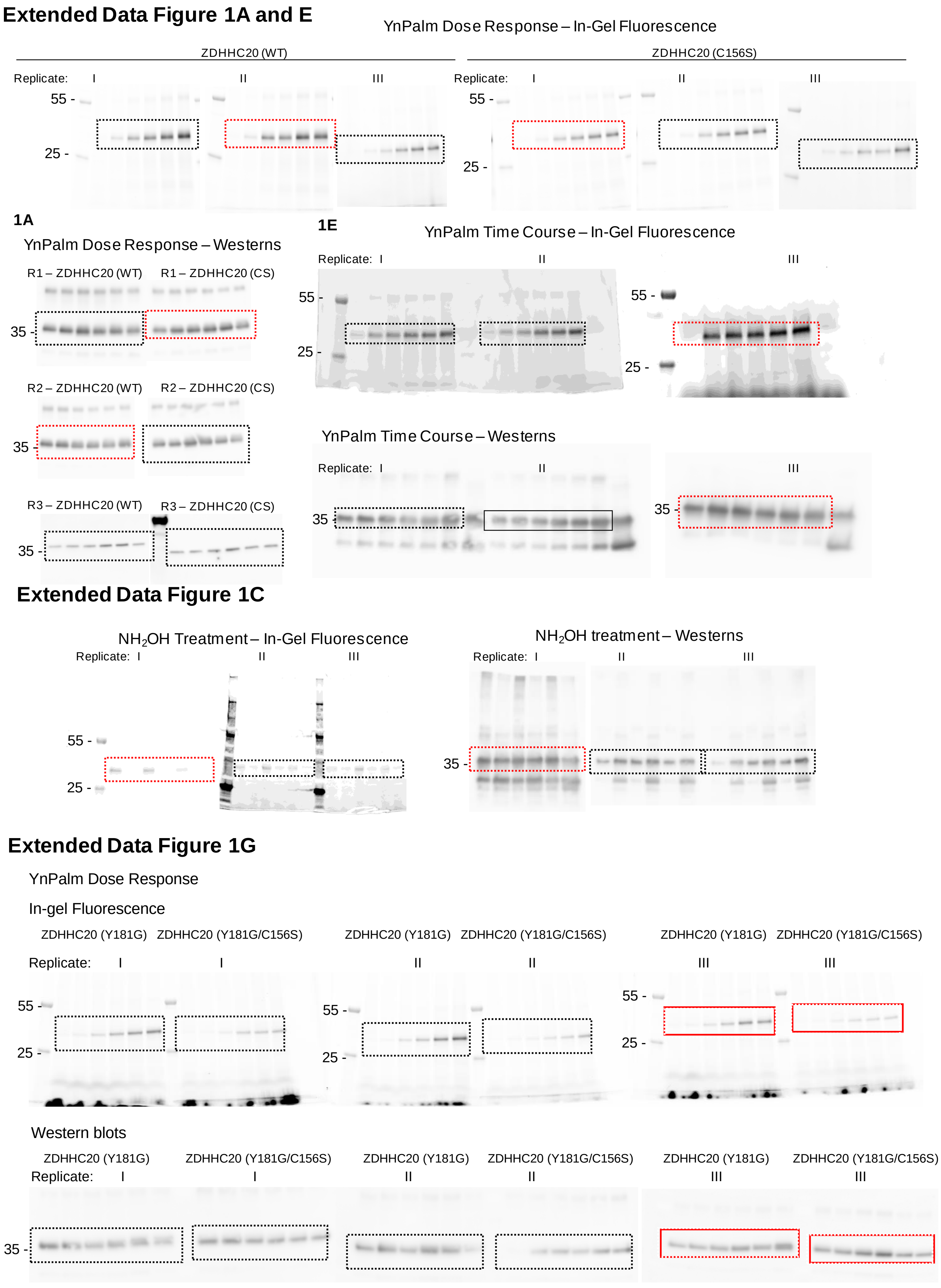

Figure A20. Uncropped gels and blots for Extended Data Figure 1A,C,E,G. Areas within red borders are in the supplementary figure.

Extended Data Figure 1I and K

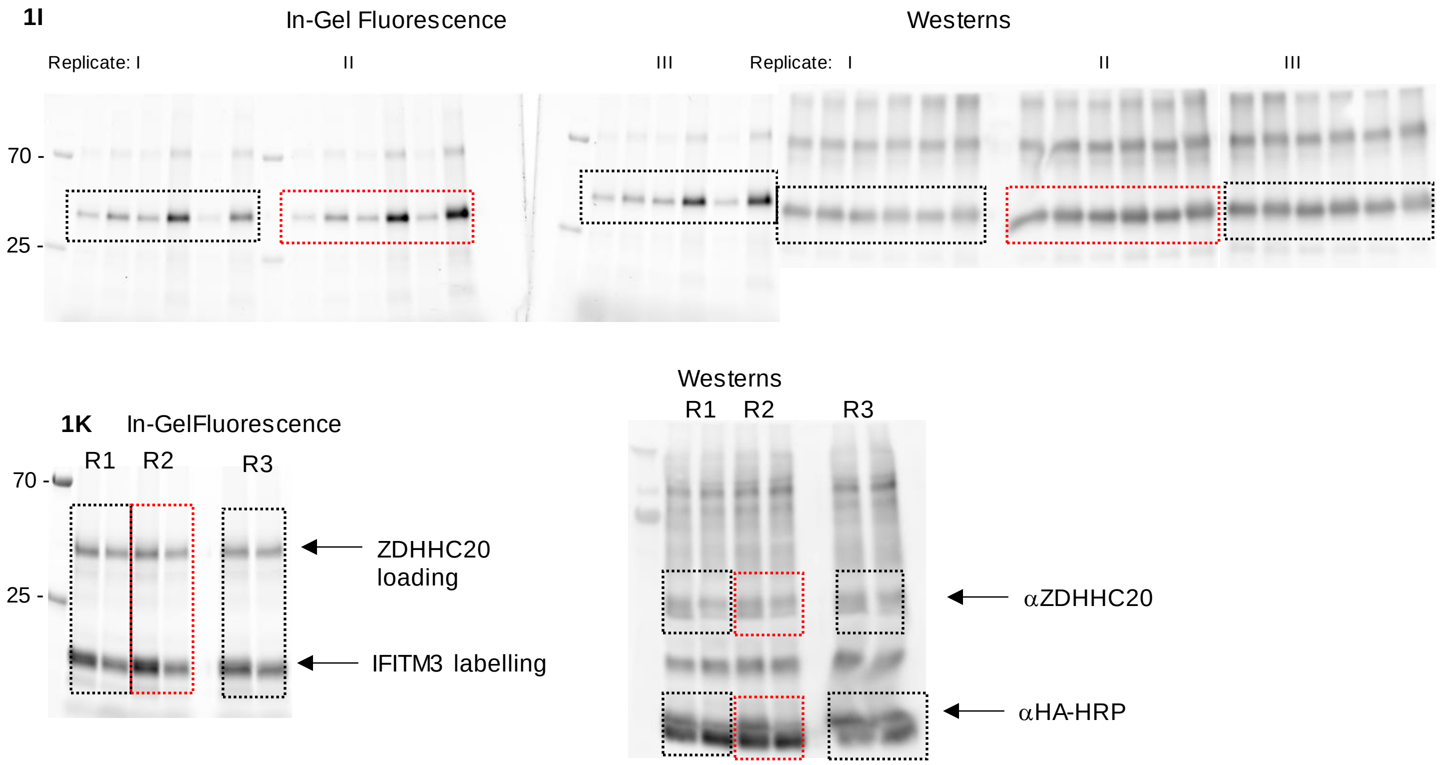

Figure A21. Uncropped gels and blots for Extended Data Figure 1I,K. Areas within red borders are in the supplementary figure.

Extended Data Figure 2A

Replicate 1

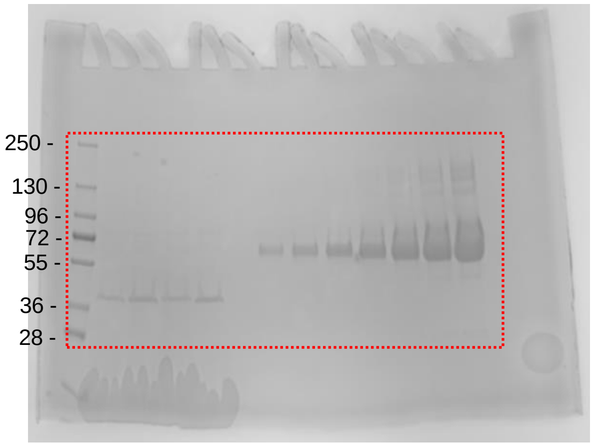

Replicate 3

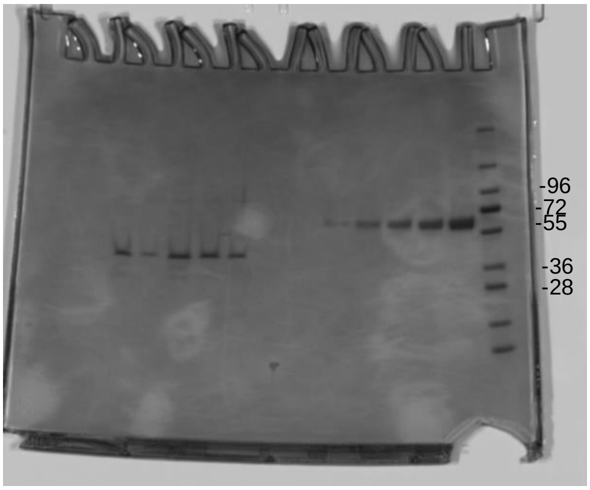

Figure A22. Uncropped gels and blots for Extended Data Figure 2A. Areas within red borders are in the supplementary figure.

Extended Data Figure 3A

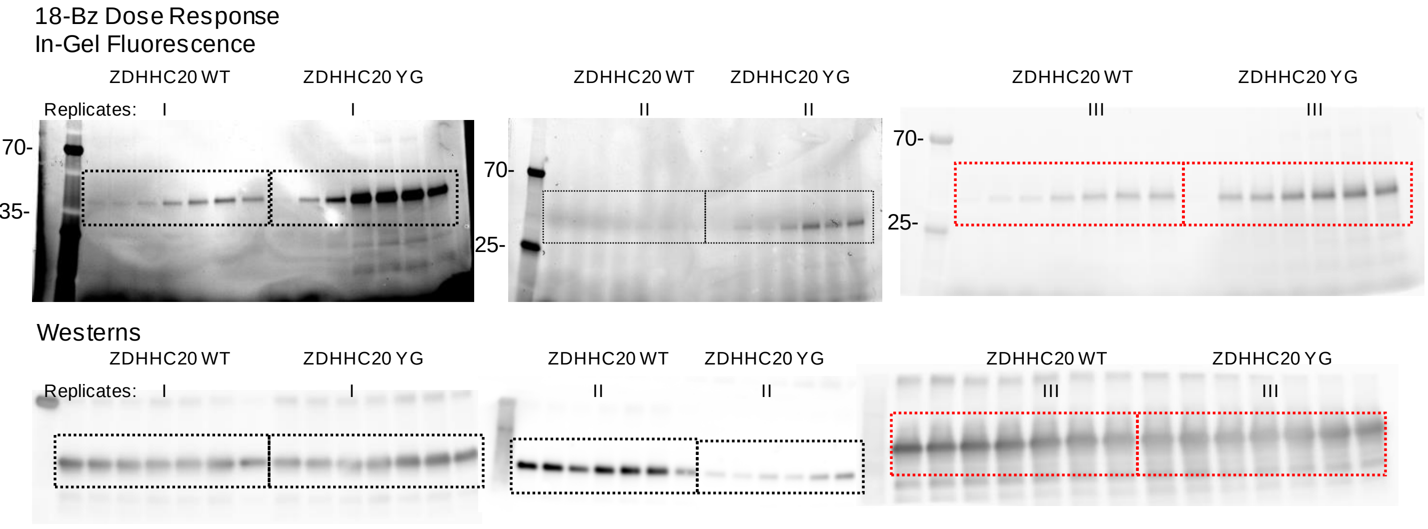

Figure A23. Uncropped gels and westerns for Supplementary Figure 3A. Areas within red borders are in the supplementary figure.

Extended Data Figure 3C

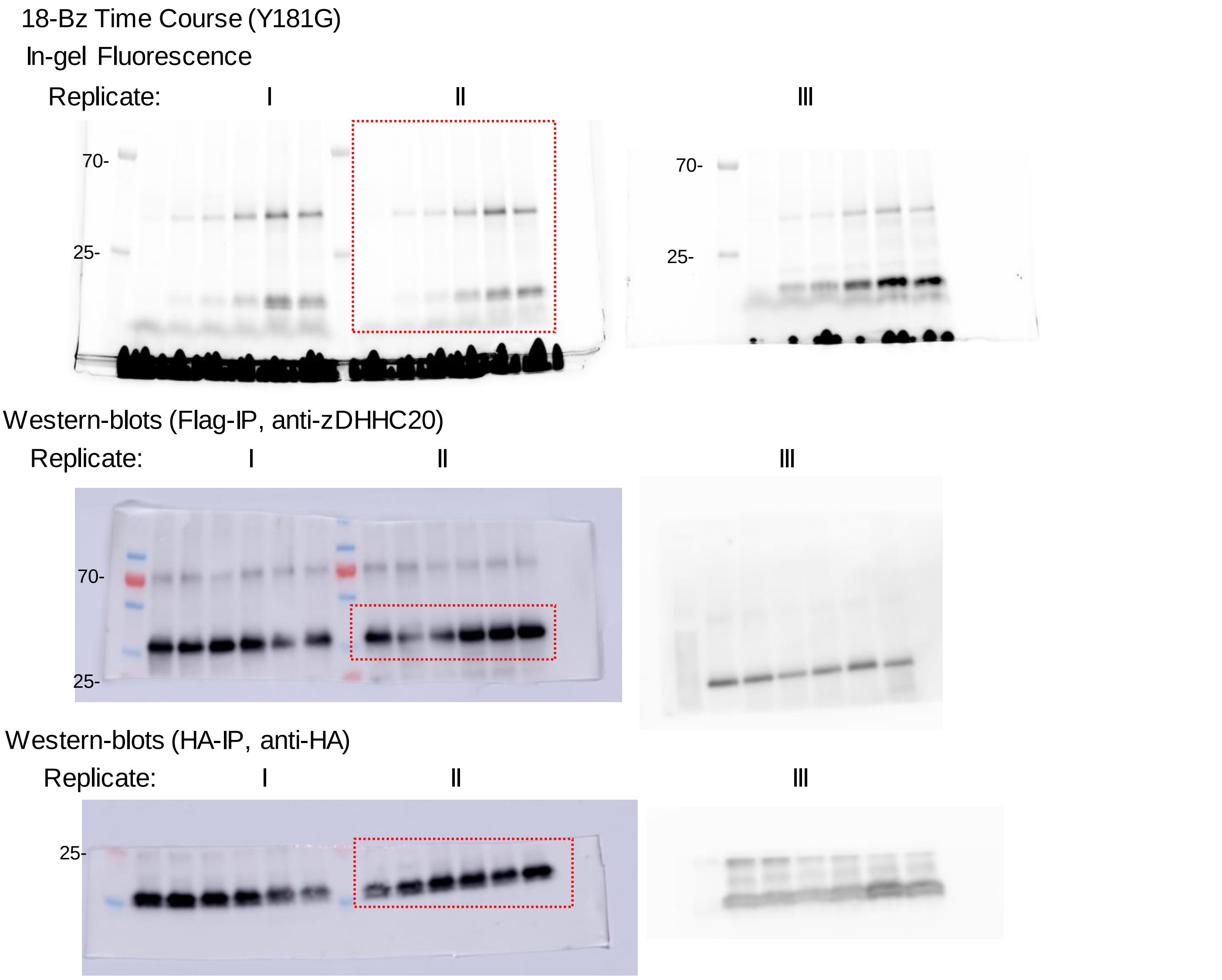

Figure A24. Uncropped gels and westerns for Supplementary Figure 3C. Areas within red borders are in the supplementary figure. Replicates I and II for HA-IP and Flag-IP are overlays of the chemiluminescence scan and the white light image.

Extended Data Figure 6A

HEK293T

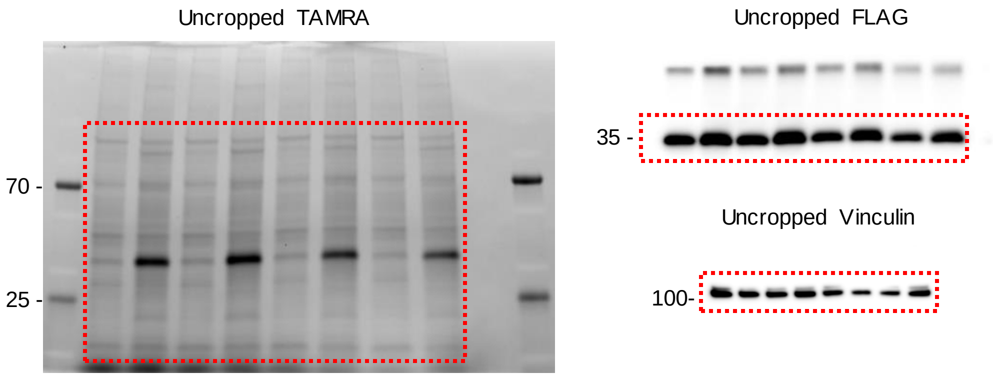

Figure A25. Uncropped gels and westerns for Extended Data Figure 6A. Areas within red borders are within the supplementary figure.

Extended Data Figure 6D

PANC1

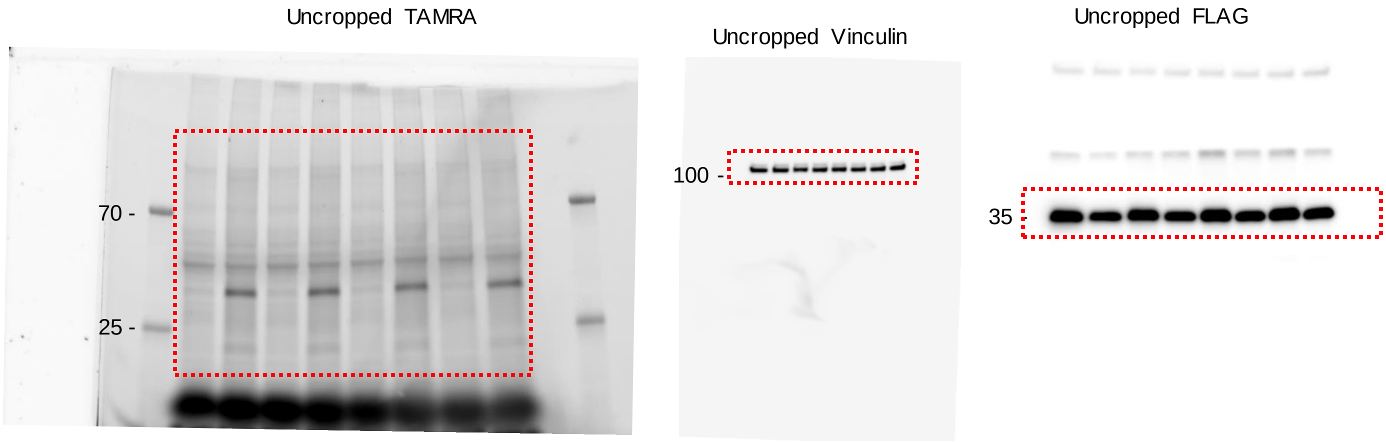

Figure A26. Uncropped gels and westerns for Extended Data Figure 6D. Areas within red borders are in the supplementary figure.

Extended Data Figure 6E

MDA-MB-231

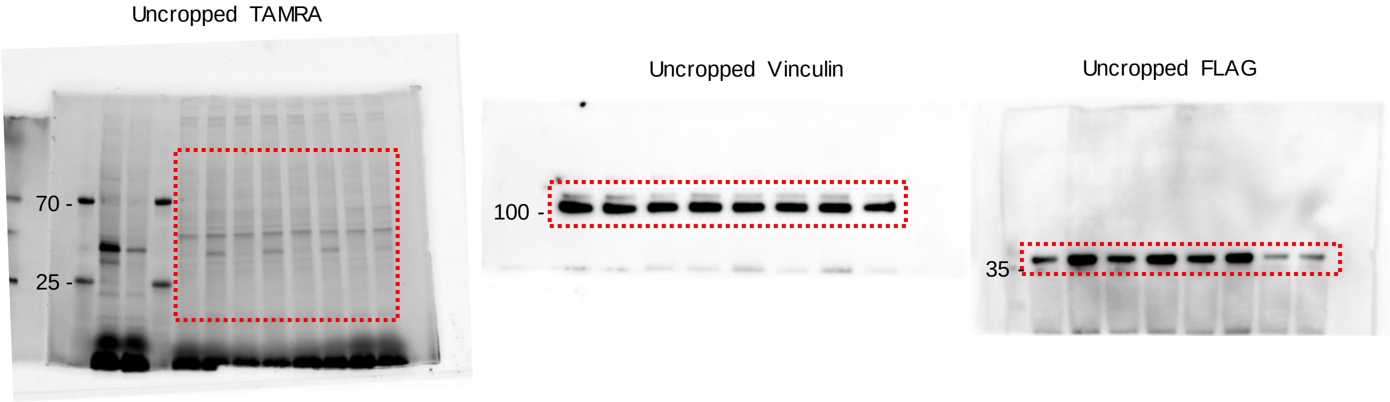

Figure A27. Uncropped gels and westerns for Extended Data Figure 6E. Areas within red borders are in the supplementary figure.

Extended Data Figure 3E

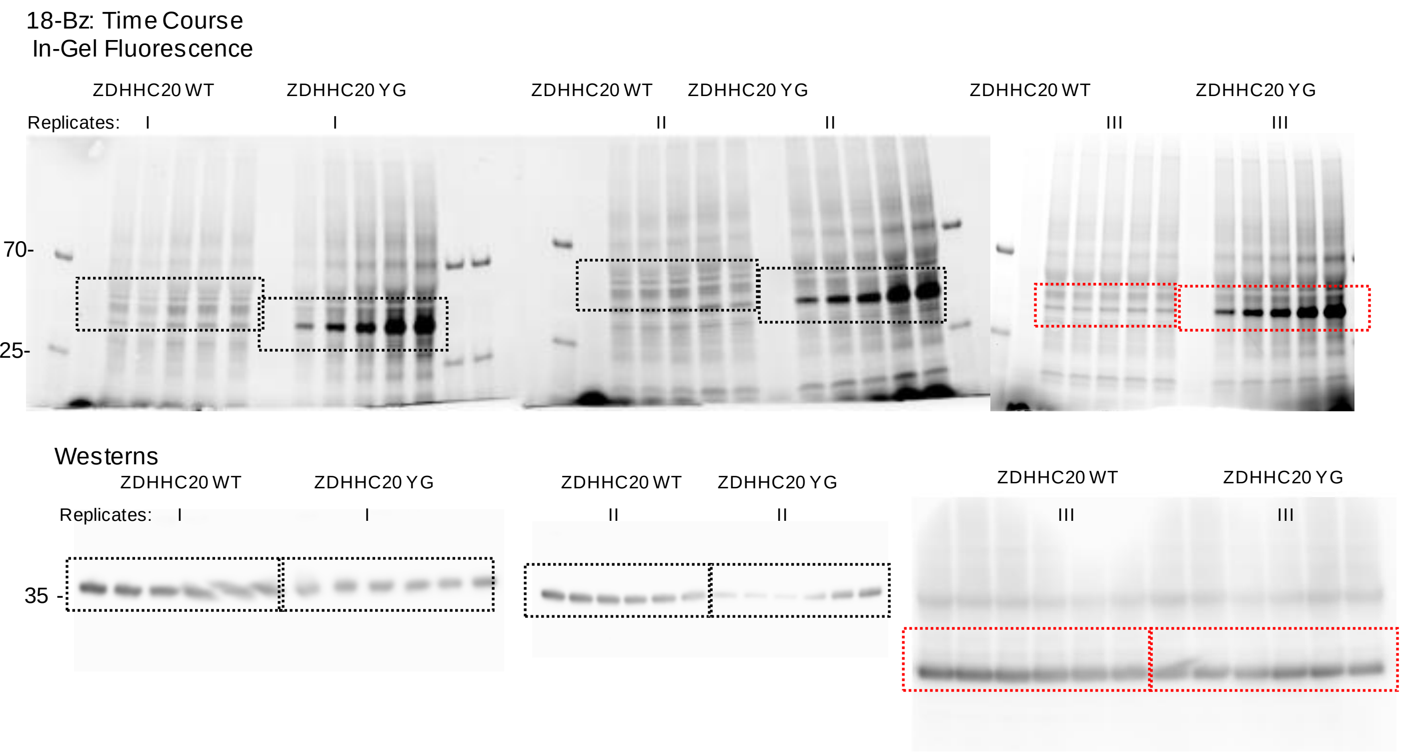

Figure A28. Uncropped gels and westerns for Extended Data Figure 3E. Areas within red borders are in the supplementary figure.

Extended Data Figure 3G

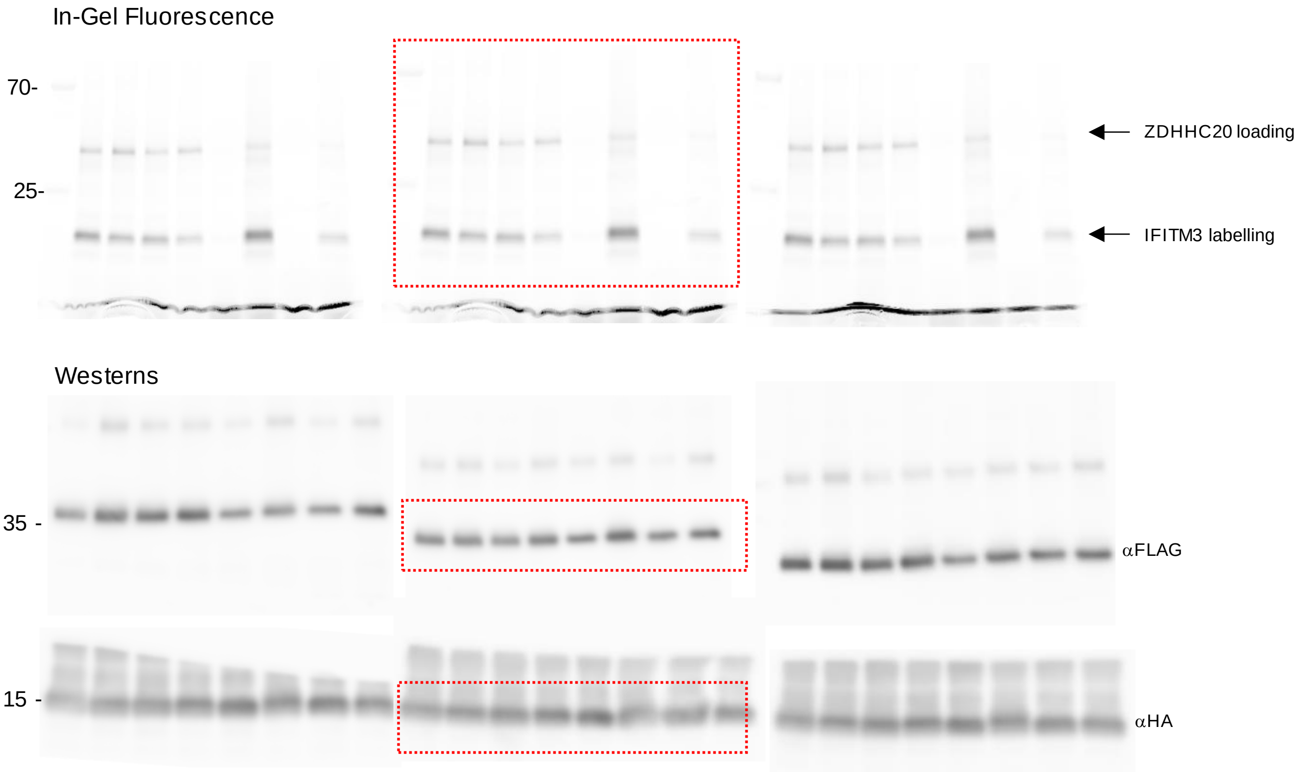

Figure A29. Uncropped gels and westerns for Extended Data Figure 3G. Areas within red borders are in the supplementary figure.

Extended Data Figure 6G

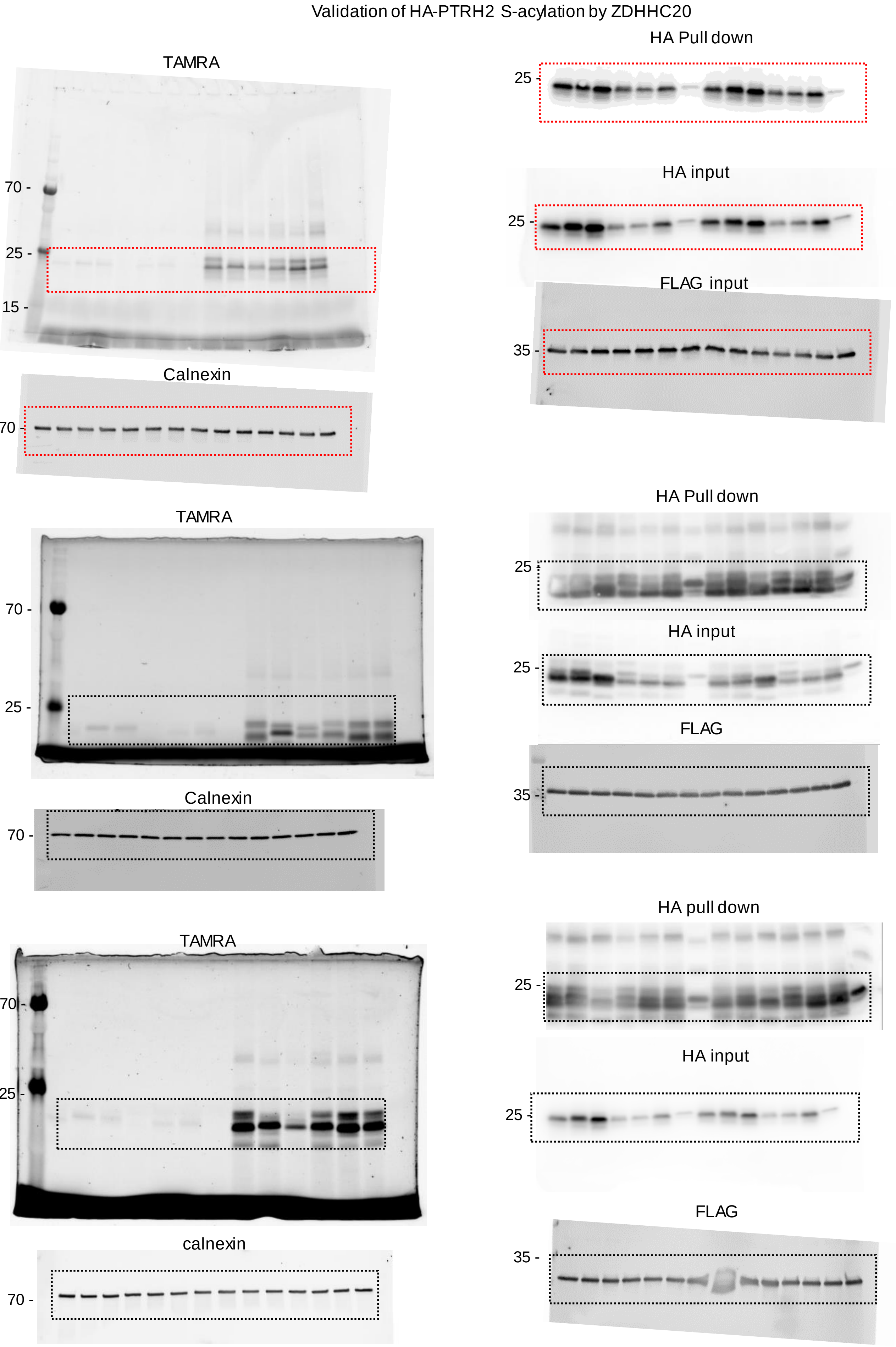

Figure A30. Uncropped gels and westerns for Extended Data Figure 6G. Areas within red borders are in the supplementary figure.

Extended Data Figure 7A

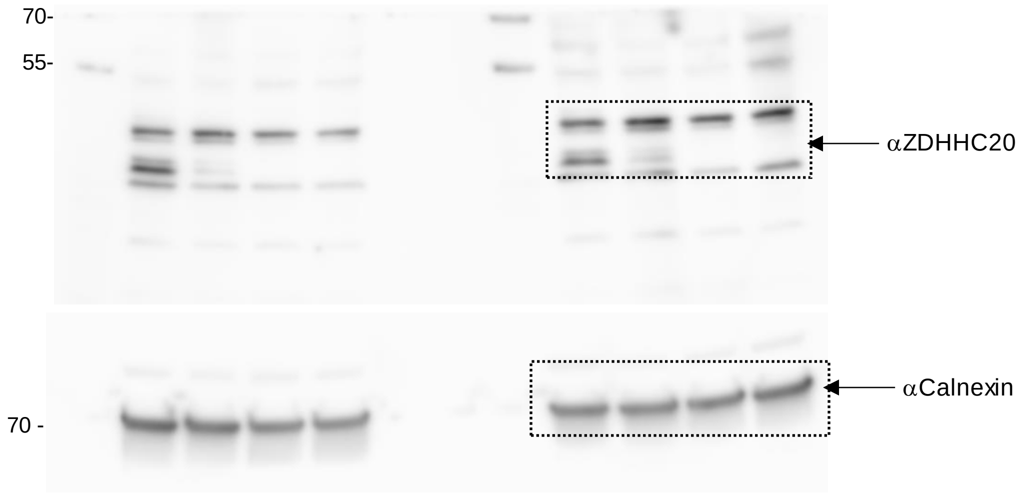

Figure A31. Uncropped westerns for Extended Data Figure 7A. Areas within red borders are in the supplementary figure.

Extended Data Figure 7B

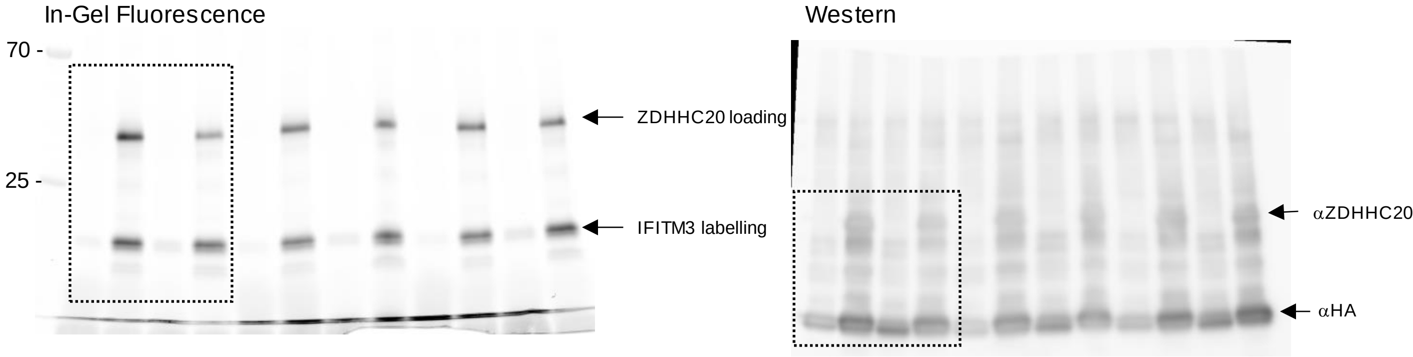

Figure A32. Uncropped gels and westerns for Extended Data Figure 7B. Areas within red borders are in the supplementary figure.

Extended Data Figure 7D

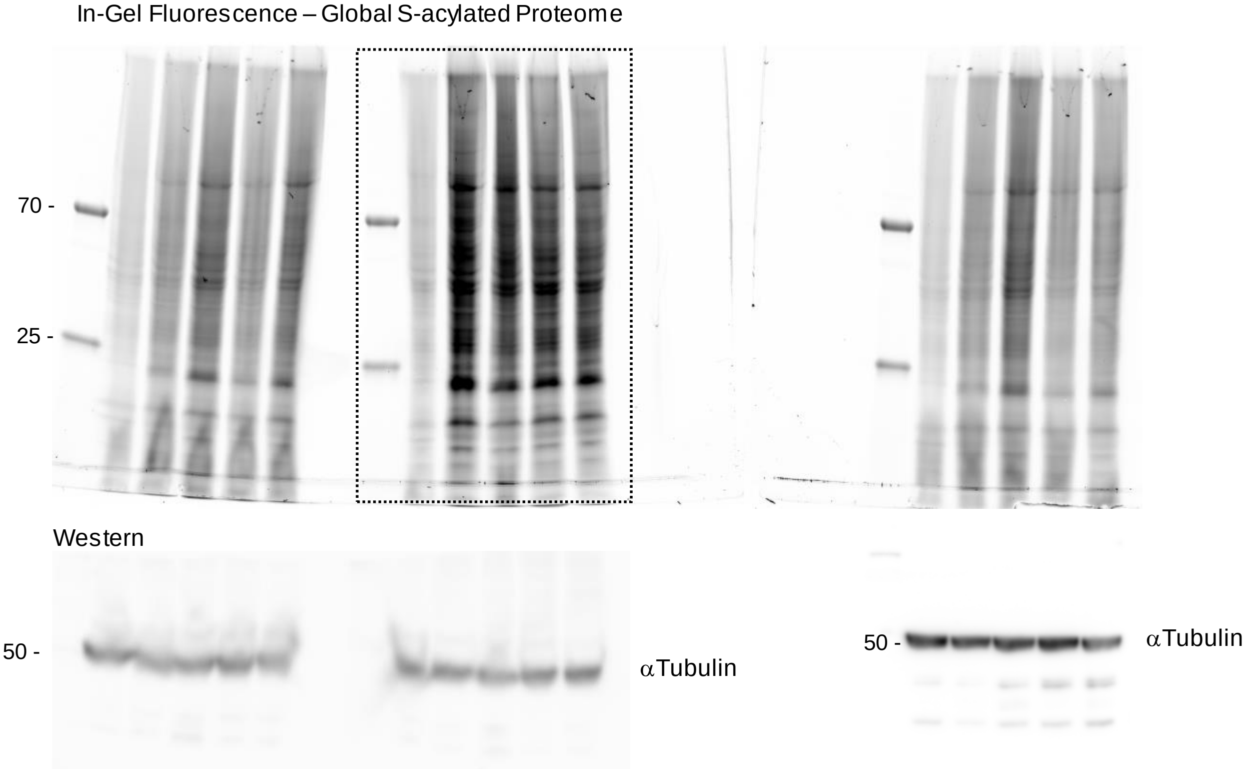

Figure A33. Uncropped gels Extended Data Figure 7D. Areas within red borders are in the supplementary figure.

Extended Data Figure 8B

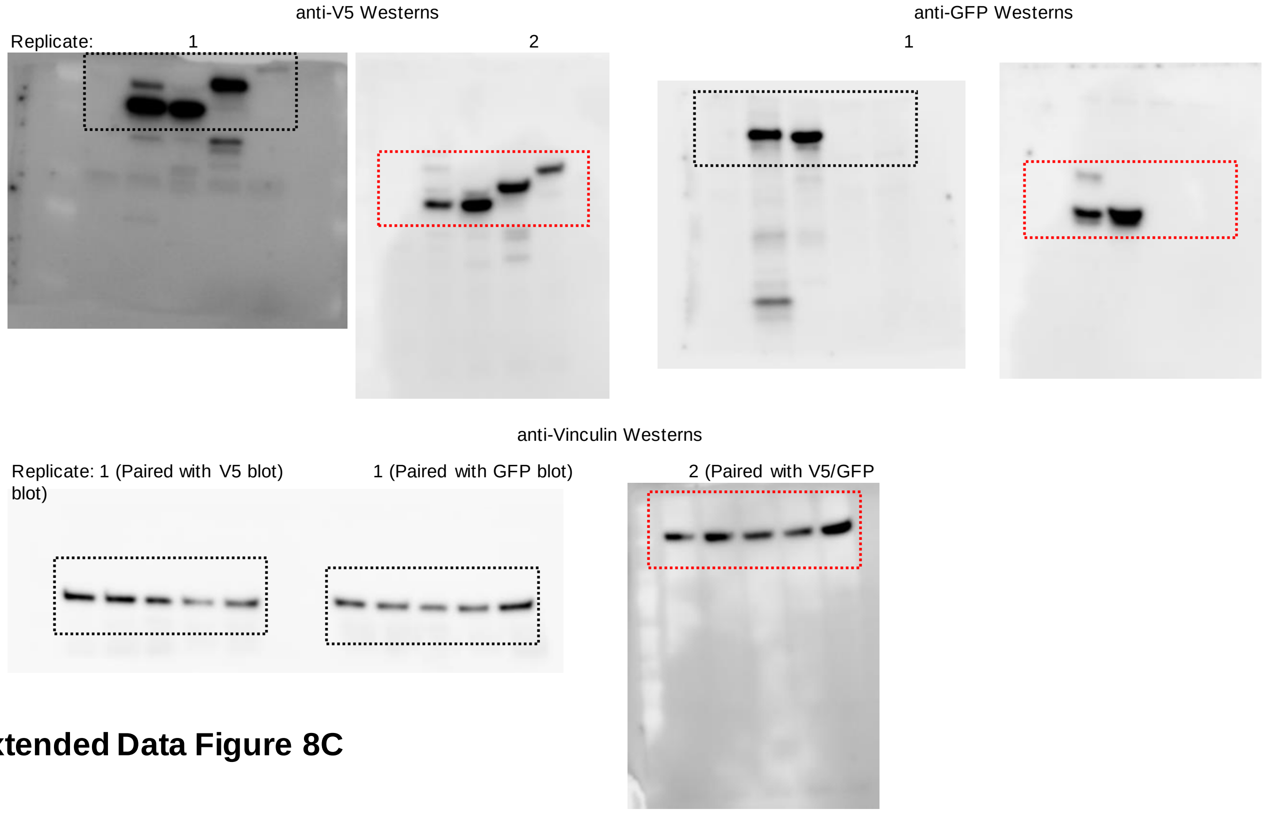

Extended Data Figure 8C

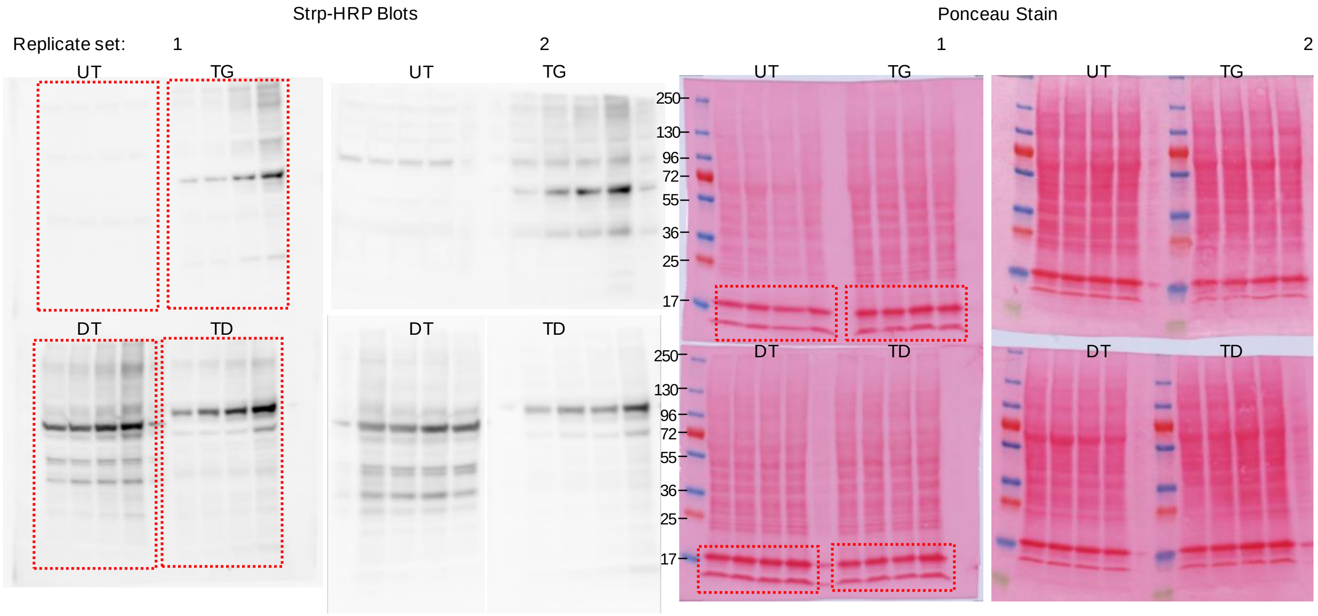

Figure A34. Uncropped westerns and blots for Extended Data Figure 8B-C. Areas within red borders are in the supplementary figure.

Extended Data Figure 9A-G

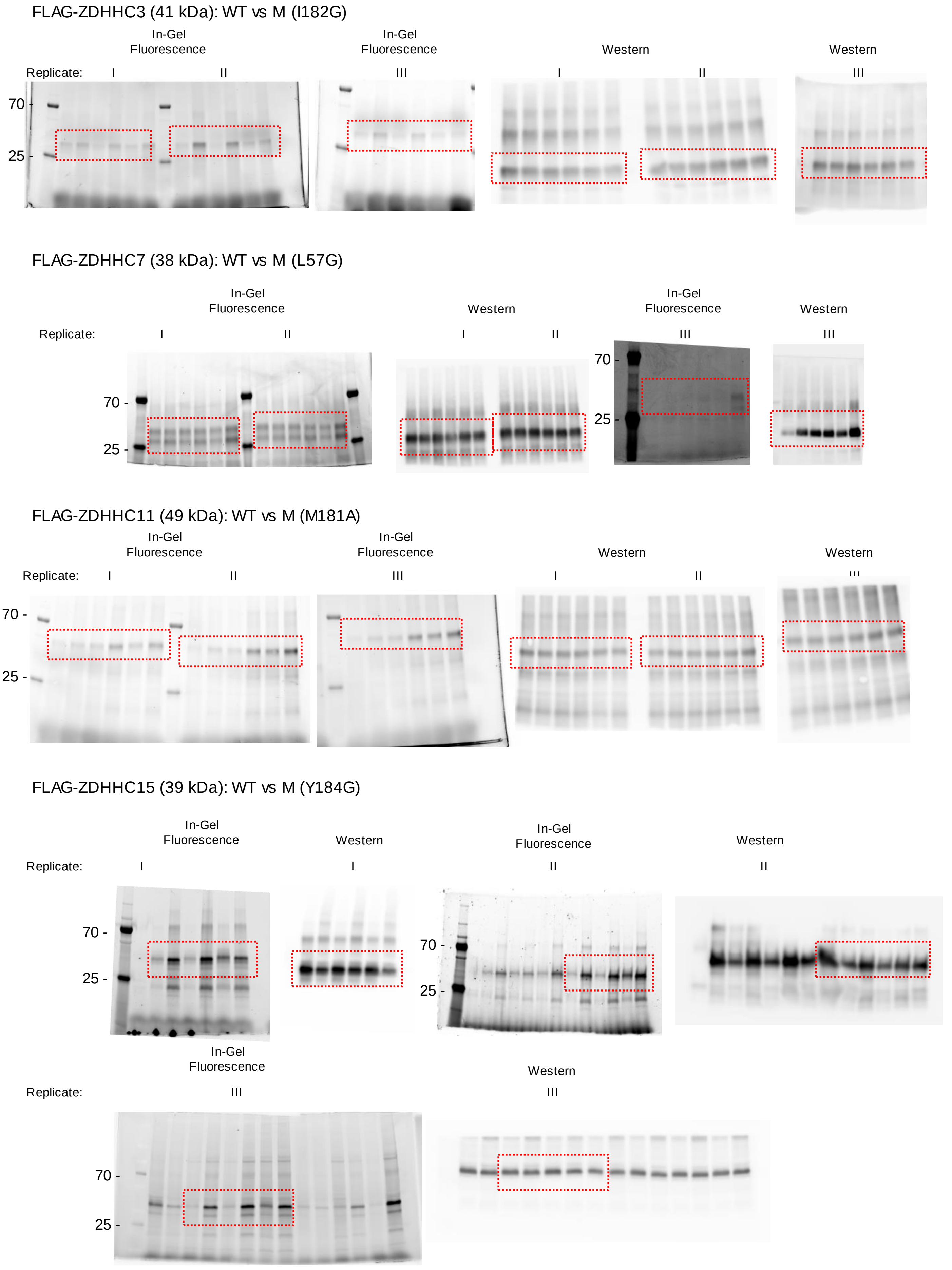

Figure A35. Uncropped gels and westerns for Extended Data Figure 9A-G. Areas within red borders are in the supplementary figure.

Extended Data Figure 9l

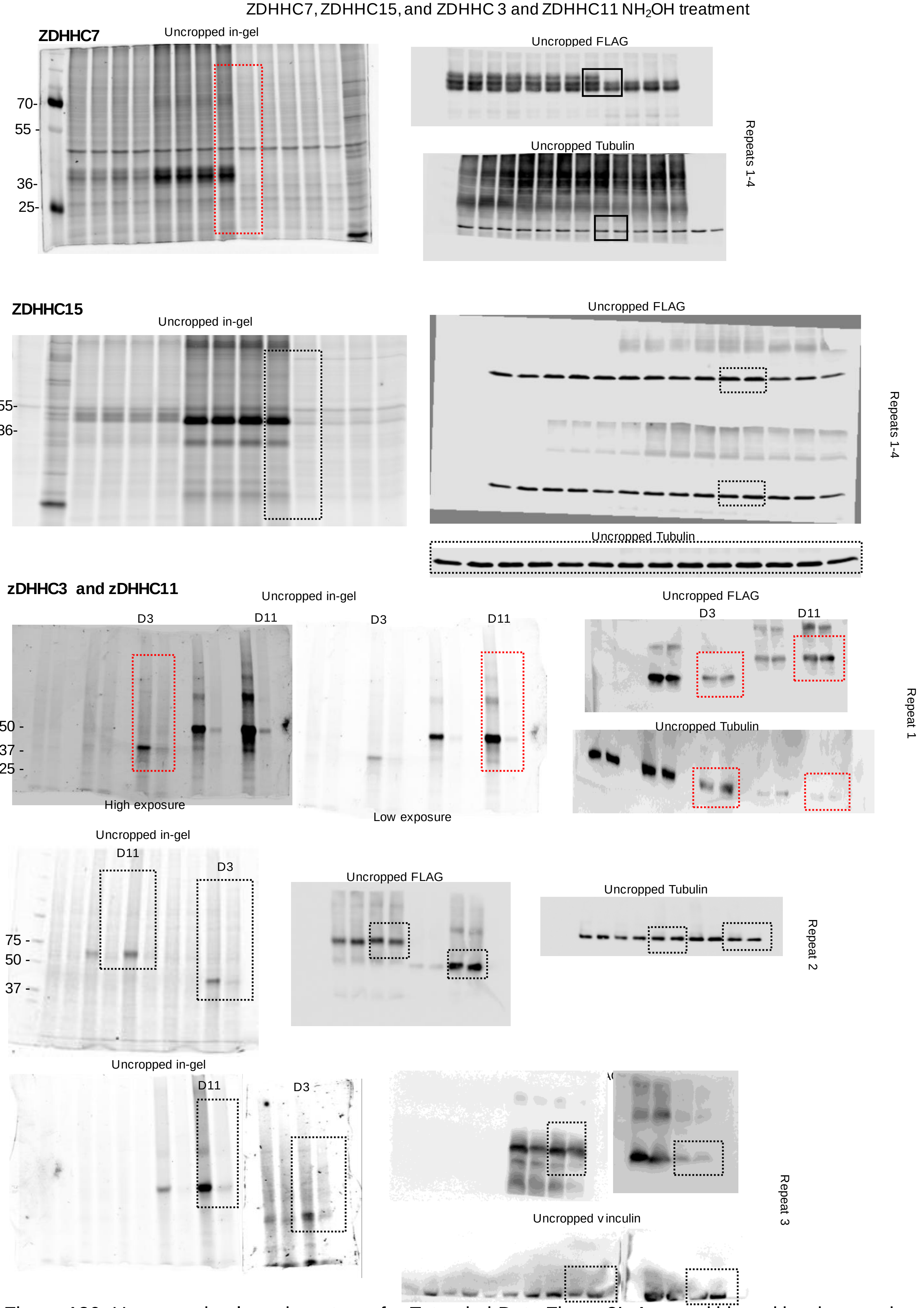

Figure A36. Uncropped gels and westerns for Extended Data Figure 8l. Areas within red borders are in the supplementary figure.

Extended Data Figure 10 A-E

10A

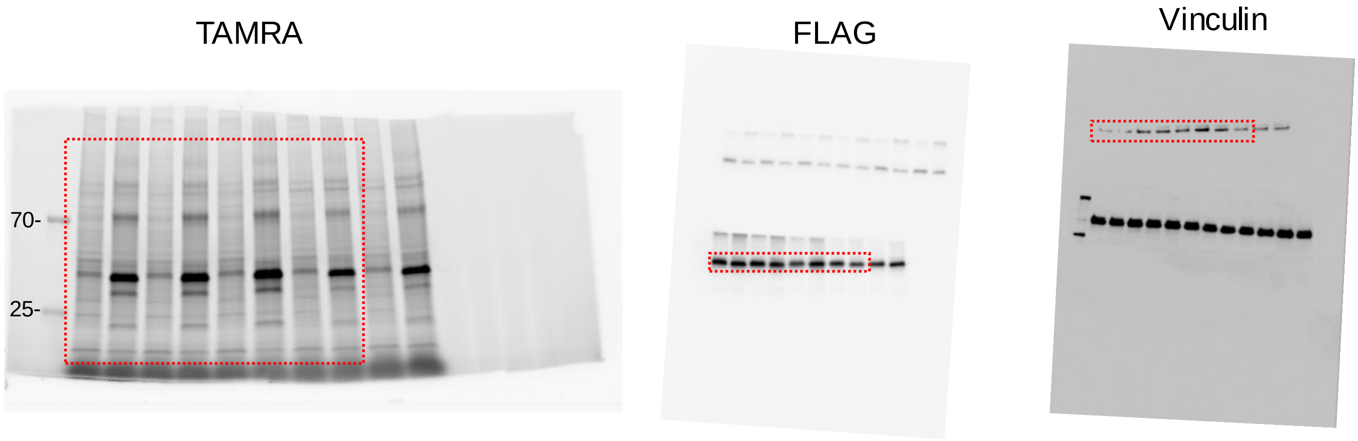

Figure A37. Uncropped gels and westerns for Extended Data Figure 9A. Areas within red borders are in the supplementary figure.

10C

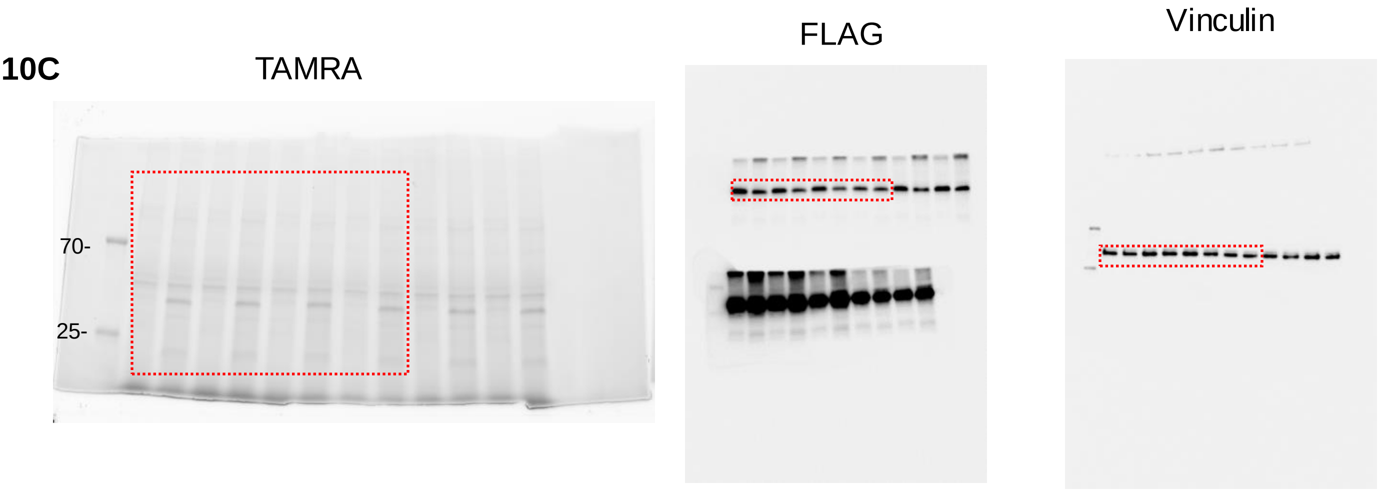

Figure A38. Uncropped gels and westerns for Extended Data Figure 9C. Areas within red borders are in the supplementary figure.

10E

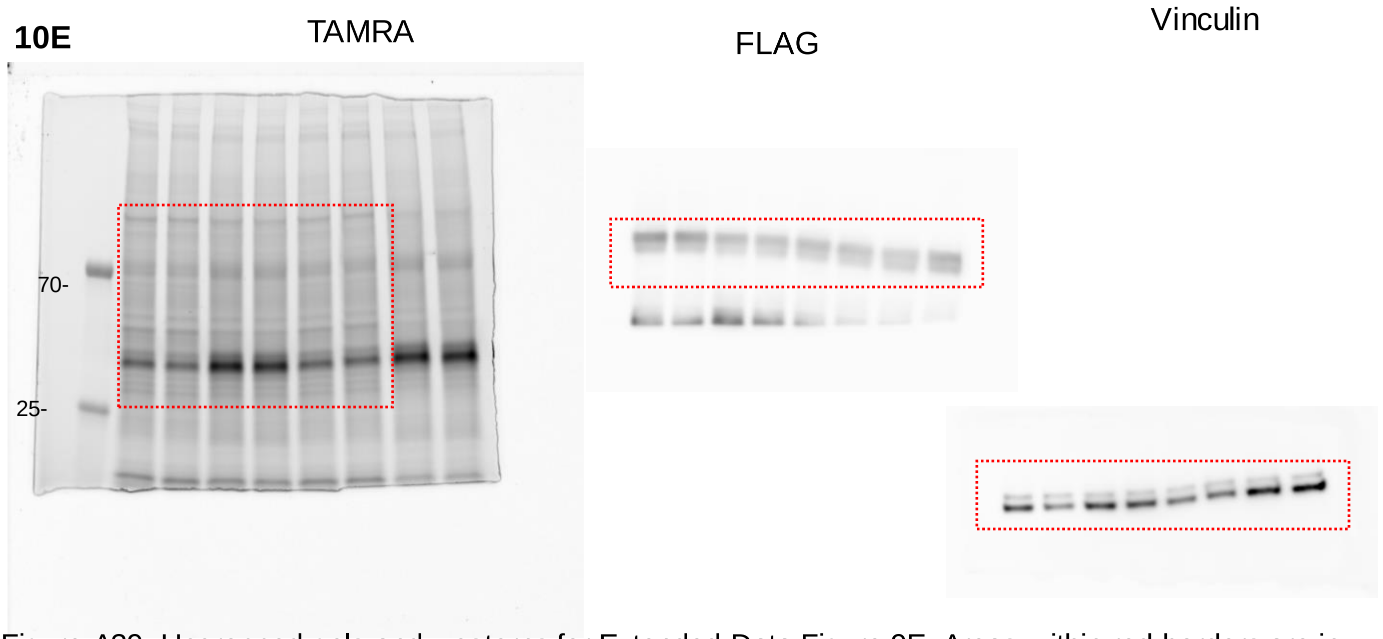

Figure A39. Uncropped gels and westerns for Extended Data Figure 9E. Areas within red borders are in the supplementary figure.
